# Enantioselective S_N_2 Alkylation of Homoenolates by N‐Heterocyclic Carbene Catalysis

**DOI:** 10.1002/advs.202303517

**Published:** 2023-08-04

**Authors:** En Li, Kai Tang, Zhuhui Ren, Xiaoyun Liao, Qianchen Liu, Yong Huang, Jiean Chen

**Affiliations:** ^1^ Pingshan Translational Medicine Center Shenzhen Bay Laboratory Shenzhen 518118 China; ^2^ College of Pharmacy Shenzhen Technology University Shenzhen 518118 China; ^3^ Department of Chemistry The Hong Kong University of Science and Technology Clear Water Bay Kowloon Hong Kong SAR 999077 China

**Keywords:** enantioselective β‐alkylation, homoenolate, N‐heterocyclic carbene, S_N_2 strategy

## Abstract

The functionalization of the β‐carbon of enals with electrophiles is a signature umpolung reactivity of N‐heterocyclic carbene (NHC) derived homoenolates. However, only a limited number of electrophiles are shown to be compatible, with most of them being *π*‐electrophiles. In this study, the successful enantioselective *β*‐alkylation of homoenolates is reported using *C*_sp3_ electrophiles through an S_N_2 strategy. The protocol shows a broad scope regarding alkyl electrophiles, delivering good yields, and excellent enantioselectivities (up to 99% ee). It enables the installation of drug‐like structural motifs in either enals or alkylating agents, demonstrating its potential as a valuable tool for late‐stage modification. Furthermore, a concise synthetic route is presented to chiral pyrroloindoline‐type skeletons. Preliminary mechanistic studies support a direct S_N_2 mechanism.

## Graphical abstract

The presented work describes a novel S_N_2 reaction mode for NHC‐mediated homoenolate chemistry, enabling enantioselective β‐alkylation, allylation, and propargylation of enals. By combining with a “transient acyl trap” strategy, a diverse collection of chiral oxindole derivatives can be accessed, offering an efficient synthetic entry to the pyrroloindoline motif frequently encountered in natural products.

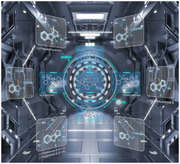

## 1 Introduction

**Figure 1:**
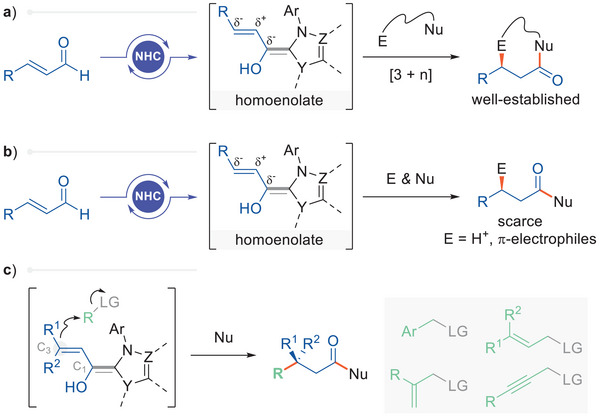
β‐Functionalization of homoenolates. a) Typical reactivity of NHC‐derived homoenolates. b) Non‐cyclizing β‐functionalization of homoenolates. c) Intermolecular S_N_2 Reaction with Csp^3^‐electrophiles (*this work*).

Chiral N‐heterocyclic carbene (NHC) catalysis enables a straightforward route to generate β‐chiral carboxylic acid derivatives through enantioselective electrophilic β‐functionalization of enals.^[^ ^1^ ^]^ The reactions involve a conjugated Breslow intermediate, commonly known as the homoenolate, which reverses the innate polarity of the β‐carbon from electrophilic to nucleophilic. Typically, electrophilic β‐functionalization reactions employ a bifunctional reagent (E···Nu) to generate cyclic products, taking advantage of the intramolecular displacement of the NHC catalyst (by the nucleophilic warhead on the electrophile) for efficient catalyst turnover (**Figure** **1**a).^[^ ^1^, ^2^ ^]^ In comparison, non‐cyclizing β‐functionalization reactions have been restricted to a few electrophiles. Among these, β‐protonation reactions have been the most extensively studied, with Scheidt and our group independently devising practical protocols for enantioselective β‐protonation of enals.^[^ ^3^ ^]^ Additionally, nitroolefins have been shown to undergo smooth β‐alkylation.^[^ ^4^ ^]^ In terms of β‐arylation, Walsh, and Mao have reported the use of homoenolates under synergistic NHC/Pd catalysis, though the asymmetric version remains unreported.^[^ ^5^ ^]^ Notably, the limited carbon electrophiles reported to undergo β‐functionalization have primarily been soft π‐electrophiles (Figure 1b).^[^ ^6^ ^]^ In stark contrast, hard electrophiles, such as alkylating agents, have yet to succeed in functionalizing homoenolates. One mechanistic complication of hard electrophiles is that they may react with the hard nucleophile added to accomplish NHC catalyst turnover, with alcohols being the most frequently used. In addition, C1 versus C3 selectivity, as well as the control of facial selectivity, has not been well established for hard electrophiles. Herein, we report our findings on direct S_N_2‐type alkylation of homoenolates using a broad scope of hard alkylating reagents (Figure 1c).

## 2 Results and Discussion

### 2.1 Condition Survey

**Figure 2:**
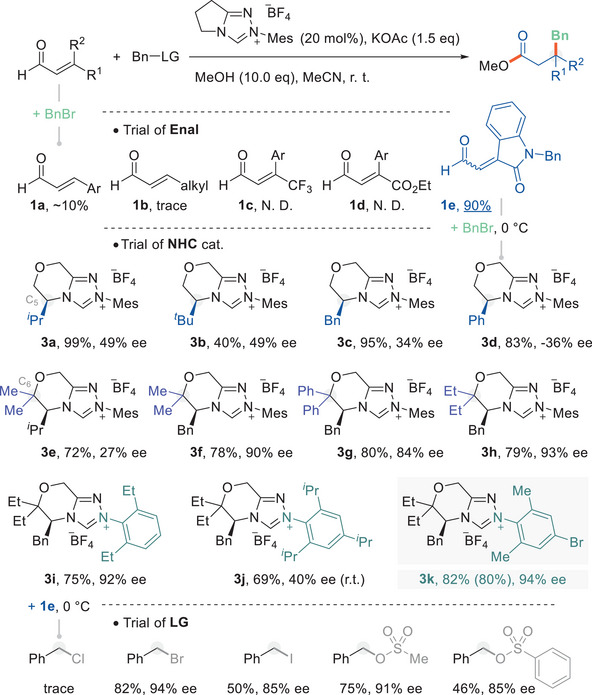
Conditions of the enantioselective S_N_2 reaction mode of homoenolates. Reactions were performed using enal (**1**, 0.1 mmol), Bn‐LG (**2**, 0.15 mmol), an NHC precursor (0.02 mmol), KOAc (0.15 mmol) and MeOH (1.0 mmol) in MeCN (1.0 mL) at the indicated temperature for 12 h. Yields were estimated by the crude NMR integration. The number in parentheses reflected the isolated yield. Values of ee were determined by chiral HPLC.

We began our investigation of this concept using benzyl bromide as an S_N_2 partner and methanol as a turnover agent to evaluate the enal substrates (**Figure** **2**). Potassium acetate, a weak inorganic base, was used to discourage potential β‐protonation. The results showed that the target β‐alkylation failed to dominate the reaction pathway with the exception of **1e**, an enal analog derived from isatin,^[^ ^6^, ^7^ ^]^ obtained in 90% yield. The β‐aryl enal **1a** could afford the detectable product; however, the β‐protonation pathway still dominated, which was the same as the classical β, β‐disubstituted enal **1c** and **1d**.^[^ ^3c,e^ ^]^ Conjecture was that the exocyclic C═C bond tethered to a lactam motif was inclined toward the S_N_2 pathway. Based on this result, this reaction was attempted using chiral NHCs and showed that the morpholinone‐type triazolium compound is a potent catalyst (see Supporting Information for more details). Fine‐tuning the structure of the chiral center (C_5_‐position) offered a response to the reaction performance involving efficiency and enantioselectivity (cat. **3a**–**3d**). The increased steric hindrance suppressed the reaction, and **3b** delivered a 40% yield with 49% ee. The control of the chirality diminished in contrast case, and **3c** gave a yield of 95%, 34% ee. The Thorpe‐Ingold effect was introduced by installing a geminal disubstituent on the C_6_‐position, further adjusting the ring conformation to maintain a balance between reaction efficiency and facial selectivity (cat. **3e**–**3h**). The catalyst **3h** gave the desired product with a good yield and ee value. We also tried to understand the N‐substituent effect of the triazolium moiety, and a hybrid‐type^[^ ^8^ ^]^ analogue **3k** was confirmed as the most effective (80% isolated yield, 94% ee). The LG‐variants of S_N_2 partners were also subjected to a preliminary investigation. The results indicated that the protocol was potent for most cases (─Br, ─I, ─OMs, ─O─phenylsulfonyl) except for chloride.

### 2.2 Substrates Scope

**Figure 3:**
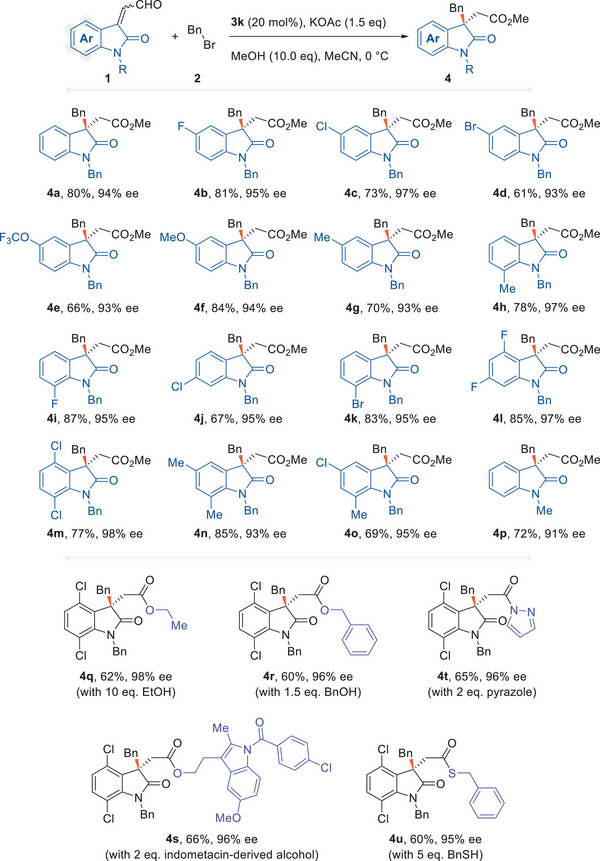
Scope of isatin‐derived enals. Reactions were performed using enal (**1**, 0.1 mmol), BnBr (0.15 mmol), NHC precursor (**3k**, 0.02 mmol), KOAc (0.15 mmol), and MeOH (1.0 mmol) in MeCN (1.0 mL) at 0 °C for 12 h. Yields are of isolated products. Ee was determined by chiral HPLC.

The scope of isatin‐derived enals when benzyl bromide was used as an S_N_2 partner was examined under the optimized reaction conditions (**Figure** **3**). At the four free sites (C_4_─C_7_) of the aromatic ring in isatin, diverse functional groups with different electronic properties, including the electron‐withdrawing groups F, Cl, Br, OCF_3_, and the electron‐donating groups Me and OMe were assembled on the enal substrate and showed broad adaptability of the protocol. The products (**4a**–**4k**) were produced with an average isolated yield of 75% and up to 97% ee. Several disubstituted enal analogues were also tolerated, giving products (**4l**–**4o**) similar yields and enantioselectivity. The substitution patterns of the N atom were then evaluated, and it was found that benzyl, methyl (**4q**), allyl, and even an unprotected ─NH were tolerated (vide infra). With different NHC‐turnover reagents, this reaction could afford diverse carboxylic derivatives in a one‐pot reaction. With the standard conditions, methyl carboxylates were produced when using methanol, and ethanol and benzyl alcohol were also proved to react appropriately, giving products **4q**–**4r**. Another primary alcohol with a more complex structure, an indomethacin analogue, could also deliver the target product **4s** consistently. Additionally, benzyl mercaptan and pyrazole were also compatible with affording corresponding *β*‐chiral thioester and amide (**4t**, **4u**). Based on the transient acyl trap (TAT) strategy previously demonstrated by our group,^[^ ^3^, ^6^ ^]^ acyl pyrazole might be an intermediate to trigger further transacylation. In subsequent verification, methylamine can indeed be used as a final turnover reagent to afford the target amide product (vide infra).

**Figure 4:**
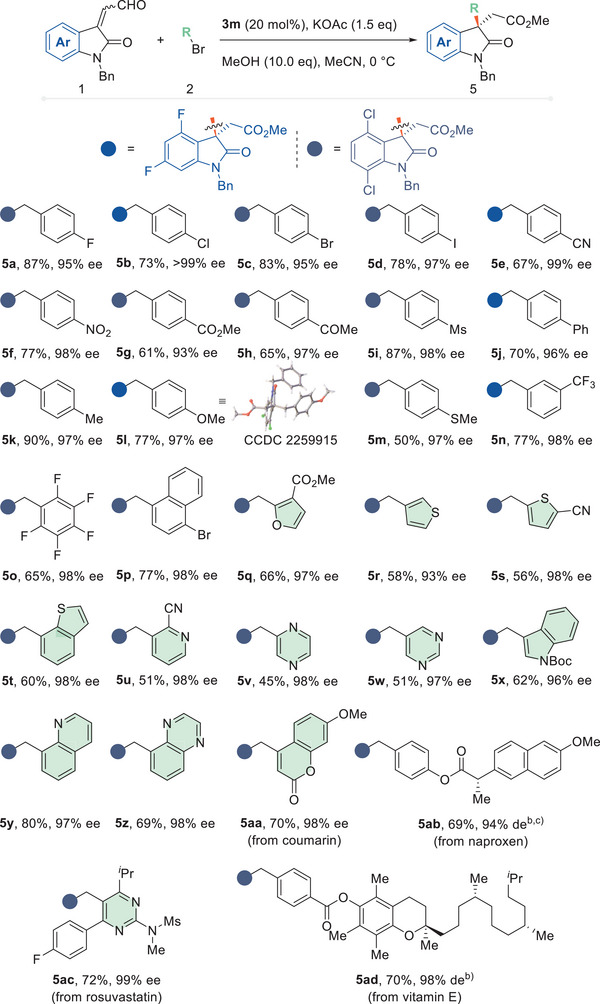
Scope of benzyl bromide. a) Reactions were performed using enal (**1**, 0.1 mmol), bromide (**2**, 0.15 mmol), NHC precursor (**3k**, 0.02 mmol), KOAc (0.15 mmol), and MeOH (1.0 mmol) in MeCN (1.0 mL) at 0 °C for 12 h. Yields are of isolated products. Ee was determined by chiral HPLC. b) DCM was used as a solvent. c) The reaction was carried out at room temperature.

Different benzylic bromides were then evaluated as alkylation reagents under standard conditions (**Figure** **4**). This catalytic protocol tolerates benzyl bromides with various aromatic substituents. Electron‐rich and electron‐poor functional groups did not affect the reaction performance, giving products **5a**–**5p**. The absolute configuration of this series of products was assigned as *S* by X‐ray crystallographic analysis of product **5l** (CCDC 2 259 915). The aryl ring might also be an electron‐rich heteroaromatic ring such as furan or thiophene, which gave products **5q**–**5t** in good yields and high ee values. Various nitrogen‐containing heterocyclic compounds were also tolerated, giving products **5u**–**5z**. This series of compounds containing basic sites are usually excluded from transition metal‐mediated catalyzed reactions but can serve as suitable alkylation reagents with this S_N_2 protocol. In examining the scope of the reaction, several bromides were found to give moderate yields. A competitive pathway could occur in which the acetate ion from the base was involved in a direct nucleophilic attack on the C─Br bond. Some structurally complex and biologically active motifs involving coumarin (**5aa**), naproxen (**5ab**), rosuvastatin (**5ac**) or vitamin E (**5ad**) were installed as side chains and survived the standard reaction well. This exhibited a potential for this methodology to serve as a late‐stage modification method.

**Figure 5:**
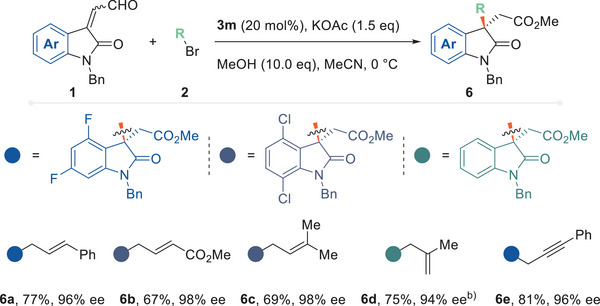
Other categories of S_N_2 partners. a) Reactions were performed using enal (**1**, 0.1 mmol), bromide reagent (**2**, 0.15 mmol), NHC precursor (**3k**, 0.02 mmol), KOAc (0.15 mmol), and MeOH (1.0 mmol) in MeCN (1.0 mL) at 0 °C for 12 h. Yields are of isolated products. Ee was determined by chiral HPLC. b) K_2_CO_3_ (0.15 mmol) was used instead.

Other two types of S_N_2 partners were then used in the enantioselective allylation and propargylation reactions (**Figure** **5**). These transformations were investigated via a synergy of NHC and transition metal catalysis,^[^ ^6a,b^ ^]^ and a solely NHC‐mediated pathway has not been realized. At this stage, we hoped to achieve the universal nature of a broader spectrum of alkylation precursor reagents. The linear and branched allylic bromides were tolerated to afford the same product (**6a**), with the reaction of the branched bromides proceeding through an S_N_2’ mechanism. The ester‐tethered, electron‐poor C═C bond and the trisubstituted, electron‐rich C═C bond produced the linear alkylation product with good yields and enantioselectivities (products **6b**, **6c**). When 3‐bromo‐2‐methylpropene was used, a product bearing a terminal alkene could be formed (**6e**). The enantioselective propargylation could also be realized using the same methodology, with 3‐bromo‐1‐phenylpropyneas as a representative reagent (**6e**).

### 2.3 Application and Derivatization

**Figure 6:**
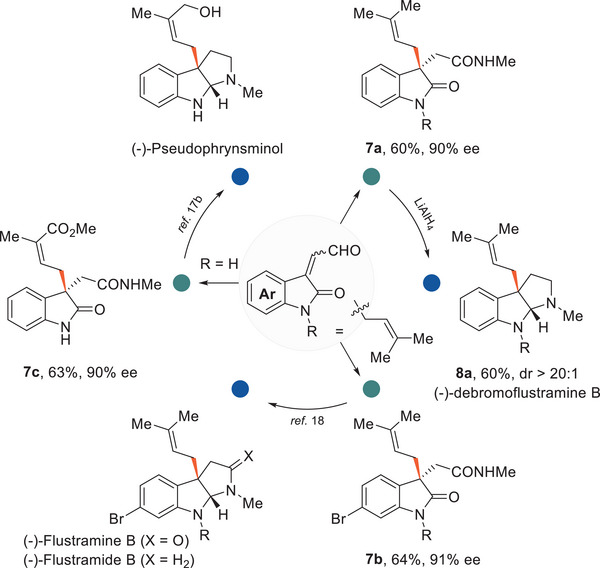
Total synthesis of the pyrroloindoline‐type natural product from enal.

The resulting oxindole product could be a vital synthon for interesting pyrroloindoline skeletons with a broad spectrum of biological activities.^[^ ^9^ ^]^ This encouraged us to synthesize this natural framework using this methodology (**Figure** **6**). Debromoflustramine B was previously demonstrated to have significant butyrylcholinesterase inhibitory activity.^[^ ^10^ ^]^ The N‐isoprenyl‐protected isatin‐derived enals were designated as the starting materials. With the aforementioned transient acyl trap (TAT) strategy, an enantioselective S_N_2 reaction via a homoenolate intermediate proceeded smoothly, affording the target β‐allyl amides (**7a**, **7b**) in 60% yield, 90% ee and 64% yield, 91% ee, respectively. A further reduction with LiAlH_4_ could directly convert these amides to the target pyrroloindoline skeletons, e.g. (−)‐debromoflustramine B (**8a**), in 60% yield, dr > 20:1.^[^ ^11^ ^]^ An N‐unprotected enal was also successfully converted to the amide product (**7c**), which could serve as a precursor for (−)‐pseudophrynsminol (63% and 90% ee).^[^ ^12^ ^]^ This synthetic strategy features a straightforward and efficient method to synthesize these pyrroloindolines from enals in two or three steps.^[^ ^13^ ^]^

### 2.4 Mechanistic Investigation

**Figure 7:**
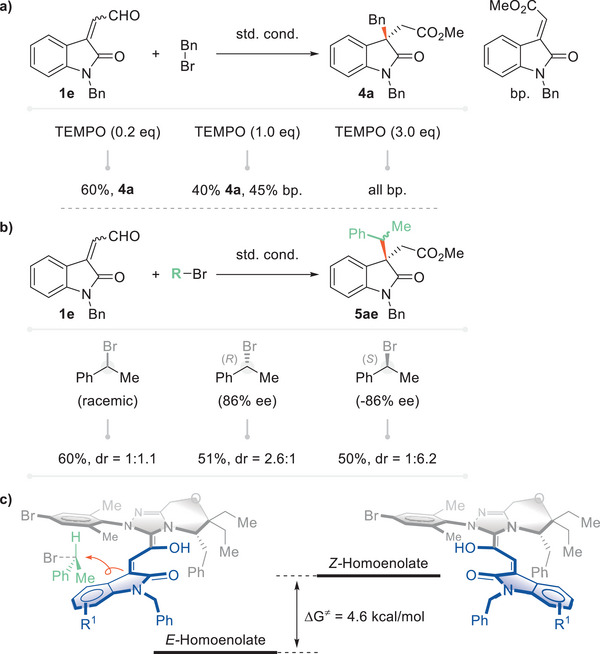
Mechanism experiments.

The mechanism of the reaction was also preliminarily investigated. TEMPO was first applied to decide whether a radical reaction pathway was operating.^[^ ^14^ ^]^ With an increasing amount of up to 1.0 equivalent of TEMPO, the typical S_N_2 reaction still operated at half the efficiency (**4a**, 40% yield), but the reaction was shut down by further increasing the level of TEMPO to 3.0 equivalents and the reported oxidative esterification pathway then dominated. A single‐electron transfer mechanism could be eliminated in this scenario (**Figure** **7**a). Using racemic (1‐bromoethyl)benzene as an S_N_2 partner, the reaction proceeded smoothly to afford a decent yield with diastereoselectivity of 1:1.1. When two chiral secondary bromides (*R* and *S* isomers) were used, the reactions led to nucleophilic substitution products with opposite absolute configurations (Figure 7b, 51%, dr = 2.6:1; 50%, dr = 1:6.2). This indicated a chirality inversion of the secondary bromide, which is consistent with the characteristic of an S_N_2 reaction. A Gibbs free energy calculation suggested that the *E*‐homoenolate intermediate was 4.6 kcal mol^−1^ lower in energy than the *Z*‐type intermediate. The repulsion from the secondary bromide might enable the *S*‐configuration to have the reaction priority (Figure 7c).

**Figure 8:**
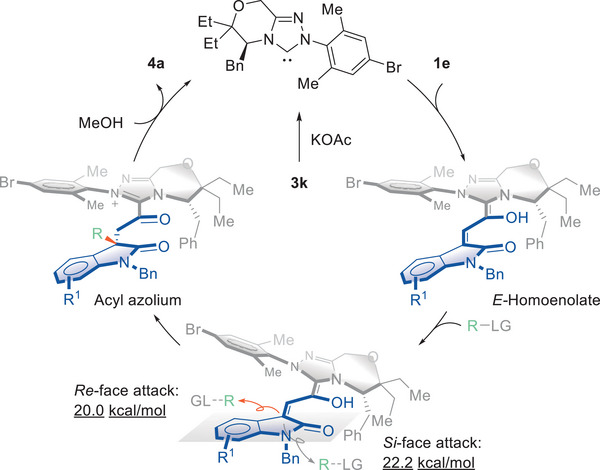
Proposed reaction mechanism.

Here we proposed a catalytic cycle based on mechanistic investigations, which consists of the following steps. The NHC pre‐catalyst **3k** was deprotonated by potassium acetate and formed *E*‐homoenolate with the enal **1e**. The chiral information from the NHC catalyst directed the nucleophilic substitution to occur at the *Re*‐face of the homoenolate, as supported by theoretical calculations that indicated a 2.2 kcal mol^−1^ energy difference between the two approaches (see the Supplementary Information for more details). Then, an acyl azolium intermediate was generated via β‐alkylation, which underwent methanolysis to regenerate the NHC catalyst and afford the desired product (**Figure** **8**).

## 3 Conclusion

In conclusion, we have shown that NHC homoenolates can participate in S_N_2 reactions with hard nucleophiles, such as alkyl halides, mesylates, and tosylates, to afford enantioenriched β‐chiral carboxylates. This is the first example of enantioselective intermolecular S_N_2 reactions in NHC‐mediated homoenolate chemistry. Notably, we found that homoenolates derived from isatin‐based enals are highly reactive toward benzyl, allyl, and propargyl halides, forming chiral carboxylates bearing a quaternary β‐stereocenter. It provides a complementary approach to the current *π*‐electrophiles that can tolerate diverse functional groups. By combining this reaction with a TAT strategy that we previously developed, we could access a diverse collection of chiral oxindole derivatives, offering an efficient synthetic entry to the pyrroloindoline motif frequently encountered in natural products. Preliminary mechanistic studies are consistent with an S_N_2 mechanism for this transformation.

## Conflict of Interest

The authors declare no conflict of interest.

## Supporting information

Supporting Information

## Data Availability

The data that support the findings of this study are available in the supplementary material of this article.
