# Supplementary material for: Enantioselective SN2 Alkylation of Homoenolates by N‐Heterocyclic Carbene Catalysis
Source: Adv Sci (Weinh). 2023 Aug 4;10(29):2303517. doi: 10.1002/advs.202303517 (PMC10582416; doi:10.1002/advs.202303517)
Supplement: Supplementary file 1 — Supporting Information [file ADVS-10-2303517-s001.pdf]

ADVANCED SCIENCE
Open Access

Supporting Information

for *Adv. Sci.*, DOI 10.1002/advs.202303517

Enantioselective S_N2 Alkylation of Homoenolates by N-Heterocyclic Carbene Catalysis

En Li, Kai Tang, Zhuhui Ren, Xiaoyun Liao, Qianchen Liu, Yong Huang and Jiean Chen**

Supporting Information

Enantioselective S_N2 Alkylation of Homoenolates by N-Heterocyclic Carbene Catalysis

En Li, Kai Tang, Zhuhui Ren, Xiaoyun Liao, Qianchen Liu, Yong Huang,* and Jiean Chen*

General methods and materials	2
Synthesis of unreported products	3
Conditions screening	6
Synthesis of alkylation products	9
Characterization of alkylation products	10
Synthetic derivatization	37
X-ray crystal structure	40
Computational data	49
References	65
HPLC traces	66
NMR spectra	125

General methods and materials:

All solvents were distilled according to general practice before use. All reagents were purchased and used without further purification unless specified otherwise. Solvents for flash column chromatography were technical grade and distilled before use. Analytical thin-layer chromatography (TLC) was performed using Huanghai silica gel plates with HSGF 254. The developed chromatogram was visualized by UV absorbance (254 nm) and appropriate stains. Flash column chromatography was performed using the standard techniques of Qingdao Haiyang Chemical HG/T2354-92 silica gel (200-300 mesh) with the indicated solvent system. ^{1}H NMR, ^{13}C NMR and ^{19}F NMR data were recorded on Bruker 400 MHz (101 MHz for ^{13}C, 376MHz for ^{19}F) nuclear resonance spectrometers unless otherwise specified, respectively. Chemical shifts (δ) in ppm are reported as quoted relative to the residual signals of chloroform (^{1}H 7.26 ppm and ^{13}C 77.16 ppm). Multiplicities are described as s (singlet), bs (broad singlet), d (doublet), t (triplet), q (quartet), and m (multiplet), and coupling constants (J) are reported in Hertz (Hz). ^{13}C NMR spectra were recorded with total proton decoupling. Chiral HPLC was recorded on a Thermo Fisher Dionex UltiMate 3000 fast-performance liquid chromatography using Daicel ChiralcelTM columns. HRMS (ESI or APCI) analysis was performed by The Analytical Instrumentation Center at Peking University, Shenzhen Graduate School and (HRMS) data were reported with ion mass/charge (m/z) ratios as values in atomic mass units.

Synthesis of unreported substrates:

1) Synthesis of alcohol derived from indometacin

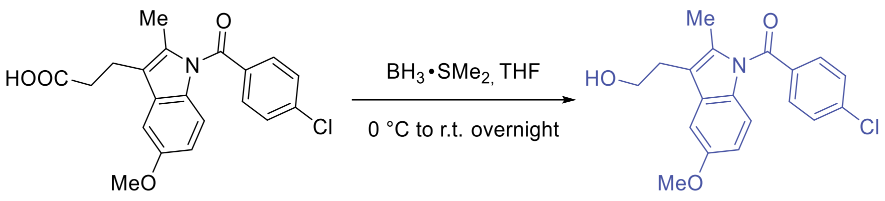

A cooled solution (0 °C) of indometacin (15.0 mmol) in THF (75 mL) was slowly added BH3•SMe2 (9 mL, 2M in THF) dropwise. Then it was allowed to warm to room temperature and stirred overnight, with the reaction progress monitored by TLC. After the reaction, it was slowly quenched by MeOH (16.5 mmol). Then the mixture was concentrated under reduced pressure. The residue was purified by column chromatography to offer the alcohol in 90% yield.

(4-chlorophenyl)(3-(2-hydroxyethyl)-5-methoxy-2-methyl-1H-indol-1-yl)methanone

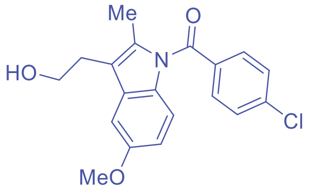

Pale yellow solid. **^{1}H NMR** (400 MHz, $CDCl_3$) δ 7.72 – 7.60 (m, 2H), 7.56 – 7.40 (m, 2H), 6.97 (d, J = 2.6 Hz, 1H), 6.89 (d, J = 9.0 Hz, 1H), 6.68 (dd, J = 9.0, 2.5 Hz, 1H), 3.89 – 3.77 (m, 5H), 2.96 (s, 2H), 2.38 (d, J = 1.5 Hz, 3H). **^{13}C NMR** (101 MHz, $CDCl_3$) δ 168.4, 156.0, 139.2, 135.5, 134.0, 131.1, 131.1, 131.0, 129.1, 116.0, 115.1, 111.4, 101.3, 62.1, 55.8, 27.7, 13.4. **HRMS** (ESI-TOF) [M+H] calculated for $[C_{19}H_{19}ClNO_3]^+$ 344.1048, found 344.1050.

(2) Synthesis of bromides derived from naproxen

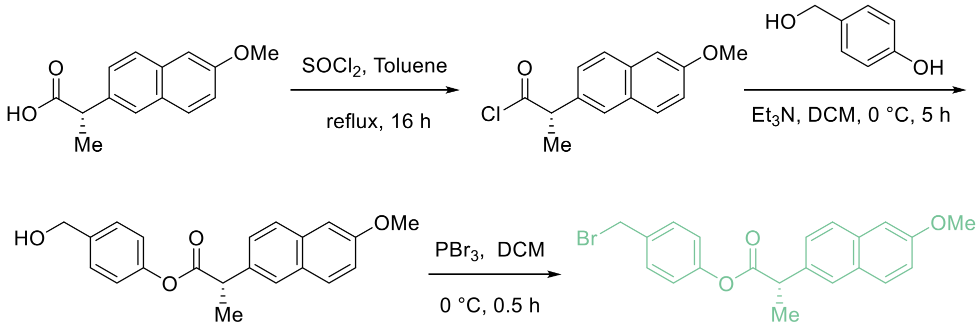

Step 1: To a cooled solution of naproxen (20.0 mmol) in dry toluene (30 mL) was dropwise added $SOCl_2$ (150 mmol). The mixture was heated to reflux for 16 hours. After cooling to room temperature, the mixture was concentrated under reduced pressure. The crude product was used for the next step without further purification.

Step 2: To a cooled solution (0 °C) of 4-(hydroxymethyl)phenol (10.0 mmol) in DCM (30 mL) was added Et_3N (10.0 mmol). The mixture was stirred for 0.5 hours at 0 °C. Then the corresponding acid chloride from step 1 (10.0 mmol) was added dropwise and stirred for 5 hours at 0 °C. Finally, the reaction progress was monitored by TLC. After the reaction, the mixture was washed with Na_2CO_3 (sat.) and brine, dried over Na_2SO_4 and evaporated. The corresponding product was purified by flash column chromatography.

Step 3: To a cooled solution (0 °C) of the alcohol from step 2 (5.0 mmol) in DCM (30 mL) was added PBr_3 (10.0 mmol). The mixture was stirred for 0.5 hours at 0 °C. Then, the reaction progress was monitored by TLC. After the reaction, the mixture was washed with $NaHCO_3$ (sat.) and brine, dried over Na_2SO_4 and evaporated. The corresponding product was purified by flash column chromatography.

4-(bromomethyl)phenyl (S)-2-(6-methoxynaphthalen-2-yl)propanoate

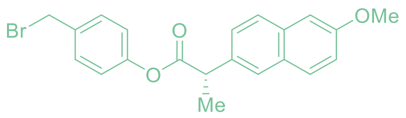

White solid. **^{1}H NMR** (400 MHz, $CDCl_3$) δ 7.89 – 7.64 (m, 3H), 7.52 (dd, J = 8.6, 1.8 Hz, 1H), 7.44 – 7.29 (m, 2H), 7.27 – 7.11 (m, 2H), 7.09 – 6.91 (m, 2H), 4.48 (s, 2H), 4.12 (d, J = 7.1 Hz, 1H), 3.95 (s, 3H), 1.71 (d, J = 7.2 Hz, 3H). **^{13}C NMR** (101 MHz, $CDCl_3$) δ 173.0, 157.8, 150.7, 135.3, 135.0, 133.9, 130.2, 129.3, 129.0, 127.4, 126.2, 126.1, 121.8, 119.2, 105.6, 55.4, 45.6, 32.7, 18.5. **HRMS** (ESI-TOF) [M+H] calculated for $[C_{21}H_{20}BrO_3]^+$ 399.0590, found 399.0591.

(3) Synthesis of bromides derived from D-α-tocopherol

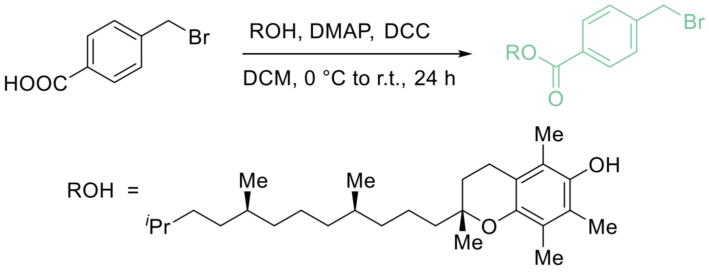

To a cooled solution (0 °C) of the 4-(bromomethyl)benzoic acid (10.0 mmol) in DCM (50 mL) was added alcohol (10.0 mmol), DMAP (1.0 mmol) and DCC (10.0 mmol). The mixture was degassed and back-filled with argon (3x). Then it was stirred at 0 °C for 1 h, allowed to warm to room temperature, and stirred overnight with the reaction progress monitored by TLC. After the reaction, the solution was washed with water and brine, dried over Na_2SO_4 and evaporated. The corresponding product was purified by flash column chromatography.

(R)-2,5,7,8-tetramethyl-2-((4R,8R)-4,8,12-trimethyltridecyl)chroman-6-yl 4-(bromomethyl)benzoate

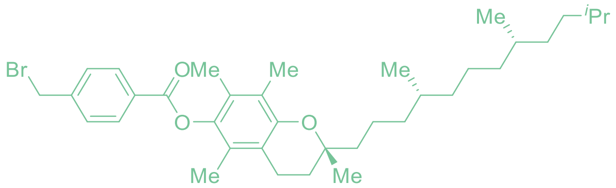

Colorless oil. **^{1}H NMR** (400 MHz, $CDCl_3$) δ 8.51 – 8.05 (m, 2H), 7.73 – 7.45 (m, 2H), 4.57 (s, 2H), 2.65 (t, J = 6.8 Hz, 2H), 2.16 (s, 3H), 2.08 (d, J = 2.0 Hz, 3H), 2.04 (s, 3H), 1.86 (s, 2H), 1.63 – 1.52 (m, 3H), 1.47 – 1.39 (m, 3H), 1.31 (qd, J = 6.8, 3.4 Hz, 12H), 1.21 – 1.07 (m, 6H), 0.89 (t, J = 6.7 Hz, 12H). **^{13}C NMR** (101 MHz, $CDCl_3$) δ 164.6, 149.6, 143.2, 140.6, 130.7, 129.6, 129.3, 126.9, 125.1, 123.2, 117.5, 75.1, 60.4, 40.4, 39.4, 37.5, 37.3, 32.8, 32.7, 32.2, 28.0, 24.8, 24.5, 22.8, 22.7, 21.08, 21.06, 20.7, 19.8, 19.7, 14.2, 13.1, 12.2, 11.9. **HRMS** (ESI-TOF) [M+H] calculated for $[C_{37}H_{56}BrO_3]^+$ 627.3407, found 627.3409.

Conditions screening

Figure S1. Screening of the skeleton of NHC precusor[a]

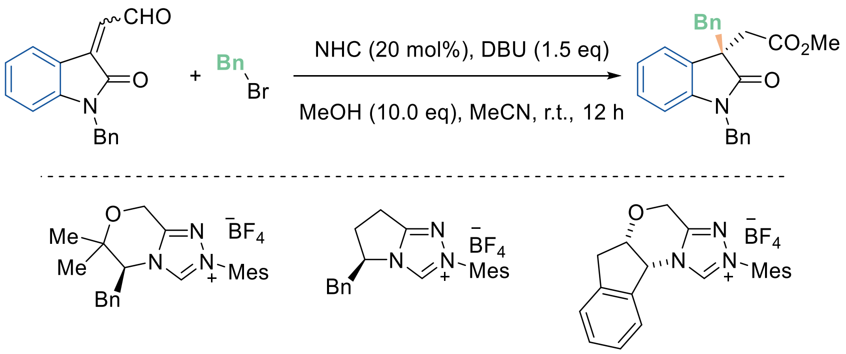

3f, 75%, 77% ee   **3l**, 91%, 30% ee   **3m**, 79%, -25% ee

[a] Reactions were performed using enal **1a** (0.1 mmol), BnBr **2** (0.15 mmol), NHC precursor (0.02 mmol), DBU (0.15 mmol) and MeOH (1.0 mmol) in MeCN (1.0 mL) at room temperature for 12 hours. Yields were estimated by crude NMR integration. Ee values were determined by chiral HPLC.

Figure S2. Screening of temperature[a]

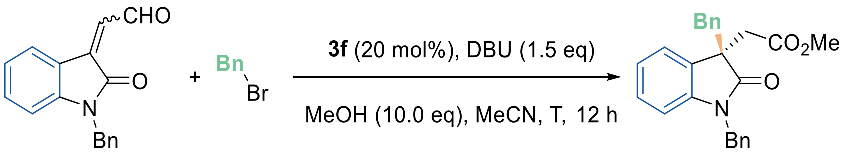

entry	T	yield (%)	ee (%)
1	25	80	78
2	0	78	90
3	-10	50	89
4	-20	27	91

[a] Reactions were performed using enal **1a** (0.1 mmol), BnBr **2** (0.15 mmol), NHC precursor (0.02 mmol), DBU (0.15 mmol) and MeOH (1.0 mmol) in MeCN (1.0 mL) at T for 12 hours. Yields were estimated by crude NMR integration. Ee values were determined by chiral HPLC.

Figure S3. Screening of solvent[a]

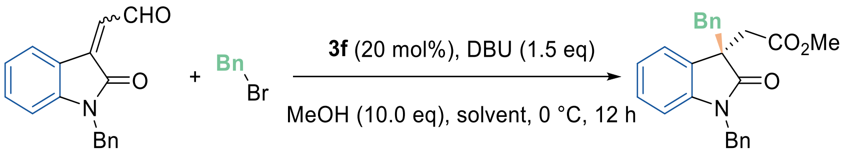

entry	base	yield (%)	ee (%)
1	DCM	88	77
2	$CHCl_3$	85	79
3	DCE	84	77
4	MeCN	66	84
5	DMF	43	76
6	DMSO	N.D.	-

7	dioxane	43	73
8	THF	48	70

[a] Reactions were performed using enal **1a** (0.1 mmol), BnBr **2** (0.15 mmol), NHC precursor **3f** (0.02 mmol), DBU (0.15 mmol) and MeOH (1.0 mmol) in solvent (1.0 mL) at 0 °C for 12 hours. Yields were estimated by crude NMR integration. Ee values were determined by chiral HPLC.

Figure S4. Screening of base[a]

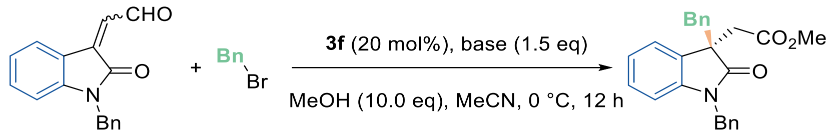

entry	base	yield (%)	ee (%)
1	K_2CO_3	74	90
2	Cs_2CO_3	5%	-
3	KOAc	78	90
4	K_3PO_4	72	89
5	PivONa	41	88
6	K_3PO_4	72	87
7	DBU	75	84
8	TEA	85	82

[a] Reactions were performed using enal **1a** (0.1 mmol), BnBr **2** (0.15 mmol), NHC precursor **3f** (0.02 mmol), base (0.15 mmol) and MeOH (1.0 mmol) in MeCN (1.0 mL) at 0 °C for 12 hours. Yields were estimated by crude NMR integration. Ee values were determined by chiral HPLC.

Figure S5. Screening of the amount of MeOH[a]

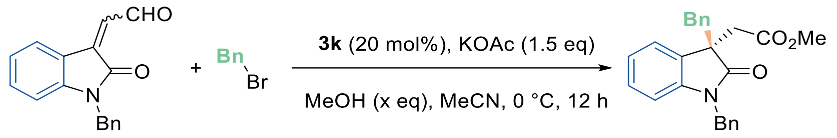

entry	MeOH (x eq)	yield (%)	ee (%)
1	2.5	56	92
2	5	62	92
3	10	80	94
4	20	55	91

[a] Reactions were performed using enal **1a** (0.1 mmol), BnBr **2** (0.15 mmol), NHC precursor **3k** (0.02 mmol), KOAc (0.15 mmol) and MeOH (x eq) in MeCN (1.0 mL) at 0 °C for 12 hours. Yields were estimated by crude NMR integration. Ee values were determined by chiral HPLC.

Figure S6. Screening of the amount of NHC precursor **3k**[a]

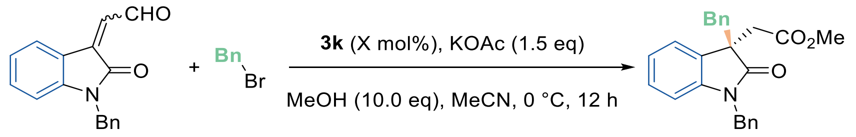

entry	NHC (x mol%)	yield (%)	ee (%)
1	5	71	92
2	10	79	92
3	20	80	94

[a] Reactions were performed using enal **1a** (0.1 mmol), BnBr **2** (0.15 mmol), NHC precursor **3k** (x mol%), KOAc (0.15 mmol) and MeOH (10.0 eq) in MeCN (1.0 mL) at 0 °C for 12 hours. Yields were estimated by crude NMR integration. Ee values were determined by chiral HPLC.

Synthesis of alkylation products

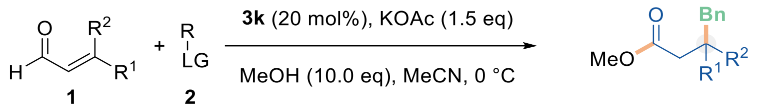

The catalyst precursor **3m** (10.9 mg, 20 mol%), enal 1 (0.1 mmol, 1.0 eq) and KOAc (15 mg, 1.5 eq) were mixed in MeCN (1.0 mL) in a 20-mL test tube. The reaction vessel was degassed and back-filled with argon, sealed with a rubber septum and stirred at 0 °C. for 10 min. Then, RLG **2** (0.15 mmol, 1.5 eq) and MeOH (40 μL, 10.0 eq) were added. And the mixture was stirred at 0 °C for 12 hours. Upon complete consumption of **1**, the reaction was quickly filtered through a plug of silica gel and concentrated. The residue was purified by silica gel flash column chromatography [eluent: hexane (100mL), then hexane/EtOAc = 5:1) to afford corresponding products. The ee was determined by chiral HPLC.

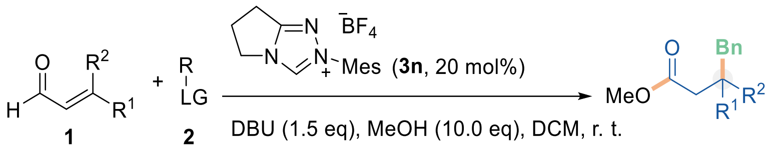

The following procedure prepared racemic samples: enal 1 (0.1 mmol, 1.0 equiv.), the catalyst precursor **3n** (6.2 mg, 20 mol%), DBU (0.15 mmol, 1.5 equiv.), RLG (0.15 mmol, 1.5 equiv.), MeOH (40 μL, 10.0 eq) were dissolved in 1.0 mL of DCM. The mixture was stirred at room temperature for 2 hours, and the solvent was removed under reduced pressure. The crude mixture was purified by flash column chromatography [eluent: hexane (100mL), then hexane/EtOAc = 5:1].

Characterization of alkylation products

methyl (S)-2-(1,3-dibenzyl-2-oxoindolin-3-yl)acetate (**4a**)

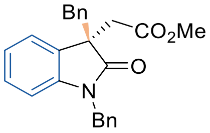

Colorless oil, 80% yield, 94% ee. **^{1}H NMR** (400 MHz, $CDCl_3$) δ 7.25 (dd, J = 7.0, 1.5 Hz, 1H), 7.19 (qd, J = 5.1, 4.6, 1.4 Hz, 4H), 7.14 – 7.02 (m, 4H), 6.91 – 6.78 (m, 4H), 6.43 (dd, J = 7.5, 1.2 Hz, 1H), 4.91 (d, J = 16.0 Hz, 1H), 4.61 (d, J = 16.0 Hz, 1H), 3.46 (s, 3H), 3.26 (d, J = 16.2 Hz, 1H), 3.18 (s, 2H), 3.06 (d, J = 16.2 Hz, 1H). **^{13}C NMR** (101 MHz, $CDCl_3$) δ 178.4, 170.1, 143.6, 135.6, 134.9, 130.2, 130.1, 128.5, 128.3, 127.9, 127.1, 126.8, 126.8, 123.2, 122.1, 109.2, 51.7, 51.4, 43.9, 43.8, 41.2. **HRMS** (ESI-TOF) [M+H] calculated for $[C_{25}H_{24}NO_3]^+$ 386.1751, found 386.1752. **Specific Rotation** $[\alpha]_D^{25}$ = +43.1 (c = 0.60 in CH_2Cl_2). **HPLC** (Chiralpak-IA-H column, isopropanol/hexane = 20/80, 1.0 mL/min): t(minor) = 12.227 min, t(major) = 10.447 min.

methyl (S)-2-(1,3-dibenzyl-5-fluoro-2-oxoindolin-3-yl)acetate (**4b**)

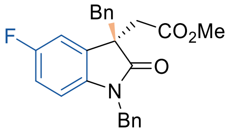

Colorless oil, 81% yield, 95% ee. **^{1}H NMR** (400 MHz, $CDCl_3$) δ 7.25 – 7.14 (m, 4H), 7.12 – 7.05 (m, 2H), 7.04 – 6.94 (m, 4H), 6.89 (ddd, J = 11.3, 7.2, 2.3 Hz, 1H), 6.85 – 6.76 (m, 2H), 4.88 (qd, J = 15.6, 1.1 Hz, 2H), 3.43 (s, 3H), 3.26 (d, J = 16.4 Hz, 1H), 3.13 (d, J = 1.3 Hz, 2H), 3.02 (d, J = 16.4 Hz, 1H). **^{13}C NMR** (101 MHz, $CDCl_3$) δ 178.1, 170.0, 147.3 (d, J = 244.4 Hz), 137.0, 134.4, 133.3 (d, J = 3.5 Hz), 130.2, 130.1, 128.4, 128.0, 127.1, 127.0, 127.0 (d, J = 1.7 Hz), 122.7 (d, J = 6.4 Hz), 119.1 (d, J = 3.2 Hz), 116.5 (d, J = 19.6 Hz), 51.7, 51.6 (d, J = 1.9 Hz), 45.2 (d, J = 4.4 Hz), 44.1, 41.2**.** **^{19}F NMR** (376 MHz, $CDCl_3$) δ -134.09. **HRMS** (ESI-TOF) [M+H] calculated for $[C_{25}H_{23}FNO_3]^+$ 404.1656, found 404.1657. **Specific Rotation** $[\alpha]_D^{25}$ = +38.9 (c = 0.20 in CH_2Cl_2). **HPLC** (Chiralpak-IC-H column, ethanol/hexane = 30/70, 1.0 mL/min): t(minor) = 7.613 min, t(major) = 12.163 min.

methyl (S)-2-(1,3-dibenzyl-5-chloro-2-oxoindolin-3-yl)acetate (**4c**)

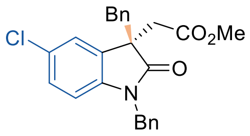 Yellow oil, 73% yield, 97% ee, **^{1}H NMR** (400 MHz, $CDCl_3$) δ 7.25 – 7.16 (m, 5H), 7.16 – 7.09 (m, 2H), 7.06 (dd, J = 8.3, 2.1 Hz, 1H), 6.92 – 6.82 (m, 2H), 6.78 (dd, J = 7.3, 2.3 Hz, 2H), 6.32 (d, J = 8.3 Hz, 1H), 4.87 (d, J = 16.0 Hz, 1H), 4.60 (d, J = 16.0 Hz, 1H), 3.51 (s, 3H), 3.26 (d, J = 16.6 Hz, 1H), 3.15 (d, J = 2.3 Hz, 2H), 3.04 (d, J = 16.6 Hz, 1H). **^{13}C NMR** (101 MHz, $CDCl_3$) δ 178.0, 169.9, 142.2, 135.1, 134.4, 132.1, 130.2, 128.6, 128.2, 128.0, 127.5, 127.3, 127.1, 126.7, 123.5, 110.1, 51.8, 51.6, 43.91, 43.86, 40.9. **HRMS** (ESI-TOF) [M+H] calculated for $[C_{25}H_{23}ClNO_3]^+$ 420.1361, found 420.1361. **Specific Rotation** $[\alpha]_D^{25}$ = +50.4 (c = 0.72 in CH_2Cl_2). **HPLC** (Chiralpak-IC-H column, ethanol/hexane = 30/70, 1.0 mL/min): t(minor) = 7.610 min, t(major) = 11.530 min.

methyl (S)-2-(1,3-dibenzyl-5-bromo-2-oxoindolin-3-yl)acetate (**4d**)

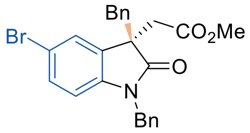 Yellow oil, 61% yield, 93% ee, **^{1}H NMR** (400 MHz, $CDCl_3$) δ 7.35 (d, J = 1.9 Hz, 1H), 7.26 – 7.16 (m, 5H), 7.16 – 7.08 (m, 2H), 6.91 – 6.83 (m, 2H), 6.78 (dd, J = 7.2, 2.4 Hz, 2H), 6.27 (d, J = 8.3 Hz, 1H), 4.86 (d, J = 16.1 Hz, 1H), 4.59 (d, J = 16.1 Hz, 1H), 3.52 (s, 3H), 3.26 (d, J = 16.7 Hz, 1H), 3.14 (d, J = 1.6 Hz, 2H), 3.04 (d, J = 16.7 Hz, 1H). **^{13}C NMR** (101 MHz, $CDCl_3$) δ 177.9, 169.9, 142.7, 135.0, 134.4, 132.5, 131.1, 130.2, 128.6, 128.0, 127.3, 127.1, 126.7, 126.2, 114.8, 110.7, 51.9, 51.5, 43.89, 43.88, 40.9. **HRMS** (ESI-TOF) [M+H] calculated for $[C_{25}H_{23}BrNO_3]^+$ 464.0856, found 464.0858. **Specific Rotation** $[\alpha]_D^{25}$ = +207.1 (c = 0.20 in CH_2Cl_2). **HPLC** (Chiralpak-IC-H column, isopropanol /hexane = 30/70, 1.0 mL/min): t(minor) = 7.643 min, t(major) = 11.463 min.

methyl (S)-2-(1,3-dibenzyl-2-oxo-5-(trifluoromethoxy)indolin-3-yl)acetate (**4e**)

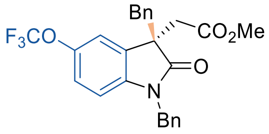 Colorless oil, 66% yield, 93% ee. **^{1}H NMR** (400 MHz, $CDCl_3$) δ 7.27 – 7.16 (m, 4H), 7.15 – 7.06 (m, 3H), 6.96 (ddd, J = 8.5, 2.5, 1.0 Hz, 1H), 6.93 – 6.80 (m, 4H), 6.39 (d, J = 8.5 Hz, 1H), 4.90 (d, J = 16.0 Hz, 1H), 4.65 (d, J = 16.0 Hz, 1H), 3.50 (s, 3H), 3.26 (d, J = 16.5 Hz, 1H), 3.17 (s, 2H), 3.04 (d, J = 16.5 Hz, 1H). **^{13}C**

NMR (101 MHz, $CDCl_3$) δ 178.1, 170.0, 147.3 (d, J = 244.4 Hz), 137.0, 134.4, 133.3 (d, J = 3.5 Hz), 130.2, 130.1, 128.4, 128.0, 127.1, 127.02, 126.98 (d, J = 1.7 Hz), 122.7 (d, J = 6.4 Hz), 119.1 (d, J = 3.2 Hz), 116.5 (d, J = 19.6 Hz), 51.7, 51.6 (d, J = 1.9 Hz), 45.2 (d, J = 4.4 Hz), 44.1, 41.2. **^{19}F NMR** (376 MHz, $CDCl_3$) δ -58.35. **HRMS** (ESI-TOF) [M+H] calculated for $[C_{26}H_{23}F_3NO_4]^+$ 470.1574, found 470.1577. **Specific Rotation** $[\alpha]_D^{25}$ = +20.6 (c = 0.60 in CH_2Cl_2). **HPLC** (Chiralpak-IC-H column, isopropanol/hexane = 30/70, 1.0 mL/min): t(minor) = 5.847 min, t(major) = 8.510 min.

methyl (S)-2-(1,3-dibenzyl-5-methoxy-2-oxoindolin-3-yl)acetate (**4f**)

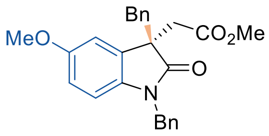

Colorless oil, 84% yield, 94% ee. **^{1}H NMR** (400 MHz, $CDCl_3$) δ 7.26 – 7.15 (m, 4H), 7.11 (t, J = 7.4 Hz, 2H), 6.97 – 6.88 (m, 2H), 6.85 (d, J = 2.5 Hz, 1H), 6.84 – 6.75 (m, 2H), 6.61 (dd, J = 8.5, 2.6 Hz, 1H), 6.31 (d, J = 8.5 Hz, 1H), 4.89 (d, J = 16.0 Hz, 1H), 4.59 (d, J = 16.0 Hz, 1H), 3.78 (s, 3H), 3.48 (s, 3H), 3.25 (d, J = 16.4 Hz, 1H), 3.15 (s, 2H), 3.02 (d, J = 16.4 Hz, 1H). **^{13}C NMR** (101 MHz, $CDCl_3$) δ 178.1, 170.1, 155.5, 137.1, 135.6, 134.9, 131.5, 130.3, 128.5, 127.9, 127.1, 126.9, 126.8, 112.3, 110.8, 109.5, 55.8, 51.7, 51.7, 43.9, 41.1. **HRMS** (ESI-TOF) [M+H] calculated for $[C_{26}H_{26}NO_4]^+$ 416.1856, found 416.1858. **Specific Rotation** $[\alpha]_D^{25}$ = +126.3 (c = 1.0 in CH_2Cl_2). **HPLC** (Chiralpak-IC-H column, isopropanol/hexane = 30/70, 1.0 mL/min): t(minor) = 13.463 min, t(major) = 21.673 min.

methyl (S)-2-(1,3-dibenzyl-5-methyl-2-oxoindolin-3-yl)acetate (**4g**)

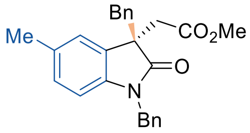

Yellow oil, 70% yield, 93% ee, **^{1}H NMR** (400 MHz, $CDCl_3$) δ 7.23 – 7.15 (m, 4H), 7.10 (t, J = 7.5 Hz, 2H), 7.05 (d, J = 1.7 Hz, 1H), 6.93 – 6.84 (m, 3H), 6.84 – 6.74 (m, 2H), 6.30 (d, J = 7.9 Hz, 1H), 4.88 (d, J = 16.0 Hz, 1H), 4.59 (d, J = 16.0 Hz, 1H), 3.47 (s, 3H), 3.24 (d, J = 16.3 Hz, 1H), 3.15 (s, 2H), 3.03 (d, J = 16.3 Hz, 1H). **^{13}C NMR** (101 MHz, $CDCl_3$) δ 178.3, 170.2, 141.1, 135.7, 135.0, 131.5, 130.27, 130.1, 128.6, 128.5, 127.8, 127.0, 126.8, 126.8, 123.9, 108.9, 51.7, 51.4, 43.9, 43.8, 41.1, 21.2. **HRMS** (ESI-TOF) [M+H] calculated for $[C_{26}H_{26}NO_3]^+$ 400.1907, found 400.1905. **Specific Rotation** $[\alpha]_D^{25}$ = +86.7 (c = 0.50 in CH_2Cl_2). **HPLC**

(Chiralpak-IC-H column, isopropanol/hexane = 30/70, 1.0 mL/min): t(minor) = 11.280 min, t(major) = 18.437 min.

methyl (S)-2-(1,3-dibenzyl-7-methyl-2-oxoindolin-3-yl)acetate (**4h**)

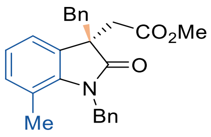

Yellow oil, 78% yield, 97% ee, **^{1}H NMR** (400 MHz, $CDCl_3$) δ 7.26 – 7.16 (m, 4H), 7.12 (ddd, J = 7.4, 4.8, 3.3 Hz, 3H), 6.75 – 6.66 (m, 2H), 5.08 (d, J = 17.1 Hz, 1H), 4.89 (d, J = 17.1 Hz, 1H), 3.52 (s, 3H), 3.25 (d, J = 16.2 Hz, 1H), 3.16 (s, 2H), 3.04 (d, J = 16.2 Hz, 1H), 2.04 (s, 3H). **^{13}C NMR** (101 MHz, $CDCl_3$) δ 179.3, 170.2, 137.9, 135.0, 132.3, 130.9, 130.3, 128.7, 127.9, 126.9, 126.7, 125.5, 122.2, 121.0, 119.6, 51.7, 50.7, 45.1, 44.4, 41.5, 18.5. **HRMS** (ESI-TOF) [M+H] calculated for $[C_{26}H_{26}NO_3]^+$ 400.1907, found 400.1906. **Specific Rotation** $[\alpha]_D^{25}$ = +87.9 (c = 0.20 in CH_2Cl_2). **HPLC** (Chiralpak-OD-H column, ethanol/hexane = 20/80, 1.0 mL/min): t(minor) = 6.987 min, t(major) = 8.957 min.

methyl (S)-2-(1,3-dibenzyl-7-fluoro-2-oxoindolin-3-yl)acetate (**4i**)

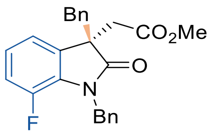

Yellow oil, 87% yield, 95% ee, **^{1}H NMR** (400 MHz, $CDCl_3$) δ 7.24 – 7.15 (m, 4H), 7.08 (dd, J = 8.4, 6.9 Hz, 2H), 7.04 – 6.95 (m, 4H), 6.89 (ddd, J = 11.4, 7.2, 2.4 Hz, 1H), 6.84 – 6.79 (m, 2H), 4.88 (qd, J = 15.6, 1.1 Hz, 2H), 3.43 (s, 3H), 3.25 (d, J = 16.3 Hz, 1H), 3.18 – 3.09 (m, 2H), 3.02 (d, J = 16.3 Hz, 1H). **^{13}C NMR** (101 MHz, $CDCl_3$) δ 178.1, 167.0, 147.25 (d, J = 244.3 Hz), 137.9, 134.4, 133.29 (d, J = 3.6 Hz), 130.1, 128.4, 127.9, 127.1, 127.0, 126.99, 126.97, 122.73 (d, J = 6.4 Hz), 119.07 (d, J = 3.2 Hz), 116.54 (d, J = 19.6 Hz), 51.71, 51.61 (d, J = 1.9 Hz), 45.23 (d, J = 4.4 Hz), 44.13, 41.16. **^{19}F NMR** (376 MHz, $CDCl_3$) δ -134.08. **HRMS** (ESI-TOF) [M+H] calculated for $[C_{25}H_{23}FNO_3]^+$ 404.1656, found 404.1658. **Specific Rotation** $[\alpha]_D^{25}$ = +29.8 (c = 0.60 in CH_2Cl_2). **HPLC** (Chiralpak-IC-H column, isopropanol/hexane = 30/70, 1.0 mL/min): t(minor) = 7.873 min, t(major) = 12.813 min.

methyl (S)-2-(1,3-dibenzyl-6-chloro-2-oxoindolin-3-yl)acetate (**4j**)

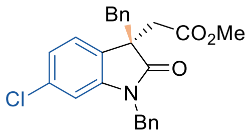

Yellow solid, 67% yield, 95% ee, **^{1}H NMR** (400 MHz, $CDCl_3$) δ 7.21 (tt, J = 4.8, 2.0 Hz, 4H), 7.16 – 7.07 (m, 3H), 7.02 (dd, J = 7.9, 1.8 Hz, 1H), 6.94 – 6.75 (m, 4H), 6.42 (d, J = 1.9 Hz, 1H), 4.87 (d, J = 16.0 Hz, 1H), 4.59 (d, J = 16.0 Hz, 1H), 3.48 (s, 3H), 3.25 (d, J = 16.4 Hz, 1H), 3.14 (s, 2H), 3.03 (d, J = 16.4 Hz, 1H). **^{13}C NMR** (101 MHz, $CDCl_3$) δ 178.4, 170.0, 144.8, 135.0, 134.5, 134.0, 130.2, 128.7, 128.6, 128.0, 127.4, 127.1, 126.7, 124.0, 122.0, 109.8, 51.8, 51.1, 43.9, 43.8, 41.0. **HRMS** (ESI-TOF) [M+H] calculated for $[C_{25}H_{23}ClNO_3]^+$ 420.1361, found 420.1365. **Specific Rotation** $[\alpha]_D^{25}$ = -13.1 (c = 0.48 in CH_2Cl_2). **HPLC** (Chiralpak-IC-H column, isopropanol/hexane = 30/70, 1.0 mL/min): t(minor) = 7.587 min, t(major) = 10.593 min.

methyl (S)-2-(1,3-dibenzyl-7-bromo-2-oxoindolin-3-yl)acetate (**4k**)

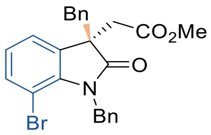

Colorless oil, 83% yield, 95% ee, **^{1}H NMR** (400 MHz, $CDCl_3$) δ 7.26 – 7.15 (m, 4H), 7.12 (ddd, J = 7.4, 4.7, 1.7 Hz, 3H), 6.98 (t, J = 7.5 Hz, 1H), 6.94 – 6.88 (m, 1H), 6.88 – 6.77 (m, 2H), 6.71 (ddd, J = 6.2, 3.4, 1.6 Hz, 2H), 5.08 (d, J = 17.1 Hz, 1H), 4.89 (d, J = 17.1 Hz, 1H), 3.52 (s, 3H), 3.25 (d, J = 16.2 Hz, 1H), 3.16 (s, 2H), 3.04 (d, J = 16.2 Hz, 1H), 2.04 (s, 3H). **^{13}C NMR** (101 MHz, $CDCl_3$) δ 179.3, 170.2, 137.9, 135.0, 132.3, 130.9, 130.3, 128.6, 127.9, 126.9, 126.7, 125.5, 122.2, 121.0, 119.6, 51.7, 50.7, 45.1, 44.4, 41.5, 18.5. **HRMS** (ESI-TOF) [M+H] calculated for $[C_{25}H_{23}BrNO_3]^+$ 464.0856, found 464.0858. **Specific Rotation** $[\alpha]_D^{25}$ = -7.3 (c = 0.90 in CH_2Cl_2). **HPLC** (Chiralpak-IC-H column, isopropanol/hexane = 30/70, 1.0 mL/min): t(minor) = 9.330 min, t(major) = 25.373 min.

methyl (S)-2-(1,3-dibenzyl-4,6-difluoro-2-oxoindolin-3-yl)acetate (**4l**)

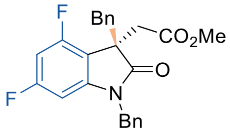

Colorless oil, 85% yield, 97% ee, **^{1}H NMR** (400 MHz, $CDCl_3$) δ 7.25 – 7.15 (m, 4H), 7.15 – 7.05 (m, 2H), 6.95 – 6.87 (m, 2H), 6.83 – 6.73 (m, 2H), 6.47 (td, J = 9.6, 2.1 Hz, 1H), 5.92 (dd, J = 8.6, 2.1 Hz, 1H), 4.83 (d, J = 16.1 Hz, 1H), 4.56 (d, J = 16.1 Hz, 1H), 3.52 (s, 3H), 3.29 (s, 2H), 3.28 – 3.18 (m, 2H). **^{13}C NMR** (101 MHz, $CDCl_3$) δ 178.0, 170.0, 163.30 (dd, J = 247.2, 13.0 Hz), 158.51 (dd, J = 246.5, 14.3 Hz),

146.87 – 145.51 (m), 134.6, 134.5, 129.68, 128.7, 128.1, 127.4, 127.0, 126.7, 110.92 (dd, J = 19.7, 3.1 Hz), 100.59 – 96.42 (m), 94.53 (dd, J = 27.6, 3.6 Hz), 51.9, 51.34 (d, J = 2.5 Hz), 44.3, 42.2, 40.5. **^{19}F NMR** (376 MHz, $CDCl_3$) δ -107.81 (d, J = 8.8 Hz), -118.19 (d, J = 17.0 Hz). **HRMS** (ESI-TOF) [M+H] calculated for $[C_{25}H_{22}F_2NO_3]^+$ 422.1562, found 422.1563. **Specific Rotation** $[\alpha]_D^{25}$ = +56.1 (c = 0.20 in CH_2Cl_2). **HPLC** (Chiralpak-IC-H column, isopropanol/hexane = 30/70, 1.0 mL/min): t(minor) = 5.637 min, t(major) = 7.347 min.

methyl (S)-2-(1,3-dibenzyl-4,7-dichloro-2-oxoindolin-3-yl)acetate (**4m**)

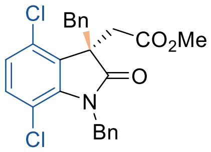

Pale yellow oil, 77% yield, 98% ee, **^{1}H NMR** (400 MHz, $CDCl_3$) δ 7.21 – 7.15 (m, 4H), 7.09 (t, J = 7.6 Hz, 2H), 7.04 (d, J = 8.7 Hz, 1H), 6.95 (d, J = 8.8 Hz, 1H), 6.89 – 6.83 (m, 2H), 6.81 – 6.73 (m, 2H), 5.16 – 5.02 (m, 2H), 3.69 – 3.58 (m, 2H), 3.54 (s, 3H), 3.30 (d, J = 16.9 Hz, 1H), 3.12 (d, J = 12.9 Hz, 1H). **^{13}C NMR** (101 MHz, $CDCl_3$) δ 178.4, 170.0, 141.8, 137.4, 134.3, 132.0, 129.6, 129.1, 128.9, 128.4, 128.1, 127.2, 126.6, 125.9, 124.0, 113.8, 53.0, 51.9, 45.1, 40.5, 39.5. **HRMS** (ESI-TOF) [M+Na] calculated for $[C_{25}H_{21}Cl_2NNaO_3]^+$ 476.0791, found 476.0791. **Specific Rotation** $[\alpha]_D^{25}$ = +12.8 (c = 0.20 in CH_2Cl_2). **HPLC** (Chiralpak-IC-H column, isopropanol/hexane = 30/70, 1.0 mL/min): t(minor) = 6.910 min, t(major) = 11.403 min.

methyl (S)-2-(1,3-dibenzyl-5,7-dimethyl-2-oxoindolin-3-yl)acetate (**4n**)

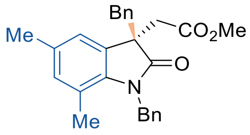

Pale yellow oil, 85% yield, 93% ee, **^{1}H NMR** (400 MHz, $CDCl_3$) δ 7.26 – 7.09 (m, 6H), 6.91 (d, J = 1.8 Hz, 1H), 6.89 – 6.80 (m, 2H), 6.71 (q, J = 2.7, 1.9 Hz, 3H), 5.04 (d, J = 17.1 Hz, 1H), 4.87 (d, J = 17.0 Hz, 1H), 3.53 (s, 3H), 3.24 (d, J = 16.3 Hz, 1H), 3.13 (s, 2H), 3.02 (d, J = 16.3 Hz, 1H), 2.32 (s, 3H), 1.99 (s, 3H). **^{13}C NMR** (101 MHz, $CDCl_3$) δ 179.2, 170.2, 139.3, 138.0, 135.1, 132.7, 131.4, 131.0, 130.3, 128.6, 127.8, 126.8, 126.6, 125.5, 121.7, 119.2, 51.7, 50.7, 45.0, 44.4, 41.4, 20.9, 18.3. **HRMS** (ESI-TOF) [M+H] calculated for $[C_{27}H_{28}O_3]^+$ 414.2064, found 414.2065. **Specific Rotation** $[\alpha]_D^{25}$ = +80.9 (c = 0.70 in CH_2Cl_2). **HPLC** (Chiralpak-OD-H column, isopropanol/hexane = 20/80, 1.0 mL/min): t(minor) = 5.900 min, t(major) = 7.430 min.

methyl (S)-2-(1,3-dibenzyl-5-chloro-7-methyl-2-oxoindolin-3-yl)acetate (**4o**)

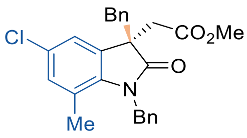

Colorless oil, 69% yield, 95% ee, **^{1}H NMR** (400 MHz, $CDCl_3$) δ 7.27 – 7.22 (m, 1H), 7.22 – 7.12 (m, 5H), 7.09 (d, J = 2.2 Hz, 1H), 6.94 – 6.83 (m, 3H), 6.71 (ddd, J = 6.1, 2.5, 1.2 Hz, 2H), 5.01 (d, J = 17.1 Hz, 1H), 4.88 (d, J = 17.1 Hz, 1H), 3.56 (s, 3H), 3.27 (d, J = 16.7 Hz, 1H), 3.19 – 3.07 (m, 2H), 3.02 (d, J = 16.6 Hz, 1H), 2.00 (s, 3H). **^{13}C NMR** (101 MHz, $CDCl_3$) δ 178.9, 170.0, 137.6, 134.5, 132.9, 131.8, 130.2, 128.7, 128.0, 127.2, 127.1, 126.8, 125.4, 121.2, 121.1, 51.9, 50.9, 45.1, 44.4, 41.2, 18.3. **HRMS** (ESI-TOF) [M+H] calculated for $[C_{26}H_{25}ClNO_3]^+$ 434.9395, found 434.9396. **Specific Rotation** $[\alpha]_D^{25}$ = +235.8 (c = 0.20 in CH_2Cl_2). **HPLC** (Chiralpak-IC-H column, isopropanol/hexane = 30/70, 1.0 mL/min): t(minor) = 8.633 min, t(major) = 23.020 min.

methyl (S)-2-(3-benzyl-1-methyl-2-oxoindolin-3-yl)acetate (**4p**)

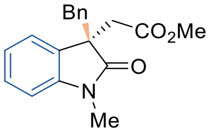

Colorless oil, 72% yield, 91% ee, **^{1}H NMR** (400 MHz, $CDCl_3$) δ 7.25 – 7.18 (m, 1H), 7.16 – 7.00 (m, 5H), 6.83 (dt, J = 6.6, 1.7 Hz, 2H), 6.63 (d, J = 7.8 Hz, 1H), 3.47 (s, 3H), 3.19 (d, J = 16.4 Hz, 1H), 3.06 (s, 2H), 3.02 (s, 3H), 2.99 (d, J = 16.4 Hz, 1H). **^{13}C NMR** (101 MHz, $CDCl_3$) δ 178.5, 170.3, 144.1, 134.8, 130.1, 130.1, 128.3, 127.5, 126.8, 123.2, 121.9, 107.8, 51.7, 51.2, 43.9, 40.2, 26.0. **HRMS** (ESI-TOF) [M+H] calculated for $[C_{19}H_{20}NO_3]^+$ 310.1438, found 310.1439. **Specific Rotation** $[\alpha]_D^{25}$ = +16.7 (c = 0.48 in CH_2Cl_2). **HPLC** (Chiralpak-IC-H column, isopropanol/hexane = 30/70, 1.0 mL/min): t(minor) = 14.947 min, t(major) = 11.877 min.

ethyl (S)-2-(1,3-dibenzyl-4,7-dichloro-2-oxoindolin-3-yl)acetate (**4q**)

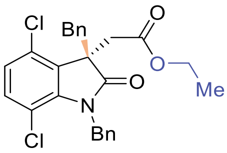

Colorless oil, 62% yield, 98% ee, **^{1}H NMR** (400 MHz, $CDCl_3$) δ 7.22 – 7.15 (m, 4H), 7.11 – 7.03 (m, 3H), 6.95 (d, J = 8.8 Hz, 1H), 6.90 – 6.83 (m, 2H), 6.73 (d, J = 3.4 Hz, 2H), 5.10 (s, 2H), 3.98 (dd, J = 21.1, 7.1 Hz, 2H), 3.69 – 3.58 (m, 2H),

3.29 (d, J = 16.6 Hz, 1H), 3.13 (d, J = 12.9 Hz, 1H), 1.08 (t, J = 7.1 Hz, 3H). **^{13}C NMR** (101 MHz, $CDCl_3$) δ 178.4, 169.4, 141.8, 137.4, 134.4, 132.0, 129.6, 129.2, 129.0, 128.4, 128.3, 128.1, 127.2, 126.6, 125.9, 123.9, 113.8, 60.7, 53.1, 45.1, 40.5, 40.0, 14.0. **HRMS** (ESI-TOF) [M+Na] calculated for $[C_{26}H_{23}Cl_2NNaO_3]^+$ 490.0947, found 490.0947. **Specific Rotation** $[\alpha]_D^{25}$ = +50.7 (c = 0.52 in CH_2Cl_2). **HPLC** (Chiralpak-OD-H column, isopropanol/hexane = 20/80, 1.0 mL/min): t(minor) = 5.730 min, t(major) = 4.960 min.

benzyl (S)-2-(1,3-dibenzyl-4,7-dichloro-2-oxoindolin-3-yl)acetate (**4r**)

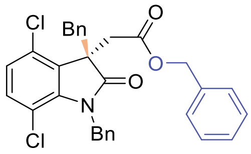

Colorless oil, 60% yield, 96% ee, **^{1}H NMR** (400 MHz, $CDCl_3$) δ 7.37 – 7.32 (m, 3H), 7.17 (tdd, J = 6.2, 5.9, 5.0, 2.0 Hz, 6H), 7.07 (t, J = 7.6 Hz, 2H), 7.00 (d, J = 8.7 Hz, 1H), 6.92 (d, J = 8.8 Hz, 1H), 6.87 – 6.80 (m, 2H), 6.68 – 6.57 (m, 2H), 5.03 – 4.87 (m, 3H), 4.76 (d, J = 16.7 Hz, 1H), 3.70 – 3.58 (m, 2H), 3.35 (d, J = 16.5 Hz, 1H), 3.12 (d, J = 12.9 Hz, 1H). **^{13}C NMR** (101 MHz, $CDCl_3$) δ 178.2, 169.2, 141.7, 137.4, 135.2, 134.3, 132.0, 129.6, 129.0, 128.9, 128.6, 128.42, 128.35, 128.3, 128.2, 127.2, 126.5, 125.8, 123.9, 66.7, 53.0, 44.9, 40.5, 40.0. **HRMS** (ESI-TOF) [M+Na] calculated for $[C_{31}H_{25}Cl_2NNaO_3]^+$ 552.104, found 552.1105. **Specific Rotation** $[\alpha]_D^{25}$ = +50.1 (c = 0.44 in CH_2Cl_2). **HPLC** (Chiralpak-IC-H column, isopropanol/hexane = 30/70, 1.0 mL/min): t(minor) = 7.850 min, t(major) = 9.303 min.

(S)-1,3-dibenzyl-4,7-dichloro-3-(2-oxo-2-(1H-pyrazol-1-yl)ethyl)indolin-2-one (**4s**)

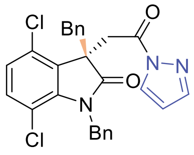

Colorless oil, 65% yield, 96% ee, **^{1}H NMR** (400 MHz, $CDCl_3$) δ 8.11 (d, J = 2.8 Hz, 1H), 7.76 (d, J = 1.4 Hz, 1H), 7.21 (dt, J = 4.0, 1.9 Hz, 4H), 7.11 (dd, J = 8.3, 6.9 Hz, 2H), 7.05 (d, J = 8.7 Hz, 1H), 6.93 (d, J = 8.8 Hz, 1H), 6.92 – 6.70 (m, 4H), 6.45 (dd, J = 2.9, 1.5 Hz, 1H), 5.18 (d, J = 16.6 Hz, 1H), 5.06 (d, J = 16.6 Hz, 1H), 4.82 (d, J = 18.7 Hz, 1H), 4.01 – 3.94 (m, 1H), 3.74 (d, J = 12.9 Hz, 1H), 3.23 (d, J = 12.9 Hz, 1H). **^{13}C NMR** (101 MHz, $CDCl_3$) δ 178.4, 168.3, 144.4, 141.9, 137.5, 134.1, 132.0, 129.6, 129.2, 128.7, 128.4, 128.1, 127.2, 126.6, 125.9, 123.9, 121.5, 113.9, 109.9, 52.5, 45.2, 40.8, 39.8. **HRMS** (ESI-TOF) [M+Na] calculated for $[C_{27}H_{21}Cl_2N_3NaO_2]^+$ 512.0903, found 512.0906. **Specific Rotation** $[\alpha]_D^{25}$ = +30.8 (c = 0.23 in CH_2Cl_2).

HPLC (Chiralpak-IC-H column, isopropanol/hexane = 30/70, 1.0 mL/min): t(minor) = 9.463 min, t(major) = 6.493 min.

2-(1-(4-chlorobenzoyl)-5-methoxy-2-methyl-1H-indol-3-yl)ethyl (S)-2-(1,3-dibenzyl-4,7-dichloro-2-oxoindolin-3-yl)acetate (**4t**)

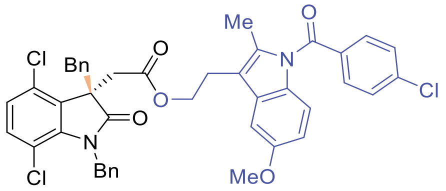

White solid, 66% yield, 96% ee, **^{1}H NMR** (400 MHz, $CDCl_3$) δ 7.76 – 7.55 (m, 2H), 7.55 – 7.41 (m, 2H), 7.23 – 7.14 (m, 4H), 7.09 (t, J = 7.7 Hz, 2H), 7.02 (d, J = 8.7 Hz, 1H), 6.96 – 6.83 (m, 5H), 6.80 – 6.72 (m, 2H), 6.69 (dd, J = 9.0, 2.6 Hz, 1H), 5.05 (d, J = 10.2 Hz, 2H), 4.12 (ddd, J = 17.1, 7.7, 6.4 Hz, 2H), 3.84 (s, 3H), 3.71 – 3.56 (m, 2H), 3.29 (d, J = 16.6 Hz, 1H), 3.13 (d, J = 12.9 Hz, 1H), 2.81 (dt, J = 8.0, 6.5 Hz, 2H), 2.32 (s, 3H). **^{13}C NMR** (101 MHz, $CDCl_3$) δ 178.3, 169.4, 168.3, 156.0, 141.8, 139.2, 137.3, 135.4, 134.3, 134.0, 132.0, 131.2, 130.9, 130.8, 129.6, 129.1, 129.1, 128.9, 128.4, 128.1, 127.2, 126.7, 125.9, 123.9, 115.1, 114.9, 113.8, 111.5, 101.0, 63.6, 55.8, 53.1, 45.1, 40.5, 39.8, 23.5, 13.3. **HRMS** (ESI-TOF) [M+Na] calculated for $[C_{43}H_{35}Cl_3N_2NaO_5]^+$ 487.1504, found 787.1511. **Specific Rotation** $[\alpha]_D^{25}$ = +21.4 (c = 0.40 in CH_2Cl_2). **HPLC** (Chiralpak-IC-H column, isopropanol/hexane = 30/70, 1.0 mL/min): t(minor) = 24.427 min, t(major) = 19.063 min.

S-benzyl (S)-2-(1,3-dibenzyl-4,7-dichloro-2-oxoindolin-3-yl)ethanethioate (**4u**)

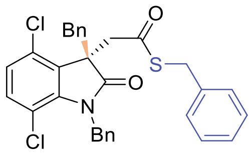

Colorless oil, 60% yield, 95% ee, **^{1}H NMR** (400 MHz, $CDCl_3$) δ 7.23 (ddd, J = 5.4, 4.3, 2.3 Hz, 3H), 7.22 – 7.16 (m, 4H), 7.16 – 7.04 (m, 4H), 7.02 (d, J = 8.8 Hz, 1H), 6.92 (d, J = 8.8 Hz, 1H), 6.90 – 6.81 (m, 2H), 6.75 (ddd, J = 5.3, 2.4, 1.3 Hz, 2H), 5.08 (s, 2H), 3.99 (s, 2H), 3.90 (d, J = 15.8 Hz, 1H), 3.62 (d, J = 12.9 Hz, 1H), 3.48 (d, J = 15.8 Hz, 1H), 3.12 (d, J = 12.9 Hz, 1H). **^{13}C NMR** (101 MHz, $CDCl_3$) δ 194.4, 177.9, 141.6, 137.4, 137.1, 134.2, 132.1, 129.7, 129.2, 128.7, 128.6, 128.4, 128.4, 128.1, 127.3, 127.2, 126.6, 125.9, 123.9, 113.9, 53.6, 48.3, 45.1, 40.1, 33.2. **HRMS** (ESI-TOF) [M+H] calculated for $[C_{31}H_{26}Cl_2NO_2S]^+$ 546.1056, found 546.1058. **Specific Rotation** $[\alpha]_D^{25}$ = -36.8 (c = 0.31 in CH_2Cl_2). **HPLC** (Chiralpak-IC-H column, isopropanol/hexane = 20/80, 1.0 mL/min): t(minor) = 6.980 min, t(major) = 9.080 min.

methyl (S)-2-(1-benzyl-4,7-dichloro-3-(4-fluorobenzyl)-2-oxoindolin-3-yl)acetate (**5a**)

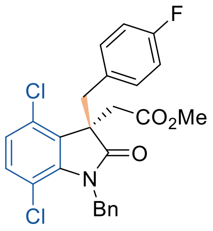

Pale yellow solid, 87% yield, 95% ee, **^{1}H NMR** (400 MHz, $CDCl_3$) δ 7.25 – 7.16 (m, 3H), 7.07 (d, J = 8.7 Hz, 1H), 6.96 (d, J = 8.8 Hz, 1H), 6.90 – 6.62 (m, 6H), 5.24 – 5.02 (m, 2H), 3.68 – 3.56 (m, 2H), 3.54 (s, 3H), 3.27 (d, J = 16.9 Hz, 1H), 3.09 (d, J = 13.1 Hz, 1H). **^{13}C NMR** (101 MHz, $CDCl_3$) δ 178.2, 169.9, 162.05 (d, J = 245.4 Hz), 141.8, 137.2, 132.2, 131.15 (d, J = 8.1 Hz), 130.10 (d, J = 3.3 Hz), 128.8, 128.3, 126.8, 126.0, 124.0, 115.1, 114.9, 114.0, 52.88 (d, J = 1.6 Hz), 51.9, 45.1, 39.5, 39.5. **^{19}F NMR** (376 MHz, $CDCl_3$) δ -115.16. **HRMS** (ESI-TOF) [M+H] calculated for $[C_{25}H_{21}Cl_2FNO_3]^+$ 472.0877, found 472.0880. **Specific Rotation** $[\alpha]_D^{25}$ = +59.5 (c = 0.60 in CH_2Cl_2). **HPLC** (Chiralpak-IC-H column, isopropanol/hexane = 30/70, 1.0 mL/min): t(minor) = 6.423 min, t(major) = 7.937 min.

methyl (S)-2-(1-benzyl-3-(4-chlorobenzyl)-4,6-difluoro-2-oxoindolin-3-yl)acetate (**5b**)

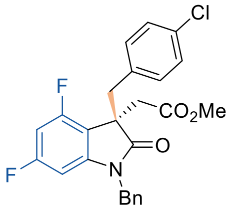

While solid, 73% yield, >99% ee, **^{1}H NMR** (400 MHz, $CDCl_3$) δ 7.28 – 7.19 (m, 3H), 7.11 – 7.02 (m, 2H), 6.87 – 6.80 (m, 2H), 6.76 (dd, J = 6.6, 2.9 Hz, 2H), 6.48 (td, J = 9.6, 2.1 Hz, 1H), 5.99 (dd, J = 8.5, 2.1 Hz, 1H), 4.92 (d, J = 16.1 Hz, 1H), 4.51 (d, J = 16.0 Hz, 1H), 3.52 (s, 3H), 3.26 (s, 2H), 3.25 – 3.10 (m, 2H). **^{13}C NMR** (101 MHz, $CDCl_3$) δ 177.8, 169.9, 163.42 (dd, J = 247.8, 13.1 Hz), 158.50 (dd, J = 246.6, 14.3 Hz), 146.31 (dd, J = 14.0, 12.3 Hz), 134.5, 133.1, 133.0, 131.0, 128.7, 128.3, 127.6, 126.7, 110.56 (dd, J = 19.4, 3.5 Hz), 97.52 (dd, J = 26.4, 24.9 Hz), 94.70 (dd, J = 27.6, 3.5 Hz), 51.9, 51.15 (d, J = 2.5 Hz), 44.4, 41.3, 40.5. **^{19}F NMR** (376 MHz, $CDCl_3$) δ -107.20 (d, J = 7.4 Hz), -118.20 (d, J = 7.6 Hz). **HRMS** (ESI-TOF) [M+H] calculated for $[C_{25}H_{21}ClF_2NO_3]^+$ 456.1173, found 456.1174. **Specific Rotation** $[\alpha]_D^{25}$ = +61.6 (c = 0.62 in CH_2Cl_2). **HPLC** (Chiralpak-IC-H column, isopropanol/hexane = 30/70, 1.0 mL/min): t(minor) = 8.597 min, t(major) = 5.177 min.

methyl (S)-2-(1-benzyl-3-(4-bromobenzyl)-4,7-dichloro-2-oxoindolin-3-yl)acetate (**5c**)

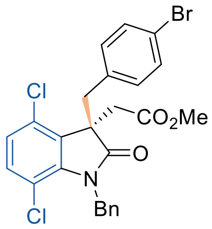

Yellow solid, 83% yield, 95% ee, **^{1}H NMR** (400 MHz, $CDCl_3$) δ 7.25 (d, J = 7.5 Hz, 3H), 7.18 (d, J = 8.4 Hz, 2H), 7.09 (d, J = 8.7 Hz, 1H), 6.97 (d, J = 8.8 Hz, 1H), 6.72 (dd, J = 10.0, 7.2 Hz, 4H), 5.15 (d, J = 2.3 Hz, 2H), 3.67 – 3.55 (m, 2H), 3.54 (s, 3H), 3.26 (d, J = 16.8 Hz, 1H), 3.06 (d, J = 13.0 Hz, 1H). **^{13}C NMR** (101 MHz, $CDCl_3$) δ 178.1, 169.8, 137.2, 133.4, 132.4, 131.3, 131.3, 128.9, 128.6, 128.4, 126.8, 126.0, 124.0, 121.3, 114.0, 52.7, 51.9, 45.2, 39.6. **HRMS** (ESI-TOF) [M+Na] calculated for $[C_{25}H_{20}BrCl_2NaNO_3]^+$ 553.9896, found 553.9899. **Specific Rotation** $[\alpha]_D^{25}$ = +48.0 (c = 0.78 in CH_2Cl_2). **HPLC** (Chiralpak-IC-H column, isopropanol/hexane = 30/70.5, 1.0 mL/min): t(minor) = 6.960 min, t(major) = 7.657 min.

methyl (S)-2-(1-benzyl-4,7-dichloro-3-(4-iodobenzyl)-2-oxoindolin-3-yl)acetate (**5d**)

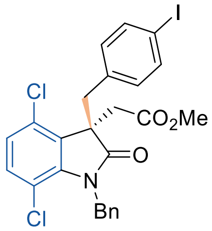

Yellow solid, 78% yield, 97% ee, **^{1}H NMR** (400 MHz, $CDCl_3$) δ 7.41 – 7.33 (m, 2H), 7.31 – 7.26 (m, 2H), 7.25 – 7.20 (m, 1H), 7.09 (d, J = 8.8 Hz, 1H), 6.96 (d, J = 8.8 Hz, 1H), 6.83 – 6.70 (m, 2H), 6.66 – 6.52 (m, 2H), 5.15 (d, J = 2.1 Hz, 2H), 3.68 – 3.54 (m, 2H), 3.54 (s, 3H), 3.25 (d, J = 16.9 Hz, 1H), 3.05 (d, J = 13.0 Hz, 1H). **^{13}C NMR** (101 MHz, $CDCl_3$) δ 178.1, 169.8, 141.8, 137.26, 137.2, 134.0, 132.4, 131.6, 128.9, 128.6, 128.5, 126.8, 126.0, 124.0, 114.0, 93.0, 52.6, 51.9, 45.2, 39.7, 39.6. **HRMS** (ESI-TOF) [M+Na] calculated for $[C_{25}H_{20}BrCl_2NaNO_3]^+$ 601.9757, found 601.9760. **Specific Rotation** $[\alpha]_D^{25}$ = +39.8 (c = 0.76 in CH_2Cl_2). **HPLC** (Chiralpak-IC-H column, isopropanol/hexane = 30/70, 1.0 mL/min): t(minor) = 7.220 min, t(major) = 7.847 min.

methyl (S)-2-(1-benzyl-3-(4-cyanobenzyl)-4,6-difluoro-2-oxoindolin-3-yl)acetate (**5e**)

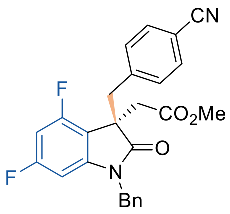

While solid, 67% yield, 99% ee, **^{1}H NMR** (400 MHz, $CDCl_3$) δ 7.35 – 7.31 (m, 2H), 7.30 – 7.25 (m, 3H), 7.04 – 6.90 (m, 2H), 6.88 – 6.76 (m, 2H), 6.49 (td, J = 9.6, 2.1 Hz, 1H), 6.11 – 5.99 (m, 1H), 4.82 (d, J = 15.9 Hz, 1H), 4.54 (d, J = 15.8 Hz, 1H), 3.53 (s, 3H), 3.27 (d, J = 3.9 Hz, 4H). **^{13}C NMR** (101 MHz, $CDCl_3$) δ 177.4, 169.7, 163.54 (dd, J = 248.6, 13.1 Hz), 160.50 – 154.24 (m), 146.93 – 145.05 (m), 140.0, 134.5, 131.8, 130.4, 128.8, 127.9, 127.0, 118.6, 111.1, 110.14 (dd, J = 19.5, 3.5 Hz), 97.68 (dd, J = 26.4, 24.8 Hz), 94.70 (dd, J = 27.6, 3.5 Hz), 52.0, 50.89 (d, J = 2.5 Hz), 44.5, 41.9, 40.45. **HRMS** (ESI-TOF) [M+H] calculated for $[C_{26}H_{21}F_2N_2O_3]^+$ 447.1515, found 447.1517. **Specific Rotation** $[\alpha]_D^{25}$ = +58.1 (c = 0.60 in CH_2Cl_2). **HPLC** (Chiralpak-IC-H column, isopropanol/hexane = 30/70, 1.0 mL/min): t(minor) = 10.590 min, t(major) = 13.973 min.

methyl (S)-2-(1-benzyl-4,7-dichloro-3-(4-nitrobenzyl)-2-oxoindolin-3-yl)acetate (**5f**)

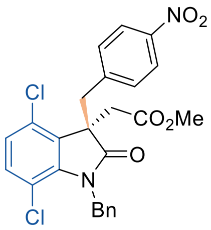

Yellow solid, 77% yield, 98% ee, **^{1}H NMR** (400 MHz, $CDCl_3$) δ 8.05 – 8.01 (m, 1H), 7.69 (t, J = 2.0 Hz, 1H), 7.25 – 7.14 (m, 5H), 7.09 (d, J = 8.7 Hz, 1H), 7.00 (d, J = 8.8 Hz, 1H), 6.91 – 6.80 (m, 2H), 5.20 (d, J = 16.3 Hz, 1H), 5.00 (d, J = 16.3 Hz, 1H), 3.67 (dd, J = 14.9, 2.0 Hz, 2H), 3.55 (s, 3H), 3.30 (d, J = 16.9 Hz, 1H), 3.23 (d, J = 13.0 Hz, 1H). **^{13}C NMR** (101 MHz, $CDCl_3$) δ 177.9, 169.7, 147.7, 141.5, 137.0, 136.3, 135.7, 132.6, 1290, 128.4, 127.0, 126.2, 124.4, 124.3, 122.3, 114.1, 52.5, 52.0, 45.2, 39.8, 39.4. **HRMS** (ESI-TOF) [M+H] calculated for $[C_{25}H_{21}Cl_2N_2O_5]^+$ 521.0641, found 521.0645. **Specific Rotation** $[\alpha]_D^{25}$ = +60.1 (c = 0.72 in CH_2Cl_2). **HPLC** (Chiralpak-IC-H column, isopropanol/hexane = 30/70, 1.0 mL/min): t(minor) = 14.820 min, t(major) = 12.433 min.

methyl (S)-4-((1-benzyl-4,7-dichloro-3-(2-methoxy-2-oxoethyl)-2-oxoindolin-3-yl)methyl)benzoate (**5g**)

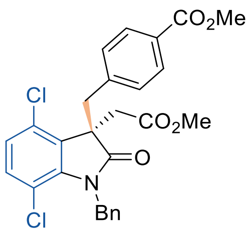

Yellow oil, 61% yield, 93% ee, **^{1}H NMR** (400 MHz, $CDCl_3$) δ 7.73 (d, J = 8.3 Hz, 2H), 7.23 – 7.12 (m, 3H), 7.06 (dd, J = 8.6, 1.5 Hz, 1H), 6.96 (d, J = 8.8 Hz, 1H), 6.94 – 6.86 (m, 2H), 6.89 – 6.72 (m, 2H), 5.22 – 4.97 (m, 2H), 3.92 (s, 3H), 3.70 – 3.60 (m, 2H), 3.54 (s, 3H), 3.29 (d, J = 16.9 Hz, 1H), 3.17 (d, J = 12.8 Hz, 1H). **^{13}C NMR** (101 MHz, $CDCl_3$) δ 178.1, 169.8, 166.9, 139.7, 137.2, 132.3, 129.6, 129.4, 129.0, 128.9, 128.6, 128.3, 126.9, 126.1, 124.0, 114.0, 52.7, 52.1, 51.9, 45.2, 40.3, 39.5. **HRMS** (ESI-TOF) [M+Na] calculated for $[C_{27}H_{23}Cl_2NNaO_5]^+$ 534.0845, found 534.0847. **Specific Rotation** $[\alpha]_D^{25}$ = +34.8 (c = 0.60 in CH_2Cl_2). **HPLC** (Chiralpak-OD-H column, isopropanol/hexane = 30/70, 1.0 mL/min): t(minor) = 6.193 min, t(major) = 7.927 min.

methyl (S)-2-(3-(4-acetylbenzyl)-1-benzyl-4,7-dichloro-2-oxoindolin-3-yl)acetate (**5h**)

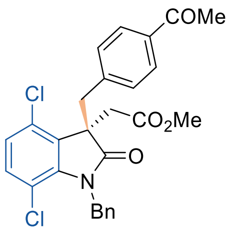

Clorless oil, 65% yield, 97% ee, **^{1}H NMR** (400 MHz, $CDCl_3$) δ 7.70 – 7.59 (m, 2H), 7.23 – 7.10 (m, 3H), 7.07 (d, J = 8.8 Hz, 1H), 7.01 – 6.91 (m, 3H), 6.90 – 6.70 (m, 2H), 5.26 – 5.03 (m, 2H), 3.68 (d, J = 4.9 Hz, 1H), 3.64 (s, 1H), 3.54 (s, 3H), 3.29 (d, J = 16.8 Hz, 1H), 3.18 (d, J = 12.8 Hz, 1H), 2.56 (s, 3H). **^{13}C NMR** (101 MHz, $CDCl_3$) δ 197.8, 178.1, 169.8, 141.7, 139.9, 137.2, 135.9, 132.4, 129.9, 128.9, 128.6, 128.3, 128.1, 126.9, 126.2, 124.1, 114.0, 52.6, 52.0, 45.2, 40.2, 39.6, 26.6. **HRMS** (ESI-TOF) [M+Na] calculated for $[C_{27}H_{23}Cl_2NNaO_4]^+$ 518.0896, found 5183.0898. **Specific Rotation** $[\alpha]_D^{25}$ = +51.1 (c = 0.42 in CH_2Cl_2). **HPLC** (Chiralpak-OD-H column, isopropanol/hexane = 30/70, 1.0 mL/min): t(minor) = 6.813 min, t(major) = 9.913 min.

Methyl (S)-2-(1-benzyl-4,7-dichloro-3-(4-(methylsulfonyl)benzyl)-2-oxoindolin-3-yl)acetate (**5i**)

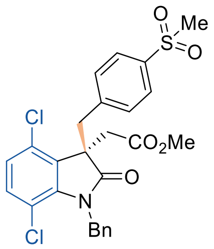

Pale yellow oil, 87% yield, 98% ee, **^{1}H NMR** (400 MHz, $CDCl_3$) δ 7.68 – 7.58 (m, 2H), 7.29 – 7.21 (m, 3H), 7.12 – 7.02 (m, 3H), 7.00 – 6.87 (m, 3H), 5.21 (d, J = 16.3 Hz, 1H), 5.02 (d, J = 16.3 Hz, 1H), 3.70 – 3.61 (m, 2H), 3.54 (s, 3H), 3.34 – 3.18 (m, 2H), 2.97 (s, 3H). **^{13}C NMR** (101 MHz, $CDCl_3$) δ 178.0, 169.7, 141.5, 140.8, 139.3, 136.9, 132.6, 130.6, 128.9, 128.5, 128.2, 127.2, 127.1, 126.3, 124.2, 114.2, 52.5, 52.0, 45.3, 44.5, 40.1, 39.5. **HRMS** (ESI-TOF) [M+Na] calculated for $[C_{26}H_{23}Cl_2NNaO_5S]^+$ 554.0566, found 554.0568. **Specific Rotation** $[\alpha]_D^{25}$ = -20.2 (c = 0.80 in CH_2Cl_2). **HPLC** (Chiralpak-AD-H column, isopropanol/hexane = 30/70, 1.0 mL/min): t(minor) = 22.190 min, t(major) = 25.870 min.

methyl (S)-2-(3-([1,1'-biphenyl]-4-ylmethyl)-1-benzyl-4,7-dichloro-2-oxoindolin-3-yl)acetate (**5j**)

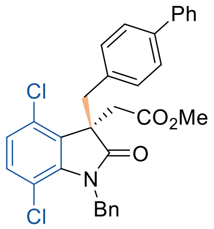

White solid, 70% yield, 96% ee, **^{1}H NMR** (400 MHz, $CDCl_3$) δ 7.62 – 7.55 (m, 2H), 7.45 (t, J = 7.5 Hz, 2H), 7.36 (dd, J = 11.2, 7.8 Hz, 3H), 7.14 – 7.04 (m, 4H), 6.98 (d, J = 8.8 Hz, 1H), 6.96 – 6.89 (m, 2H), 6.83 – 6.75 (m, 2H), 5.22 – 5.03 (m, 2H), 3.72 – 3.64 (m, 2H), 3.55 (s, 3H), 3.32 (d, J = 16.8 Hz, 1H), 3.17 (d, J = 12.9 Hz, 1H). **^{13}C NMR** (101 MHz, $CDCl_3$) δ 178.3, 170.0, 141.8, 140.5, 139.8, 137.4, 133.5, 132.1, 130.1, 129.1, 128.9, 128.8, 128.3, 127.3, 126.9, 126.6, 125.9, 124.0, 113.9, 53.0, 51.9, 45.1, 40.1, 39.6. **HRMS** (ESI-TOF) [M+Na] calculated for $[C_{31}H_{25}Cl_2NNaO_3]^+$ 552.1104, found 552.1106. **Specific Rotation** $[\alpha]_D^{25}$ = +43.4 (c = 0.70 in CH_2Cl_2). **HPLC** (Chiralpak-IC-H column, isopropanol/hexane = 30/70, 1.0 mL/min): t(minor) = 8.343 min, t(major) = 11.770 min.

methyl (S)-2-(1-benzyl-4,7-dichloro-3-(4-methylbenzyl)-2-oxoindolin-3-yl)acetate (**5k**)

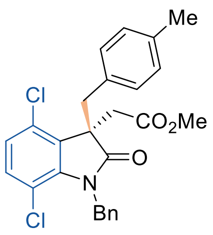

Colorless oil, 90% yield, 97% ee, **^{1}H NMR** (400 MHz, $CDCl_3$) δ 7.24 – 7.15 (m, 3H), 7.05 (d, J = 8.7 Hz, 1H), 6.95 (d, J = 8.8 Hz, 1H), 6.89 (d, J = 7.8 Hz, 2H), 6.76 (td, J = 6.6, 5.9, 1.8 Hz, 4H), 5.12 (s, 2H), 3.67 – 3.57 (m, 2H), 3.54 (s, 3H), 3.28 (d, J = 16.9 Hz, 1H), 3.09 (d, J = 13.0 Hz, 1H). **^{13}C NMR** (101 MHz, $CDCl_3$) δ 178.4, 170.0, 141.8, 137.5, 136.6, 132.0, 131.3, 129.5, 129.2, 128.9, 128.8, 128.2, 126.6, 126.0, 123.9, 113.8, 53.0, 51.9, 45.1, 40.0, 39.6, 21.2. **HRMS** (ESI-TOF) [M+Na] calculated for $[C_{26}H_{23}Cl_2NNaO_3]^+$ 490.0947, found 490.0948. **Specific Rotation** $[\alpha]_D^{25}$ = +52.2 (c = 0.80 in CH_2Cl_2). **HPLC** (Chiralpak-IC-H column, isopropanol/hexane = 30/70, 1.0 mL/min): t(minor) = 7.527 min, t(major) = 11.227 min.

methyl (S)-2-(1-benzyl-4,6-difluoro-3-(4-methoxybenzyl)-2-oxoindolin-3-yl)acetate (**5l**)

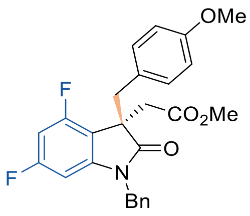

While solid, 77% yield, 97% ee, **^{1}H NMR** (400 MHz, $CDCl_3$) δ 7.26 – 7.14 (m, 3H), 6.86 – 6.79 (m, 2H), 6.79 – 6.70 (m, 2H), 6.67 – 6.60 (m, 2H), 6.47 (td, J = 9.6, 2.1 Hz, 1H), 5.95 (dd, J = 8.5, 2.1 Hz, 1H), 4.90 (d, J = 16.1 Hz, 1H), 4.53 (d, J = 16.1 Hz, 1H), 3.76 (s, 3H), 3.52 (s, 3H), 3.26 (s, 2H), 3.19 (q, J = 13.1 Hz, 2H). **^{13}C NMR** (101 MHz, $CDCl_3$) δ 178.1, 170.1, 163.29 (dd, J = 247.1, 13.0 Hz), 158.7, 158.48 (dd, J = 246.4, 14.3 Hz), 146.89 – 145.25 (m), 134.6, 130.7, 128.6, 127.5, 126.8, 126.6, 113.4, 111.04 (dd, J = 19.6, 3.6 Hz), 98.14 – 96.97 (m), 94.57 (dd, J = 27.6, 3.6 Hz), 55.1, 51.8, 51.45 (d, J = 2.8 Hz), 44.3, 40.89 (d, J = 90.5 Hz). **^{19}F NMR** (376 MHz, $CDCl_3$) δ -107.89 (d, J = 7.4 Hz), -118.37 (d, J = 7.5 Hz). **HRMS** (ESI-TOF) [M+H] calculated for $[C_{26}H_{24}F_2NO_4]^+$ 452.1668, found 452.1489. **Specific Rotation** $[\alpha]_D^{25}$ = +62.0 (c = 0.69 in CH_2Cl_2). **HPLC** (Chiralpak-IC-H column, isopropanol/hexane = 30/70, 1.0 mL/min): t(minor) = 6.847 min, t(major) = 8.553 min.

methyl (S)-2-(1-benzyl-4,7-dichloro-3-(4-(methylthio)benzyl)-2-oxoindolin-3-yl)acetate (**5m**)

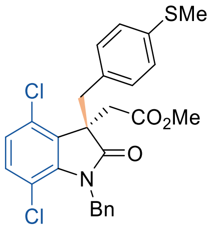

Yellow oil, 50% yield, 97% ee, **^{1}H NMR** (400 MHz, $CDCl_3$) δ 7.26 – 7.18 (m, 3H), 7.07 (d, J = 8.8 Hz, 1H), 7.00 – 6.92 (m, 3H), 6.81 – 6.75 (m, 2H), 6.75 – 6.66 (m, 2H), 5.20 – 5.09 (m, 2H), 3.67 – 3.58 (m, 2H), 3.54 (s, 3H), 3.26 (d, J = 16.9 Hz, 1H), 3.07 (d, J = 13.0 Hz, 1H), 2.44 (s, 3H). **^{13}C NMR** (101 MHz, $CDCl_3$) δ 178.22, 169.93, 141.85, 137.31, 137.29, 132.17, 131.03, 130.08, 128.94, 128.84, 128.28, 126.70, 125.97, 125.90, 123.97, 113.94, 52.88, 51.89, 45.10, 39.78, 39.58, 15.52. **HRMS** (ESI-TOF) [M+Na] calculated for $[C_{26}H_{23}Cl_2NNaO_3S]^+$ 522.0668, found 522.0671. **Specific Rotation** $[\alpha]_D^{25}$ = +50.7 (c = 0.30 in CH_2Cl_2). **HPLC** (Chiralpak-IC-H column, isopropanol/hexane = 30/70, 1.0 mL/min): t(minor) = 8.757 min, t(major) = 10.217 min.

methyl (S)-2-(1-benzyl-4,6-difluoro-2-oxo-3-(3-(trifluoromethyl)benzyl)indolin-3-yl)acetate (**5n**)

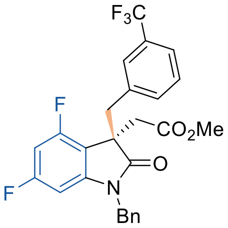

Colorless oil, 77% yield, 98% ee, **^{1}H NMR** (400 MHz, $CDCl_3$) δ 7.44 (ddt, J = 7.8, 1.9, 1.0 Hz, 1H), 7.25 – 7.17 (m, 4H), 7.15 – 7.04 (m, 2H), 6.91 – 6.81 (m, 2H), 6.49 (td, J = 9.6, 2.1 Hz, 1H), 6.00 – 5.91 (m, 1H), 4.72 – 4.57 (m, 2H), 3.53 (s, 3H), 3.35 – 3.26 (m, 4H). **^{13}C NMR** (101 MHz, $CDCl_3$) δ 177.7, 169.8, 163.51 (dd, J = 248.2, 13.0 Hz), 158.56 (dd, J = 246.5, 14.3 Hz), 149.03 – 144.32 (m), 135.5, 134.5, 133.05 (d, J = 1.5 Hz), 130.33 (q, J = 32.2 Hz), 128.7, 128.5, 127.6, 126.8, 126.22 (d, J = 3.8 Hz), 123.90 (q, J = 3.9 Hz), 123.82 (q, J = 272.4 Hz), 110.37 (dd, J = 19.5, 3.5 Hz), 97.53 (dd, J = 26.5, 24.9 Hz), 94.64 (dd, J = 27.6, 3.6 Hz), 51.9, 51.11 (d, J = 2.5 Hz), 44.4, 41.9, 40.3. **^{19}F NMR** (376 MHz, $CDCl_3$) δ -62.73, -107.18 (d, J = 7.5 Hz), -118.02 (d, J = 7.6 Hz). **HRMS** (ESI-TOF) [M+Na] calculated for $[C_{26}H_{20}F_5NNaO_3]^+$ 512.1256, found 512.1258. **Specific Rotation** $[\alpha]_D^{25}$ = +18.6 (c = 0.75 in CH_2Cl_2). **HPLC** (Chiralpak-IC-H column, isopropanol/hexane = 30/70, 1.0 mL/min): t(minor) = 5.443 min, t(major) = 6.457 min.

methyl (S)-2-(1-benzyl-4,7-dichloro-2-oxo-3-((perfluorophenyl)methyl)indolin-3-

yl)acetate (**5o**)

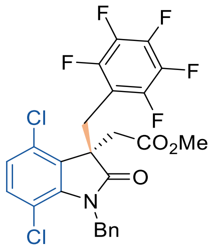

Yellow liquid, 65% yield, 98% ee, **^{1}H NMR** (400 MHz, $CDCl_3$) δ 7.34 – 7.28 (m, 3H), 7.24 – 7.14 (m, 3H), 6.96 (d, J = 8.8 Hz, 1H), 5.36 (d, J = 16.3 Hz, 1H), 5.27 (d, J = 16.3 Hz, 1H), 3.76 (d, J = 17.1 Hz, 1H), 3.54 (s, 3H), 3.50 – 3.40 (m, 1H), 3.29 – 3.14 (m, 2H). **^{13}C NMR** (101 MHz, $CDCl_3$) δ 177.2, 169.8, 147.1, 144.4, 141.7, 141.1, 138.6, 137.3, 132.5, 129.2, 128.4, 127.2, 126.5, 124.4, 114.1, 108.2, 52.0, 50.3, 45.3, 37.7, 28.1. **^{19}F NMR** (376 MHz, $CDCl_3$) δ -138.96 (d, J = 14.5 Hz), -154.02, -161.67 (d, J = 6.6 Hz). **HRMS** (ESI-TOF) [M+Na] calculated for $[C_{25}H_{16}Cl_2F_5NNaO_3]^+$ 566.0320, found 566.0322. **Specific Rotation** $[\alpha]_D^{25}$ = +17.2 (c = 0.70 in CH_2Cl_2). **HPLC** (Chiralpak-OD-H column, isopropanol/hexane = 20/80, 1.0 mL/min): t(minor) = 5.347 min, t(major) = 6.127 min.

methyl methyl (S)-2-(1-benzyl-3-((4-bromonaphthalen-1-yl)methyl)-4,6-difluoro-2-oxoindolin-3-yl)acetate (**5p**)

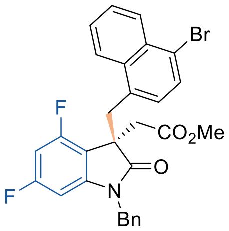

Colorless sticky oil, 77% yield, 98% ee, **^{1}H NMR** (400 MHz, $CDCl_3$) δ 8.28 – 8.16 (m, 1H), 8.12 (dd, J = 8.0, 1.5 Hz, 1H), 7.57 – 7.46 (m, 3H), 7.24 – 7.10 (m, 3H), 6.97 (d, J = 7.8 Hz, 1H), 6.57 – 6.46 (m, 2H), 6.37 (td, J = 9.7, 2.1 Hz, 1H), 5.84 (dd, J = 8.5, 2.1 Hz, 1H), 4.83 (d, J = 16.0 Hz, 1H), 4.35 (d, J = 16.0 Hz, 1H), 3.79 – 3.67 (m, 2H), 3.54 (s, 3H), 3.42 (d, J = 1.2 Hz, 2H). **^{13}C NMR** (101 MHz, $CDCl_3$) δ 178.2, 170.0, 163.32 (dd, J = 247.8, 13.1 Hz), 158.41 (dd, J = 245.8, 14.3 Hz), 149.12 – 145.11 (m), 134.4, 133.4, 131.8, 131.2, 129.1, 128.8, 128.7, 127.5, 127.5, 127.1, 126.6, 126.5, 124.40 (d, J = 2.0 Hz), 122.7, 111.09 (dd, J = 19.2, 3.5 Hz), 97.43 (t, J = 25.8 Hz), 94.62 (dd, J = 27.5, 3.5 Hz), 51.9, 51.05 (d, J = 2.5 Hz), 44.4, 37.8. **^{19}F NMR** (376 MHz, $CDCl_3$) δ -107.37 (d, J = 7.6 Hz), -116.77 (d, J = 7.7 Hz). **HRMS** (ESI-TOF) [M+H] calculated for $[C_{29}H_{23}BrF_2NO_3]^+$ 550.0824, found 550.0828. **Specific Rotation** $[\alpha]_D^{25}$ = +79.6 (c = 0.8 in CH_2Cl_2). **HPLC** (Chiralpak-IC-H column, isopropanol/hexane = 30/70, 1.0 mL/min): t(minor) = 5.870 min, t(major) = 7.247 min.

methyl (S)-2-((1-benzyl-4,7-dichloro-3-(2-methoxy-2-oxoethyl)-2-oxoindolin-3-yl)methyl)furan-3-carboxylate (**5q**)

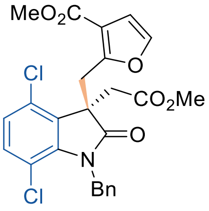

Colorless oil, 66% yield, 97% ee, **^{1}H NMR** (400 MHz, $CDCl_3$) δ 7.34 – 7.26 (m, 5H), 7.11 – 7.04 (m, 2H), 6.86 (d, J = 8.8 Hz, 1H), 6.56 (d, J = 2.0 Hz, 1H), 5.31 (q, J = 16.3 Hz, 2H), 3.91 (d, J = 14.5 Hz, 1H), 3.75 (s, 3H), 3.71 (d, J = 17.1 Hz, 1H), 3.61 (d, J = 14.6 Hz, 1H), 3.53 (s, 3H), 3.31 (d, J = 17.1 Hz, 1H). **^{13}C NMR** (101 MHz, $CDCl_3$) δ 178.2, 169.9, 163.6, 155.0, 141.6, 141.3, 137.6, 132.1, 129.3, 129.1, 128.4, 127.0, 126.7, 124.0, 116.2, 113.8, 110.8, 51.9, 51.5, 50.6, 45.4, 38.5, 33.0. **HRMS** (ESI-TOF) [M+H] calculated for $[C_{25}H_{22}Cl_2NO_6]^+$ 502.0819, found 502.0822. **Specific Rotation** $[\alpha]_D^{25}$ = -22.8 (c = 0.66 in CH_2Cl_2). **HPLC** (Chiralpak-IC-H column, isopropanol/hexane = 20/80, 1.0 mL/min): t(minor) = 12.067 min, t(major) = 24.017 min.

methyl (S)-2-(1-benzyl-4,7-dichloro-2-oxo-3-(thiophen-3-ylmethyl)indolin-3-yl)acetate (**5r**)

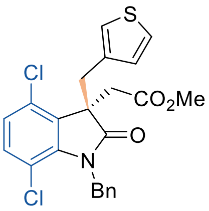

Colorless oil, 58% yield, 93% ee, **^{1}H NMR** (400 MHz, $CDCl_3$) δ 7.28 – 7.20 (m, 3H), 7.08 (d, J = 8.8 Hz, 1H), 7.06 – 7.01 (m, 1H), 6.95 (d, J = 8.8 Hz, 1H), 6.92 – 6.85 (m, 2H), 6.75 (dd, J = 3.0, 1.3 Hz, 1H), 6.49 (dd, J = 4.9, 1.3 Hz, 1H), 5.24 – 5.08 (m, 2H), 3.69 – 3.59 (m, 2H), 3.54 (s, 3H), 3.31 – 3.16 (m, 2H). **^{13}C NMR** (101 MHz, $CDCl_3$) δ 178.5, 170.0, 141.9, 137.4, 134.5, 132.1, 129.3, 128.7, 128.5, 128.4, 126.7, 126.0, 125.1, 123.9, 123.6, 113.9, 52.6, 51.9, 45.19, 39.3, 35.1. **HRMS** (ESI-TOF) [M+Na] calculated for $[C_{23}H_{19}Cl_2NNaO_3S]^+$ 482.0355, found 482.0357. **Specific Rotation** $[\alpha]_D^{25}$ = +44.2 (c = 0.48 in CH_2Cl_2). **HPLC** (Chiralpak-IC-H column, isopropanol/hexane = 40/60, 1.0 mL/min): t(minor) = 17.800 min, t(major) = 14.107 min.

methyl (R)-2-(1-benzyl-4,7-dichloro-3-((5-cyanothiophen-2-yl)methyl)-2-oxoindolin-

3-yl)acetate (**5s**)

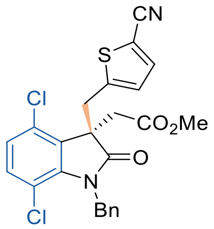

Colorless oil, 56% yield, 98% ee, **^{1}H NMR** (400 MHz, $CDCl_3$) δ 7.28 – 7.23 (m, 4H), 7.20 (d, J = 8.8 Hz, 1H), 7.02 (d, J = 8.8 Hz, 1H), 6.93 – 6.85 (m, 2H), 6.55 (d, J = 3.8 Hz, 1H), 5.27 (dd, J = 16.3, 0.9 Hz, 1H), 5.15 (d, J = 16.3 Hz, 1H), 3.81 (dd, J = 14.1, 0.8 Hz, 1H), 3.62 – 3.52 (m, 4H), 3.42 (d, J = 14.1 Hz, 1H), 3.22 (d, J = 16.7 Hz, 1H). **^{13}C NMR** (101 MHz, $CDCl_3$) δ 177.5, 169.4, 143.9, 142.1, 137.2, 136.9, 133.1, 129.0, 128.5, 128.0, 127.8, 127.2, 126.3, 124.4, 114.4, 114.1, 109.1, 52.2, 52.1, 45.3, 39.2, 34.7. **HRMS** (ESI-TOF) [M+Na] calculated for $[C_{24}H_{18}Cl_2N_2NaO_3S]^+$ 507.0307, found 507.0310. **Specific Rotation** $[\alpha]_D^{25}$ = -32.4 (c = 0.45 in CH_2Cl_2). **HPLC** (Chiralpak-IC-H column, isopropanol/hexane = 30/70, 1.0 mL/min): t(minor) = 11.527 min, t(major) = 14.170 min.

methyl (S)-2-(3-(benzo[b]thiophen-7-ylmethyl)-1-benzyl-4,7-dichloro-2-oxoindolin-3-yl)acetate (**5t**)

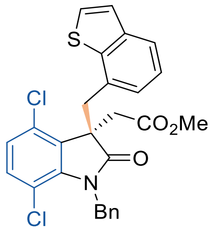

Colorless oil, 60% yield, 98% ee, **^{1}H NMR** (400 MHz, $CDCl_3$) δ 7.70 (dd, J = 7.9, 1.2 Hz, 1H), 7.33 (d, J = 5.5 Hz, 1H), 7.27 (s, 1H), 7.17 – 7.01 (m, 5H), 6.94 (d, J = 8.7 Hz, 1H), 6.79 (dd, J = 7.4, 1.1 Hz, 1H), 6.60 – 6.45 (m, 2H), 5.19 – 4.99 (m, 2H), 3.90 – 3.73 (m, 2H), 3.56 (s, 3H), 3.51 – 3.38 (m, 2H). **^{13}C NMR** (101 MHz, $CDCl_3$) δ 178.3, 170.0, 141.9, 141.3, 140.0, 137.3, 132.2, 129.6, 129.2, 128.9, 128.3, 126.5, 126.3, 125.6, 125.6, 124.1, 123.9, 122.8, 113.9, 52.2, 51.9, 45.1, 39.9, 39.6. **HRMS** (ESI-TOF) [M+Na] calculated for $[C_{27}H_{21}Cl_2NNaO_3S]^+$ 532.0511, found 532.0513. **Specific Rotation** $[\alpha]_D^{25}$ = +121.8 (c = 0.60 in CH_2Cl_2). **HPLC** (Chiralpak-IC-H column, isopropanol/hexane = 30/70, 1.0 mL/min): t(minor) = 10.933 min, t(major) = 13.337 min.

methyl (S)-2-(1-benzyl-4,7-dichloro-3-((2-cyanopyridin-3-yl)methyl)-2-oxoindolin-3-yl)acetate (**5u**)

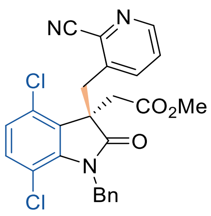

Colorless oil, 51% yield, 98% ee, **^{1}H NMR** (400 MHz, $CDCl_3$) δ 8.53 (dd, J = 4.7, 1.6 Hz, 1H), 7.37 (dd, J = 8.1, 1.6 Hz, 1H), 7.25 (dd, J = 5.2, 2.0 Hz, 3H), 7.20 – 7.12 (m, 2H), 7.09 – 6.95 (m, 3H), 5.29 (d, J = 16.2 Hz, 1H), 5.06 (d, J = 16.2 Hz, 1H), 3.80 (d, J = 17.0 Hz, 1H), 3.65 (d, J = 13.7 Hz, 1H), 3.55 (s, 3H), 3.40 (d, J = 13.7 Hz, 1H), 3.31 (d, J = 17.0 Hz, 1H). **^{13}C NMR** (101 MHz, $CDCl_3$) δ 177.4, 169.6, 149.8, 141.1, 138.9, 137.0, 134.6, 134.5, 132.8, 129.8, 128.5, 128.0, 127.1, 126.6, 125.9, 124.9, 115.7, 114.1, 52.1, 51.6, 45.2, 38.8, 37.1. **HRMS** (ESI-TOF) [M+H] calculated for $[C_{25}H_{20}Cl_2N_3O_3]^+$ 480.0876, found 480.0879. **Specific Rotation** $[\alpha]_D^{25}$ = -10.2 (c = 0.40 in CH_2Cl_2). **HPLC** (Chiralpak-IC-H column, isopropanol/hexane = 40/60, 1.0 mL/min): t(minor) = 21.470 min, t(major) = 28.670 min.

methyl (S)-2-(1-benzyl-4,7-dichloro-2-oxo-3-(pyrazin-2-ylmethyl)indolin-3-yl)acetate (**5v**)

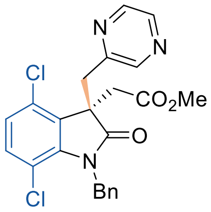

Pale yellow solid, 45% yield, 98% ee, **^{1}H NMR** (400 MHz, $CDCl_3$) δ 8.32 (d, J = 2.5 Hz, 1H), 8.28 – 8.19 (m, 2H), 7.37 – 7.29 (m, 2H), 7.28 – 7.24 (m, 1H), 7.20 (dd, J = 8.1, 1.5 Hz, 2H), 7.04 (d, J = 8.8 Hz, 1H), 6.88 (d, J = 8.8 Hz, 1H), 5.31 (d, J = 16.3 Hz, 1H), 5.15 (d, J = 16.2 Hz, 1H), 3.75 – 3.61 (m, 2H), 3.54 (s, 3H), 3.45 (d, J = 13.4 Hz, 1H), 3.33 (d, J = 16.8 Hz, 1H). **^{13}C NMR** (101 MHz, $CDCl_3$) δ 178.2, 169.7, 151.2, 144.8, 143.7, 143.2, 141.5, 137.5, 132.3, 129.0, 128.5, 128.4, 127.0, 126.8, 124.1, 113.8, 52.0, 51.2, 45.5, 40.2, 39.4. **HRMS** (ESI-TOF) [M+H] calculated for $[C_{23}H_{20}Cl_2N_3O_3]^+$ 456.0876, found 456.0877. **Specific Rotation** $[\alpha]_D^{25}$ = -10.2 (c = 0.40 in CH_2Cl_2). **HPLC** (Chiralpak-IB-H column, isopropanol/hexane = 40/60, 1.0 mL/min): t(minor) = 9.143 min, t(major) = 11.860 min.

methyl (S)-2-(1-benzyl-4,7-dichloro-2-oxo-3-(pyrimidin-5-ylmethyl)indolin-3-yl)acetate (**5w**)

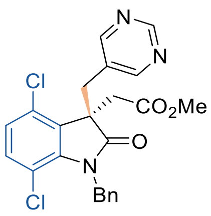

Pale yellow solid, 51% yield, 97% ee, **^{1}H NMR** (400 MHz, $CDCl_3$) δ 9.02 (s, 1H), 8.21 (s, 2H), 7.28 – 7.21 (m, 3H), 7.12 (d, J = 8.8 Hz, 1H), 7.04 – 6.91 (m, 3H), 5.24 (d, J = 16.3 Hz, 1H), 5.02 (d, J = 16.4 Hz, 1H), 3.67 (d, J = 16.8 Hz, 1H), 3.63 – 3.45 (m, 4H), 3.31 (d, J = 16.9 Hz, 1H), 3.12 (d, J = 13.3 Hz, 1H). **^{13}C NMR** (101 MHz, $CDCl_3$) 177.6, 169.5, 157.8, 157.3, 141.4, 136.9, 132.9, 128.9, 128.5, 128.1, 127.6, 127.1, 126.1, 124.5, 114.4, 52.2, 52.1, 45.3, 39.2, 35.2. **HRMS** (ESI-TOF) [M+Na] calculated for $[C_{23}H_{19}Cl_2N_3NaO_3]^+$ 478.0696, found 478.0698. **Specific Rotation** $[\alpha]_D^{25}$ = -91.5 (c = 0.26 in CH_2Cl_2). **HPLC** (Chiralpak-IC-H column, isopropanol/hexane = 40/60, 1.0 mL/min): t(minor) = 13.960 min, t(major) = 16.420 min.

tert-butyl (S)-3-((1-benzyl-4,7-dichloro-3-(2-methoxy-2-oxoethyl)-2-oxoindolin-3-yl)methyl)-1H-indole-1-carboxylate (**5x**)

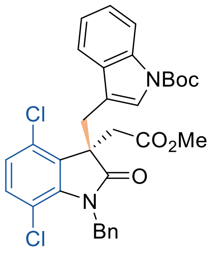

Colorless sticky oil, 62% yield, 96% ee, **^{1}H NMR** (400 MHz, $CDCl_3$) δ 8.13 (d, J = 8.3 Hz, 1H), 7.43 (dt, J = 7.9, 1.0 Hz, 1H), 7.28 – 7.23 (m, 1H), 7.17 – 7.02 (m, 5H), 6.97 (d, J = 8.8 Hz, 1H), 6.88 (s, 1H), 6.64 – 6.51 (m, 2H), 5.04 (q, J = 16.6 Hz, 2H), 3.81 – 3.64 (m, 2H), 3.56 (s, 3H), 3.33 (dd, J = 15.5, 12.9 Hz, 2H), 1.56 (s, 9H). **^{13}C NMR** (101 MHz, $CDCl_3$) δ 178.6, 170.0, 149.3, 142.2, 137.3, 132.2, 130.3, 129.8, 128.7, 128.2, 126.6, 125.5, 124.8, 124.4, 124.0, 122.6, 118.9, 114.9, 114.0, 113.5, 83.5, 52.2, 51.9, 45.1, 39.4, 30.1, 28.1. **HRMS** (ESI-TOF) [M+H] calculated for $[C_{32}H_{31}Cl_2N_2O_5]^+$ 593.1605, found 593.1608. **Specific Rotation** $[\alpha]_D^{25}$ = +81.1 (c = 0.72 in CH_2Cl_2). **HPLC** (Chiralpak-IC-H column, isopropanol/hexane = 30/70, 1.0 mL/min): t(minor) = 6.647 min, t(major) = 7.560 min.

methyl (S)-2-(1-benzyl-4,7-dichloro-2-oxo-3-(quinolin-5-ylmethyl)indolin-3-yl)acetate (**5y**)

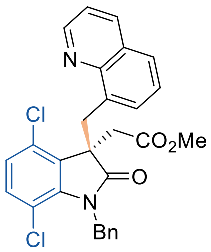

Colorless oil, 70% yield, 97% ee, **^{1}H NMR** (400 MHz, $CDCl_3$) δ 8.73 (dd, J = 4.1, 1.8 Hz, 1H), 8.05 (dd, J = 8.3, 1.8 Hz, 1H), 7.69 (dd, J = 8.1, 1.6 Hz, 1H), 7.39 (dd, J = 7.2, 1.6 Hz, 1H), 7.35 – 7.30 (m, 1H), 7.28 – 7.25 (m, 1H), 7.16 (dt, J = 4.8, 1.7 Hz, 3H), 6.86 (d, J = 8.7 Hz, 1H), 6.84 – 6.77 (m, 3H), 5.06 (d, J = 16.5 Hz, 1H), 4.93 (d, J = 16.5 Hz, 1H), 4.35 (d, J = 12.7 Hz, 1H), 3.93 – 3.81 (m, 2H), 3.54 (s, 3H), 3.45 (d, J = 17.2 Hz, 1H). **^{13}C NMR** (101 MHz, $CDCl_3$) δ 178.6, 170.5, 148.9, 147.0, 141.1, 137.7, 135.7, 133.6, 131.3, 131.0, 130.2, 130.0, 128.3, 127.9, 127.4, 126.6, 126.0, 125.8, 123.8, 120.8, 113.1, 52.7, 51.8, 44.9, 39.2, 35.4. **HRMS** (ESI-TOF) [M+H] calculated for $[C_{28}H_{23}Cl_2N_2O_3]^+$ 505.1080, found 505.1083. **Specific Rotation** $[\alpha]_D^{25}$ = +27.9 (c = 0.62 in CH_2Cl_2). **HPLC** (Chiralpak-IC-H column, isopropanol/hexane = 30/70, 1.0 mL/min): t(minor) = 14.913 min, t(major) = 29.223 min.

methyl (S)-2-(1-benzyl-4,7-dichloro-2-oxo-3-(quinoxalin-5-ylmethyl)indolin-3-yl)acetate (**5z**)

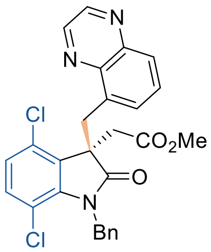

Colorless sticky oil, 69% yield, 98% ee, **^{1}H NMR** (400 MHz, $CDCl_3$) δ 8.81 (dd, J = 14.6, 1.8 Hz, 2H), 7.81 (d, J = 8.6 Hz, 1H), 7.63 (d, J = 2.0 Hz, 1H), 7.30 (dd, J = 8.7, 2.0 Hz, 1H), 7.13 – 6.98 (m, 3H), 6.92 (dd, J = 8.5, 7.0 Hz, 2H), 6.80 – 6.61 (m, 2H), 5.15 (d, J = 16.5 Hz, 1H), 5.00 (d, J = 16.5 Hz, 1H), 3.85 (d, J = 12.9 Hz, 1H), 3.73 (d, J = 16.9 Hz, 1H), 3.55 (s, 3H), 3.44 – 3.28 (m, 2H). **^{13}C NMR** (101 MHz, $CDCl_3$) δ 178.00, 169.8, 145.2, 144.9, 142.5, 142.0, 141.6, 137.1, 137.0, 132.4, 132.0, 130.1, 129.1, 129.0, 128.5, 128.1, 126.7, 125.9, 124.3, 114.0, 52.7, 52.0, 45.1, 40.3, 39.6. **HRMS** (ESI-TOF) [M+H] calculated for $[C_{27}H_{22}Cl_2N_3O_3]^+$ 506.1033, found 506.1036. **Specific Rotation** $[\alpha]_D^{25}$ = +55.3 (c = 0.80 in CH_2Cl_2). **HPLC** (Chiralpak-IC-H column, isopropanol/hexane = 40/60, 1.0 mL/min): t(minor) = 17.800 min, t(major) = 14.107 min.

methyl (S)-2-(1-benzyl-4,7-dichloro-3-((7-methoxy-2-oxo-2H-chromen-4-yl)methyl)-2-oxoindolin-3-yl)acetate (**5aa**)

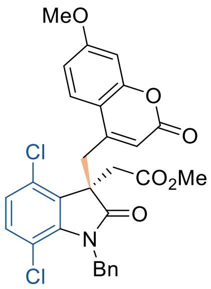

Colorless oil, 70% yield, 98% ee, **^{1}H NMR** (400 MHz, $CDCl_3$) δ 7.61 (d, J = 8.9 Hz, 1H), 7.26 – 7.05 (m, 4H), 6.97 (d, J = 8.8 Hz, 1H), 6.86 (dd, J = 6.6, 2.8 Hz, 2H), 6.81 – 6.64 (m, 2H), 5.76 (s, 1H), 5.20 – 4.93 (m, 2H), 3.84 (s, 3H), 3.72 (d, J = 16.8 Hz, 1H), 3.56 (s, 3H), 3.50 (d, J = 13.2 Hz, 1H), 3.45 – 3.28 (m, 2H). **^{13}C NMR** (101 MHz, $CDCl_3$) δ 177.7, 169.5, 162.8, 160.3, 155.3, 149.1, 141.8, 136.9, 132.9, 128.8, 128.4, 128.2, 126.9, 126.4, 125.9, 124.5, 114.5, 113.8, 112.2, 111.8, 101.2, 55.7, 52.1, 51.4, 45.4, 39.7, 34.9. **HRMS** (ESI-TOF) [M+Na] calculated for $[C_{29}H_{23}Cl_2NNaO_6]^+$ 574.0795, found 574.0797. **Specific Rotation** $[\alpha]_D^{25}$ = +64.8 (c = 0.77 in CH_2Cl_2). **HPLC** (Chiralpak-OD-H column, isopropanol/hexane = 30/70, 1.0 mL/min): t(minor) = 14.880 min, t(major) = 17.887 min.

4-(((S)-1-benzyl-4,7-dichloro-3-(2-methoxy-2-oxoethyl)-2-oxoindolin-3-yl)methyl)phenyl (S)-2-(6-methoxynaphthalen-2-yl)propanoate (**5ab**)

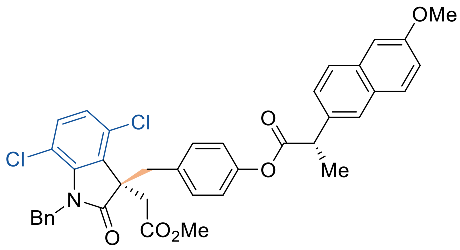

Colorless sticky oil, 69% yield, 94% de, **^{1}H NMR** (400 MHz, $CDCl_3$) δ 7.85 – 7.69 (m, 3H), 7.52 (dd, J = 8.4, 1.8 Hz, 1H), 7.25 – 7.09 (m, 4H), 7.04 (dd, J = 14.4, 8.0 Hz, 2H), 6.91 (d, J = 8.7 Hz, 1H), 6.88 – 6.74 (m, 4H), 6.73 – 6.66 (m, 2H), 5.10 (q, J = 16.6 Hz, 2H), 4.20 – 4.10 (m, 1H), 3.95 (s, 3H), 3.68 – 3.55 (m, 2H), 3.53 (s, 3H), 3.26 (d, J = 16.9 Hz, 1H), 3.08 (d, J = 13.1 Hz, 1H), 1.71 (d, J = 7.1 Hz, 3H). **^{13}C NMR** (101 MHz, $CDCl_3$) δ 178.2, 172.9, 169.9, 157.8, 150.0, 141.7, 137.3, 135.1, 133.8, 132.2, 131.9, 130.5, 129.3, 129.0, 128.8, 128.8, 128.3, 128.2, 128.0, 127.4, 126.7, 126.14, 126.0, 123.9, 121.1, 119.2, 113.9, 105.6, 55.4, 52.8, 51.9, 45.6, 45.1, 39.7, 39.5, 18.4. **HRMS** (ESI-TOF) [M+Na] calculated for

$[C_{39}H_{33}Cl_2NNaO_6]^+$ 704.1577, found 704.1580. **Specific Rotation** $[\alpha]_D^{25}$ = +81.1 (c = 0.72 in CH_2Cl_2). **HPLC** (Chiralpak-IC-H column, isopropanol/hexane = 30/70, 1.0 mL/min): t(minor) = 16.483 min, t(major) = 22.693 min.

methyl (S)-2-(1-benzyl-4,7-dichloro-3-((4-(4-fluorophenyl)-6-isopropyl-2-(N-methylmethylsulfonamido)pyrimidin-5-yl)methyl)-2-oxoindolin-3-yl)acetate (**5ac**)

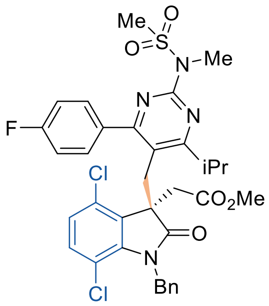

Pale yellow solid, 72% yield, 99% ee, **^{1}H NMR** (400 MHz, $CDCl_3$) δ 7.70 – 7.29 (m, 4H), 7.28 – 6.94 (m, 6H), 6.93 – 6.43 (m, 1H), 5.46 – 4.87 (m, 2H), 3.99 (dd, J = 30.5, 18.8 Hz, 1H), 3.55 (s, 3H), 3.48 (s, 3H), 3.43 (s, 3H), 2.95 (d, J = 56.3 Hz, 3H), 1.08 (d, J = 6.6 Hz, 6H). **^{13}C NMR** (101 MHz, $CDCl_3$) δ 177.9, 177.5, 171.2, 169.8, 162.96 (d, J = 249.9 Hz), 157.0, 141.0, 137.5, 135.2, 132.2, 131.2, 130.6, 128.7, 128.4, 127.1, 126.3, 124.6, 115.61 (d, J = 21.7 Hz), 114.48 (d, J = 67.0 Hz), 60.4, 51.8, 45.1, 42.4, 33.0, 32.7, 30.7, 21.1, 14.2. **HRMS** (ESI-TOF) [M+H] calculated for $[C_{34}H_{34}Cl_2FN_4O_5S]^+$ 699.1606, found 699.1608. **Specific Rotation** $[\alpha]_D^{25}$ = -9.3 (c = 1.0 in CH_2Cl_2). **HPLC** (Chiralpak-OD-H column, isopropanol/hexane = 30/70, 1.0 mL/min): t(minor) = 5.063 min, t(major) = 4.523 min.

(R)-2,5,7,8-tetramethyl-2-((4R,8S)-4,8,11-trimethyldodecyl)chroman-6-yl 4-(((S)-1-benzyl-4,7-dichloro-3-(2-methoxy-2-oxoethyl)-2-oxoindolin-3-yl)methyl)benzoate (**5ad**)

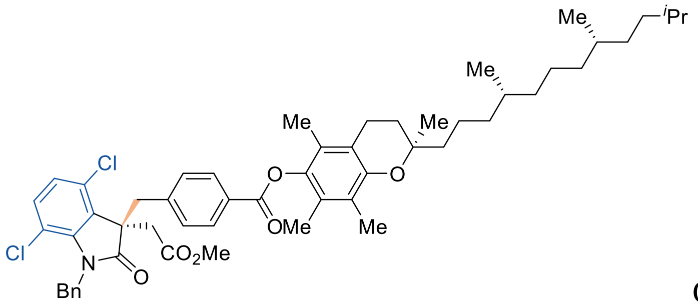

Colorless sticky oil, 70% yield, 98% de, **^{1}H NMR** (400 MHz, $CDCl_3$) δ 8.05 – 7.89 (m, 2H), 7.26 – 7.14 (m, 3H), 7.10 (d, J = 8.8 Hz, 1H), 7.06 – 6.91 (m, 3H), 6.81 (dd, J = 7.5, 2.0 Hz, 2H), 5.31 – 5.07 (m, 2H), 3.76 – 3.64 (m, 2H), 3.56 (s, 3H), 3.41 – 3.15 (m, 2H), 2.65 (t, J = 6.6 Hz, 2H), 2.15 (s, 3H), 2.06 (dd, J = 16.7, 12.8 Hz, 6H), 1.90 – 1.77 (m, 2H), 1.63 – 1.52 (m, 3H),

1.43 (q, J = 8.7, 5.5 Hz, 3H), 1.36 – 1.25 (m, 10H), 1.20 – 1.06 (m, 6H), 0.94 – 0.82 (m, 12H). **^{13}C NMR** (101 MHz, $CDCl_3$) δ 178.1, 169.8, 165.1, 149.5, 141.7, 140.6, 140.3, 137.2, 132.4, 129.98, 129.95, 128.9, 128.6, 128.5, 128.4, 127.3, 126.9, 126.0, 125.1, 124.1, 123.2, 117.5, 114.1, 75.1, 52.7, 52.0, 45.2, 40.3, 39.7, 39.4, 37.5, 37.3, 32.8, 32.7, 28.0, 24.9, 24.5, 22.8, 22.7, 21.1, 20.7, 19.8, 19.7, 13.2, 12.3, 11.9. **HRMS** (ESI-TOF) [M+H] calculated for $[C_{54}H_{68}Cl_2NO_6]^+$ 896.4418, found 896.4421. **Specific Rotation** $[\alpha]_D^{25}$ = +25.6 (c = 1.21 in CH_2Cl_2). **HPLC** (Chiralpak-IB-H column, isopropanol/hexane = 20/80, 1.0 mL/min): t(minor) = 4.693 min, t(major) = 5.367 min.

methyl (S)-2-(1-benzyl-3-cinnamyl-4,6-difluoro-2-oxoindolin-3-yl)acetate (**6a**)

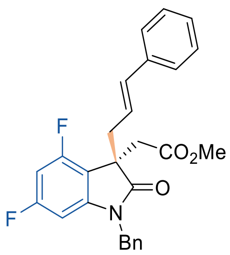

Colorless oil, 77% yield, 96% ee, **^{1}H NMR** (400 MHz, $CDCl_3$) δ 7.28 – 7.24 (m, 3H), 7.22 – 7.14 (m, 5H), 7.04 (dd, J = 8.4, 6.9 Hz, 2H), 6.54 – 6.40 (m, 2H), 6.22 (dd, J = 8.5, 2.1 Hz, 1H), 5.83 – 5.71 (m, 1H), 5.18 (d, J = 15.9 Hz, 1H), 4.63 (d, J = 15.9 Hz, 1H), 3.52 (s, 3H), 3.20 (d, J = 1.1 Hz, 2H), 2.94 – 2.85 (m, 1H), 2.80 (ddd, J = 13.3, 8.3, 1.1 Hz, 1H). **^{13}C NMR** (101 MHz, $CDCl_3$) δ 178.4, 170.1, 163.33 (dd, J = 247.3, 13.0 Hz), 158.43 (dd, J = 247.2, 14.4 Hz), 146.25 (dd, J = 13.8, 12.4 Hz), 136.6, 134.9, 134.9, 128.8, 128.6, 127.6, 127.6, 127.1, 126.4, 122.2, 111.27 (dd, J = 19.7, 3.5 Hz), 99.24 – 96.80 (m), 94.65 (dd, J = 27.6, 3.5 Hz), 51.8, 50.00 (d, J = 2.6 Hz), 44.6, 40.3, 39.8. **^{19}F NMR** (376 MHz, $CDCl_3$) δ -107.84 (d, J = 7.6 Hz), -117.90 (d, J = 7.3 Hz). **HRMS** (ESI-TOF) [M+H] calculated for $[C_{27}H_{24}F_2NO_3]^+$ 448.1719, found 448.1720. **Specific Rotation** $[\alpha]_D^{25}$ = +49.4 (c = 0.69 in CH_2Cl_2). **HPLC** (Chiralpak-IC-H column, isopropanol/hexane = 30/70, 1.0 mL/min): t(minor) = 5.907 min, t(major) = 7.570 min.

methyl (S,E)-4-(1-benzyl-4,7-dichloro-3-(2-methoxy-2-oxoethyl)-2-oxoindolin-3-yl)but-2-enoate (**6b**)

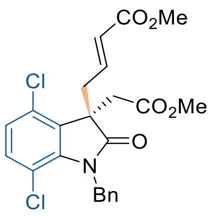

Colorless sticky oil, 67% yield, 98% ee, **^{1}H NMR** (400 MHz, $CDCl_3$) δ 7.34 – 7.25 (m, 5H), 7.16 (d, J = 8.8 Hz, 1H), 6.95 (d, J = 8.8 Hz, 1H), 6.45 (ddd, J = 15.6, 8.5, 7.1 Hz, 1H), 5.83 (dt, J = 15.5, 1.3 Hz, 1H), 5.37 (s, 2H), 3.69 (s, 3H), 3.63 – 3.39 (m, 4H), 3.27 – 3.08 (m, 2H), 2.78 (ddd, J = 13.5, 8.5, 1.1 Hz, 1H). **^{13}C NMR** (101 MHz, $CDCl_3$) δ 178.1, 169.7, 165.9, 141.5, 140.4, 137.4, 132.5, 128.8, 128.6, 128.5, 127.0, 126.4, 125.6, 124.4, 114.3, 51.9, 51.6, 50.6, 45.4, 39.1, 37.5. **HRMS** (ESI-TOF) [M+H] calculated for $[C_{23}H_{22}Cl_2NO_5]^+$ 462.0870, found 462.0870. **Specific Rotation** $[\alpha]_D^{25}$ = +12.2 (c = 0.62 in CH_2Cl_2). **HPLC** (Chiralpak-OD-H column, isopropanol/hexane = 15/85, 1.0 mL/min): t(minor) = 21.007 min, t(major) = 32.390 min.

methyl (S)-2-(1-benzyl-4,7-dichloro-3-(3-methylbut-2-en-1-yl)-2-oxoindolin-3-yl)acetate (**6c**)

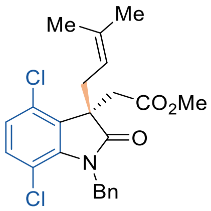

Colorless oil, 69% yield, 98% ee, **^{1}H NMR** (400 MHz, $CDCl_3$) δ 7.43 – 7.21 (m, 5H), 7.11 (dd, J = 8.8, 0.8 Hz, 1H), 6.95 (d, J = 8.7 Hz, 1H), 5.67 – 5.35 (m, 1H), 5.24 (d, J = 16.4 Hz, 1H), 5.09 (d, J = 7.7 Hz, 1H), 4.32 (dd, J = 7.8, 1.9 Hz, 1H), 4.06 (dd, J = 2.0, 0.8 Hz, 1H), 3.59 (d, J = 2.0 Hz, 7H), 3.55 (d, J = 4.7 Hz, 1H), 3.29 (s, 3H). **^{13}C NMR** (101 MHz, $CDCl_3$) δ 174.9, 141.7, 137.3, 131.9, 128.7, 128.7, 128.5, 127.3, 127.0, 126.3, 123.7, 113.7, 104.6, 79.6, 60.9, 56.3, 55.5, 47.9, 44.8. **HRMS** (ESI-TOF) [M+H] calculated for $[C_{23}H_{24}Cl_2NO_3]^+$ 432.1128, found 432.1130. **Specific Rotation** $[\alpha]_D^{25}$ = +40.0 (c = 0.63 in CH_2Cl_2). **HPLC** (Chiralpak-OD-H column, isopropanol/hexane = 30/70, 1.0 mL/min): t(minor) = 4.373 min, t(major) = 5.783 min.

methyl (S)-2-(1-benzyl-4,7-dichloro-3-(2-methylallyl)-2-oxoindolin-3-yl)acetate (**6d**)

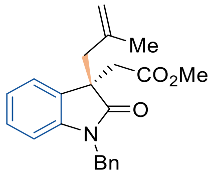

Colorless oil, 75% yield, 94% ee, **^{1}H NMR** (400 MHz, $CDCl_3$) δ 7.43 – 7.30 (m, 4H), 7.30 – 7.21 (m, 2H), 7.15 (dd, J = 7.7, 1.3 Hz, 1H), 7.03 (dd, J = 7.5, 1.1 Hz, 1H), 6.70 (d, J = 7.8 Hz, 1H), 5.02 (d, J = 15.7 Hz, 1H), 4.88 (d, J = 15.7 Hz, 1H), 4.77 – 4.44 (m, 2H), 3.43 (s, 3H), 3.11 (d, J = 16.1 Hz, 1H), 2.95 (d, J = 16.0 Hz, 1H), 2.70 (d, J = 13.1 Hz, 1H), 2.57 (d, J = 12.9 Hz, 1H), 1.32 (d, J = 1.3 Hz, 3H). **^{13}C NMR** (101 MHz, $CDCl_3$) δ 178.7, 170.1, 143.7, 139.8, 136.0, 130.8, 128.6, 128.2, 127.5, 127.4, 123.1, 122.1, 115.9, 109.1, 51.6, 50.2, 45.5, 44.1, 41.6, 24.1. **HRMS** (ESI-TOF) [M+H] calculated for $[C_{22}H_{24}NO_3]^+$ 350.1751, found 350.1750. **Specific Rotation** $[\alpha]_D^{25}$ = -13.4 (c = 0.44 in CH_2Cl_2). **HPLC** (Chiralpak-IC-H column, isopropanol/hexane = 30/70, 1.0 mL/min): t(minor) = 8.983 min, t(major) = 20.063 min.

methyl (S)-2-(1-benzyl-4,6-difluoro-2-oxo-3-(3-phenylprop-2-yn-1-yl)indolin-3-yl)acetate (**6e**)

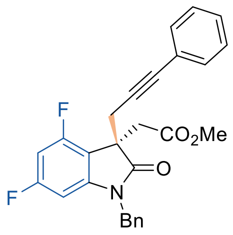

Colorless oil, 81% yield, 96% ee, **^{1}H NMR** (400 MHz, $CDCl_3$) δ 7.38 – 7.31 (m, 2H), 7.29 – 7.20 (m, 3H), 7.19 – 7.14 (m, 1H), 7.14 – 6.86 (m, 4H), 6.50 (td, J = 9.6, 2.1 Hz, 1H), 6.36 – 6.23 (m, 1H), 5.13 (d, J = 15.9 Hz, 1H), 4.78 (d, J = 15.9 Hz, 1H), 3.54 (s, 3H), 3.34 – 3.15 (m, 2H), 3.15 – 2.95 (m, 2H). **^{13}C NMR** (101 MHz, $CDCl_3$) δ 177.9, 169.8, 163.51 (dd, J = 247.4, 13.0 Hz), 158.66 (dd, J = 247.8, 14.3 Hz), 150.90 – 139.63 (m), 134.8, 131.7, 128.8, 128.2, 128.1, 127.6, 127.1, 122.7, 111.22 (dd, J = 19.6, 3.5 Hz), 101.64 – 97.04 (m), 94.57 (dd, J = 27.7, 3.6 Hz), 83.4, 83.1, 52.0, 49.04 (d, J = 2.6 Hz), 44.8, 39.2, 27.9. **^{19}F NMR** (376 MHz, $CDCl_3$) δ -107.64 (d, J = 7.6 Hz), -117.37 (d, J = 7.6 Hz). **HRMS** (ESI-TOF) [M+H] calculated for $[C_{27}H_{22}F_2NO_3]^+$ 446.1562, found 446.1563. **Specific Rotation** $[\alpha]_D^{25}$ = +9.8 (c = 0.72 in CH_2Cl_2). **HPLC** (Chiralpak-IC-H column, isopropanol/hexane = 30/70, 1.0 mL/min): t(minor) = 6.497 min, t(major) = 7.570 min.

Synthetic derivatization:

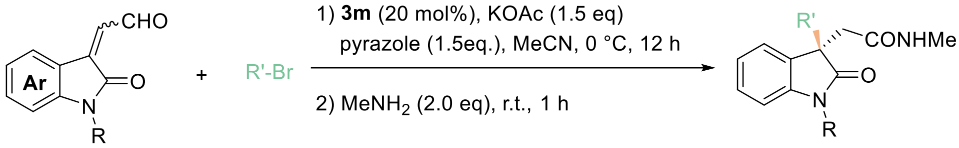

The catalyst precursor **3m** (10.9 mg, 20 mol%), enal **1** (0.1 mmol, 1.0 eq) and KOAc (15 mg, 1.5 eq) were mixed in MeCN (1.0 mL) in a 20-mL test tube. The reaction vessel was degassed and back-filled with argon, sealed with a rubber septum and stirred at 0 °C. for 10 min. Then, R'Br (0.15 mmol, 1.5 eq) and pyrazole (40 μL, 1.5 eq) were added. And the mixture was stirred at 0 °C for 12 hours. After consuming **1**, the reaction was warmed to room temperature and $MeNH_2$ (0.2 mmol, 2.0 eq) was added. Then the reaction was stirred for another 1 hour. Finally, the mixture was quickly filtered through a plug of silica gel and concentrated. The residue was purified by silica gel flash column chromatography [eluent: hexane (100mL), then hexane / EtOAc = 1:3) to afford corresponding products. The ee was determined by chiral HPLC.

(S)-2-(1,3-bis(3-methylbut-2-en-1-yl)-2-oxoindolin-3-yl)-N-methylacetamide (**7a**)

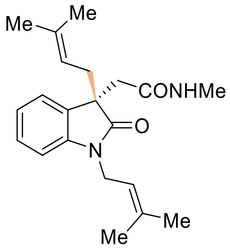

Colorless oil, 60% yield, 90% ee. **^{1}H NMR** (400 MHz, Chloroform-d) δ 7.28 – 7.20 (m, 2H), 7.06 (td, J = 7.5, 1.0 Hz, 1H), 6.80 (dd, J = 7.7, 1.2 Hz, 1H), 6.46 (s, 1H), 5.10 (ddt, J = 6.6, 4.9, 1.4 Hz, 1H), 4.78 (dddd, J = 7.6, 6.1, 2.9, 1.5 Hz, 1H), 4.53 – 4.36 (m, 1H), 4.25 (dd, J = 15.5, 6.8 Hz, 1H), 2.83 (d, J = 14.7 Hz, 1H), 2.54 (d, J = 7.6 Hz, 2H), 1.85 (d, J = 1.4 Hz, 3H), 1.73 (d, J = 1.4 Hz, 3H), 1.57 (d, J = 1.4 Hz, 3H), 1.47 (d, J = 1.4 Hz, 3H). **^{13}C NMR** δ (101 MHz, Chloroform-d) 179.60, 169.74, 142.56, 136.42, 136.00, 131.50, 128.03, 123.39, 122.48, 118.49, 117.04, 108.67, 50.67, 42.52, 38.08, 35.90, 26.18, 25.82, 25.68, 18.16, 17.96. **HRMS** (ESI-TOF) [M+H] calculated for $[C_{21}H_{29}N_2O_2]^+$ 341.2224, found 341.2221. **Specific Rotation** $[\alpha]_D^{25}$ = -24.9 (c = 0.50 in CH_2Cl_2). **HPLC** (Chiralpak-OD-H column, isopropanol/hexane = 30/70, 1.0 mL/min): t(minor) = 4.213 min, t(major) = 4.863 min.

(S)-2-(6-bromo-1,3-bis(3-methylbut-2-en-1-yl)-2-oxoindolin-3-yl)-N-methylacetamide (**7b**)

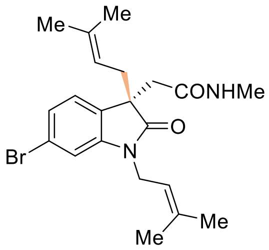

Colorless oil, 64% yield, 91% ee. **^{1}H NMR** (400 MHz, Chloroform-d) δ 7.17 (dd, J = 7.9, 1.7 Hz, 1H), 7.11 (d, J = 7.9 Hz, 1H), 6.92 (d, J = 1.8 Hz, 1H), 6.06 (d, J = 6.6 Hz, 1H), 5.07 (ddt, J = 6.6, 4.9, 1.4 Hz, 1H), 4.76 (tt, J = 7.6, 1.5 Hz, 1H), 4.54 – 4.32 (m, 1H), 4.20 (dd, J = 15.6, 6.8 Hz, 1H), 2.49 (d, J = 7.7 Hz, 2H), 1.84 (d, J = 1.4 Hz, 3H), 1.75 (d, J = 1.5 Hz, 3H), 1.58 (d, J = 1.4 Hz, 3H), 1.47 (d, J = 1.4 Hz, 3H). **^{13}C NMR** (101 MHz, Chloroform-d) δ 179.31, 169.39, 144.19, 136.92, 136.39, 130.45, 125.05, 124.58, 121.52, 118.00, 116.67, 112.10, 50.51, 42.17, 38.18, 36.05, 26.19, 25.84, 25.69, 18.19, 18.00. **HRMS** (ESI-TOF) [M+H] calculated for $[C_{21}H_{27}BrN_2O_2]^+$ 419.1329, found 419.1330. **Specific Rotation** $[\alpha]_D^{25}$ = -21.0 (c = 0.60 in CH_2Cl_2). **HPLC** (Chiralpak-IC-H column, isopropanol/hexane = 30/70, 1.0 mL/min): t(minor) = 17.200 min, t(major) = 24.673 min.

Methyl (S,E)-2-methyl-4-(3-(2-(methylamino)-2-oxoethyl)-2-oxoindolin-3-yl)but-2-enoate (**7c**)

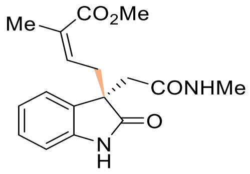

Colorless oil, 61% yield, 90% ee. **^{1}H NMR** (400 MHz, Chloroform-d) δ 8.09 (s, 1H), 7.23 (td, J = 6.6, 5.8, 1.5 Hz, 2H), 7.05 (td, J = 7.6, 1.0 Hz, 1H), 6.89 (dd, J = 8.1, 1.0 Hz, 1H), 6.52 (td, J = 7.7, 1.5 Hz, 1H), 6.00 (s, 1H), 3.68 (s, 3H), 2.98 – 2.71 (m, 4H), 2.68 (d, J = 4.5 Hz, 3H), 1.75 (d, J = 1.5 Hz, 3H). **^{13}C NMR** (101 MHz, Chloroform-d) δ 180.83, 169.25, 168.10, 140.54, 134.70, 131.12, 131.07, 128.54, 123.46, 122.66, 110.10, 51.87, 50.18, 42.28, 36.15, 26.27, 12.73. **HRMS** (ESI-TOF) [M+H] calculated for $[C_{17}H_{21}N_2O_4]^+$ 317.1496, found 317.1497. **Specific Rotation** $[\alpha]_D^{25}$ = +17.8 (c = 0.20 in CH_2Cl_2). **HPLC** (Chiralpak-IB-H column, isopropanol/hexane = 30/70, 1.0 mL/min): t(minor) = 4.600 min, t(major) = 7.873 min.

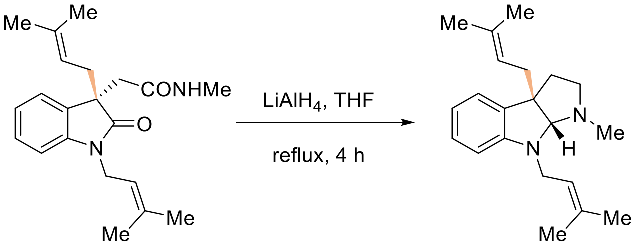

7a **8a**, 60% yield, dr > 20:1

To a solution of **7a** (0.05 mmol) in THF (1.0 mL) was slowly added $LiAlH_4$ (0.1 mL, 10.0 eq, 1M in THF). Then it was heated to reflux for 4 hours, with the reaction progress monitored by TLC. After the reaction, it was slowly quenched by H_2O/THF (1:1, 1 mL)

at 0 °C. Then the mixture was filtered and extracted with EA three times. The collected organic phase was concentrated under reduced pressure. The residue was purified by column chromatography to offer the (-)-debromoflustramine B (**8a**) in 60% yield[1,2].

(3aS,8aR)-1-methyl-3a,8-bis(3-methylbut-2-en-1-yl)-1,2,3,3a,8,8a-hexahydropyrrolo[2,3-b]indole (**8a**)

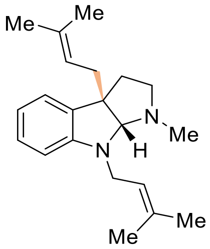

Colorless oil, 60% yield, dr > 20:1. **^{1}H NMR** (400 MHz, Chloroform-d) δ 7.07 (td, J = 7.6, 1.3 Hz, 1H), 7.00 (dd, J = 7.3, 1.3 Hz, 1H), 6.68 (td, J = 7.4, 1.0 Hz, 1H), 6.44 (d, J = 7.9 Hz, 1H), 5.27 – 5.12 (m, 1H), 5.07 – 4.92 (m, 1H), 4.31 (s, 1H), 3.95 (dd, J = 15.9, 5.4 Hz, 1H), 3.83 (dd, J = 16.1, 7.2 Hz, 1H), 2.71 (dt, J = 6.6, 3.1 Hz, 1H), 2.58 (td, J = 9.3, 5.8 Hz, 1H), 2.51 (s, 3H), 2.45 (d, J = 7.3 Hz, 2H), 2.12 – 2.06 (m, 1H), 1.94 (ddd, J = 11.9, 5.8, 3.3 Hz, 1H), 1.73 (d, J = 1.2 Hz, 3H), 1.72 (d, J = 1.4 Hz, 3H), 1.67 (d, J = 1.4 Hz, 3H), 1.60 (d, J = 1.3 Hz, 3H). **^{13}C NMR** δ (101 MHz, Chloroform-d) δ 151.9, 135.7, 134.2, 133.6, 127.6, 122.9, 121.4, 120.7, 117.5, 107.4, 91.3, 57.1, 52.8, 46.9, 39.0, 38.5, 37.9, 26.0, 25.8, 18.1, 18.1. **HRMS** (ESI-TOF) [M+H] calculated for $[C_{21}H_{31}N_2]^+$ 311.2482, found 311.2482. **Specific Rotation** $[\alpha]_D^{24}$ = +75.6 (c = 0.22 in $CHCl_3$). Lit. : $[\alpha]_D^{24}$ = +76.9 (c = 0.30, $CHCl_3$) [1].

X-ray crystal structure:

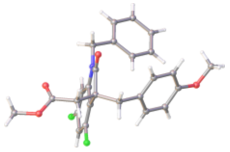

ORTEP drawing of the compound **5l** (CCDC 2259915)

Table S1 Crystal data and structure refinement for **5l**

Identification code	5l
Empirical formula	$C2_6H_{23}F_2NO_4$
Formula weight	451.445
Temperature/K	100(2)
Crystal system	orthorhombic
Space group	$P2_12_12_1$
a/Å	9.04344(4)
b/Å	9.29871(4)
c/Å	26.11808(10)
α/°	90
β/°	90
γ/°	90
Volume/Å^3	2196.328(16)
Z	4
ρ_{calc}g/cm^3	1.365
μ/mm-1	0.863
F(000)	944.0
Crystal size/mm^3	0.2 × 0.2 × 0.2
Radiation	Cu Kα (λ = 1.54184)
2Θ range for data collection/°	6.768 to 154.538
Index ranges	-11 ≤ h ≤ 11, -11 ≤ k ≤ 11, -32 ≤ l ≤ 32
Reflections collected	59378
Independent reflections	4569 [R_{int} = 0.0260, R_{sigma} = 0.0095]
Data/restraints/parameters	1569/0/301
Goodness-of-fit on F^2	1.061
Final R indexes [I>=2σ (I)]	R_1 = 0.0235, wR_2 = 0.0597
Final R indexes [all data]	R_1 = 0.0235, wR_2 = 0.0597
Largest diff. peak/hole / e Å^{-3}	0.21/-0.17
Flack parameter	-0.019()

Table S2: Fractional Atomic Coordinates ($\times 10^4$) and Equivalent Isotropic Displacement Parameters ($Å^2 \times 10^3$) for **5l**. U_{eq} is defined as 1/3 of the trace of the orthogonalized U_{ij}.

Atom	x	y	z	U_{eq}
F26	11235.8(9)	7706.4(10)	4181.9(3)	23.8(2)
F27	8642.7(11)	5012.3(10)	5410.8(3)	27.0(2)
O33	5850.5(12)	8619.5(12)	3259.2(4)	24.6(2)
O24	7884.3(12)	1772.8(12)	2656.9(4)	23.3(2)
O30	9408.9(13)	11465.6(12)	4298.2(4)	28.7(3)
O32	7369.3(14)	10146.5(13)	4427.4(5)	32.4(3)
N1	6203.6(13)	7094.4(13)	3946.7(4)	17.6(2)
C5	9952.3(16)	7115.5(15)	4353.5(5)	18.2(3)
C4	8685.0(15)	7330.0(15)	4076.5(5)	16.8(3)
C21	8103.0(16)	3217.5(16)	2733.7(5)	19.7(3)
C9	7379.2(15)	6714.9(15)	4263.6(5)	16.9(3)
C2	6651.8(16)	8049.4(15)	3571.4(5)	18.2(3)
C8	7328.1(17)	5913.4(15)	4709.6(5)	18.9(3)
C6	9987.2(17)	6321.3(16)	4803.8(5)	20.1(3)
C22	9216.2(16)	3556.2(17)	3083.3(5)	20.6(3)
C20	7311.0(17)	4305.8(17)	2494.6(5)	21.9(3)
C7	8655.0(17)	5760.0(15)	4968.3(5)	20.0(3)
C16	3040.7(16)	4468.1(17)	4011.9(6)	21.7(3)
C17	9050.3(16)	7643.5(16)	3103.7(5)	21.0(3)
C11	4421.3(15)	5056.9(16)	3897.9(5)	17.7(3)
C19	7627.7(16)	5736.1(17)	2618.5(5)	21.5(3)
C18	8714.9(16)	6097.4(16)	2973.4(5)	19.5(3)
C12	5488.4(17)	4179.8(17)	3676.4(6)	23.5(3)
C15	2732.2(17)	3033.9(17)	3903.8(6)	24.6(3)
C23	9512.2(16)	4977.0(17)	3200.6(6)	20.5(3)
C29	8416.3(17)	10459.5(15)	4168.7(6)	21.0(3)
C10	4684.4(15)	6635.8(16)	4021.3(5)	19.4(3)
C14	3805.3(18)	2166.5(17)	3684.3(6)	25.4(3)
C3	8349.1(15)	8215.1(15)	3607.9(5)	17.7(3)
C28	8803.1(16)	9796.6(15)	3659.3(6)	20.2(3)
C13	5181.8(18)	2738.5(17)	3572.9(6)	26.6(3)
C25	6811(2)	1349.4(19)	2288.6(7)	31.1(4)
C31	9124(2)	12215(2)	4774.1(7)	37.7(4)

Table S3: Anisotropic Displacement Parameters ($Å^2 \times 10^3$) **5l**. The anisotropic displacement factor exponent is $-2\pi^2[h^2a^{*2} ?U_{11} + 2hka^*b^*U_{12}+...]$

**Atom**	U_{11}	U_{22}	U_{33}	U_{23}	U_{13}	U_{12}
F26	13.0(4)	30.2(5)	28.3(4)	0.7(4)	0.0(3)	-3.0(3)
F27	34.2(5)	28.6(5)	18.3(4)	6.5(3)	-1.8(4)	2.6(4)
O33	20.4(5)	28.0(5)	25.3(5)	6.2(4)	-5.9(4)	0.8(4)
O24	23.9(5)	23.7(5)	22.3(5)	-2.5(4)	-4.7(4)	0.8(4)
O30	30.2(6)	25.6(5)	30.5(6)	-6.9(5)	9.4(5)	-9.0(5)
O32	29.2(6)	28.6(6)	39.4(6)	-3.5(5)	16.0(5)	-6.1(5)
N1	13.0(5)	20.3(6)	19.4(5)	2.0(5)	-0.7(4)	-1.2(4)
C5	13.5(6)	19.6(6)	21.4(6)	-3.2(5)	1.3(5)	-0.6(5)
C4	16.1(6)	17.3(6)	17.1(6)	-0.8(5)	0.8(5)	-0.2(5)
C21	17.2(6)	25.1(7)	16.8(6)	-1.8(5)	3.1(5)	0.7(6)
C9	14.9(6)	17.4(6)	18.4(6)	-2.5(5)	-0.4(5)	0.3(5)
C2	17.2(6)	18.1(6)	19.4(7)	0.0(5)	-0.2(5)	-0.3(5)
C8	18.9(7)	18.4(6)	19.5(6)	0.1(5)	3.1(5)	-1.2(5)
C6	18.8(6)	21.3(7)	20.3(6)	-2.9(5)	-4.7(5)	3.4(6)
C22	18.1(7)	26.5(7)	17.3(6)	0.9(6)	-0.4(5)	2.1(6)
C20	18.6(7)	29.3(7)	17.9(6)	-1.7(6)	-2.0(5)	0.3(6)
C7	26.6(7)	18.4(6)	15.0(6)	-0.5(5)	-0.2(5)	3.2(6)
C16	15.9(7)	25.7(7)	23.4(7)	1.2(6)	0.3(5)	-0.8(6)
C17	19.3(7)	25.4(7)	18.2(6)	2.2(6)	2.5(5)	-1.9(6)
C11	15.4(6)	22.6(7)	15.0(6)	1.5(5)	-1.3(5)	-1.6(5)
C19	19.4(7)	27.1(7)	17.9(6)	1.0(6)	-0.1(6)	3.4(6)
C18	16.7(6)	25.3(7)	16.6(6)	0.3(5)	3.8(5)	-0.6(6)
C12	19.7(7)	26.8(7)	23.9(7)	-1.7(6)	5.1(6)	-4.2(6)
C15	20.1(7)	28.0(8)	25.8(7)	3.2(6)	-1.1(6)	-7.0(6)
C23	15.3(6)	28.7(8)	17.4(6)	-1.0(6)	-0.6(5)	0.1(5)
C29	19.9(7)	15.8(6)	27.4(7)	3.8(6)	2.8(6)	0.2(5)
C10	12.5(6)	23.3(7)	22.3(7)	-1.1(6)	1.0(5)	-0.8(5)
C14	33.7(8)	22.4(7)	19.9(7)	0.6(6)	-2.4(6)	-5.8(6)
C3	14.4(6)	19.8(7)	18.7(6)	2.1(5)	0.0(5)	-1.6(5)
C28	17.8(7)	20.1(7)	22.8(7)	3.5(5)	1.5(6)	-3.0(5)
C13	27.7(8)	25.8(8)	26.4(7)	-3.5(6)	5.9(6)	0.8(6)
C25	31.4(8)	28.9(8)	33.2(8)	-4.9(7)	-11.8(7)	-1.4(7)
C31	47.7(11)	30.1(9)	35.5(9)	-12.0(7)	12.3(8)	-8.2(8)

Table S4: Bond Lengths for **5l**

Atom	Atom	Length/°
F26	C5	1.3601(16)
F27	C7	1.3488(16)
O33	C2	1.2129(18)
O24	C21	1.3726(18)
O24	C25	1.4220(18)
O30	C29	1.3400(18)
O30	C31	1.448(2)
O32	C29	1.1991(19)
N1	C2	1.3832(18)
N1	C9	1.3928(18)
N1	C10	1.4517(17)
C5	C4	1.370(2)
C5	C6	1.389(2)
C4	C9	1.4002(19)
C4	C3	1.5059(19)
C21	C20	1.388(2)
C21	C22	1.395(2)
C9	C8	1.3837(19)
C2	C3	1.5456(19)
C8	C7	1.384(2)
C6	C7	1.381(2)
C22	C23	1.382(2)
C20	C19	1.398(2)
C16	C15	1.391(2)
C16	C11	1.395(2)
C17	C18	1.508(2)
C17	C3	1.555(2)
C11	C12	1.390(2)
C11	C10	1.522(2)
C19	C18	1.392(2)
C18	C23	1.399(2)
C12	C13	1.395(2)
C15	C14	1.386(2)
C29	C28	1.508(2)
C14	C13	1.385(2)
C3	C28	1.533(2)

Table S5: Bond Angless for **5l**

Atom	Atom	Atom	Angle/°
C21	O24	C25	117.92(12)
C29	O30	C31	115.70(13)
C2	N1	C9	111.11(11)
C2	N1	C10	124.13(12)
C9	N1	C10	124.62(11)
F26	C5	C4	118.76(12)
F26	C5	C6	118.32(13)
C4	C5	C6	122.92(14)
C5	C4	C9	117.51(12)
C5	C4	C3	132.65(13)
C9	C4	C3	109.68(12)
O24	C21	C20	124.98(13)
O24	C21	C22	114.88(13)
C20	C21	C22	120.14(14)
C8	C9	N1	127.68(13)
C8	C9	C4	122.82(13)
N1	C9	C4	109.44(12)
O33	C2	N1	125.59(13)
O33	C2	C3	126.24(13)
N1	C2	C3	108.14(12)
C9	C8	C7	115.94(13)
C7	C6	C5	116.38(13)
C23	C22	C21	120.04(14)
C21	C20	C19	118.92(13)
F27	C7	C6	117.93(13)
F27	C7	C8	117.65(13)
C6	C7	C8	124.42(13)
C15	C16	C11	120.81(14)
C18	C17	C3	115.77(12)
C12	C11	C16	118.68(14)
C12	C11	C10	123.08(13)
C16	C11	C10	118.24(13)
C18	C19	C20	121.88(14)
C19	C18	C23	117.81(14)
C19	C18	C17	121.48(13)
C23	C18	C17	120.69(13)
C11	C12	C13	120.43(14)
C14	C15	C16	120.08(14)
C22	C23	C18	121.18(14)
O32	C29	O30	123.79(14)
O32	C29	C28	125.54(14)
O30	C29	C28	110.67(12)

N1	C10	C11	113.76(12)
C13	C14	C15	119.53(14)
C4	C3	C28	113.52(11)
C4	C3	C2	101.30(11)
C28	C3	C2	111.53(12)
C4	C3	C17	114.78(12)
C28	C3	C17	107.03(11)
C2	C3	C17	108.59(12)
C29	C28	C3	114.05(11)
C14	C13	C12	120.47(15)

Table S6: Torsion Angles for **5l**

Atom	Atom	Atom	Atom	Angle/°
F26	C5	C4	C9	-179.70(12)
C6	C5	C4	C9	0.2(2)
F26	C5	C4	C3	-4.9(2)
C6	C5	C4	C3	175.02(14)
C25	O24	C21	C20	2.9(2)
C25	O24	C21	C22	-177.28(13)
C2	N1	C9	C8	172.47(14)
C10	N1	C9	C8	-3.3(2)
C2	N1	C9	C4	-4.91(16)
C10	N1	C9	C4	179.35(12)
C5	C4	C9	C8	0.0(2)
C3	C4	C9	C8	-176.01(13)
C5	C4	C9	N1	177.49(12)
C3	C4	C9	N1	1.52(16)
C9	N1	C2	O33	-175.94(14)
C10	N1	C2	O33	-0.2(2)
C9	N1	C2	C3	6.13(16)
C10	N1	C2	C3	-178.10(12)
N1	C9	C8	C7	-176.46(13)
C4	C9	C8	C7	0.6(2)
F26	C5	C6	C7	179.00(12)
C4	C5	C6	C7	-0.9(2)
O24	C21	C22	C23	-178.47(13)
C20	C21	C22	C23	1.4(2)
O24	C21	C20	C19	178.48(14)
C22	C21	C20	C19	-1.3(2)
C5	C6	C7	F27	-178.43(12)
C5	C6	C7	C8	1.5(2)
C9	C8	C7	F27	178.57(12)
C9	C8	C7	C6	-1.4(2)
C15	C16	C11	C12	-0.4(2)
C15	C16	C11	C10	179.47(13)
C21	C20	C19	C18	0.1(2)
C20	C19	C18	C23	1.0(2)
C20	C19	C18	C17	179.88(13)
C3	C17	C18	C19	99.83(16)
C3	C17	C18	C23	-81.35(17)
C16	C11	C12	C13	-0.3(2)
C10	C11	C12	C13	179.90(14)
C11	C16	C15	C14	0.6(2)
C21	C22	C23	C18	-0.2(2)
C19	C18	C23	C22	-1.0(2)

C17	C18	C23	C22	-179.87(13)
C31	O30	C29	O32	-1.9(2)
C31	O30	C29	C28	177.97(14)
C2	N1	C10	C11	112.43(15)
C9	N1	C10	C11	-72.37(17)
C12	C11	C10	N1	-7.33(19)
C16	C11	C10	N1	172.85(12)
C16	C15	C14	C13	-0.1(2)
C5	C4	C3	C28	-53.5(2)
C9	C4	C3	C28	121.64(13)
C5	C4	C3	C2	-173.18(15)
C9	C4	C3	C2	1.96(15)
C5	C4	C3	C17	70.1(2)
C9	C4	C3	C17	-114.80(13)
O33	C2	C3	C4	177.29(14)
N1	C2	C3	C4	-4.79(15)
O33	C2	C3	C28	56.20(19)
N1	C2	C3	C28	-125.88(12)
O33	C2	C3	C17	-61.50(19)
N1	C2	C3	C17	116.42(12)
C18	C17	C3	C4	54.12(17)
C18	C17	C3	C28	-178.93(12)
C18	C17	C3	C2	-58.40(16)
O32	C29	C28	C3	-31.7(2)
O30	C29	C28	C3	148.38(12)
C4	C3	C28	C29	-43.27(17)
C2	C3	C28	C29	70.40(15)
C17	C3	C28	C29	-170.96(12)
C15	C14	C13	C12	-0.5(2)
C11	C12	C13	C14	0.7(2)

Table S7: Hydrogen Atom Coordinates (Å×104) and Isotropic Displacement Parameters (Å2×103) for **5l**

**Atom**	**x**	**y**	**z**	**U(eq)**
H8	6436.7	5494.01	4830.98	23
H6	10878.8	6172.19	4988.64	24
H22	9771.1	2810.01	3240.79	25
H20	6566.03	4082.58	2250.82	26
H16	2302.48	5053.59	4165.21	26
H17A	8706.1	8255.84	2817.43	25
H17B	10136.69	7755.91	3127.84	25
H19	7085.57	6482.54	2455.94	26
H12	6432.77	4564.23	3594.82	28
H15	1784.81	2648.69	3980.55	30
H23	10270.69	5196.54	3439.85	25
H10A	4032.2	7229.55	3802.24	23
H10B	4402.63	6813.5	4382.09	23
H14	3597.78	1186.53	3610.73	30
H28A	9884.56	9872.82	3607.15	24
H28B	8315.13	10356.06	3384.57	24
H13	5922.74	2145.17	3425.06	32
H25A	7106.4	1698.52	1949.63	47
H25B	5848.64	1759.93	2380.1	47
H25C	6738.62	298.03	2282.21	47
H31A	8178.11	12724.81	4750.14	57
H31B	9919.05	12908.19	4838.46	57
H31C	9082.4	11519.76	5055.65	57

Computational Data:

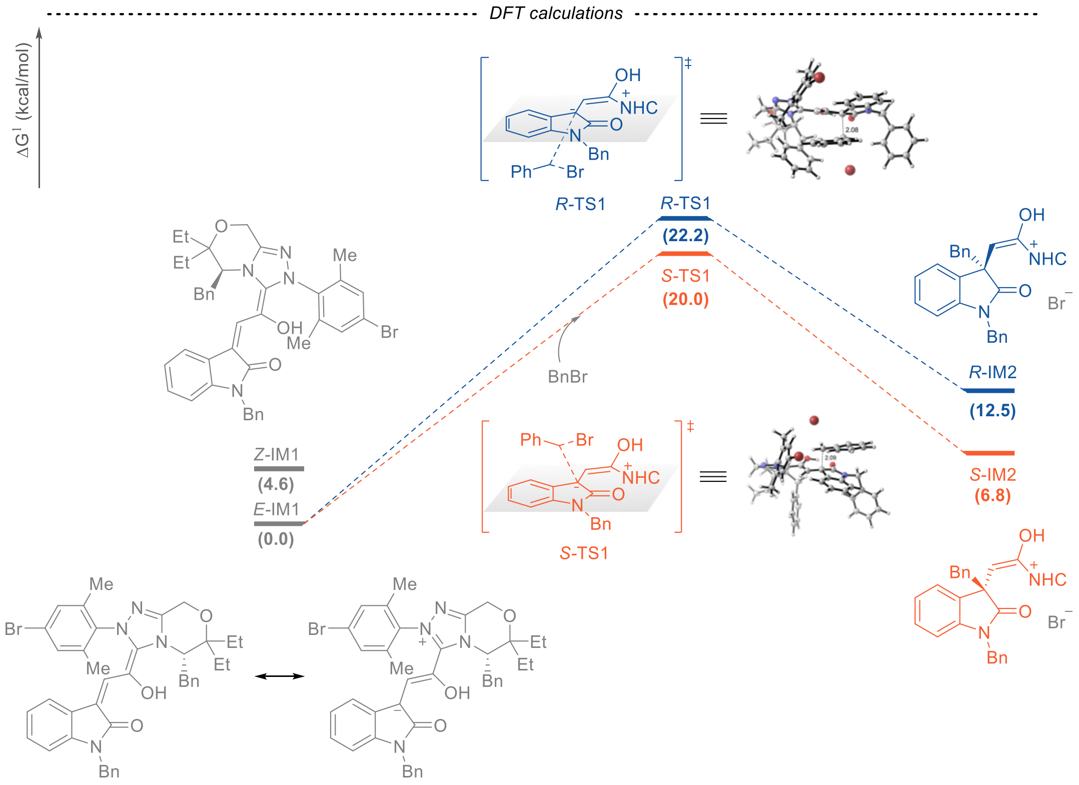

Figure S7. DFT calculations. Gibbs free energies were given in kcal/mol at the theoretical level of B3LYP-D3/6-31G(d)//B3LYP-D3/6-311++G(d,p). (color code, C: grey, N: blue, O: red, H: white, Br reddish brown.).

Result:

DFT calculations were performed to investigate the possible nucleophilic attack process of the homoenolate intermediate with benzyl bromide (**Figure 1**). The program determined that the *E*- homoenolate is 4.6 kcal/mol lower than the *Z*- homoenolate. This illustrates that *E*-homoenolate intermediates would be generated from free NHC and enals. Moreover, the energy of the transition state *S*-TSI formed by nucleophilic substitution took place at the *Re*-face of the *E*-homoenolate is 2.2 kcal/mol lower than that of the transition state *R*-TSI formed by nucleophilic substitution took place at the *Si*-face of the *E*-homoenolate, which is consistent with our observation of 93% ee value. In addition, the Gibbs free energy of *S*-IM2 is stable at 5.7 kcal/mol compared to *R*-IM2. This indicates that the intermediate mainly engages in nucleophilic attacks to benzyl bromide from the *Re*-face of the *E*-homoenolate, resulting in corresponding *S*-shaped products *S*-IM2.

Computational Details

All calculations were conducted using the Gaussian 16 software package.[3] All stationary points were optimized at B3LYP-D3/6-31G(d) theoretical level.[4] Frequency calculations were performed at the same level to verify the stationary points are minima (0 imaginary frequency). Single point calculations were carried out with B3LYP-D3/6-

311++G(d,p) theoretical level for all atoms. Dispersion effects are described using Grimme's D3 corrections. Computed structures were illustrated by CYLView software.[5]

Coordinates for all stationary points

E-IM1

C	5.18650400	0.97363300	-0.83659100
C	3.62645600	1.05793200	-0.96641900
C	3.80009800	-1.43170500	-0.61374700
C	5.26892100	-1.41739200	-0.90075600
H	5.50529900	-2.22373000	-1.60022300
N	3.04384600	-0.29355000	-0.78678700
O	5.61736400	-0.20983400	-1.54385600
C	5.79632300	2.18121000	-1.60232400
C	7.07511400	1.88688500	-2.39456100
H	5.98513000	2.98296400	-0.87914100
H	5.05172100	2.57574300	-2.30320800
H	7.42250000	2.80711700	-2.87679300
H	7.88187600	1.51115700	-1.76153000
H	6.88900800	1.14111000	-3.17025300
H	5.85087200	-1.58804300	0.01409700
C	5.68038500	0.93037500	0.62459500
C	7.18689000	0.70369800	0.78820400
H	5.12273300	0.17466300	1.18959900
H	5.41886100	1.87532800	1.09741900
H	7.42719400	0.54954200	1.84525200
H	7.54239200	-0.16553400	0.22722200
H	7.75949400	1.57073100	0.44574700
C	1.71168700	-0.66964100	-0.69349100
N	1.77670100	-2.00243900	-0.34834800
N	3.08079800	-2.47198300	-0.31987400
C	0.68809000	-2.89476000	-0.12863800
C	-0.07396800	-3.32434600	-1.22572500
C	0.37161600	-3.23502400	1.19530500
C	-1.23847600	-4.05136600	-0.96679800
C	-0.79325400	-3.97128100	1.42302700
C	-1.59569500	-4.33874800	0.34673100
H	-1.87821800	-4.36342500	-1.78432500
H	-1.09019600	-4.22490300	2.43431000
C	1.24568700	-2.78661400	2.33721600
H	2.23719500	-3.24770000	2.27250700
H	1.40287000	-1.70328800	2.31955400
H	0.79924500	-3.05496900	3.29808700
C	0.30918400	-2.95539800	-2.63370700

H	*0.06165600*	*-1.90777000*	*-2.84041500*
H	*1.38496600*	*-3.07734600*	*-2.79426500*
H	*-0.22331400*	*-3.58016300*	*-3.35538000*
C	*0.58030300*	*0.10485800*	*-1.03081000*
O	*0.83983100*	*1.12874200*	*-1.90861300*
H	*0.05665200*	*1.76744000*	*-1.91102100*
C	*-0.68790300*	*-0.21252000*	*-0.54513600*
H	*-0.73765900*	*-1.07608300*	*0.10606200*
C	*-3.21013800*	*-0.10963800*	*-0.38487600*
C	*-4.18167100*	*0.84242600*	*-0.77974900*
C	*-5.53849300*	*0.64742800*	*-0.56251800*
C	*-5.93557000*	*-0.54957400*	*0.04961500*
C	*-4.99294300*	*-1.51271900*	*0.42545200*
C	*-3.62832500*	*-1.29953700*	*0.21103600*
C	*-1.90995200*	*0.40997500*	*-0.77246900*
C	*-2.14727800*	*1.70138800*	*-1.39537300*
H	*-6.26927800*	*1.39668800*	*-0.84869200*
H	*-6.99190100*	*-0.72870500*	*0.22855800*
H	*-5.31518800*	*-2.44572100*	*0.87885300*
H	*-2.90999400*	*-2.05916900*	*0.49889000*
O	*-1.33141900*	*2.55659100*	*-1.82210600*
N	*-3.52037100*	*1.90845300*	*-1.40187600*
C	*-4.13539200*	*3.13701900*	*-1.86900900*
H	*-3.40028300*	*3.60520600*	*-2.52941400*
H	*-5.01973400*	*2.88471000*	*-2.46460700*
C	*-4.50673800*	*4.07266900*	*-0.73675500*
C	*-3.50591100*	*4.56063900*	*0.11335100*
C	*-5.83234300*	*4.44990600*	*-0.51554800*
C	*-3.83262400*	*5.40960800*	*1.16780400*
H	*-2.47424200*	*4.26952300*	*-0.06401900*
C	*-6.16176100*	*5.30329900*	*0.53982700*
H	*-6.61337900*	*4.07375600*	*-1.17277200*
C	*-5.16208900*	*5.78312300*	*1.38449200*
H	*-3.04964400*	*5.78346500*	*1.82202400*
H	*-7.19785700*	*5.58712600*	*0.70304500*
H	*-5.41527400*	*6.44457900*	*2.20845100*
H	*3.40835800*	*1.32755700*	*-2.00188400*
C	*2.95859200*	*2.16864500*	*-0.10627800*
H	*3.49962500*	*3.08630600*	*-0.36350100*
C	*2.88250900*	*2.05632300*	*1.40285400*
C	*3.57581700*	*2.96945900*	*2.20974600*
C	*2.06637100*	*1.11138000*	*2.03893200*
C	*3.50047000*	*2.91451000*	*3.60071500*
H	*4.18376000*	*3.73858200*	*1.73801100*

C	*1.99025600*	*1.04808600*	*3.43040300*
H	*1.44845500*	*0.45214800*	*1.44169800*
C	*2.71492100*	*1.94157400*	*4.21899400*
H	*4.05091100*	*3.63487300*	*4.19947800*
H	*1.34634800*	*0.30798000*	*3.89794600*
H	*2.65208900*	*1.89375100*	*5.30230600*
H	*1.95331000*	*2.29176600*	*-0.51075100*
Br	*-3.26581200*	*-5.19169600*	*0.69073600*

Z-IM1

C	*0.89000500*	*4.02066700*	*0.66276800*
C	*0.82427600*	*2.47626000*	*0.92657500*
C	*-1.63278500*	*2.98881300*	*0.86504100*
C	*-1.39970600*	*4.41369300*	*1.25735300*
H	*-1.97343400*	*4.64487400*	*2.16038800*
N	*-0.60943000*	*2.05675500*	*0.89787000*
O	*-0.04312200*	*4.62794100*	*1.58198600*
C	*2.29234700*	*4.52482400*	*1.09456400*
C	*2.33139000*	*5.91323200*	*1.74312000*
H	*2.94692500*	*4.50515700*	*0.21594900*
H	*2.72239000*	*3.81385000*	*1.81043000*
H	*3.36708000*	*6.16364900*	*1.99710600*
H	*1.94734200*	*6.69524000*	*1.08526300*
H	*1.73854600*	*5.93014200*	*2.65984600*
H	*-1.75040200*	*5.08201000*	*0.45992500*
C	*0.55511500*	*4.40701300*	*-0.79352200*
C	*0.47928400*	*5.91354000*	*-1.06339600*
H	*-0.38376000*	*3.93109700*	*-1.09875700*
H	*1.31066600*	*3.96986900*	*-1.44411000*
H	*0.07630400*	*6.09109900*	*-2.06571400*
H	*-0.15850900*	*6.43416800*	*-0.34292600*
H	*1.46917300*	*6.37737600*	*-1.02291700*
C	*-1.20323600*	*0.82315600*	*0.66215600*
N	*-2.49857100*	*1.12334600*	*0.34440900*
N	*-2.76999400*	*2.47131000*	*0.51554200*
C	*-3.59182100*	*0.22032800*	*0.14551500*
C	*-4.29338200*	*-0.22535400*	*1.27188900*
C	*-3.92367100*	*-0.16128200*	*-1.15865900*
C	*-5.38137100*	*-1.07788800*	*1.07142900*
C	*-5.01843700*	*-1.01111400*	*-1.33173900*
C	*-5.72933900*	*-1.45481600*	*-0.22154600*
H	*-5.95024200*	*-1.44483000*	*1.91819000*
H	*-5.30818800*	*-1.33004900*	*-2.32652800*
C	*-3.09422400*	*0.28756800*	*-2.33074800*

H	-2.87594900	1.35911700	-2.28561300
H	-2.13698700	-0.24486300	-2.32250400
H	-3.60186100	0.07497100	-3.27540700
C	-3.87631900	0.19619200	2.65708800
H	-2.85837500	-0.14620600	2.87756000
H	-3.88641800	1.28735600	2.75615300
H	-4.54691300	-0.22388400	3.41096800
C	-0.64985000	-0.46781400	0.75129200
O	-1.25707300	-1.40722100	-0.04246300
H	-0.57092200	-2.11283900	-0.26155800
C	0.44677400	-0.69514700	1.57950500
H	0.66036900	0.09988900	2.29182600
C	2.51574200	-1.89596800	2.42885000
C	3.34157500	-2.89339600	1.85212400
C	4.57626600	-3.22873200	2.38802700
C	4.99185100	-2.55691400	3.54529700
C	4.18455100	-1.58106500	4.13853400
C	2.94330600	-1.24676900	3.58715400
C	1.34624900	-1.75877800	1.57854800
C	1.49136800	-2.73566900	0.51336600
H	5.20767100	-3.97960200	1.92449400
H	5.95407400	-2.80199900	3.98539400
H	4.52395800	-1.07814700	5.03973200
H	2.31964400	-0.49131600	4.05953300
O	0.77138900	-2.94869100	-0.49237300
N	2.69071200	-3.40890900	0.72374500
C	3.34519000	-4.13365800	-0.35889000
H	2.54343500	-4.56889300	-0.95983600
H	1.14792400	2.35970200	1.96586300
C	1.83102300	1.63134000	0.09114300
H	2.79929400	2.08308500	0.32707300
C	1.73141200	1.49861600	-1.41505400
C	2.65897800	2.16665000	-2.22804800
C	0.81093300	0.64218000	-2.03356300
C	2.64250000	2.02769800	-3.61511600
H	3.41122700	2.80070400	-1.76389400
C	0.79460100	0.49579200	-3.42138500
H	0.12785500	0.05044700	-1.43933400
C	1.70059100	1.19400800	-4.21979400
H	3.37126900	2.56030700	-4.21981900
H	0.07576000	-0.18026000	-3.87559500
H	1.68531400	1.07615300	-5.29943900
H	1.87521600	0.63044200	0.51690100
Br	-7.22536400	-2.61525200	-0.47901500

C	*4.21312600*	*-3.21077500*	*-1.19382700*
C	*3.61825800*	*-2.17790000*	*-1.93398000*
C	*5.60401800*	*-3.33365100*	*-1.19668400*
C	*4.40534000*	*-1.28275800*	*-2.65428400*
H	*2.53766900*	*-2.07858100*	*-1.93773600*
C	*6.39513200*	*-2.44142500*	*-1.92410900*
H	*6.07354400*	*-4.13008500*	*-0.62374600*
C	*5.79702800*	*-1.41263800*	*-2.65061500*
H	*3.92918100*	*-0.48550700*	*-3.21682500*
H	*7.47658100*	*-2.54793300*	*-1.91603300*
H	*6.41166300*	*-0.71452100*	*-3.21281700*
H	*3.93651400*	*-4.94948900*	*0.06760400*

BnBr

C	*3.21945100*	*0.00004900*	*-0.48572500*
C	*2.57127500*	*-1.20824000*	*-0.22244300*
C	*1.28097300*	*-1.20736100*	*0.30316100*
C	*0.62612300*	*-0.00004000*	*0.57517700*
C	*1.28092000*	*1.20732100*	*0.30324000*
C	*2.57122800*	*1.20828800*	*-0.22236700*
H	*4.22577900*	*0.00007600*	*-0.89456000*
H	*3.07138700*	*-2.15065400*	*-0.42629000*
H	*0.77185700*	*-2.14707300*	*0.50128500*
H	*0.77177100*	*2.14700300*	*0.50141800*
H	*3.07128900*	*2.15074300*	*-0.42615100*
C	*-0.75382700*	*-0.00009000*	*1.13842100*
H	*-0.97658300*	*-0.89336800*	*1.71939900*
H	*-0.97659900*	*0.89309700*	*1.71953500*
Br	*-2.13530700*	*0.00001800*	*-0.31518300*

S-TS1

C	*5.76832100*	*-0.21041200*	*-1.05008800*
C	*4.22808900*	*0.01373700*	*-1.21237500*
C	*4.13129800*	*-2.23373500*	*-0.06057500*
C	*5.56587400*	*-2.50801600*	*-0.38439200*
H	*5.64891200*	*-3.50763200*	*-0.81927900*
N	*3.50777000*	*-1.09486000*	*-0.52163700*
O	*6.02009800*	*-1.59913100*	*-1.36326800*
C	*6.48348100*	*0.61612500*	*-2.15251000*
C	*7.71799000*	*-0.04061000*	*-2.78043500*
H	*6.75407700*	*1.58797100*	*-1.72406000*
H	*5.77236900*	*0.82714000*	*-2.96004500*
H	*8.14331000*	*0.63469900*	*-3.53034900*
H	*8.49475800*	*-0.26149600*	*-2.04572000*

H	*7.45119200*	*-0.97739000*	*-3.27381800*
H	*6.17803400*	*-2.48634500*	*0.52653900*
C	*6.29780700*	*0.13069000*	*0.35884200*
C	*7.77143800*	*-0.21716700*	*0.59519500*
H	*5.67370500*	*-0.35045000*	*1.12120900*
H	*6.15945700*	*1.19809700*	*0.52439900*
H	*8.01608100*	*-0.09269800*	*1.65489900*
H	*8.01096100*	*-1.24519900*	*0.30810400*
H	*8.43243000*	*0.44424600*	*0.02792200*
C	*2.18153400*	*-1.22525100*	*-0.22134000*
N	*2.09135600*	*-2.38466300*	*0.46710300*
N	*3.30182500*	*-3.02239600*	*0.56746700*
C	*0.86644700*	*-2.95426900*	*0.95207800*
C	*-0.01342500*	*-3.51228700*	*0.01624900*
C	*0.54385500*	*-2.74306100*	*2.30099000*
C	*-1.32476500*	*-3.77402300*	*0.44363400*
C	*-0.76087600*	*-3.03014300*	*2.69970500*
C	*-1.68289800*	*-3.47945200*	*1.75255500*
H	*-2.06858300*	*-4.07583500*	*-0.29256900*
H	*-1.07165800*	*-2.85281200*	*3.72336300*
C	*1.54886600*	*-2.15868500*	*3.26164700*
H	*2.45527100*	*-2.77117100*	*3.31119600*
H	*1.86081400*	*-1.15142900*	*2.95658900*
H	*1.12553500*	*-2.08608200*	*4.26645900*
C	*0.38028700*	*-3.78182100*	*-1.41182600*
H	*1.10969600*	*-3.05989900*	*-1.78910700*
H	*0.82954100*	*-4.77983600*	*-1.49269000*
H	*-0.50847600*	*-3.74661000*	*-2.05336700*
C	*1.08219200*	*-0.39670600*	*-0.67773500*
O	*1.26029300*	*0.03144900*	*-1.95769000*
H	*0.59828700*	*0.77582400*	*-2.14208000*
C	*0.01905000*	*-0.15051400*	*0.13416600*
H	*0.04569200*	*-0.60141000*	*1.11805200*
C	*-2.16869500*	*0.91777400*	*0.88006700*
C	*-2.80397800*	*2.11631700*	*0.50009900*
C	*-3.85630800*	*2.65964300*	*1.21967700*
C	*-4.28456100*	*1.96368400*	*2.35745100*
C	*-3.67437300*	*0.76816000*	*2.74485800*
C	*-2.61414800*	*0.23223800*	*2.00396100*
C	*-1.18974300*	*0.58203800*	*-0.16816700*
C	*-1.19499700*	*1.74487300*	*-1.08307000*
H	*-4.32899600*	*3.58727900*	*0.91774300*
H	*-5.10994500*	*2.36075100*	*2.94087200*
H	*-4.03618100*	*0.23536000*	*3.61913800*

H	-2.17949000	-0.72210300	2.28265100
O	-0.46357700	1.96338000	-2.07407100
N	-2.20377800	2.58321200	-0.68271300
C	-2.60262900	3.77317600	-1.41182800
H	-2.10035300	3.71004400	-2.38131100
H	-3.68288900	3.72479500	-1.58595500
C	-2.22442000	5.04834800	-0.68898900
C	-0.90461900	5.24562500	-0.26600000
C	-3.17569000	6.03874100	-0.43733300
C	-0.54587900	6.41521800	0.39938700
H	-0.16298000	4.47593900	-0.46220100
C	-2.81779900	7.21420400	0.22619100
H	-4.20327800	5.88883600	-0.76093000
C	-1.50240800	7.40346300	0.64751400
H	0.48082700	6.55767200	0.72525200
H	-3.56791400	7.97620400	0.41842600
H	-1.22234300	8.31476700	1.16831900
H	4.00117000	-0.13221500	-2.27121300
C	3.71442900	1.43366400	-0.86242700
H	4.27916400	2.09733300	-1.52545600
C	3.81269300	1.97144000	0.55040500
C	4.61328000	3.09372000	0.80477100
C	3.08892700	1.42606600	1.61851300
C	4.71692100	3.63486300	2.08558800
H	5.16697200	3.54650900	-0.01479600
C	3.19259900	1.95957900	2.90276800
H	2.40748400	0.60341400	1.44509000
C	4.01255500	3.06169200	3.14453500
H	5.34566000	4.50448100	2.25426600
H	2.61833600	1.51917700	3.71325700
H	4.08991600	3.47866700	4.14425600
H	2.68273200	1.49018800	-1.21068500
Br	-3.51945100	-3.58624800	2.25431600
C	-5.81966000	-0.04751400	-0.55394600
C	-4.56407900	-0.64071200	-0.52862200
C	-3.58013400	-0.26086000	-1.45803000
C	-3.89586900	0.70132400	-2.43038100
C	-5.15774000	1.28914500	-2.45879900
C	-6.11914900	0.92459400	-1.51295400
H	-6.57016600	-0.34404900	0.17289500
H	-4.33139000	-1.41051300	0.19953400
H	-3.14126700	0.98392300	-3.15939100
H	-5.39515900	2.02466200	-3.22292000
H	-7.10315200	1.38514700	-1.53224600

C	*-2.23025100*	*-0.79481900*	*-1.34496500*
H	*-1.56585600*	*-0.76079500*	*-2.19838300*
H	*-2.06480800*	*-1.60547900*	*-0.65651800*
Br	*-2.96641400*	*-3.26994700*	*-2.61863000*

R-TS1

C	*-5.53405800*	*-1.46656200*	*-0.94863800*
C	*-3.97861800*	*-1.28422700*	*-1.06948600*
C	*-4.51952500*	*1.16318100*	*-0.73409100*
C	*-5.98434800*	*0.89692500*	*-0.90036400*
H	*-6.42025700*	*1.67503500*	*-1.53232300*
N	*-3.61410600*	*0.15118000*	*-0.93725800*
O	*-6.16254900*	*-0.32710500*	*-1.57946100*
C	*-5.92068100*	*-2.70729900*	*-1.80723600*
C	*-7.21117300*	*-2.57293400*	*-2.62392100*
H	*-5.99300100*	*-3.57263200*	*-1.13884200*
H	*-5.10713000*	*-2.93511000*	*-2.50410000*
H	*-7.39068400*	*-3.50642300*	*-3.16777500*
H	*-8.08387300*	*-2.37174500*	*-1.99943700*
H	*-7.12966600*	*-1.76234000*	*-3.35131500*
H	*-6.50306100*	*0.92321600*	*0.06688000*
C	*-6.04366200*	*-1.62914300*	*0.49864700*
C	*-7.55613900*	*-1.83185700*	*0.62399800*
H	*-5.76025700*	*-0.76364300*	*1.09970400*
H	*-5.52008400*	*-2.47315300*	*0.95594800*
H	*-7.84500500*	*-1.82185900*	*1.67976000*
H	*-8.11181200*	*-1.04008100*	*0.11150400*
H	*-7.87692000*	*-2.78982000*	*0.20535700*
C	*-2.36743400*	*0.70737600*	*-0.89654700*
N	*-2.58502200*	*2.01405200*	*-0.60145900*
N	*-3.92953200*	*2.30475900*	*-0.50327100*
C	*-1.58383400*	*3.01928700*	*-0.39976800*
C	*-0.90184800*	*3.52265400*	*-1.51604800*
C	*-1.25542900*	*3.35679300*	*0.92102500*
C	*0.20560600*	*4.34070900*	*-1.28045900*
C	*-0.14307000*	*4.17678000*	*1.12221300*
C	*0.58695800*	*4.62914800*	*0.02686500*
H	*0.78642400*	*4.72216400*	*-2.11219600*
H	*0.17228300*	*4.42636800*	*2.12828200*
C	*-2.05192300*	*2.82288300*	*2.08031200*
H	*-3.10681200*	*3.10237100*	*1.99045700*
H	*-2.00743700*	*1.73030700*	*2.12541800*
H	*-1.66803300*	*3.21576700*	*3.02406800*
C	*-1.29953500*	*3.14419300*	*-2.91836100*

H	-0.95677000	2.13142000	-3.16056100
H	-2.38658800	3.16632900	-3.04432100
H	-0.85513600	3.83116700	-3.64272000
C	-1.13618400	0.06540400	-1.29956700
O	-1.36132400	-0.92981000	-2.19729500
H	-0.51545200	-1.48246300	-2.31056000
C	0.07575600	0.50239200	-0.85171100
H	0.06729800	1.31400600	-0.13769600
C	2.60472900	0.75891000	-1.04750500
C	3.59421200	0.07193400	-1.78118400
C	4.90030900	0.53249300	-1.86313900
C	5.20400200	1.73705000	-1.21267500
C	4.22960900	2.44073900	-0.50127600
C	2.92311200	1.94783000	-0.40345400
C	1.37997200	-0.05462000	-1.09165400
C	1.67814100	-1.14838800	-2.04986000
H	5.66168400	-0.01934700	-2.40198400
H	6.21742700	2.12395000	-1.26549200
H	4.47700900	3.37828100	-0.01382200
H	2.18140500	2.48167200	0.17841400
O	0.92379700	-2.06174500	-2.44260000
N	3.01103700	-1.06474400	-2.37244800
C	3.73935800	-2.14844300	-3.02913700
H	2.97382300	-2.78153200	-3.48438600
H	4.35465100	-1.72328000	-3.82928500
C	4.59051500	-2.92014600	-2.04251100
C	3.98514800	-3.57618600	-0.96352700
C	5.98277700	-2.93814100	-2.15817600
C	4.76096900	-4.20978400	0.00503200
H	2.90468900	-3.59768400	-0.85106000
C	6.76249400	-3.59570500	-1.20450400
H	6.46024700	-2.43320100	-2.99579600
C	6.15256400	-4.22305400	-0.11749700
H	4.25421000	-4.66131700	0.85255200
H	7.84467300	-3.60386500	-1.30482000
H	6.76042200	-4.71764800	0.63529100
H	-3.71484600	-1.52903600	-2.09752100
C	-3.13388700	-2.21155500	-0.16566500
H	-3.69333200	-3.15350700	-0.10013100
C	-2.70868400	-1.80495400	1.23931500
C	-1.69359600	-2.57773900	1.82636600
C	-3.25623700	-0.76347700	2.00056700
C	-1.28073800	-2.36153700	3.13936100
H	-1.17769700	-3.34927100	1.25912500

C	*-2.85288900*	*-0.54909400*	*3.32259400*
H	*-4.00300700*	*-0.10090900*	*1.58328500*
C	*-1.87768900*	*-1.35737200*	*3.90278900*
H	*-0.45237600*	*-2.95408400*	*3.51464900*
H	*-3.30716000*	*0.25837400*	*3.89143500*
H	*-1.55766400*	*-1.18268600*	*4.92572900*
H	*-2.22918300*	*-2.45704700*	*-0.72254400*
Br	*2.18335000*	*5.62155800*	*0.33078000*
C	*3.23754900*	*0.92898500*	*3.10212600*
C	*3.04772900*	*-0.00755200*	*2.09061700*
C	*1.75231300*	*-0.37452500*	*1.68781900*
C	*0.65184300*	*0.22866600*	*2.31934100*
C	*0.84293000*	*1.16143700*	*3.33386500*
C	*2.13599500*	*1.51831600*	*3.72627900*
H	*4.24510000*	*1.20088200*	*3.40287000*
H	*3.90019100*	*-0.46883200*	*1.60190000*
H	*-0.34874400*	*-0.05847800*	*2.02109000*
H	*-0.01799200*	*1.59488400*	*3.83396000*
H	*2.28391700*	*2.24504600*	*4.52055100*
C	*1.57337300*	*-1.28820000*	*0.56873400*
H	*0.62741300*	*-1.78598600*	*0.42546100*
H	*2.44322200*	*-1.83198100*	*0.23310400*
Br	*1.64256900*	*-3.85896500*	*1.88631600*

S-IM2

C	*-5.35575600*	*-1.16657100*	*1.37176900*
C	*-3.89231600*	*-0.60805500*	*1.45981400*
C	*-3.36948200*	*-2.81057300*	*0.33495700*
C	*-4.71148200*	*-3.36560000*	*0.69062000*
H	*-4.57639900*	*-4.36442500*	*1.11393000*
N	*-2.95970400*	*-1.58367400*	*0.81015500*
O	*-5.29644800*	*-2.57094400*	*1.69835800*
C	*-6.18555700*	*-0.49286600*	*2.50066600*
C	*-7.18988200*	*-1.40347100*	*3.21607000*
H	*-6.70763900*	*0.36943800*	*2.07052100*
H	*-5.50395300*	*-0.08742700*	*3.25746500*
H	*-7.72816600*	*-0.82208400*	*3.97198000*
H	*-7.92495500*	*-1.83304800*	*2.53242000*
H	*-6.67990000*	*-2.22907600*	*3.71653400*
H	*-5.34781300*	*-3.46080300*	*-0.19784900*
C	*-6.01270500*	*-0.96660100*	*-0.00894600*
C	*-7.38751800*	*-1.62458600*	*-0.16869700*
H	*-5.33788100*	*-1.30570400*	*-0.80384700*
H	*-6.12104300*	*0.10167300*	*-0.17902800*

H	*-7.71337800*	*-1.55340300*	*-1.21125200*
H	*-7.38419500*	*-2.68060200*	*0.11601500*
H	*-8.14147300*	*-1.12304400*	*0.44477100*
C	*-1.62285700*	*-1.49589900*	*0.54256700*
N	*-1.32674600*	*-2.60363000*	*-0.16651100*
N	*-2.40768700*	*-3.43407300*	*-0.28915100*
C	*-0.08818400*	*-2.89479600*	*-0.84100300*
C	*0.97179400*	*-3.44922100*	*-0.12111800*
C	*0.00580000*	*-2.48191000*	*-2.18421500*
C	*2.21200300*	*-3.54267800*	*-0.77393300*
C	*1.25792100*	*-2.55739100*	*-2.78823200*
C	*2.34405600*	*-3.04996600*	*-2.06308700*
H	*3.08119800*	*-3.86741900*	*-0.21329000*
H	*1.39262300*	*-2.21889400*	*-3.80939500*
C	*-1.19574000*	*-1.98260200*	*-2.94506100*
H	*-1.90677000*	*-2.79651100*	*-3.13087500*
H	*-1.74167400*	*-1.20252800*	*-2.40626800*
H	*-0.89352800*	*-1.57150700*	*-3.91177200*
C	*0.87196100*	*-3.87452900*	*1.31702000*
H	*-0.02915500*	*-3.49884000*	*1.80879800*
H	*0.86445400*	*-4.96857000*	*1.39157500*
H	*1.76620100*	*-3.50715000*	*1.83935000*
C	*-0.69274000*	*-0.50206500*	*1.07774000*
O	*-1.14187200*	*0.03423800*	*2.24529900*
H	*-0.75241800*	*0.95671100*	*2.32227900*
C	*0.47296100*	*-0.20685200*	*0.46579900*
H	*0.70425300*	*-0.69368800*	*-0.46973700*
C	*2.52947800*	*1.06361200*	*-0.11030000*
C	*2.44969000*	*2.41113800*	*-0.47728100*
C	*3.25489200*	*2.95284000*	*-1.46757800*
C	*4.15857400*	*2.08644900*	*-2.10056500*
C	*4.24913800*	*0.74182100*	*-1.73802500*
C	*3.43404800*	*0.21336300*	*-0.72669000*
C	*1.49536100*	*0.78841000*	*0.96076000*
C	*0.82296200*	*2.16364200*	*1.11073600*
H	*3.19014200*	*3.99682600*	*-1.75342900*
H	*4.80025800*	*2.47840700*	*-2.88463900*
H	*4.95226500*	*0.08506000*	*-2.23971300*
H	*3.56481300*	*-0.80846800*	*-0.37310900*
O	*-0.13524800*	*2.42324300*	*1.86199900*
N	*1.44766800*	*3.04882100*	*0.30194900*
C	*1.08783200*	*4.46387700*	*0.24182500*
H	*0.73619500*	*4.73307700*	*1.24069700*
H	*2.00221000*	*5.02751000*	*0.03993000*

C	*0.02319600*	*4.74302600*	*-0.79608500*
C	*-1.31140900*	*4.41649400*	*-0.52221000*
C	*0.34947500*	*5.29283600*	*-2.03819400*
C	*-2.29982200*	*4.63482600*	*-1.47764500*
H	*-1.56168800*	*3.98181400*	*0.44048900*
C	*-0.64050100*	*5.51022000*	*-2.99839400*
H	*1.38121400*	*5.55654300*	*-2.25642400*
C	*-1.96613500*	*5.17900200*	*-2.71956300*
H	*-3.32815100*	*4.36830400*	*-1.25867100*
H	*-0.37449500*	*5.93819100*	*-3.96088800*
H	*-2.73836800*	*5.34308500*	*-3.46594800*
H	*-3.61144200*	*-0.62728400*	*2.51367500*
C	*-3.72298600*	*0.87179600*	*1.00332800*
H	*-4.49430800*	*1.41530100*	*1.55969400*
C	*-3.80358300*	*1.26762800*	*-0.45801500*
C	*-4.91509800*	*1.96972700*	*-0.94631400*
C	*-2.74000400*	*1.03593900*	*-1.33937000*
C	*-4.99936700*	*2.35549600*	*-2.28372300*
H	*-5.72611300*	*2.22293700*	*-0.26731700*
C	*-2.81545400*	*1.42316300*	*-2.67739400*
H	*-1.82185500*	*0.59408600*	*-0.97137900*
C	*-3.95295300*	*2.06750700*	*-3.16042000*
H	*-5.87692200*	*2.89043700*	*-2.63550400*
H	*-1.97076400*	*1.24080600*	*-3.33456300*
H	*-4.00997500*	*2.37116600*	*-4.20130700*
H	*-2.77153100*	*1.21510100*	*1.40675200*
Br	*4.07757100*	*-2.98839400*	*-2.85216300*
C	*2.12012400*	*0.38090100*	*2.34544700*
H	*1.30672800*	*0.31130600*	*3.07435200*
H	*2.56574100*	*-0.61244400*	*2.21774400*
C	*3.16846300*	*1.37275300*	*2.79060800*
C	*2.81094100*	*2.57419000*	*3.41825300*
C	*4.52178600*	*1.11593100*	*2.53240200*
C	*3.78268400*	*3.51421500*	*3.76153700*
H	*1.76552300*	*2.77313200*	*3.63948900*
C	*5.49173000*	*2.05578800*	*2.88015100*
H	*4.79874200*	*0.16174900*	*2.09007100*
C	*5.12716100*	*3.25930400*	*3.48628300*
H	*3.49080900*	*4.44020700*	*4.25086800*
H	*6.53819900*	*1.84256200*	*2.67920400*
H	*5.88660200*	*3.98940000*	*3.75430900*
Br	*4.16376000*	*-2.45030000*	*1.59345400*

R-IM2

C	5.71741200	-1.08474700	-1.10195900
C	4.35065300	-0.33304600	-1.16939300
C	3.42286000	-2.66240100	-0.85905100
C	4.72523300	-3.27207200	-1.27481100
H	4.53808700	-4.04134400	-2.02861900
N	3.26382900	-1.29512900	-0.81141300
O	5.54889400	-2.29841100	-1.87582400
C	6.75731100	-0.22915800	-1.86961500
C	7.81872100	-1.00983300	-2.65294500
H	7.23767800	0.44085700	-1.14761300
H	6.22973900	0.41760800	-2.58103600
H	8.49277700	-0.30261700	-3.14732700
H	8.42124100	-1.65829900	-2.01425900
H	7.35498600	-1.63428000	-3.41931200
H	5.20591200	-3.75585500	-0.41393000
C	6.17240500	-1.41125400	0.33571700
C	7.42826200	-2.28352700	0.43735500
H	5.35057600	-1.88105800	0.88920500
H	6.35151800	-0.46995800	0.85475100
H	7.57064200	-2.60691400	1.47333700
H	7.37155700	-3.17533700	-0.19399000
H	8.32363300	-1.72899500	0.14371100
C	1.96493000	-1.06383300	-0.49997500
N	1.40879600	-2.27759600	-0.34039100
N	2.31393800	-3.28700700	-0.55861100
C	0.00961600	-2.57550600	-0.14475200
C	-0.85603000	-2.42503400	-1.23633100
C	-0.41541400	-2.97683900	1.12856000
C	-2.20723300	-2.70799800	-1.02384100
C	-1.77387100	-3.24210800	1.30527000
C	-2.65062600	-3.11076900	0.23092300
H	-2.91160200	-2.60546000	-1.84030900
H	-2.14905700	-3.53451500	2.27831800
C	0.55625200	-3.10200900	2.27351600
H	0.04060200	-3.41781100	3.18300300
H	1.34358900	-3.83019600	2.05063000
H	1.04963600	-2.14532200	2.48842100
C	-0.38605900	-1.94018900	-2.58314300
H	-0.33605500	-0.84611100	-2.60944100
H	0.60456400	-2.33051300	-2.83713500
H	-1.08280400	-2.25640100	-3.36345500
C	1.18862000	0.17995700	-0.49158900
O	1.35369900	0.87533200	-1.63858500
H	0.51245500	1.43013300	-1.77644000

C	0.33314500	0.36280300	0.53173000
H	0.41818900	-0.34158500	1.34984800
C	-1.88257600	0.68265600	1.52829500
C	-3.02303200	0.37125300	0.76988900
C	-4.10213900	-0.27787900	1.36451600
C	-4.00921300	-0.60976100	2.72121000
C	-2.87824600	-0.30495700	3.47672100
C	-1.80083300	0.35338600	2.86971900
C	-0.83400400	1.31748200	0.63928600
C	-1.56661700	1.30989700	-0.72188000
H	-4.98556300	-0.54563000	0.81003300
H	-4.84674600	-1.12325600	3.18442000
H	-2.83499900	-0.56645300	4.52987700
H	-0.91098700	0.60348200	3.44307000
O	-1.06964600	1.59997500	-1.82094500
N	-2.78956100	0.72435800	-0.57718300
C	-3.70793700	0.79609500	-1.73116300
H	-3.64725900	1.83844500	-2.06378200
H	-3.30180800	0.14725500	-2.51518100
C	-5.13109200	0.42808400	-1.41637800
C	-5.95596600	1.34637100	-0.75174200
C	-5.63208200	-0.83251200	-1.75168500
C	-7.25687900	0.98384300	-0.40824000
H	-5.54285300	2.31863000	-0.48520800
C	-6.93342000	-1.19580000	-1.40295100
H	-4.99595400	-1.54080300	-2.27699500
C	-7.74611800	-0.28735600	-0.72374500
H	-7.89027000	1.69482200	0.11506100
H	-7.30719900	-2.18419700	-1.65656600
H	-8.75924500	-0.56641000	-0.44611100
H	4.17334400	-0.12017900	-2.22746500
C	4.25883100	1.01860500	-0.43716300
H	4.98938800	1.65700500	-0.94302400
C	4.51116600	1.09381700	1.05283300
C	5.54332500	1.91497300	1.52524300
C	3.72773800	0.41512500	1.99587900
C	5.79675600	2.04815400	2.89044700
H	6.15545600	2.45954900	0.81036200
C	3.98118000	0.53804000	3.36099400
H	2.89768000	-0.20031000	1.67370100
C	5.01788300	1.35394300	3.81543100
H	6.60006800	2.69599900	3.22882900
H	3.35806400	0.00307200	4.07231600
H	5.20956300	1.45455100	4.87941300

H	*3.29410400*	*1.47074300*	*-0.67665500*
Br	*-4.49219800*	*-3.51008900*	*0.47079000*
C	*-0.43907300*	*2.73895000*	*1.15902200*
H	*-1.37268700*	*3.31263800*	*1.18338500*
H	*-0.07503300*	*2.59723200*	*2.18365700*
C	*0.60513700*	*3.45688700*	*0.34404700*
C	*0.24172200*	*4.17322100*	*-0.80733300*
C	*1.94940200*	*3.43300700*	*0.73364600*
C	*1.21884900*	*4.82101200*	*-1.56233800*
H	*-0.81083100*	*4.23392700*	*-1.07590500*
C	*2.92313500*	*4.09536800*	*-0.01583300*
H	*2.23523000*	*2.90197500*	*1.63838600*
C	*2.56074800*	*4.78085300*	*-1.17571200*
H	*0.92609600*	*5.37250500*	*-2.45178900*
H	*3.95882100*	*4.08006500*	*0.31429000*
H	*3.31531300*	*5.29780800*	*-1.76357200*
Br	*-3.37549200*	*3.91188300*	*-0.30470300*

References:

[1] Z. Zhang, J. C. Antilla. *Angew. Chem. Int. Ed.* **2012**, 51, 11778–11782.
[2] T. Kawasaki, M. Shinada, D. Kamimura, M. Ohzonoa, A. Ogawa. *Chem. Commun.* **2006**, 420–422.
[3] Gaussian 16 Rev. A.03, M. J. Frisch, et al. Gaussian Inc. Wallingford CT, 2016.61
[4] a) A. Bergner, M. Dolg, W. Küchle, H. Stoll, H. Preuss. *Mol. Phys.* **1993**, *80*, 1431–1441. b) R. Ditchfield, W. J. Hehre, J. A. Pople. *J. Chem. Phys*. **1971**, *54*, 724–728. c) T. Clark, J. Chandrasekhar, G. W. Spitznagel, P.v.R. Schleyer. *J. Comput.. Chem*. **1983**, *4*, 294–301.
[5] CYLview, 1.0b, C. Y. Legault, Université de Sherbrooke, 2009, http://www.cylview.org/.

HPLC Traces

methyl (S)-2-(1,3-dibenzyl-2-oxoindolin-3-yl)acetate (**4a**)

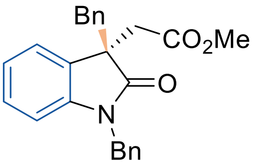

Chromatogram

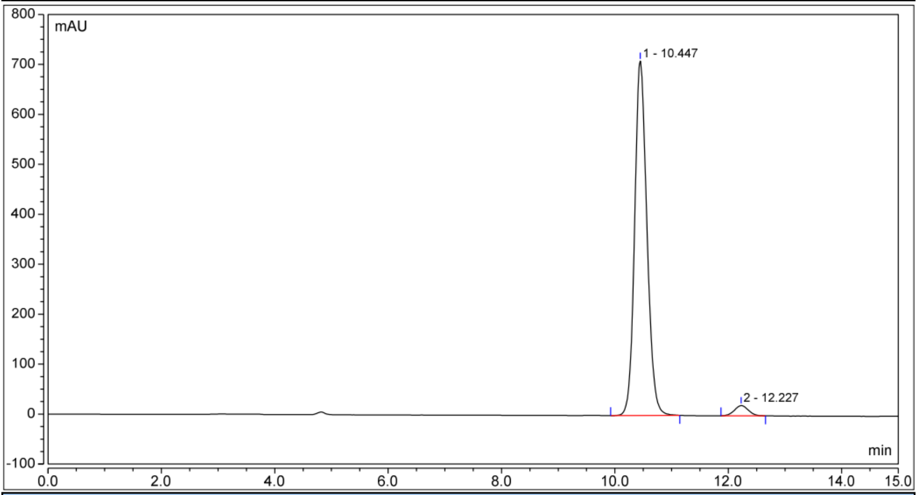

Integration Results

No.	Retention Time min	Area mAu*min	Height mAU	Relative Area %	Relative Height %
1	10.447	178.209	709.667	96.86	97.21
2	12.227	5.784	20.343	3.14	2.79
**Total:**		**183.993**	**730.010**	**100.00**	**100.00**

Chromatogram

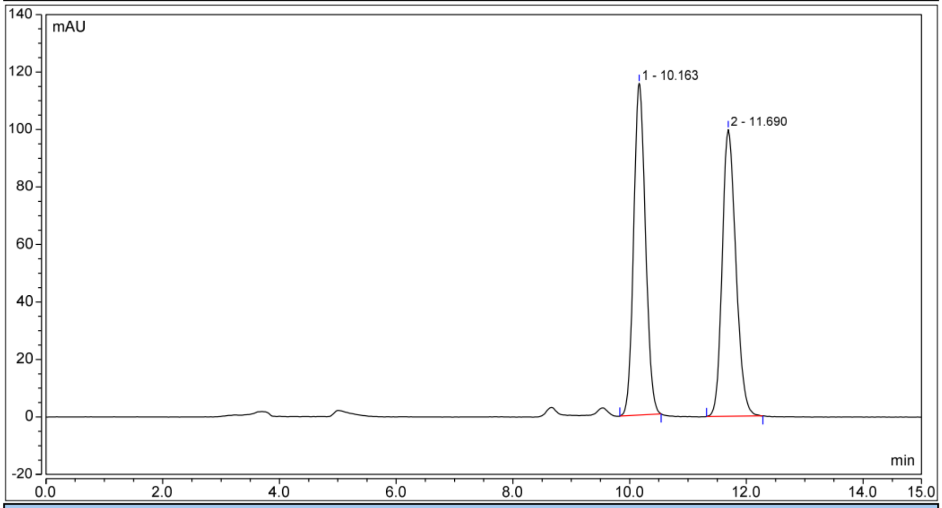

Integration Results

No.	Retention Time min	Area mAu*min	Height mAU	Relative Area %	Relative Height %
1	10.163	27.001	115.429	49.77	53.65
2	11.690	27.246	99.734	50.23	46.35
**Total:**		**54.247**	**215.164**	**100.00**	**100.00**

methyl (S)-2-(1,3-dibenzyl-5-fluoro-2-oxoindolin-3-yl)acetate (**4b**)

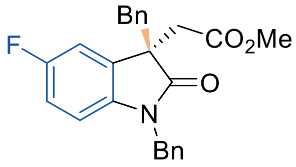

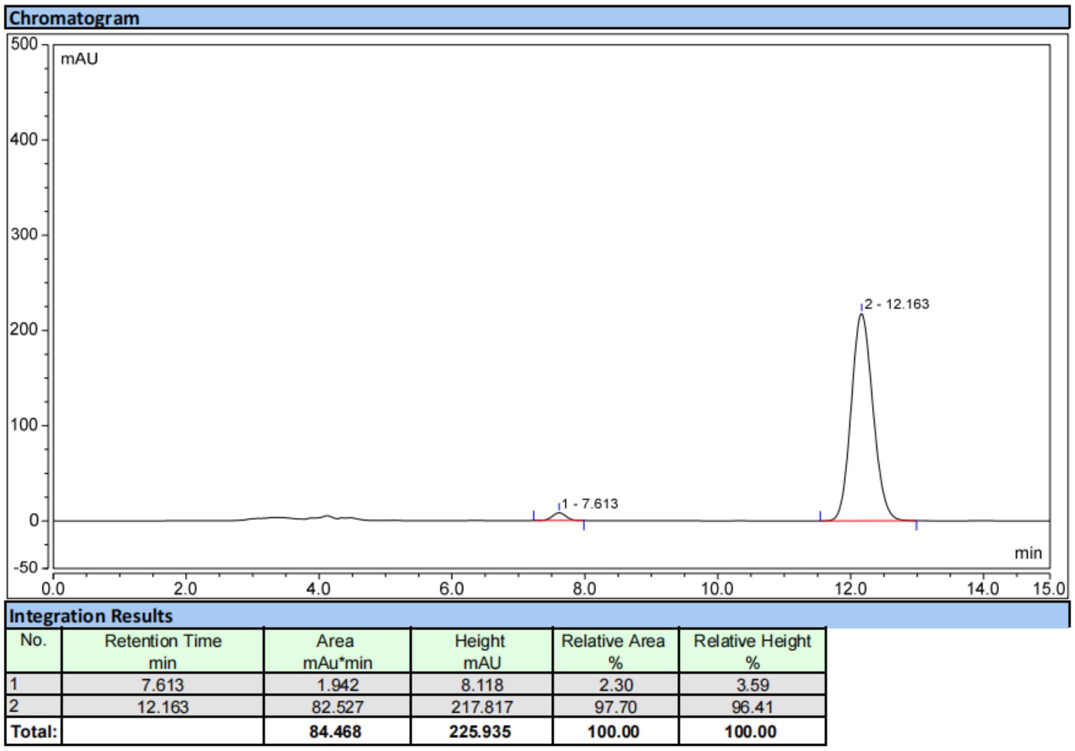

Integration Results

No.	Retention Time min	Area mAu*min	Height mAU	Relative Area %	Relative Height %
1	7.613	1.942	8.118	2.30	3.59
2	12.163	82.527	217.817	97.70	96.41
**Total:**		**84.468**	**225.935**	**100.00**	**100.00**

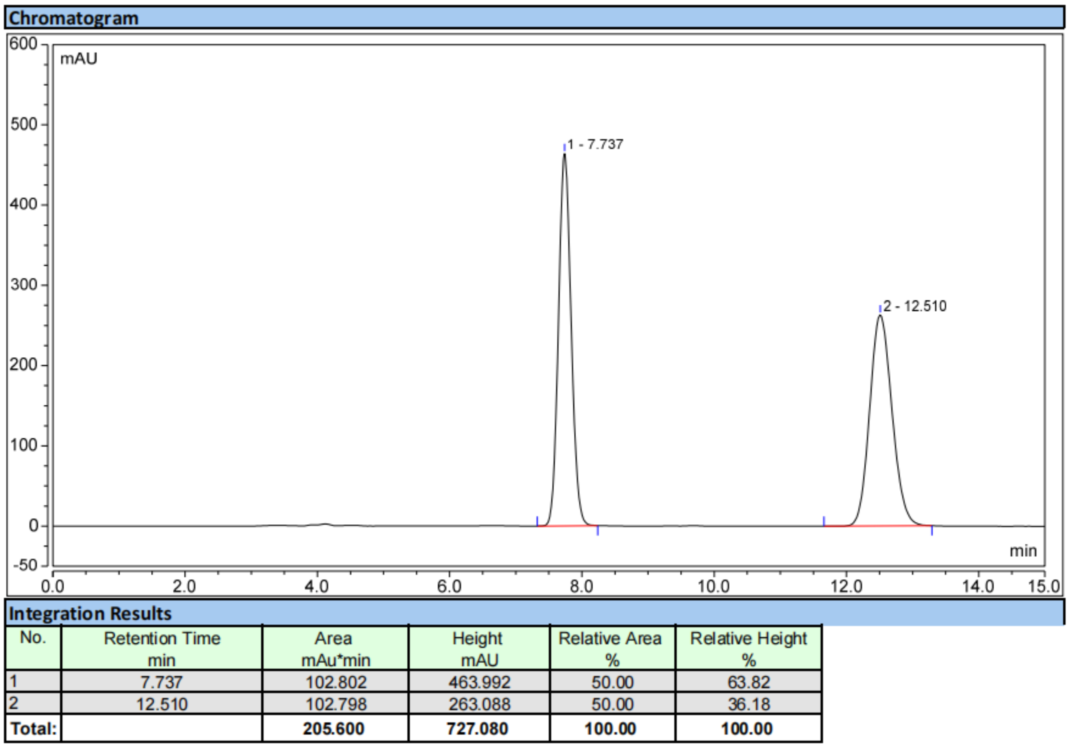

Integration Results

No.	Retention Time min	Area mAu*min	Height mAU	Relative Area %	Relative Height %
1	7.737	102.802	463.992	50.00	63.82
2	12.510	102.798	263.088	50.00	36.18
**Total:**		**205.600**	**727.080**	**100.00**	**100.00**

methyl (S)-2-(1,3-dibenzyl-5-chloro-2-oxoindolin-3-yl)acetate (**4c**)

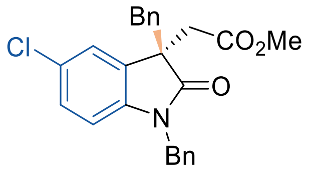

Chromatogram

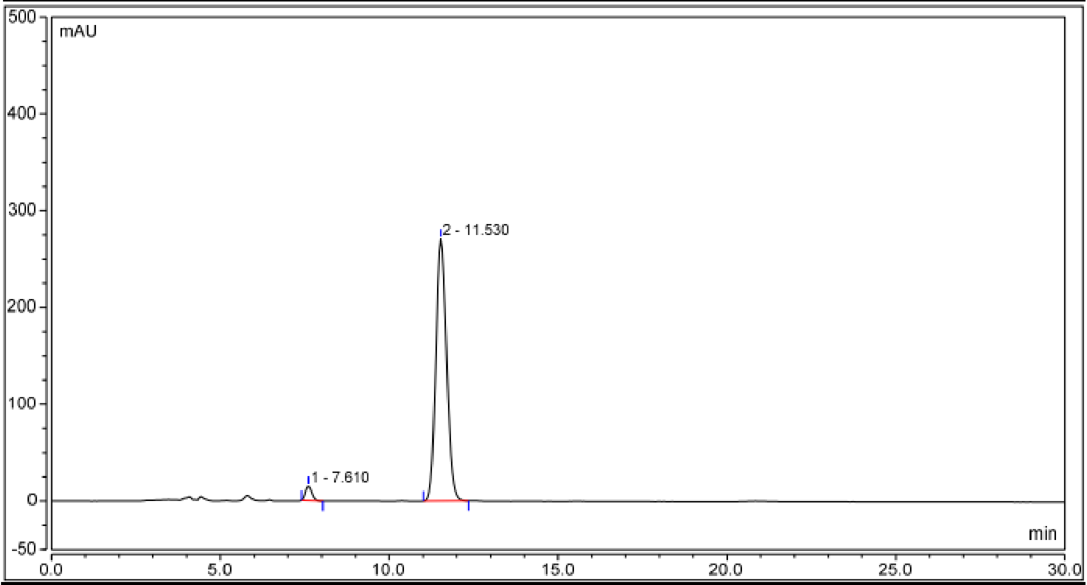

Integration Results

No.	Retention Time min	Area mAu*min	Height mAU	Relative Area %	Relative Height %
1	7.610	3.173	14.692	3.07	5.15
2	11.530	100.253	270.645	96.93	94.85
**Total:**		**103.426**	**285.337**	**100.00**	**100.00**

Chromatogram

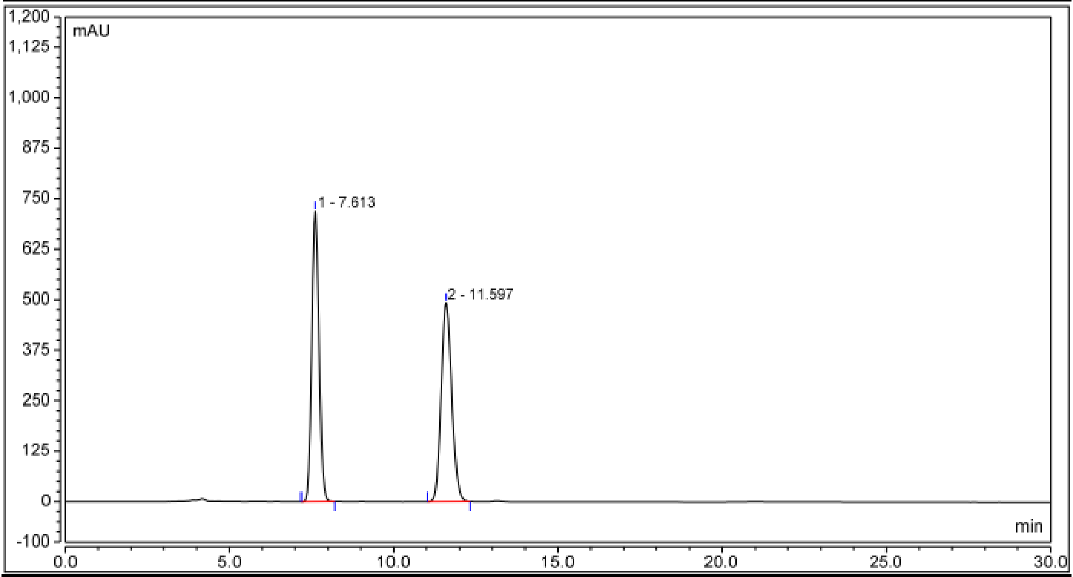

Integration Results

No.	Retention Time min	Area mAu*min	Height mAU	Relative Area %	Relative Height %
1	7.613	183.594	718.549	49.94	59.39
2	11.597	184.062	491.389	50.06	40.61
**Total:**		**367.656**	**1209.938**	**100.00**	**100.00**

methyl (S)-2-(1,3-dibenzyl-5-bromo-2-oxoindolin-3-yl)acetate (**4d**)

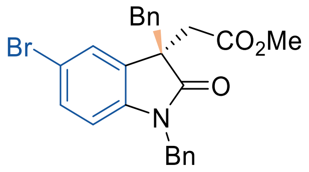

Chromatogram

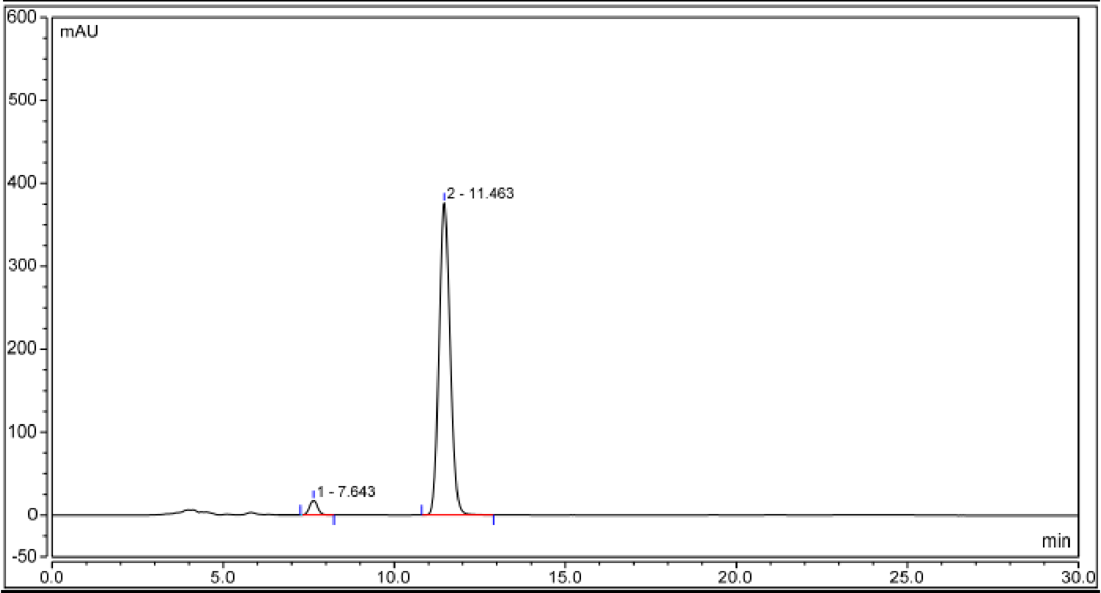

Integration Results

No.	Retention Time min	Area mAu*min	Height mAU	Relative Area %	Relative Height %
1	7.643	4.826	17.538	3.31	4.45
2	11.463	141.065	376.238	96.69	95.55
**Total:**		**145.891**	**393.776**	**100.00**	**100.00**

Chromatogram

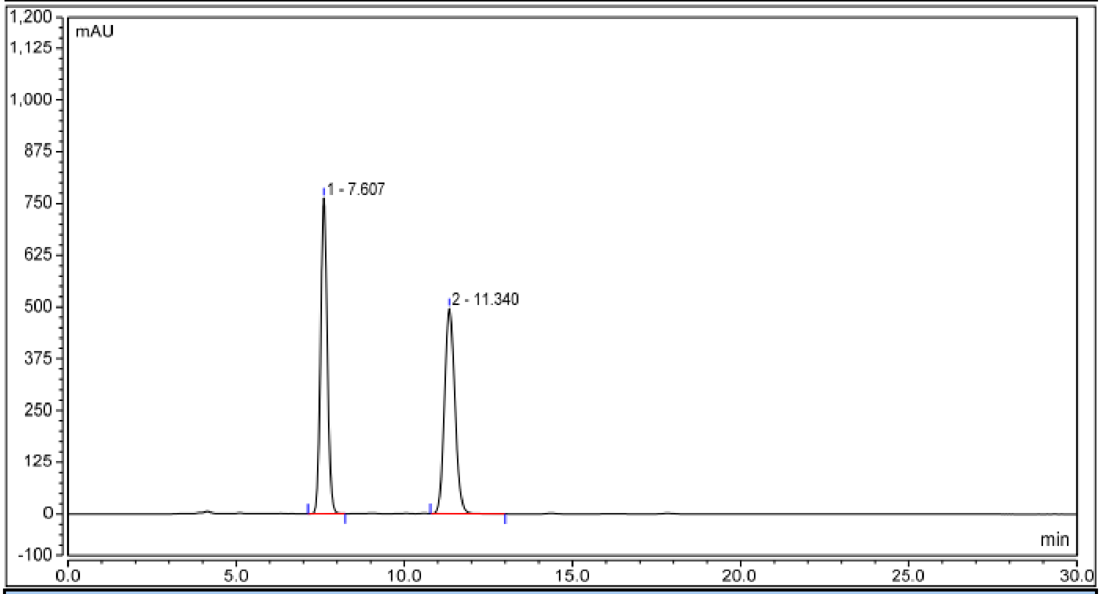

Integration Results

No.	Retention Time min	Area mAu*min	Height mAU	Relative Area %	Relative Height %
1	7.607	177.033	762.173	49.94	60.58
2	11.340	177.460	495.870	50.06	39.42
**Total:**		**354.492**	**1258.043**	**100.00**	**100.00**

methyl (S)-2-(1,3-dibenzyl-2-oxo-5-(trifluoromethoxy)indolin-3-yl)acetate (**4e**)

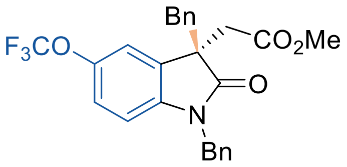

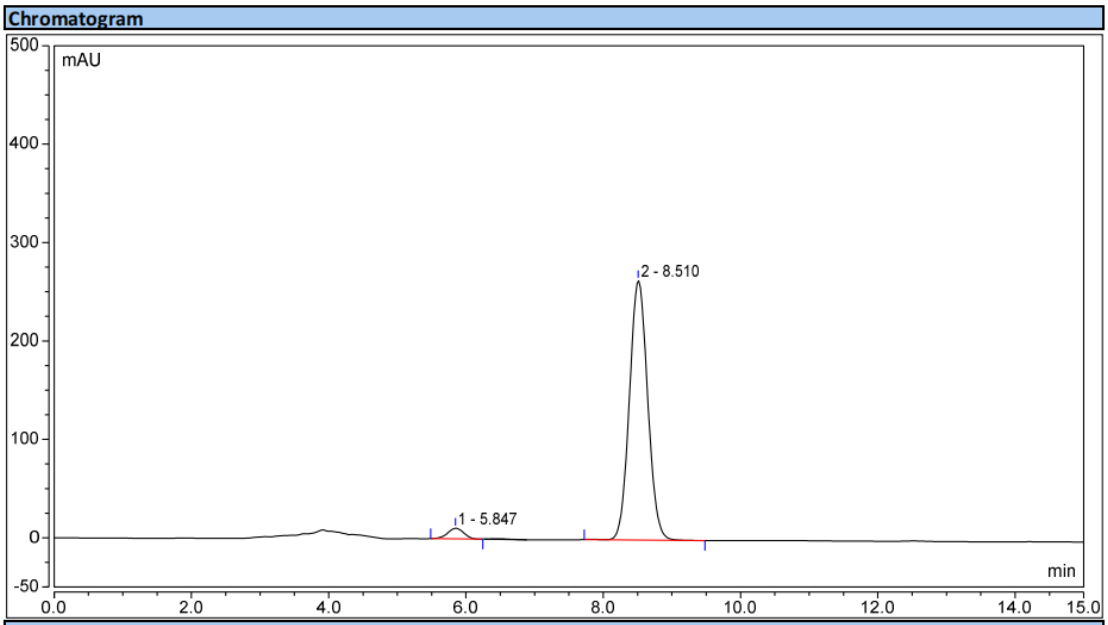

Integration Results

No.	Retention Time min	Area mAu*min	Height mAU	Relative Area %	Relative Height %
1	5.847	2.949	10.805	3.50	3.94
2	8.510	81.236	263.728	96.50	96.06
**Total:**		**84.185**	**274.534**	**100.00**	**100.00**

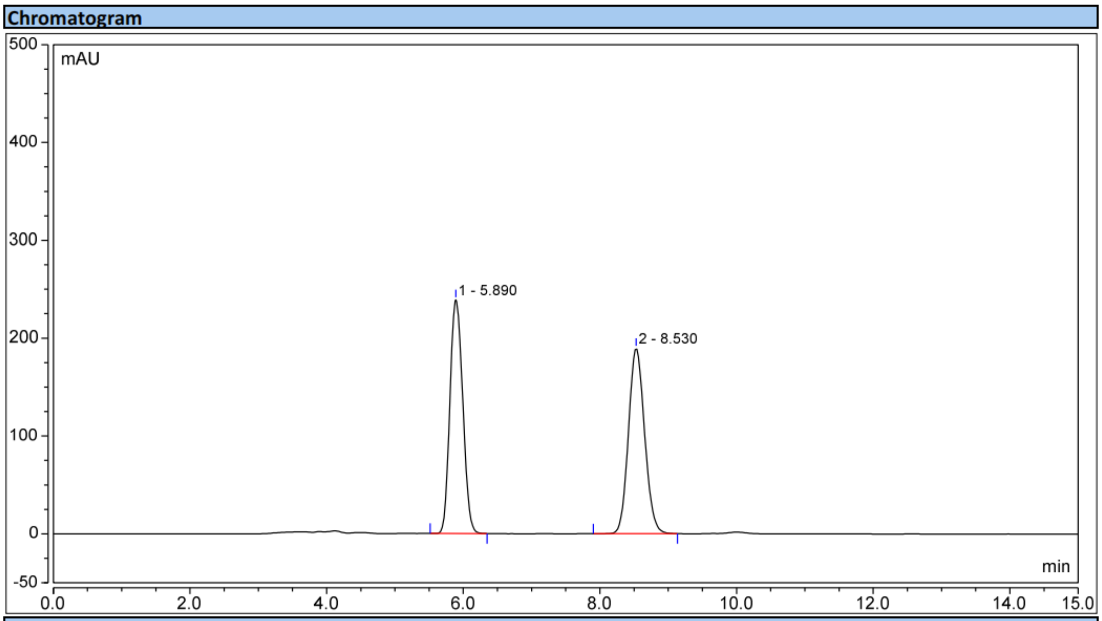

Integration Results

No.	Retention Time min	Area mAu*min	Height mAU	Relative Area %	Relative Height %
1	5.890	51.956	238.949	50.04	55.77
2	8.530	51.869	189.505	49.96	44.23
**Total:**		**103.825**	**428.453**	**100.00**	**100.00**

methyl (S)-2-(1,3-dibenzyl-5-methoxy-2-oxoindolin-3-yl)acetate (**4f**)

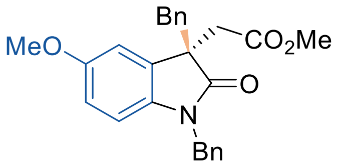

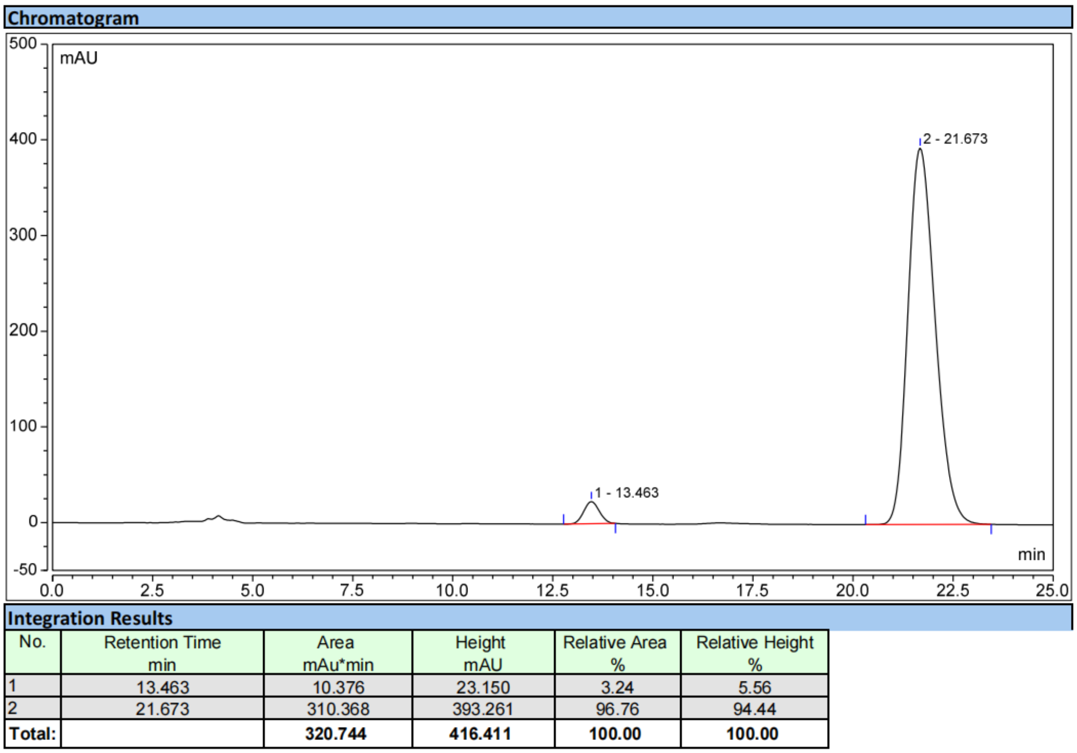

Integration Results

No.	Retention Time min	Area mAu*min	Height mAU	Relative Area %	Relative Height %
1	13.463	10.376	23.150	3.24	5.56
2	21.673	310.368	393.261	96.76	94.44
**Total:**		**320.744**	**416.411**	**100.00**	**100.00**

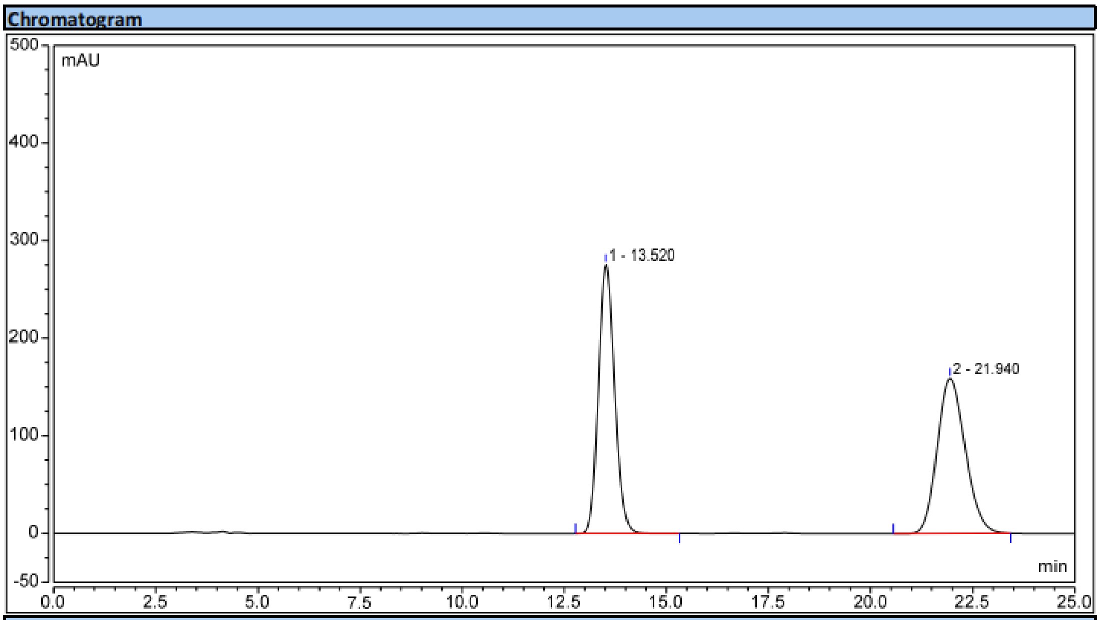

Integration Results

No.	Retention Time min	Area mAu*min	Height mAU	Relative Area %	Relative Height %
1	13.520	126.853	275.937	50.13	63.44
2	21.940	126.176	159.002	49.87	36.56
**Total:**		**253.029**	**434.939**	**100.00**	**100.00**

methyl (S)-2-(1,3-dibenzyl-5-methyl-2-oxoindolin-3-yl)acetate (**4g**)

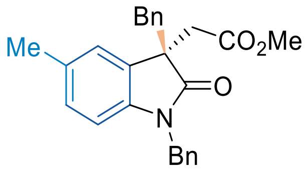

Chromatogram

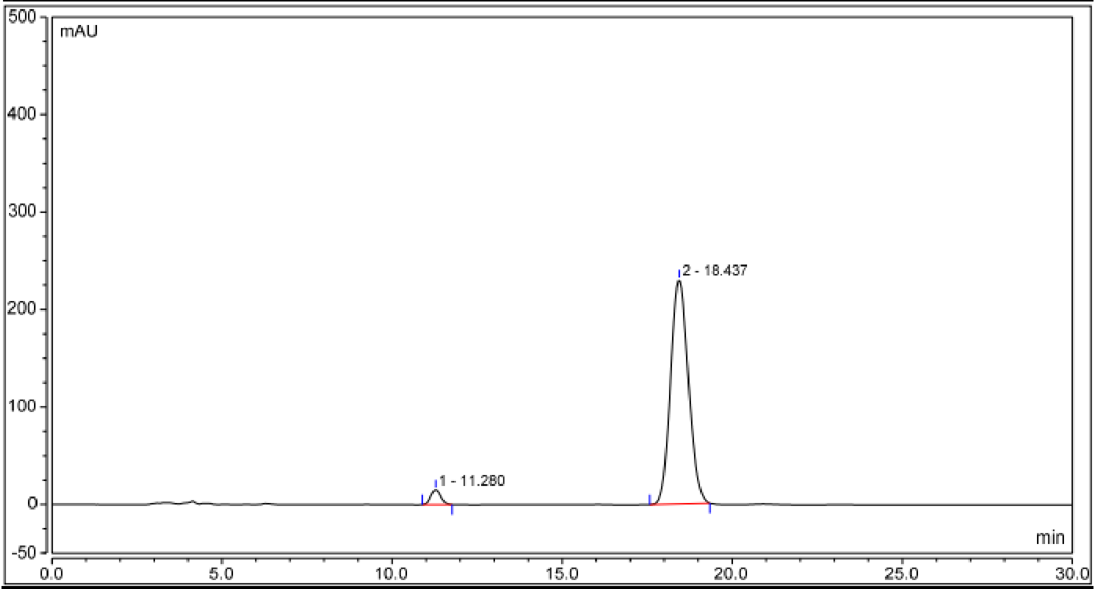

Integration Results

No.	Retention Time min	Area mAu*min	Height mAU	Relative Area %	Relative Height %
1	11.280	5.222	15.254	3.61	6.22
2	18.437	139.573	229.893	96.39	93.78
**Total:**		**144.795**	**245.147**	**100.00**	**100.00**

Chromatogram

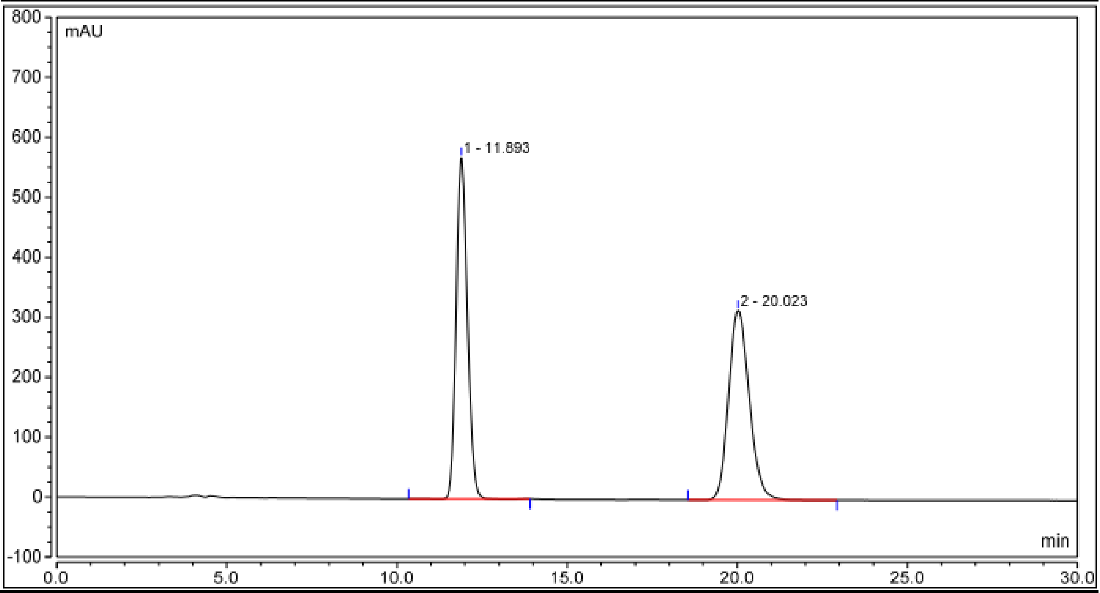

Integration Results

No.	Retention Time min	Area mAu*min	Height mAU	Relative Area %	Relative Height %
1	11.893	217.921	568.923	49.47	64.26
2	20.023	222.602	316.483	50.53	35.74
**Total:**		**440.522**	**885.406**	**100.00**	**100.00**

methyl (S)-2-(1,3-dibenzyl-7-methyl-2-oxoindolin-3-yl)acetate (**4h**)

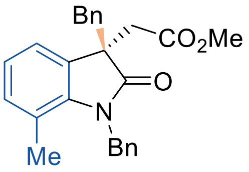

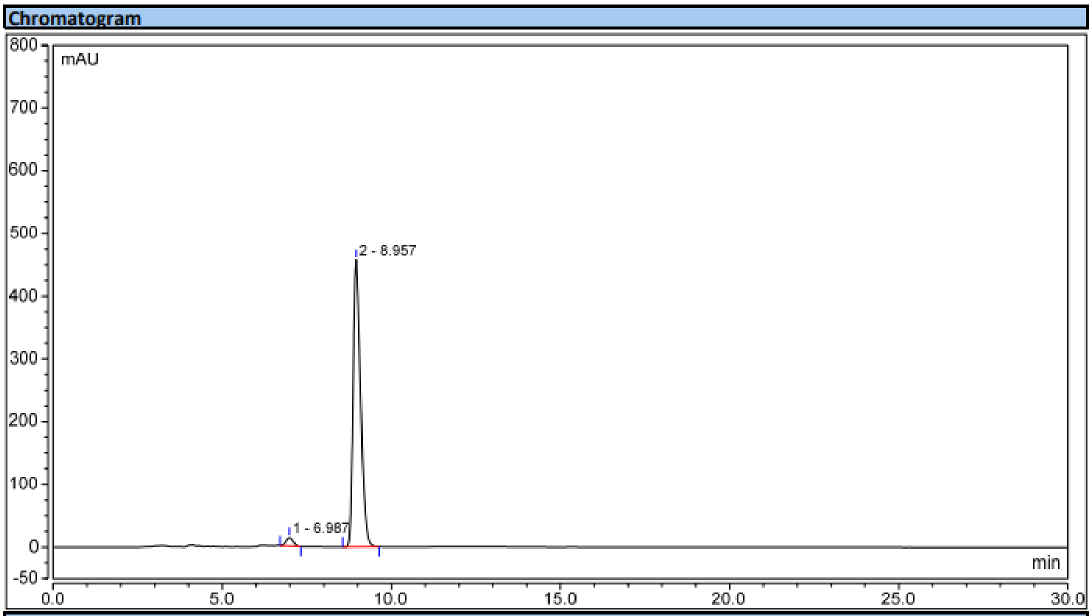

Integration Results

No.	Retention Time min	Area mAu*min	Height mAU	Relative Area %	Relative Height %
1	6.987	3.341	13.663	2.75	2.90
2	8.957	118.021	458.038	97.25	97.10
**Total:**		**121.362**	**471.702**	**100.00**	**100.00**

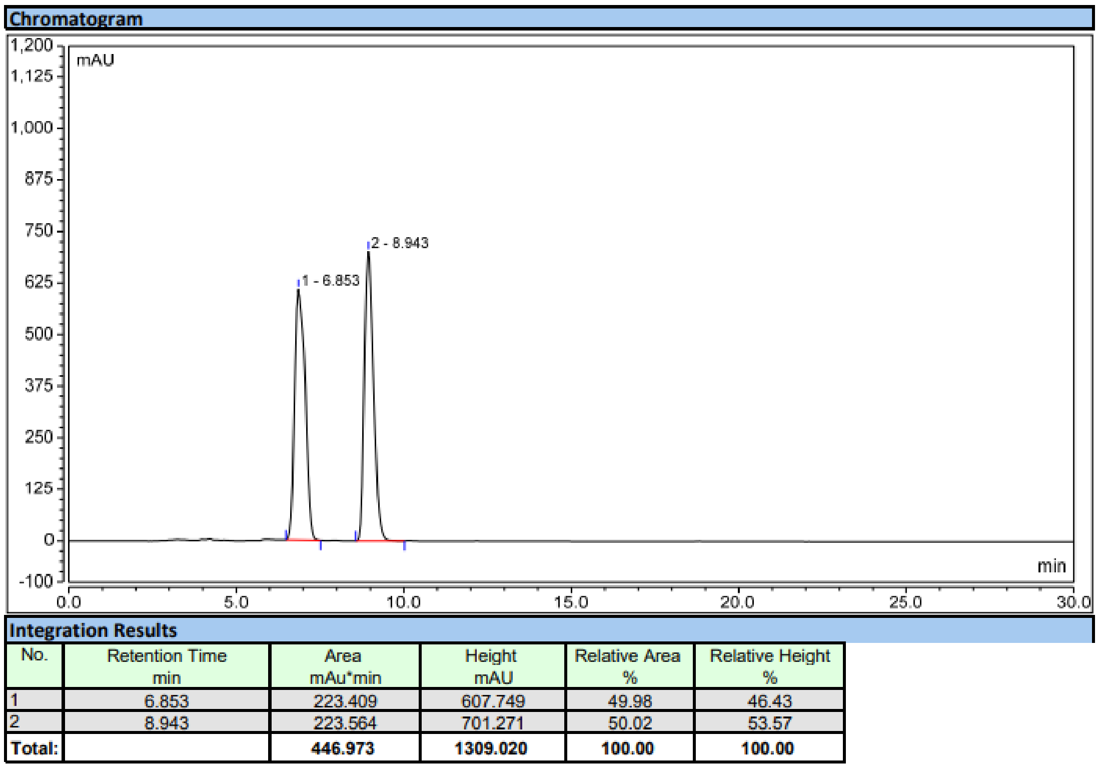

Integration Results

No.	Retention Time min	Area mAu*min	Height mAU	Relative Area %	Relative Height %
1	6.853	223.409	607.749	49.98	46.43
2	8.943	223.564	701.271	50.02	53.57
**Total:**		**446.973**	**1309.020**	**100.00**	**100.00**

methyl (S)-2-(1,3-dibenzyl-7-fluoro-2-oxoindolin-3-yl)acetate (**4i**)

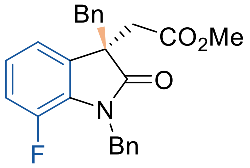

Chromatogram

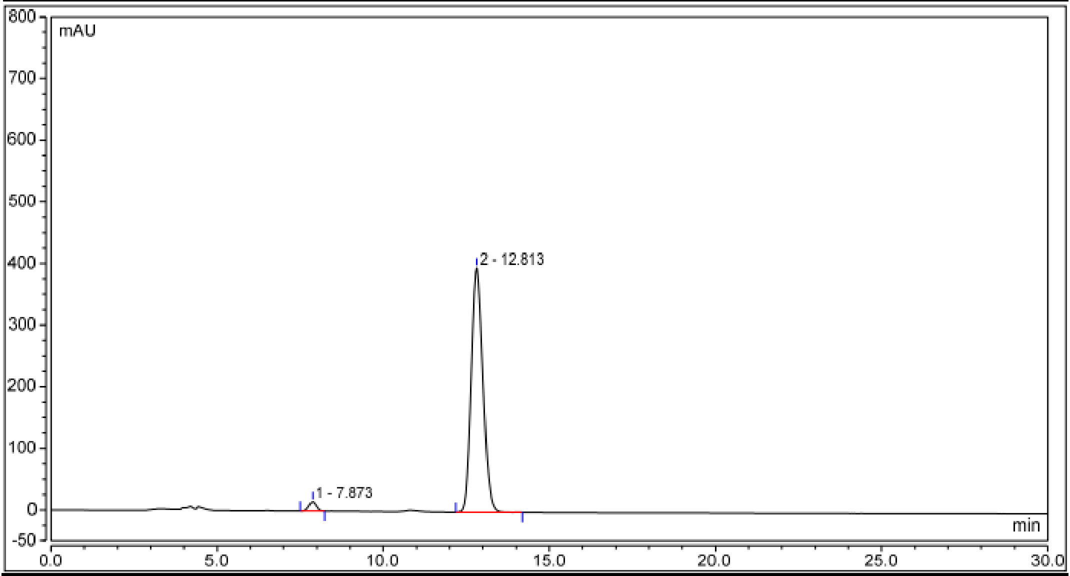

Integration Results

No.	Retention Time min	Area mAu*min	Height mAU	Relative Area %	Relative Height %
1	7.873	4.045	15.290	2.42	3.71
2	12.813	163.406	396.329	97.58	96.29
**Total:**		**167.451**	**411.619**	**100.00**	**100.00**

Chromatogram

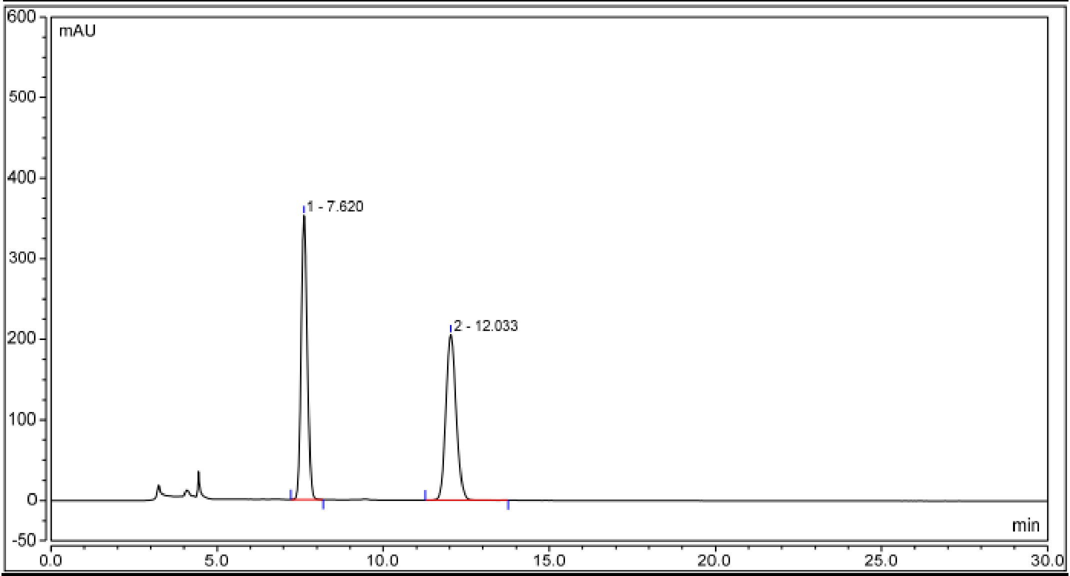

Integration Results

No.	Retention Time min	Area mAu*min	Height mAU	Relative Area %	Relative Height %
1	7.620	74.562	352.361	50.11	63.19
2	12.033	74.234	205.249	49.89	36.81
**Total:**		**148.796**	**557.610**	**100.00**	**100.00**

methyl (S)-2-(1,3-dibenzyl-6-chloro-2-oxoindolin-3-yl)acetate (**4j**)

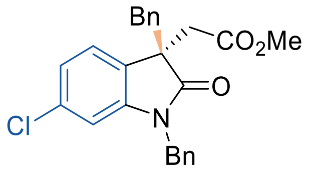

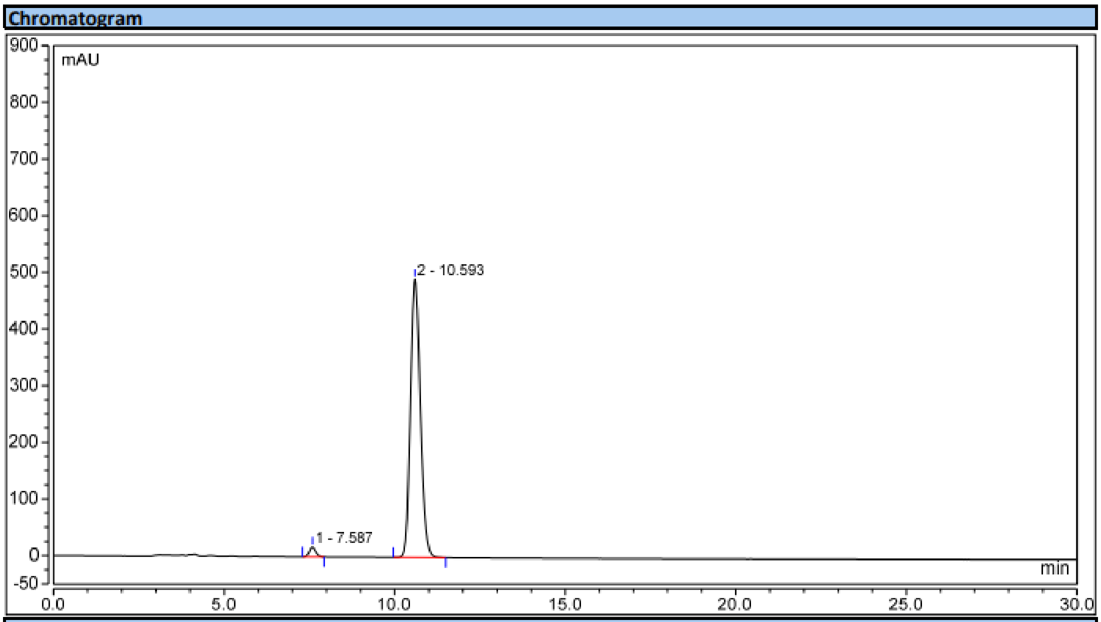

Integration Results

No.	Retention Time min	Area mAu*min	Height mAU	Relative Area %	Relative Height %
1	7.587	3.827	17.181	2.26	3.38
2	10.593	165.436	491.534	97.74	96.62
**Total:**		**169.262**	**508.715**	**100.00**	**100.00**

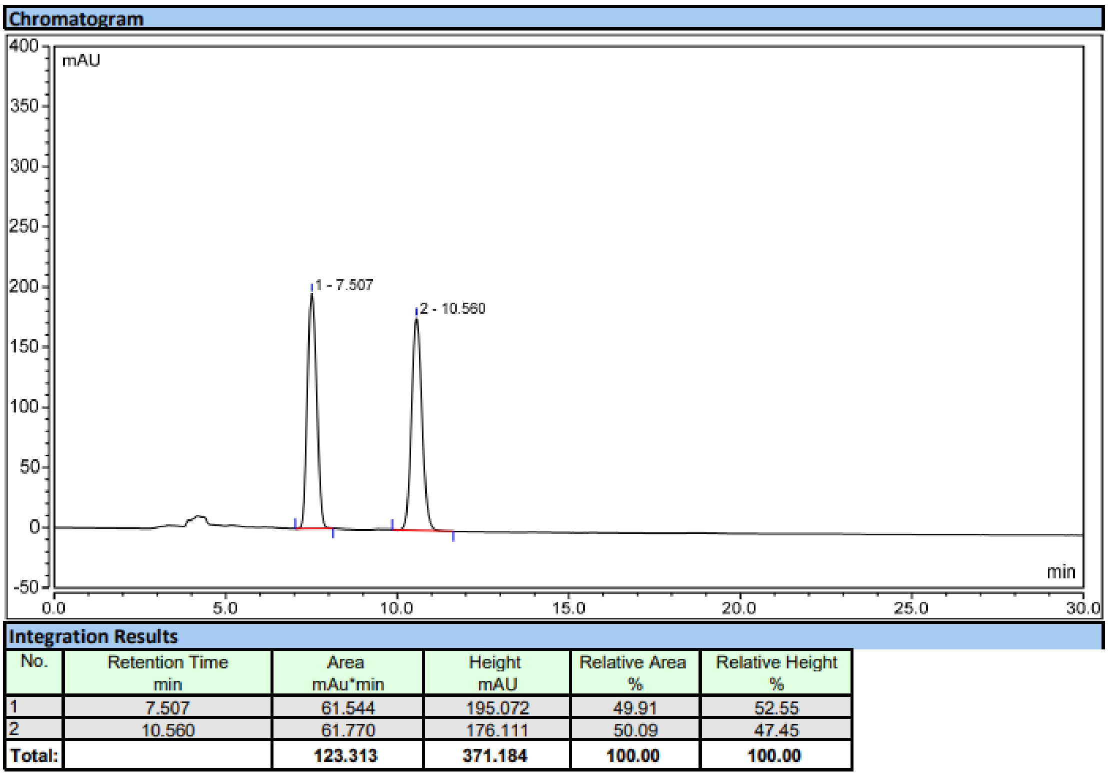

Integration Results

No.	Retention Time min	Area mAu*min	Height mAU	Relative Area %	Relative Height %
1	7.507	61.544	195.072	49.91	52.55
2	10.560	61.770	176.111	50.09	47.45
**Total:**		**123.313**	**371.184**	**100.00**	**100.00**

methyl (S)-2-(1,3-dibenzyl-7-bromo-2-oxoindolin-3-yl)acetate (**4k**)

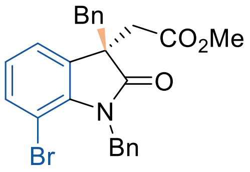

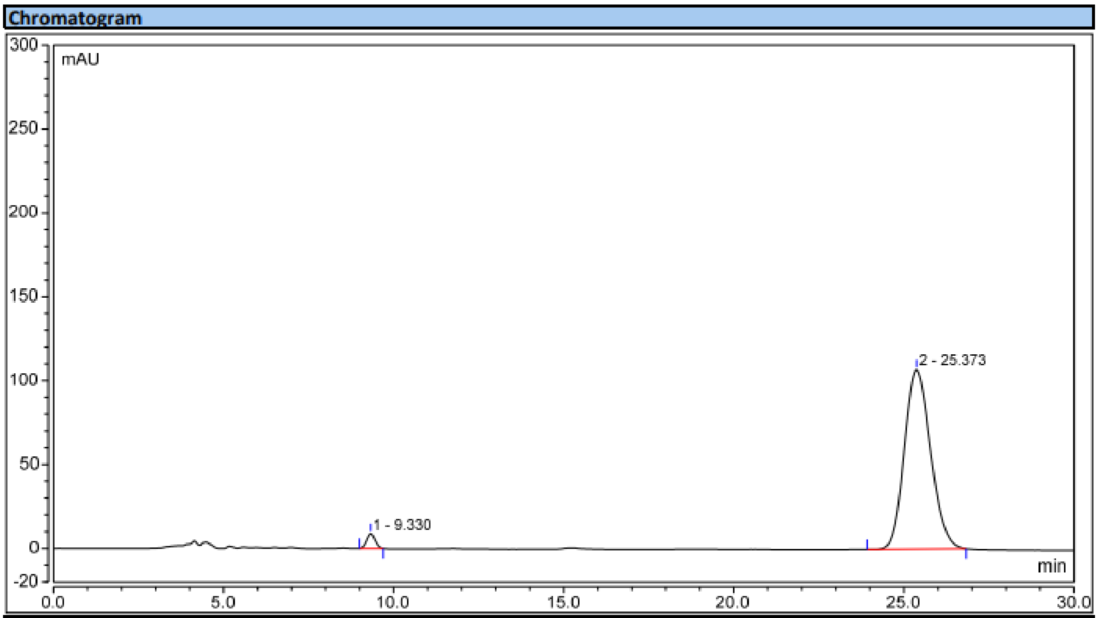

Integration Results

No.	Retention Time min	Area mAu*min	Height mAU	Relative Area %	Relative Height %
1	9.330	2.526	8.905	2.57	7.68
2	25.373	95.829	107.016	97.43	92.32
**Total:**		**98.355**	**115.922**	**100.00**	**100.00**

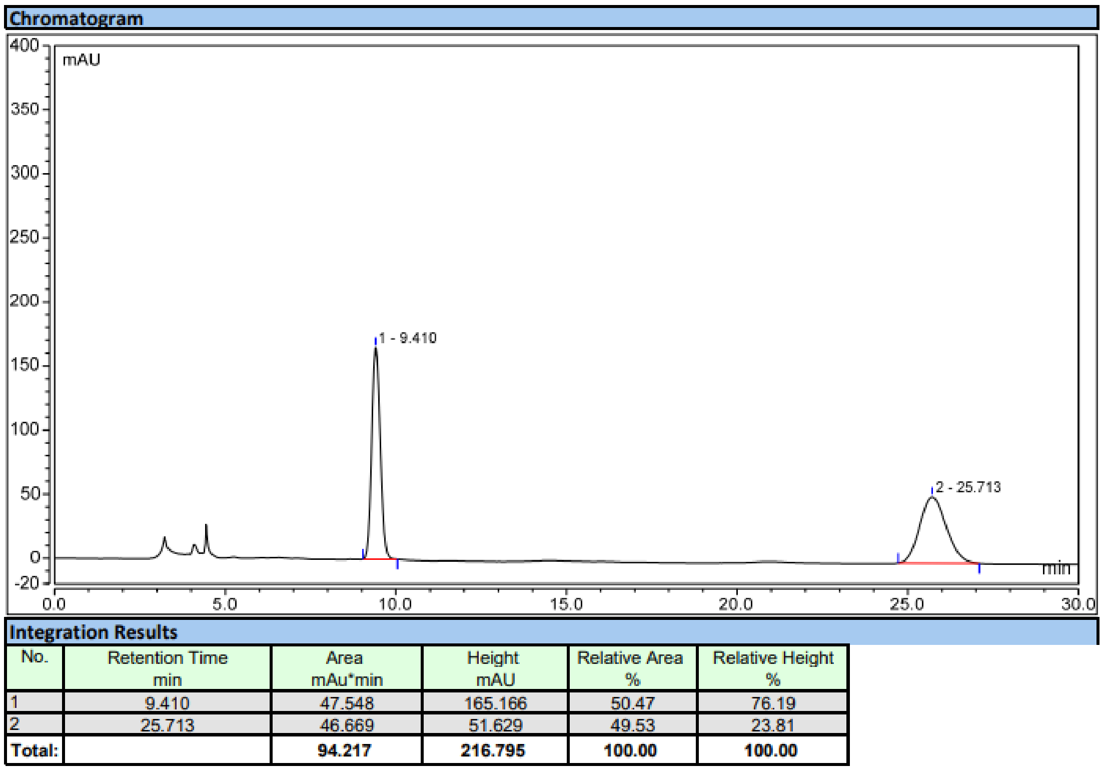

Integration Results

No.	Retention Time min	Area mAu*min	Height mAU	Relative Area %	Relative Height %
1	9.410	47.548	165.166	50.47	76.19
2	25.713	46.669	51.629	49.53	23.81
**Total:**		**94.217**	**216.795**	**100.00**	**100.00**

methyl (S)-2-(1,3-dibenzyl-4,6-difluoro-2-oxoindolin-3-yl)acetate (**4l**)

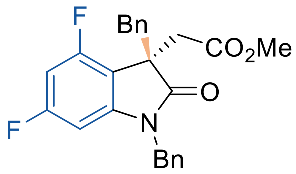

Chromatogram

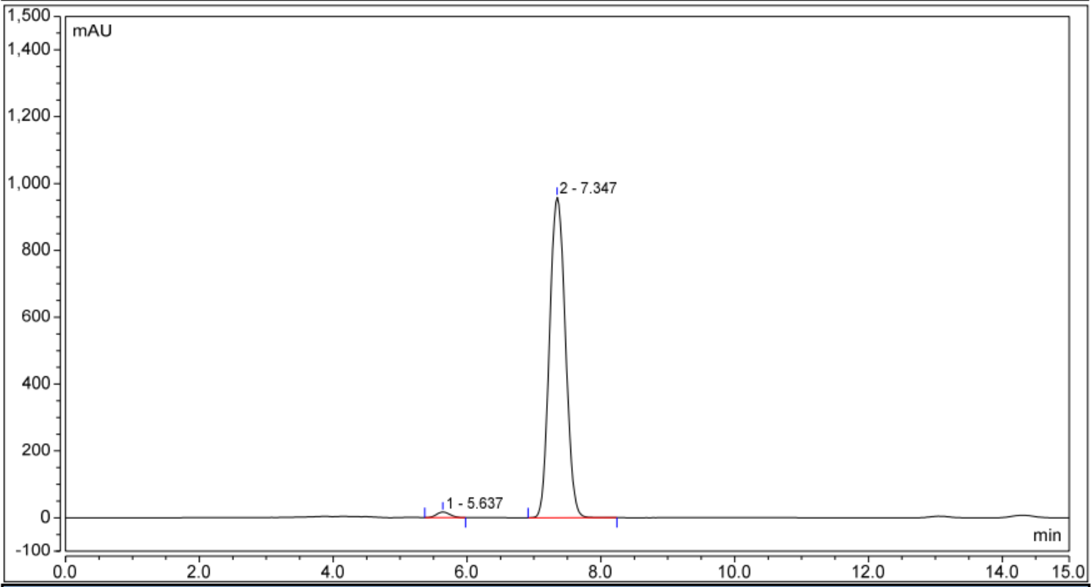

Integration Results

No.	Retention Time<br>min	Area<br>mAu*min	Height<br>mAU	Relative Area<br>%	Relative Height<br>%
1	5.637	3.931	16.790	1.47	1.72
2	7.347	264.044	958.627	98.53	98.28
**Total:**		**267.975**	**975.416**	**100.00**	**100.00**

Chromatogram

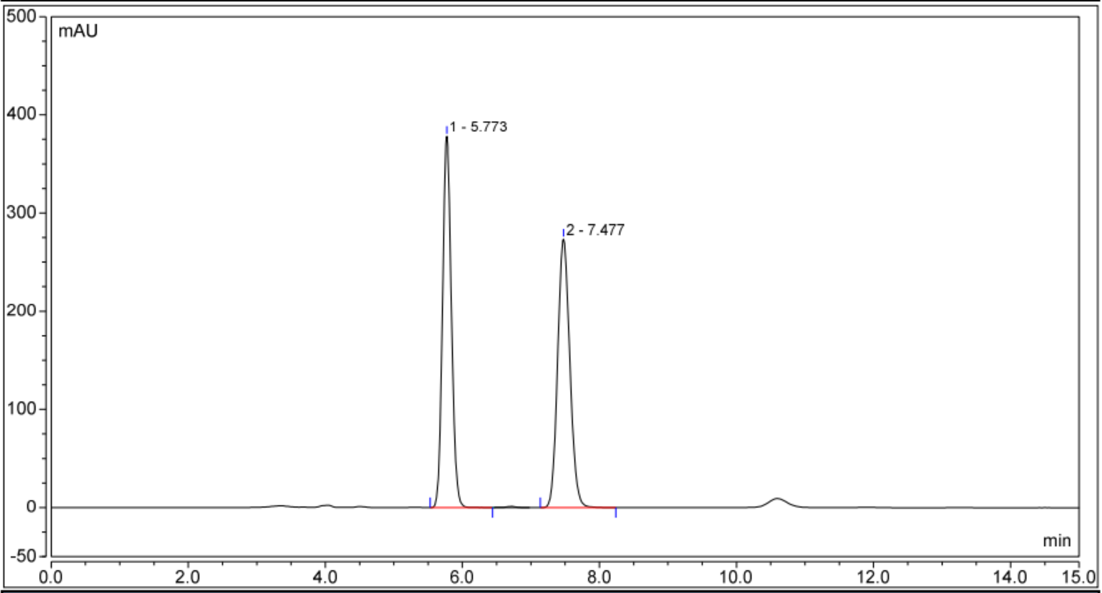

Integration Results

No.	Retention Time<br>min	Area<br>mAu*min	Height<br>mAU	Relative Area<br>%	Relative Height<br>%
1	5.773	56.448	378.261	49.98	58.03
2	7.477	56.493	273.581	50.02	41.97
**Total:**		**112.942**	**651.841**	**100.00**	**100.00**

methyl (S)-2-(1,3-dibenzyl-4,7-dichloro-2-oxoindolin-3-yl)acetate (**4m**)

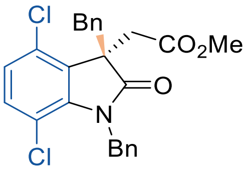

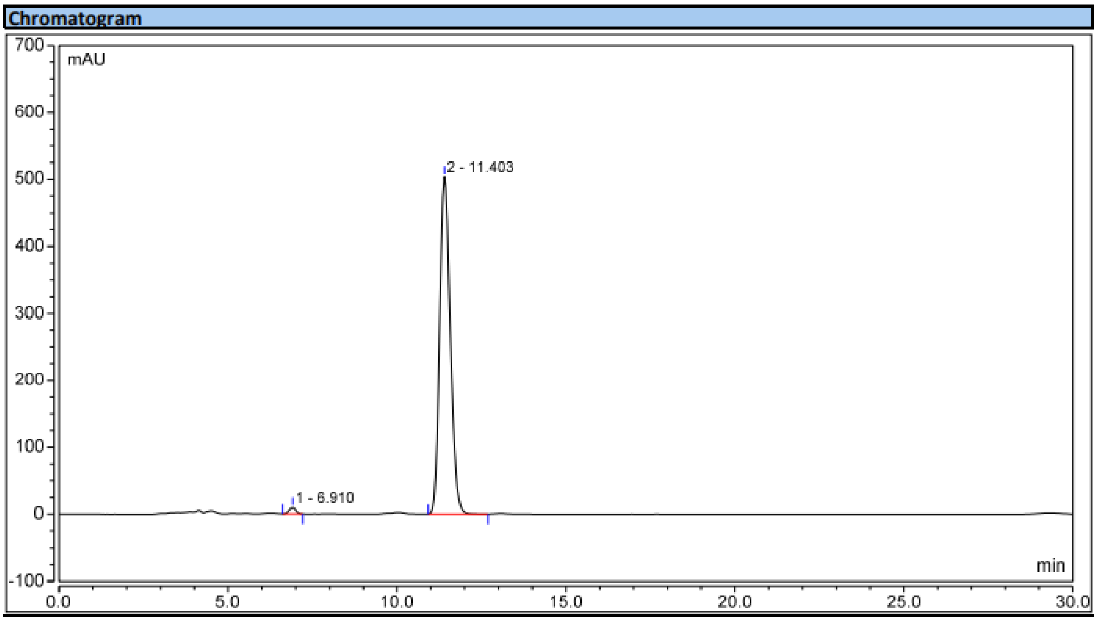

Integration Results

No.	Retention Time min	Area mAu*min	Height mAU	Relative Area %	Relative Height %
1	6.910	2.251	9.775	1.20	1.90
2	11.403	184.558	504.208	98.80	98.10
**Total:**		**186.809**	**513.983**	**100.00**	**100.00**

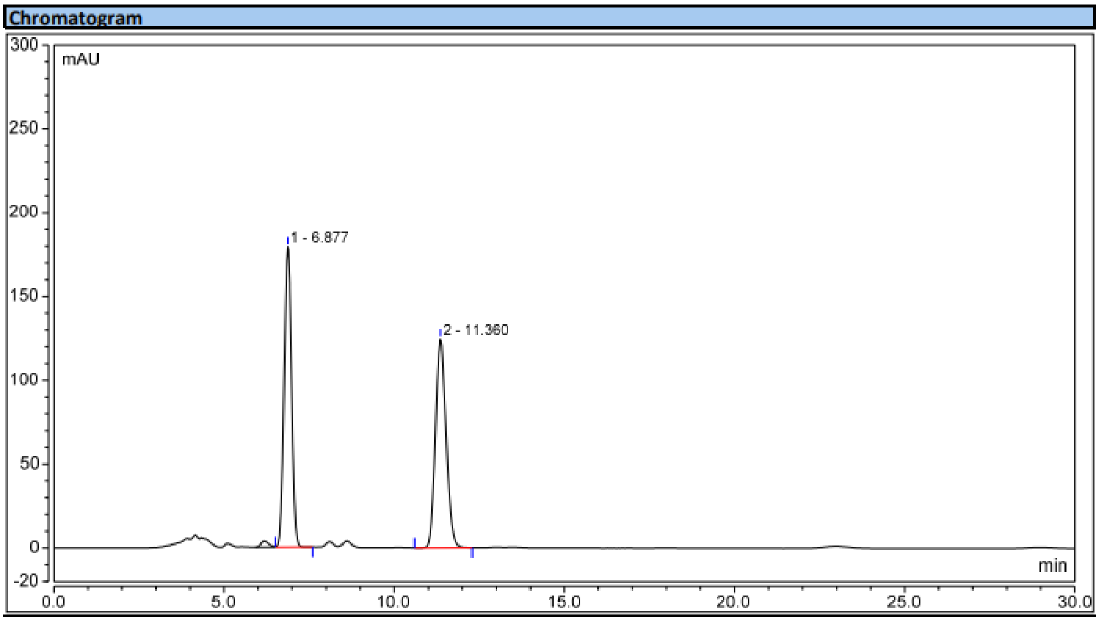

Integration Results

No.	Retention Time min	Area mAu*min	Height mAU	Relative Area %	Relative Height %
1	6.877	45.135	179.123	50.01	58.97
2	11.360	45.121	124.645	49.99	41.03
**Total:**		**90.256**	**303.768**	**100.00**	**100.00**

methyl (S)-2-(1,3-dibenzyl-5,7-dimethyl-2-oxoindolin-3-yl)acetate (**4n**)

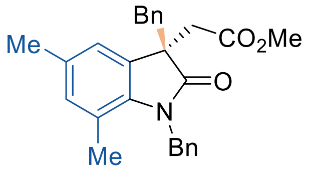

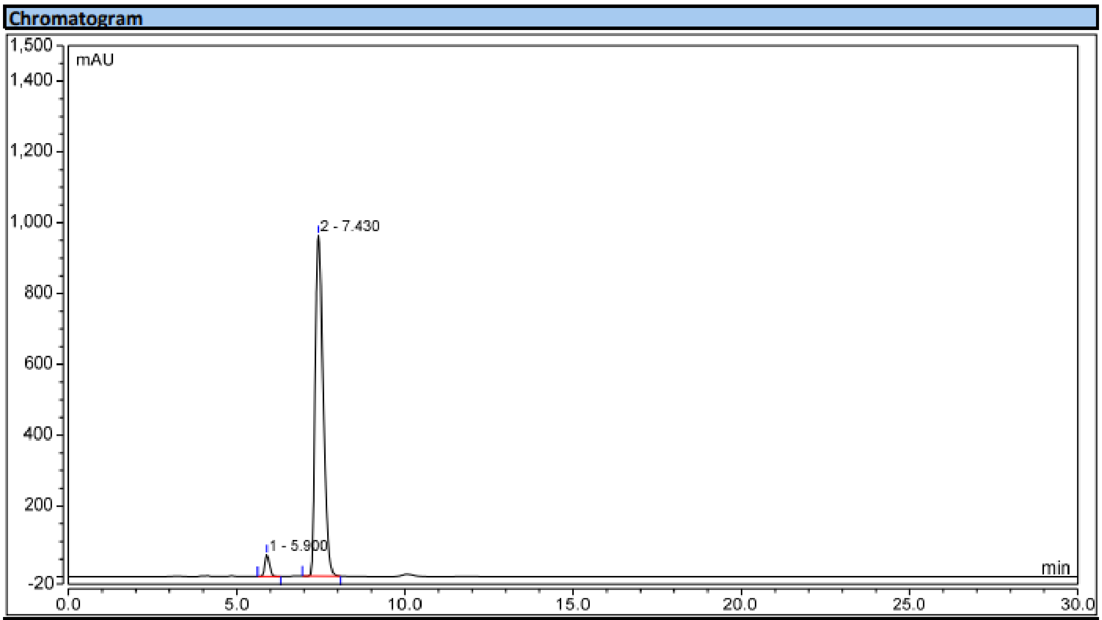

Integration Results

No.	Retention Time min	Area mAu*min	Height mAU	Relative Area %	Relative Height %
1	5.900	9.653	61.037	3.53	5.97
2	7.430	264.025	961.588	96.47	94.03
**Total:**		**273.678**	**1022.624**	**100.00**	**100.00**

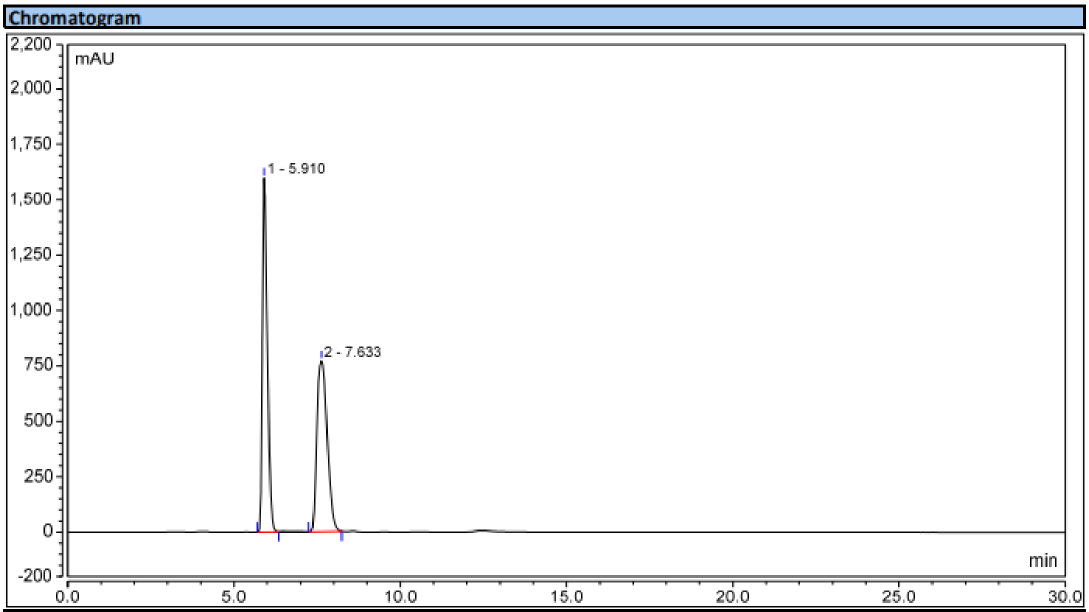

Integration Results

No.	Retention Time min	Area mAu*min	Height mAU	Relative Area %	Relative Height %
1	5.910	276.873	1598.174	49.94	67.45
2	7.633	277.507	771.288	50.06	32.55
**Total:**		**554.380**	**2369.462**	**100.00**	**100.00**

methyl (S)-2-(1,3-dibenzyl-5-chloro-7-methyl-2-oxoindolin-3-yl)acetate (**4o**)

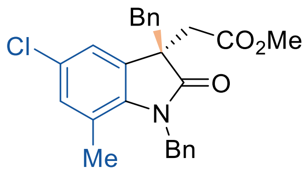

Chromatogram

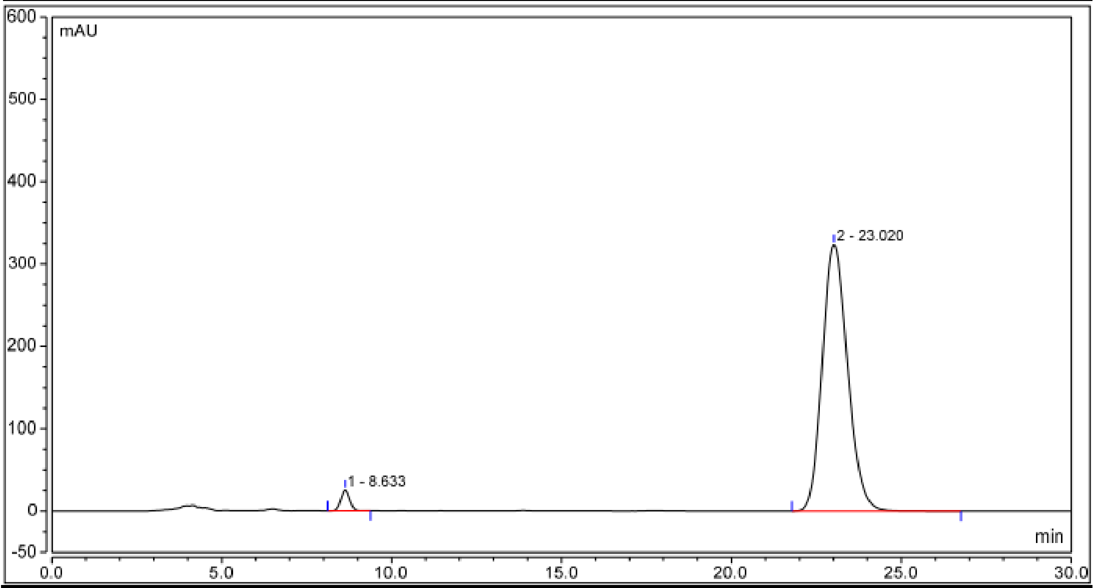

Integration Results

No.	Retention Time min	Area mAu*min	Height mAU	Relative Area %	Relative Height %
1	8.633	7.533	25.205	2.62	7.23
2	23.020	279.892	323.599	97.38	92.77
**Total:**		**287.425**	**348.804**	**100.00**	**100.00**

Chromatogram

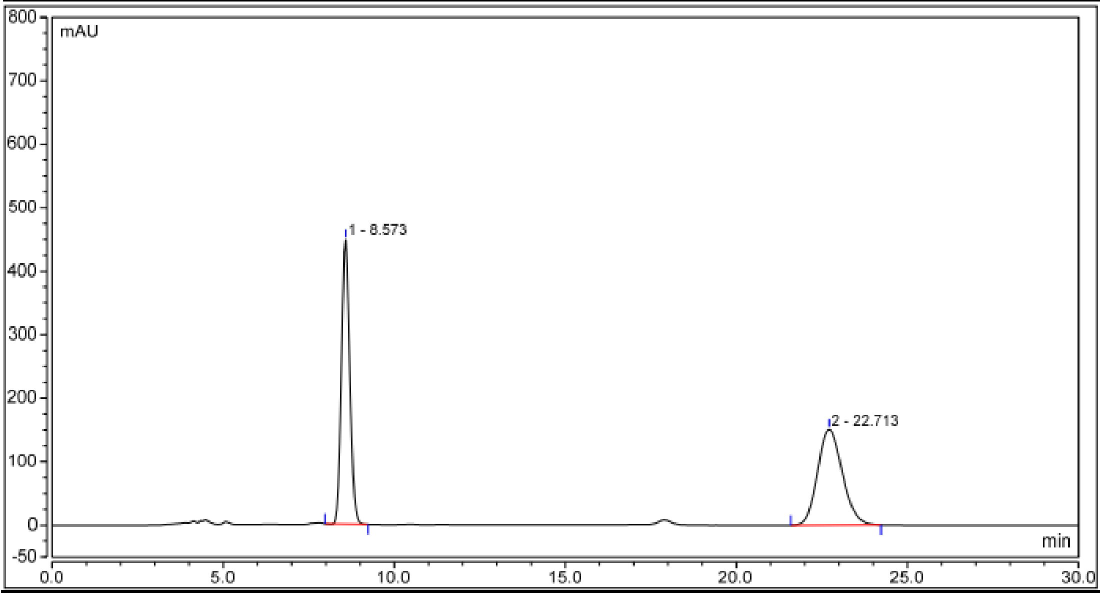

Integration Results

No.	Retention Time min	Area mAu*min	Height mAU	Relative Area %	Relative Height %
1	8.573	125.749	447.941	49.85	74.85
2	22.713	126.490	150.542	50.15	25.15
**Total:**		**252.239**	**598.483**	**100.00**	**100.00**

methyl (S)-2-(3-benzyl-1-methyl-2-oxoindolin-3-yl)acetate (**4p**)

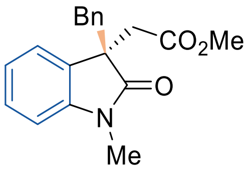

Chromatogram

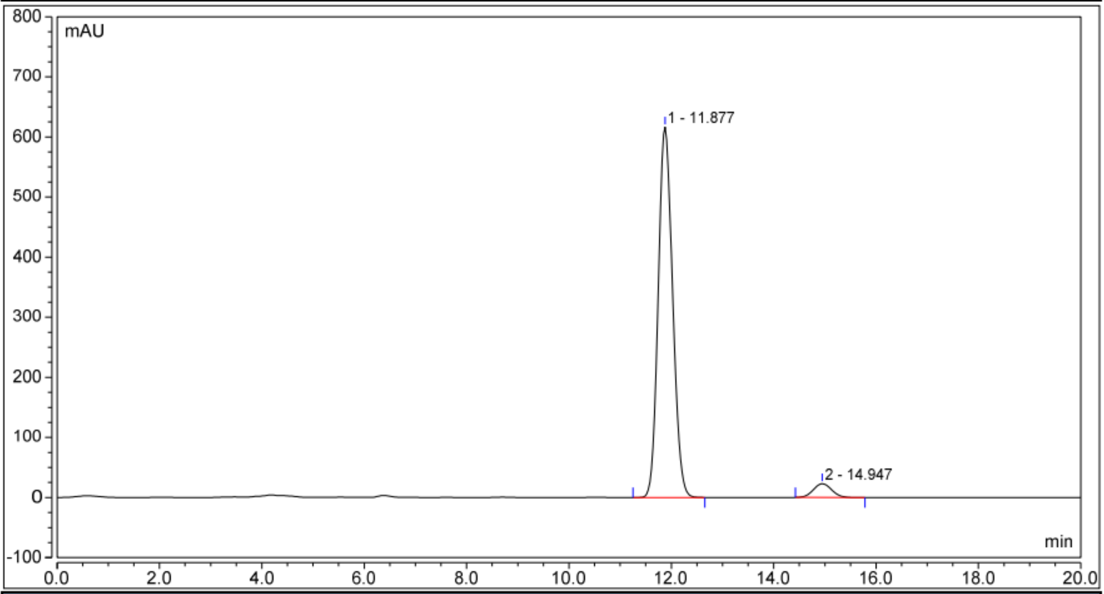

Integration Results

No.	Retention Time<br>min	Area<br>mAu*min	Height<br>mAU	Relative Area<br>%	Relative Height<br>%
1	11.877	200.485	616.592	95.46	96.41
2	14.947	9.538	22.937	4.54	3.59
**Total:**		**210.023**	**639.528**	**100.00**	**100.00**

Chromatogram

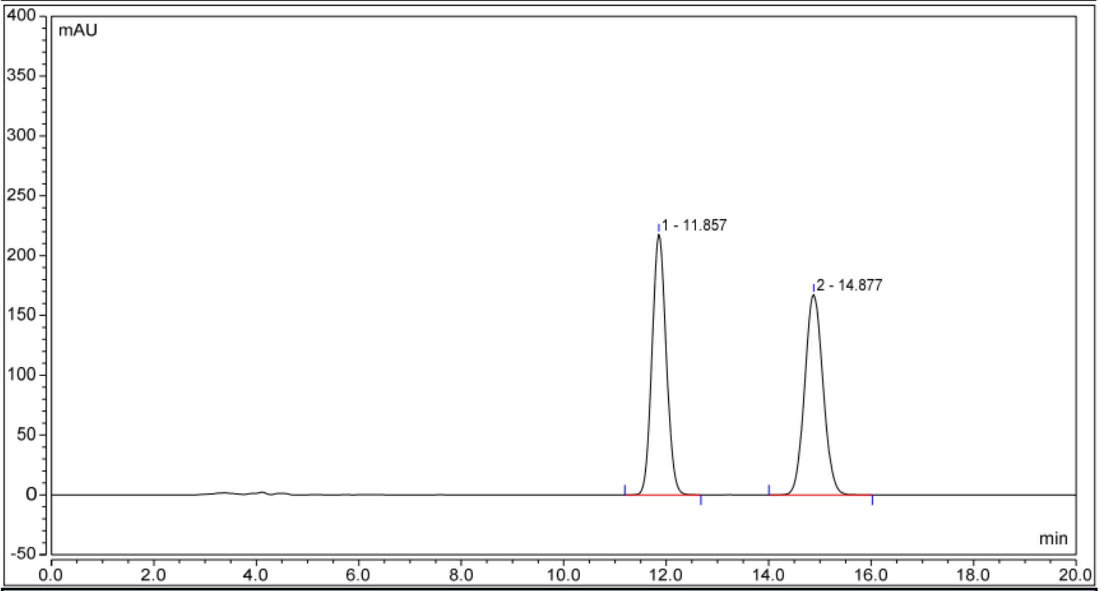

Integration Results

No.	Retention Time<br>min	Area<br>mAu*min	Height<br>mAU	Relative Area<br>%	Relative Height<br>%
1	11.857	69.644	218.029	50.01	56.53
2	14.877	69.605	167.670	49.99	43.47
**Total:**		**139.249**	**385.699**	**100.00**	**100.00**

ethyl (S)-2-(1,3-dibenzyl-4,7-dichloro-2-oxoindolin-3-yl)acetate (**4q**)

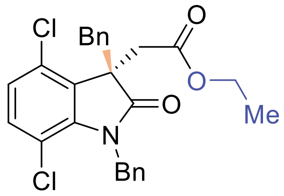

Chromatogram

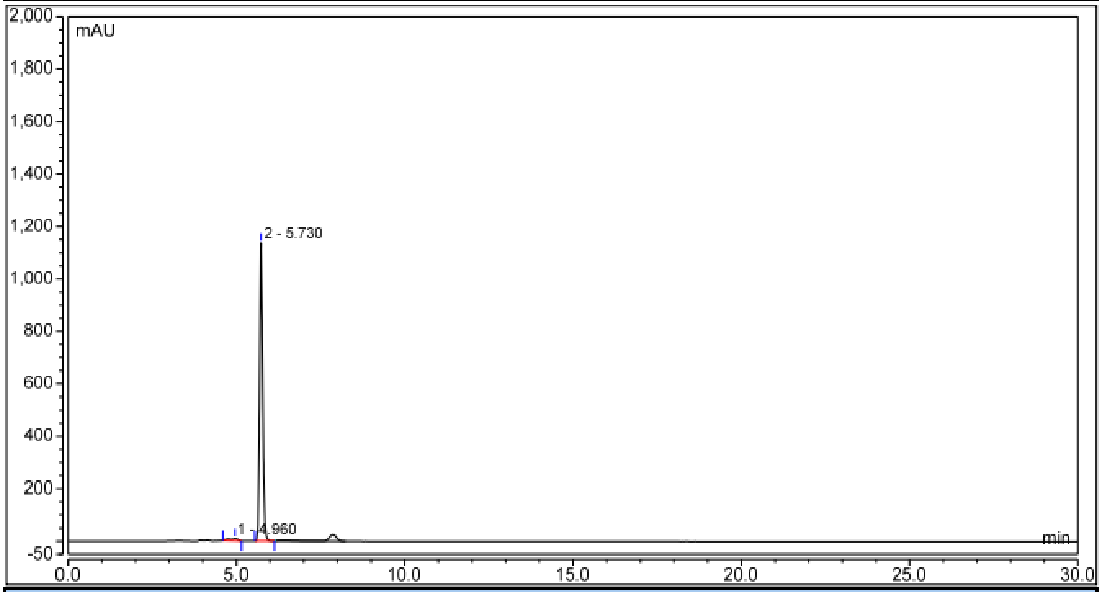

Integration Results

No.	Retention Time min	Area mAu*min	Height mAU	Relative Area %	Relative Height %
1	4.960	2.583	8.182	2.06	0.72
2	5.730	122.979	1135.690	97.94	99.28
**Total:**		**125.562**	**1143.872**	**100.00**	**100.00**

Chromatogram

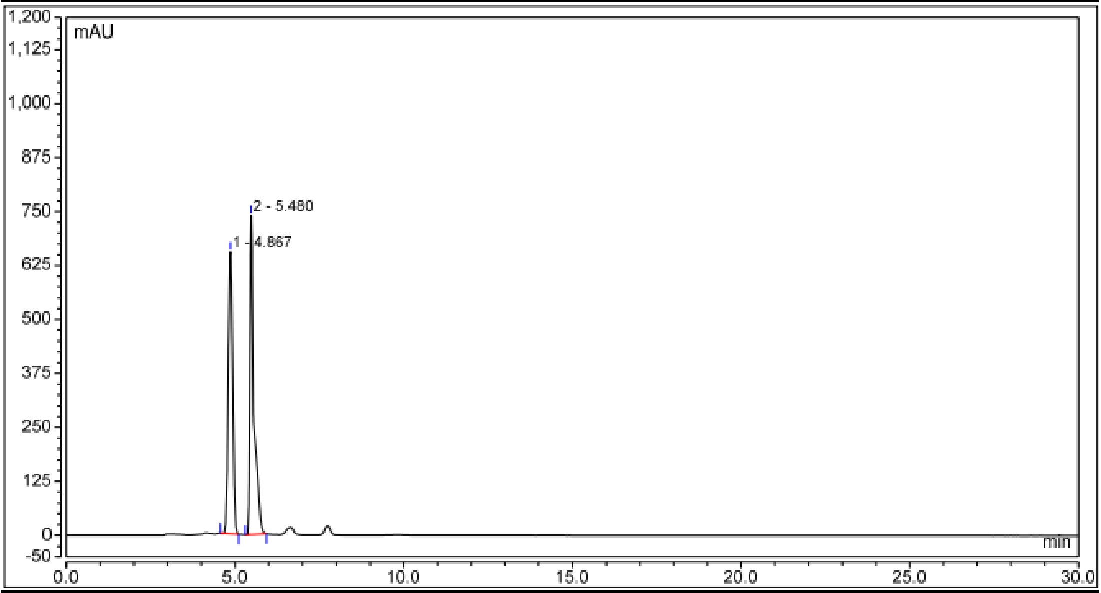

Integration Results

No.	Retention Time min	Area mAu*min	Height mAU	Relative Area %	Relative Height %
1	4.867	98.000	653.528	49.70	46.95
2	5.480	99.195	738.574	50.30	53.05
**Total:**		**197.195**	**1392.103**	**100.00**	**100.00**

benzyl (S)-2-(1,3-dibenzyl-4,7-dichloro-2-oxoindolin-3-yl)acetate (**4r**)

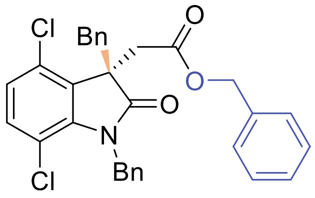

Chromatogram

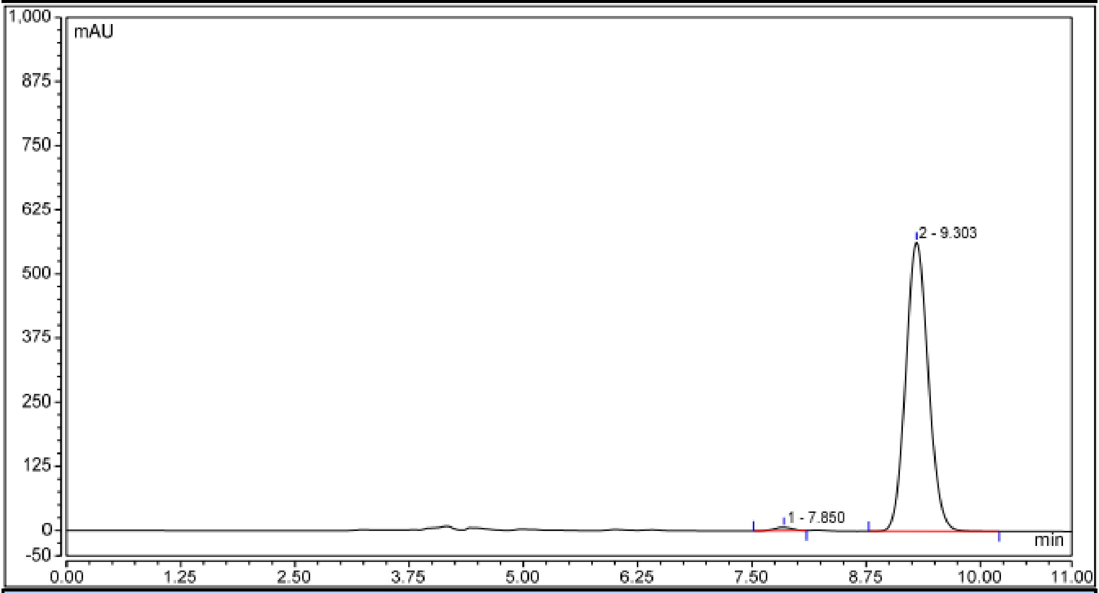

Integration Results

No.	Retention Time min	Area mAu*min	Height mAU	Relative Area %	Relative Height %
1	7.850	1.526	6.548	0.94	1.15
2	9.303	161.409	563.170	99.06	98.85
**Total:**		**162.935**	**569.718**	**100.00**	**100.00**

Chromatogram

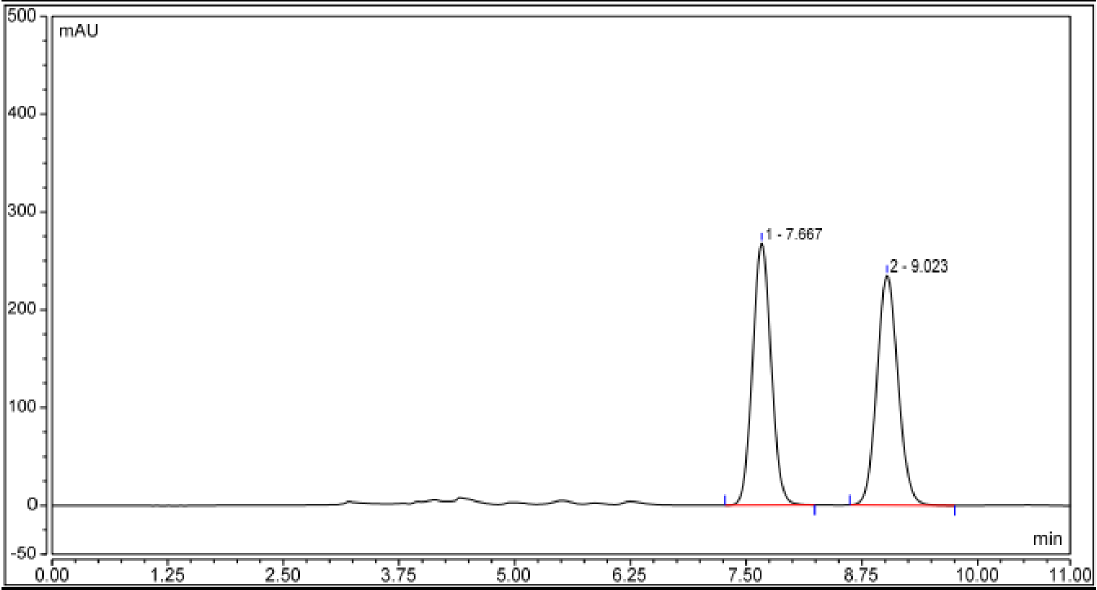

Integration Results

No.	Retention Time min	Area mAu*min	Height mAU	Relative Area %	Relative Height %
1	7.667	62.230	267.995	49.95	53.29
2	9.023	62.364	234.888	50.05	46.71
**Total:**		**124.594**	**502.883**	**100.00**	**100.00**

(S)-1,3-dibenzyl-4,7-dichloro-3-(2-oxo-2-(1H-pyrazol-1-yl)ethyl)indolin-2-one (**4s**)

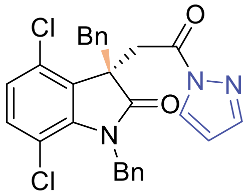

Chromatogram

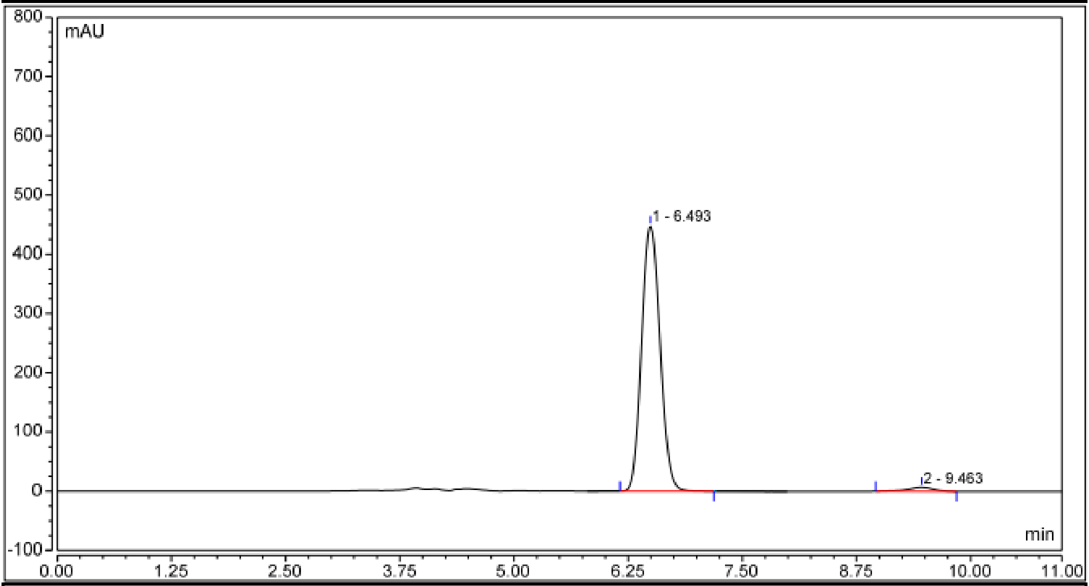

Integration Results

No.	Retention Time min	Area mAu*min	Height mAU	Relative Area %	Relative Height %
1	6.493	105.254	447.930	97.94	98.49
2	9.463	2.215	6.848	2.06	1.51
**Total:**		**107.469**	**454.778**	**100.00**	**100.00**

Chromatogram

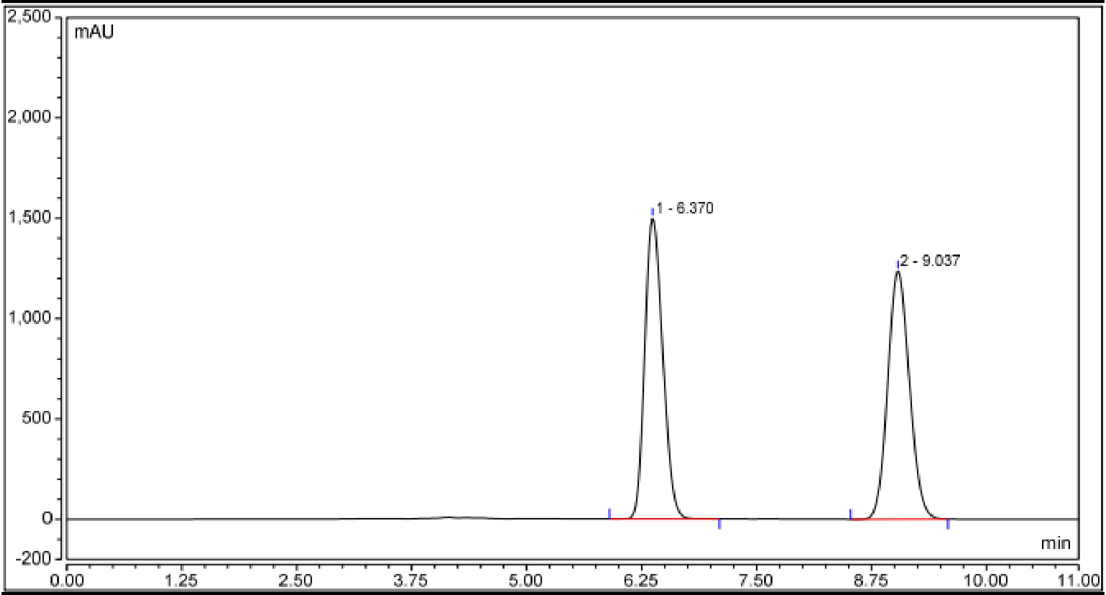

Integration Results

No.	Retention Time min	Area mAu*min	Height mAU	Relative Area %	Relative Height %
1	6.370	338.635	1497.990	49.95	54.77
2	9.037	339.365	1236.951	50.05	45.23
**Total:**		**678.001**	**2734.941**	**100.00**	**100.00**

2-(1-(4-chlorobenzoyl)-5-methoxy-2-methyl-1H-indol-3-yl)ethyl (S)-2-(1,3-dibenzyl-4,7-dichloro-2-oxoindolin-3-yl)acetate (**4t**)

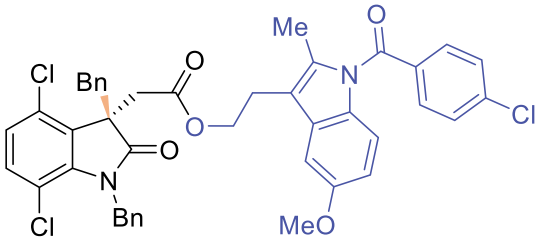

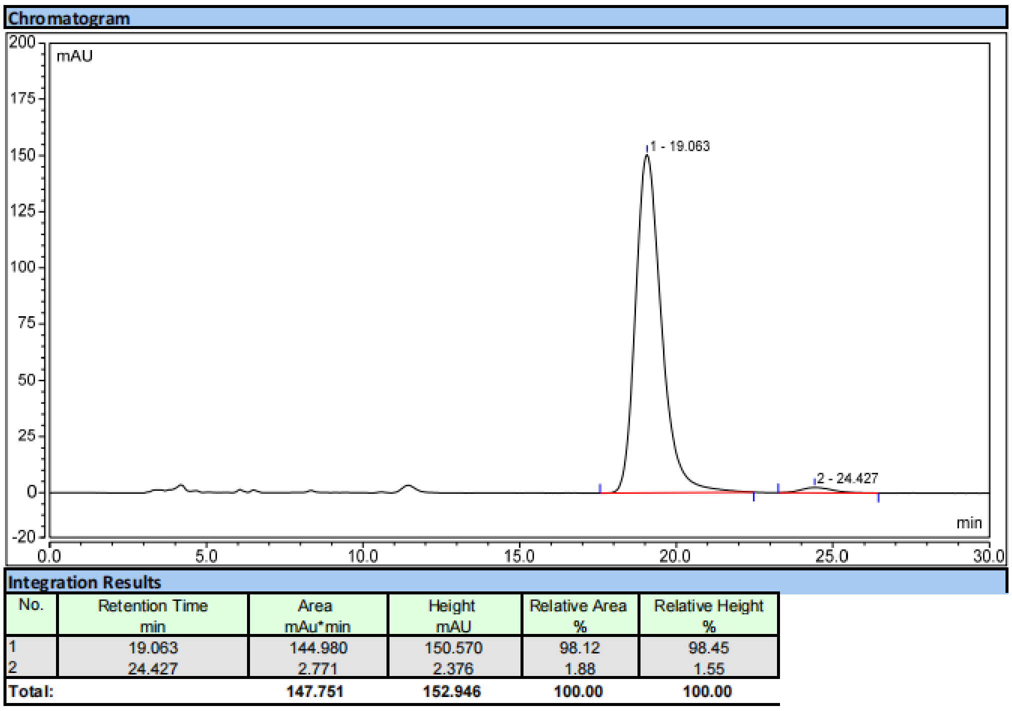

Integration Results

No.	Retention Time min	Area mAu*min	Height mAU	Relative Area %	Relative Height %
1	19.063	144.980	150.570	98.12	98.45
2	24.427	2.771	2.376	1.88	1.55
**Total:**		**147.751**	**152.946**	**100.00**	**100.00**

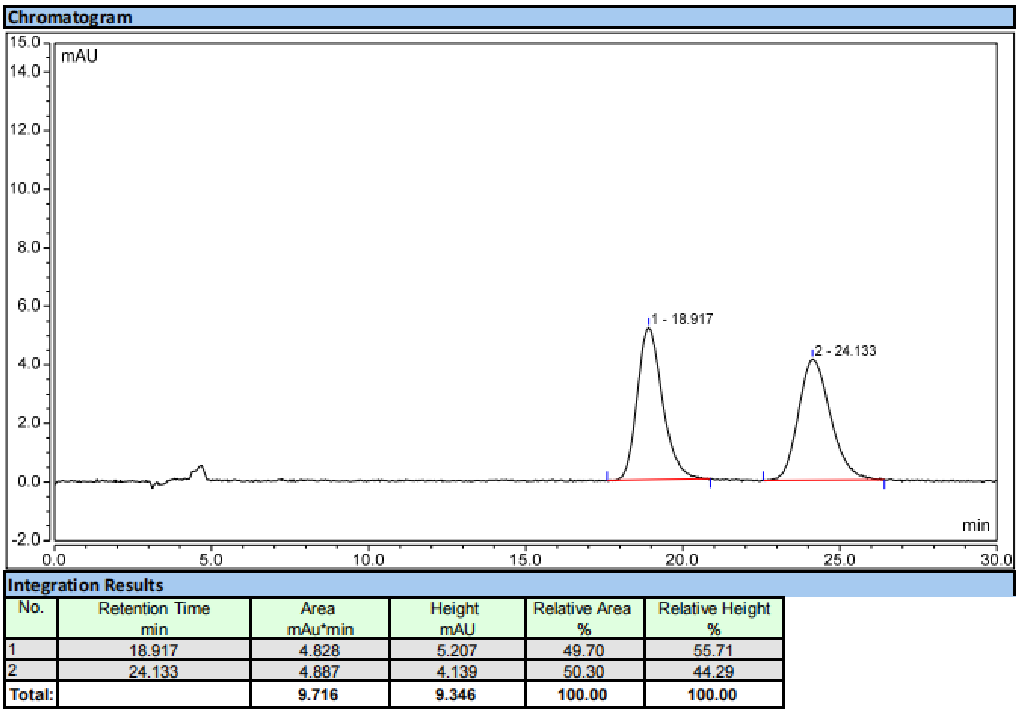

Integration Results

No.	Retention Time min	Area mAu*min	Height mAU	Relative Area %	Relative Height %
1	18.917	4.828	5.207	49.70	55.71
2	24.133	4.887	4.139	50.30	44.29
**Total:**		**9.716**	**9.346**	**100.00**	**100.00**

S-benzyl (S)-2-(1,3-dibenzyl-4,7-dichloro-2-oxoindolin-3-yl)ethanethioate (**4u**)

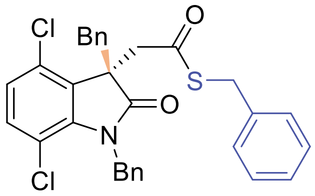

Chromatogram

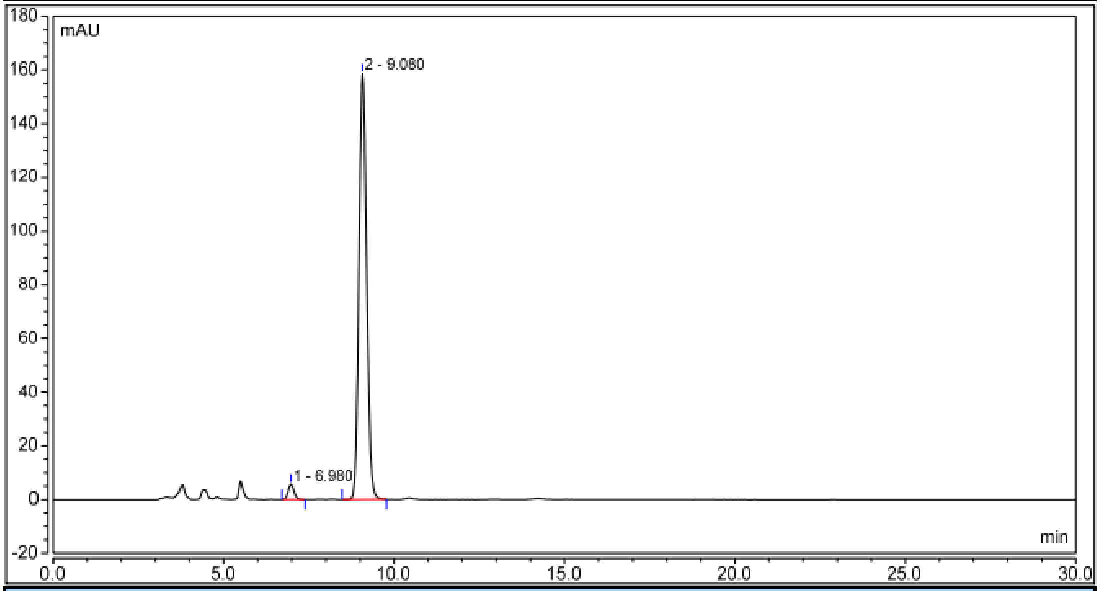

Integration Results

No.	Retention Time<br>min	Area<br>mAu*min	Height<br>mAU	Relative Area<br>%	Relative Height<br>%
1	6.980	1.111	5.675	2.62	3.46
2	9.080	41.212	158.500	97.38	96.54
Total:		42.323	164.175	100.00	100.00

Chromatogram

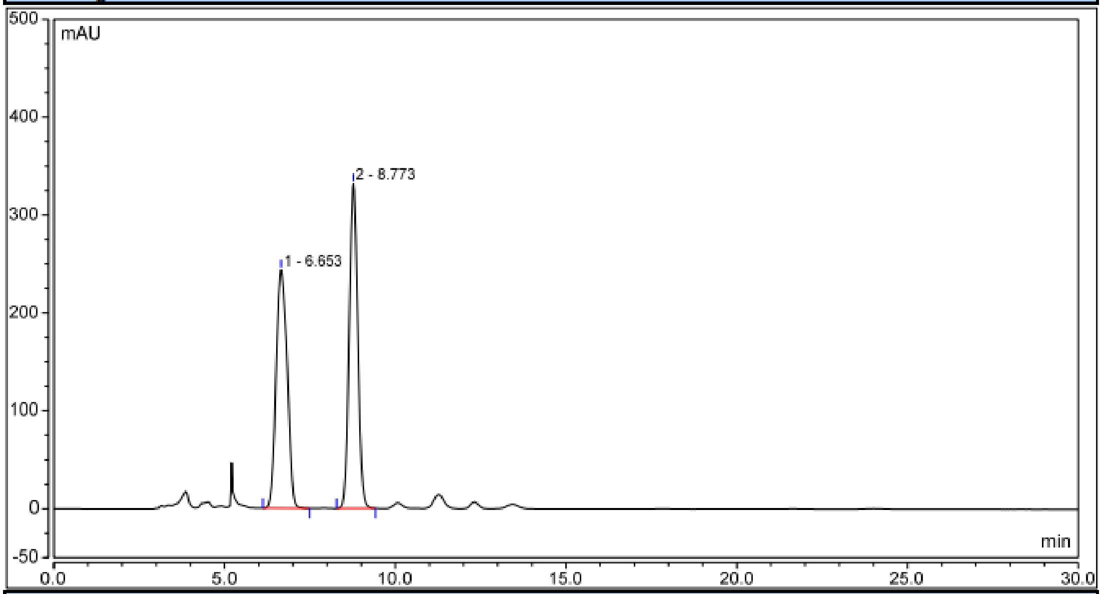

Integration Results

No.	Retention Time<br>min	Area<br>mAu*min	Height<br>mAU	Relative Area<br>%	Relative Height<br>%
1	6.653	91.770	243.272	49.02	42.31
2	8.773	95.439	331.704	50.98	57.69
Total:		187.209	574.976	100.00	100.00

methyl (S)-2-(1-benzyl-4,7-dichloro-3-(4-fluorobenzyl)-2-oxoindolin-3-yl)acetate (**5a**)

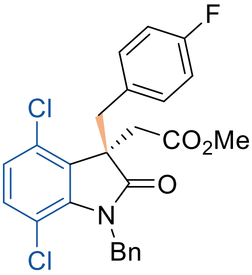

Chromatogram

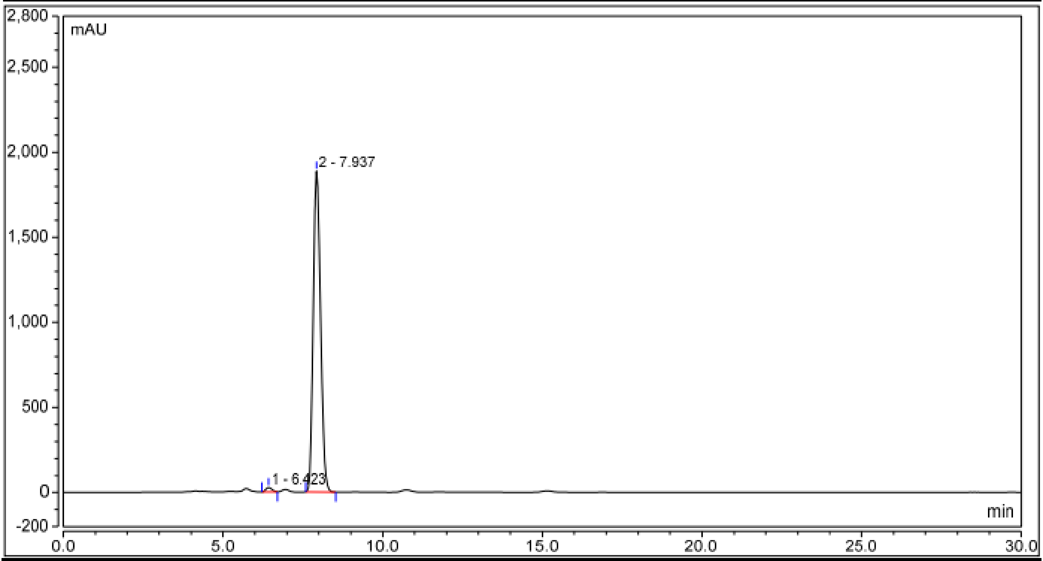

Integration Results

No.	Retention Time min	Area mAu*min	Height mAU	Relative Area %	Relative Height %
1	6.423	5.623	25.839	1.12	1.35
2	7.937	496.985	1887.295	98.88	98.65
**Total:**		**502.609**	**1913.134**	**100.00**	**100.00**

Chromatogram

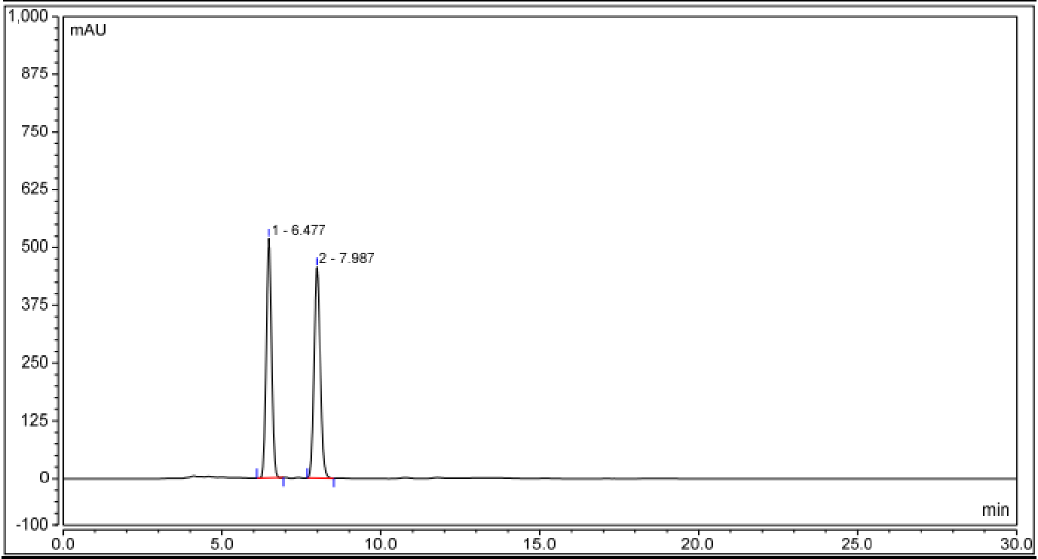

Integration Results

No.	Retention Time min	Area mAu*min	Height mAU	Relative Area %	Relative Height %
1	6.477	105.641	517.243	49.94	53.16
2	7.987	105.911	455.826	50.06	46.84
**Total:**		**211.552**	**973.069**	**100.00**	**100.00**

methyl (S)-2-(1-benzyl-3-(4-chlorobenzyl)-4,6-difluoro-2-oxoindolin-3-yl)acetate (**5b**)

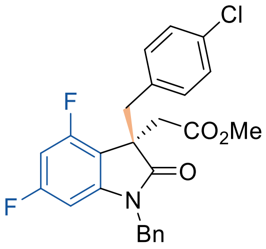

Chromatogram

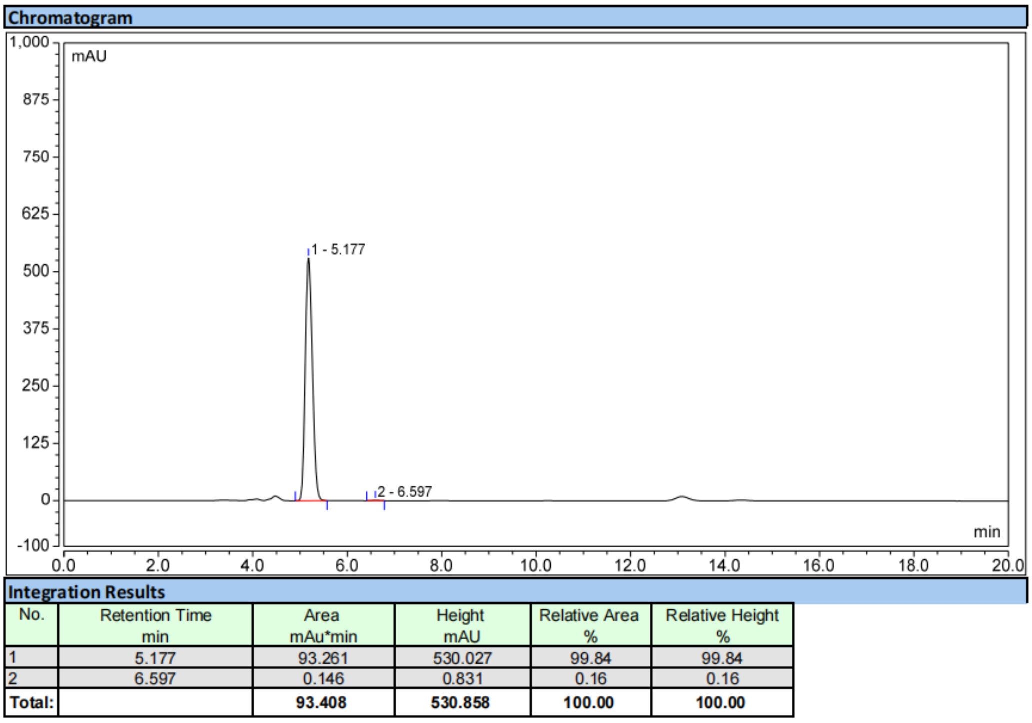

Integration Results

No.	Retention Time min	Area mAu*min	Height mAU	Relative Area %	Relative Height %
1	5.177	93.261	530.027	99.84	99.84
2	6.597	0.146	0.831	0.16	0.16
**Total:**		**93.408**	**530.858**	**100.00**	**100.00**

Chromatogram

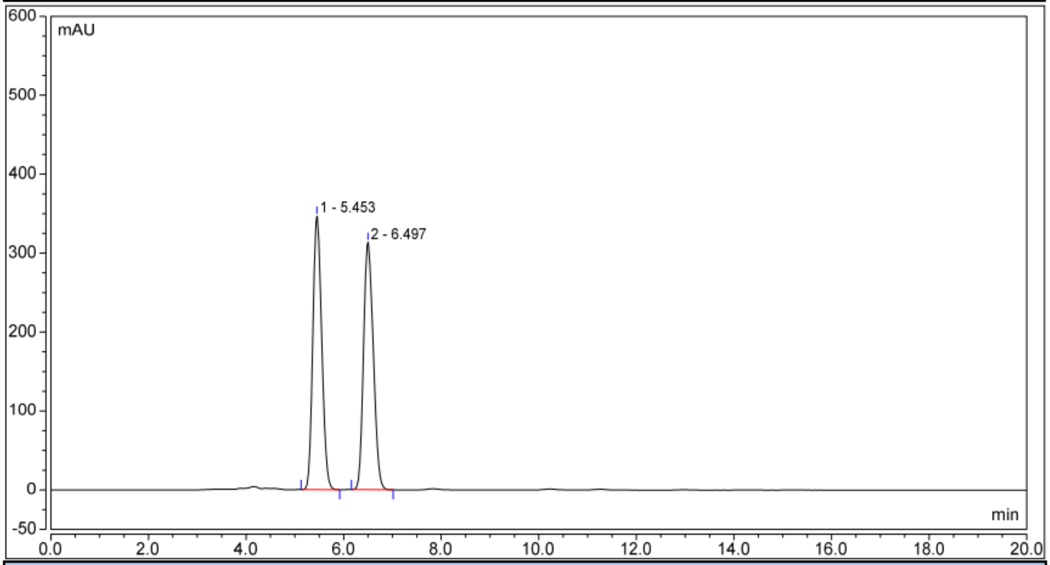

Integration Results

No.	Retention Time min	Area mAu*min	Height mAU	Relative Area %	Relative Height %
1	5.453	72.670	346.358	49.97	52.52
2	6.497	72.768	313.139	50.03	47.48
**Total:**		**145.437**	**659.498**	**100.00**	**100.00**

methyl (S)-2-(1-benzyl-3-(4-bromobenzyl)-4,7-dichloro-2-oxoindolin-3-yl)acetate (**5c**)

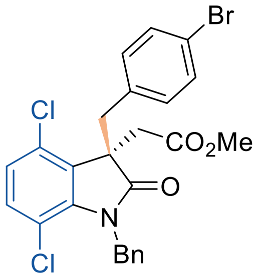

Chromatogram

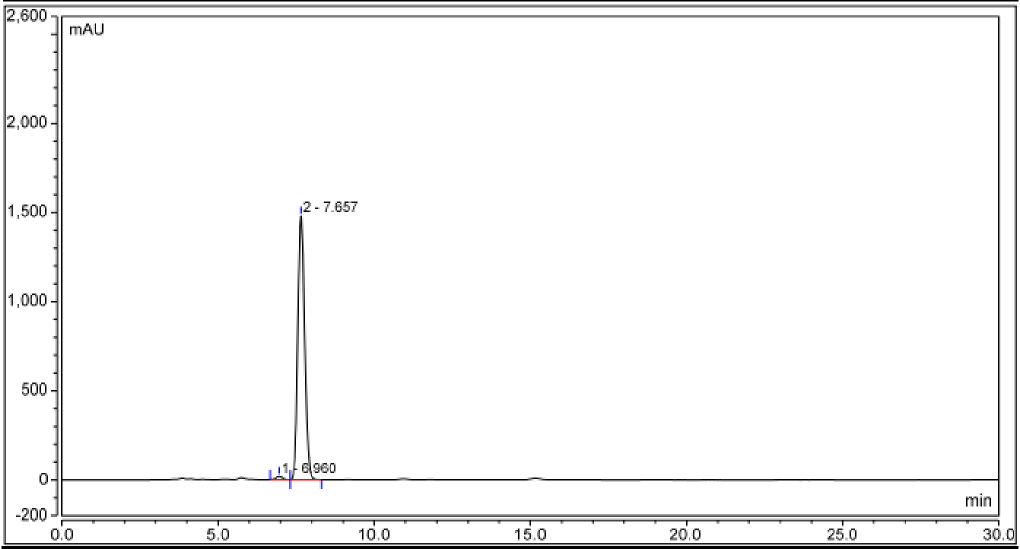

Integration Results

No.	Retention Time min	Area mAu*min	Height mAU	Relative Area %	Relative Height %
1	6.960	4.260	18.575	1.14	1.24
2	7.657	368.811	1478.489	98.86	98.76
**Total:**		**373.071**	**1497.064**	**100.00**	**100.00**

Chromatogram

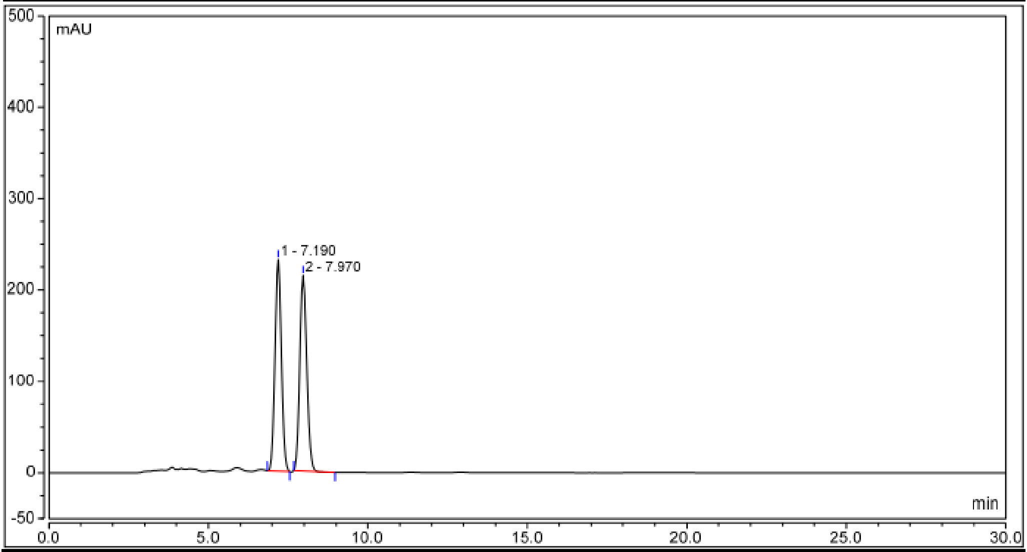

Integration Results

No.	Retention Time min	Area mAu*min	Height mAU	Relative Area %	Relative Height %
1	7.190	53.273	231.266	50.11	51.95
2	7.970	53.048	213.891	49.89	48.05
**Total:**		**106.321**	**445.156**	**100.00**	**100.00**

methyl (S)-2-(1-benzyl-4,7-dichloro-3-(4-iodobenzyl)-2-oxoindolin-3-yl)acetate (**5d**)

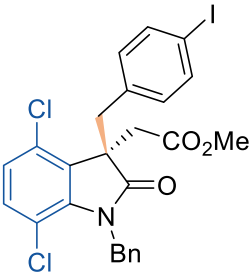

Chromatogram

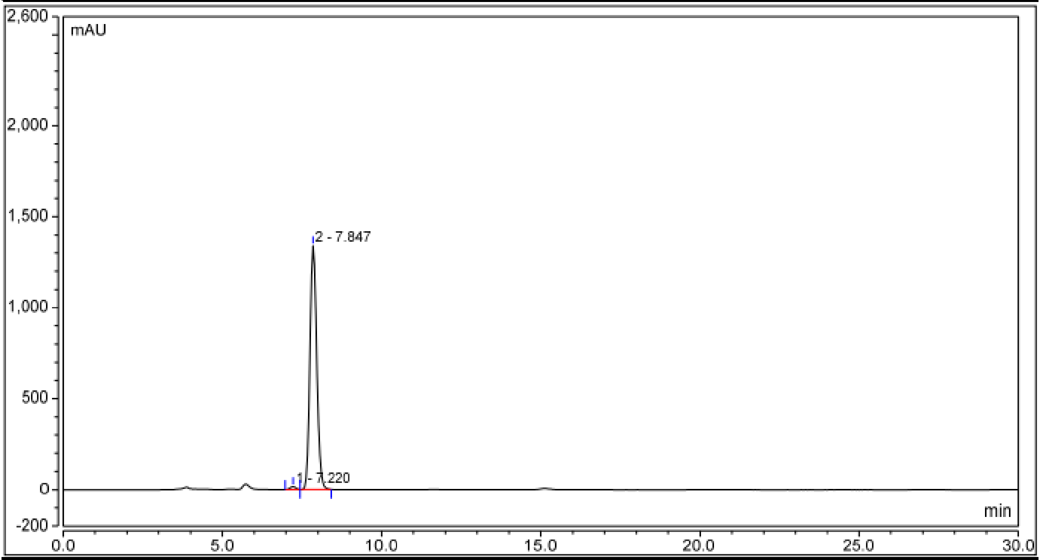

Integration Results

No.	Retention Time min	Area mAu*min	Height mAU	Relative Area %	Relative Height %
1	7.220	3.292	15.521	0.99	1.15
2	7.847	327.872	1338.209	99.01	98.85
**Total:**		**331.164**	**1353.730**	**100.00**	**100.00**

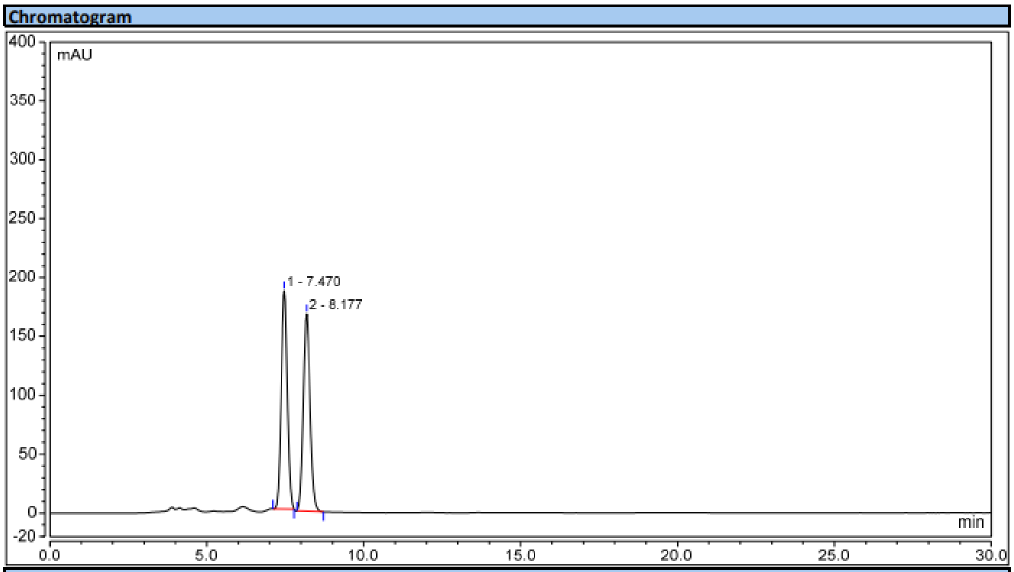

Integration Results

No.	Retention Time min	Area mAu*min	Height mAU	Relative Area %	Relative Height %
1	7.470	41.711	185.636	49.66	52.53
2	8.177	42.284	167.770	50.34	47.47
**Total:**		**83.995**	**353.407**	**100.00**	**100.00**

methyl (S)-2-(1-benzyl-3-(4-cyanobenzyl)-4,6-difluoro-2-oxoindolin-3-yl)acetate (**5e**)

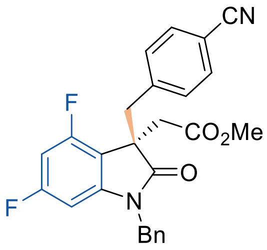

Chromatogram

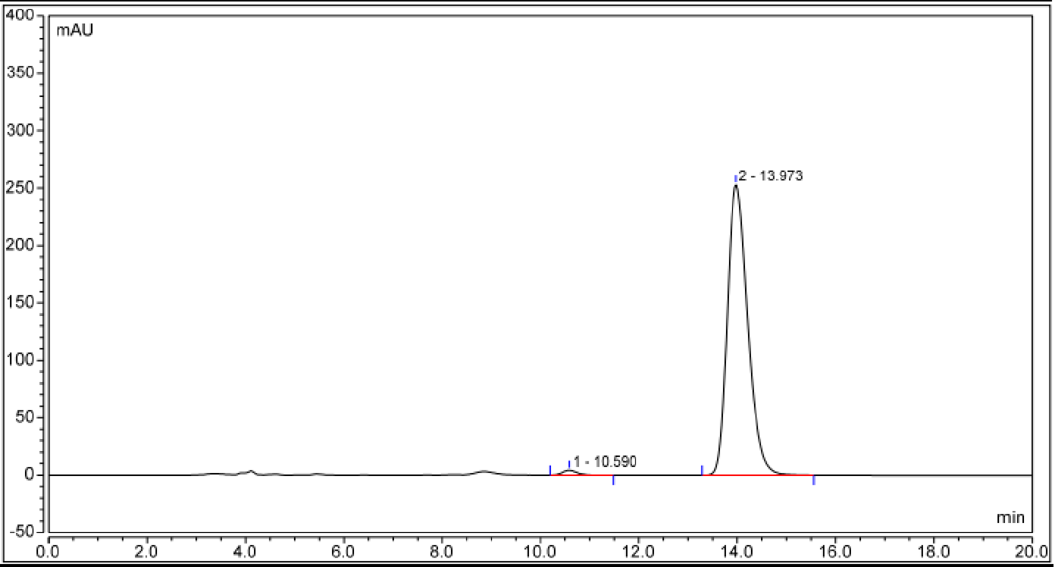

Integration Results

No.	Retention Time min	Area mAu*min	Height mAU	Relative Area %	Relative Height %
1	10.590	1.444	4.202	1.18	1.63
2	13.973	121.293	253.040	98.82	98.37
**Total:**		**122.737**	**257.242**	**100.00**	**100.00**

Chromatogram

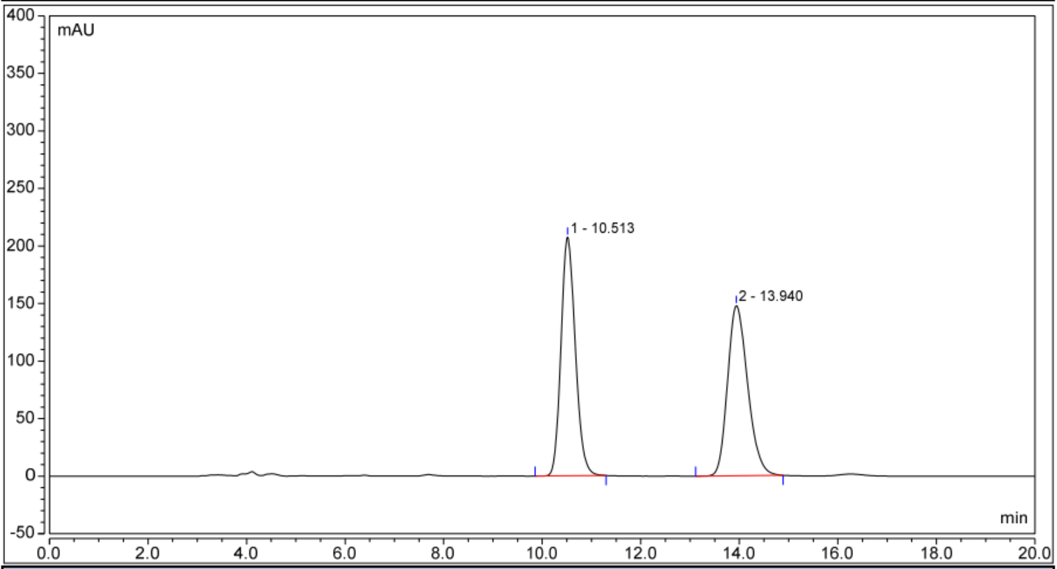

Integration Results

No.	Retention Time min	Area mAu*min	Height mAU	Relative Area %	Relative Height %
1	10.513	69.988	207.482	50.01	58.37
2	13.940	69.967	147.980	49.99	41.63
**Total:**		**139.956**	**355.462**	**100.00**	**100.00**

methyl (S)-2-(1-benzyl-4,7-dichloro-3-(4-nitrobenzyl)-2-oxoindolin-3-yl)acetate (**5f**)

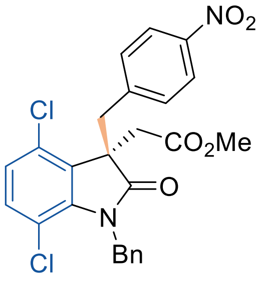

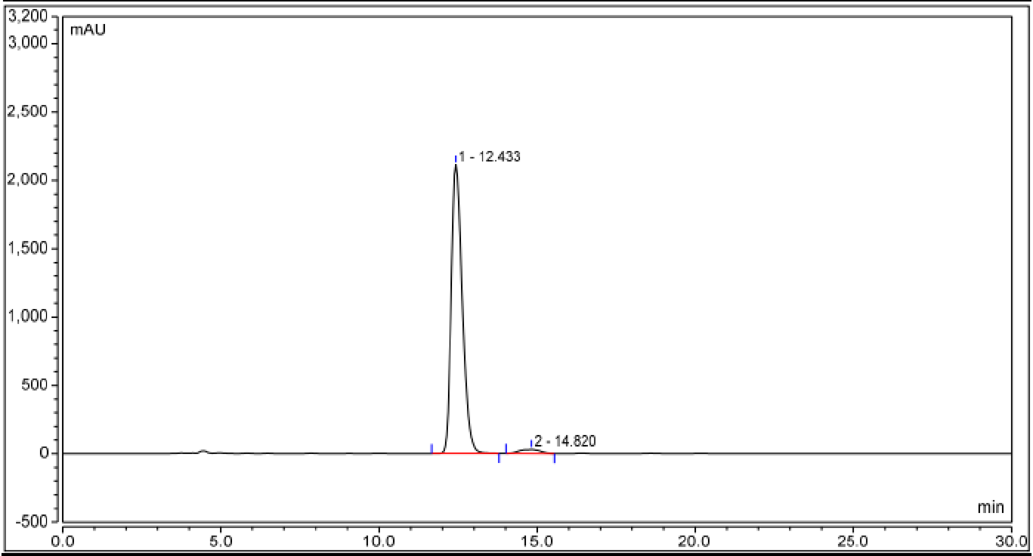

Integration Results

No.	Retention Time<br>min	Area<br>mAu*min	Height<br>mAU	Relative Area<br>%	Relative Height<br>%
1	12.433	855.235	2112.439	97.61	98.71
2	14.820	20.950	27.567	2.39	1.29
**Total:**		**876.184**	**2140.006**	**100.00**	**100.00**

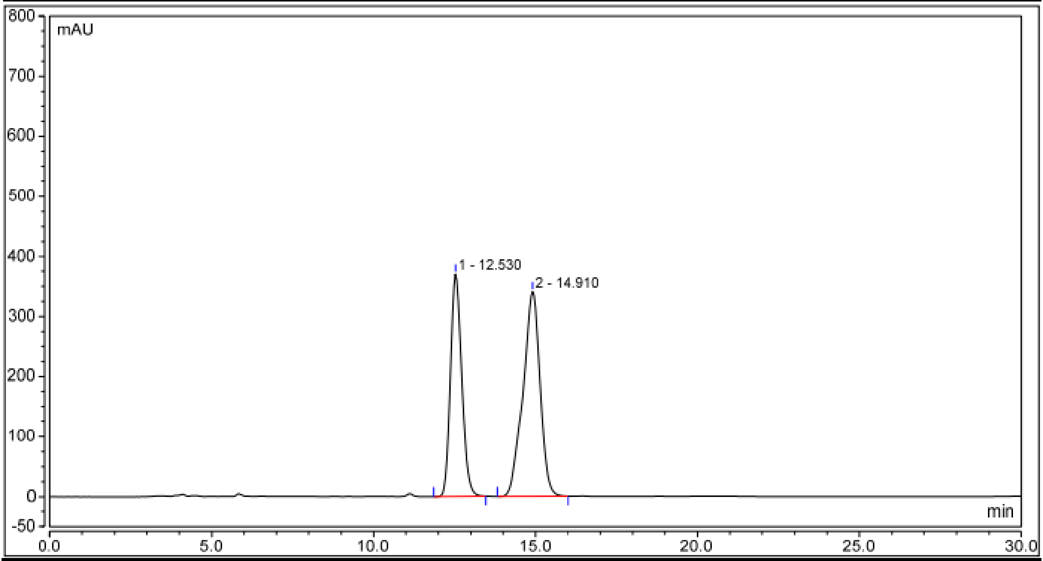

Integration Results

No.	Retention Time<br>min	Area<br>mAu*min	Height<br>mAU	Relative Area<br>%	Relative Height<br>%
1	12.530	148.441	369.802	41.84	52.05
2	14.910	206.303	340.634	58.16	47.95
**Total:**		**354.743**	**710.436**	**100.00**	**100.00**

methyl (S)-4-((1-benzyl-4,7-dichloro-3-(2-methoxy-2-oxoethyl)-2-oxoindolin-3-yl)methyl)benzoate (**5g**)

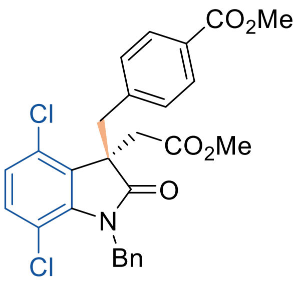

Chromatogram

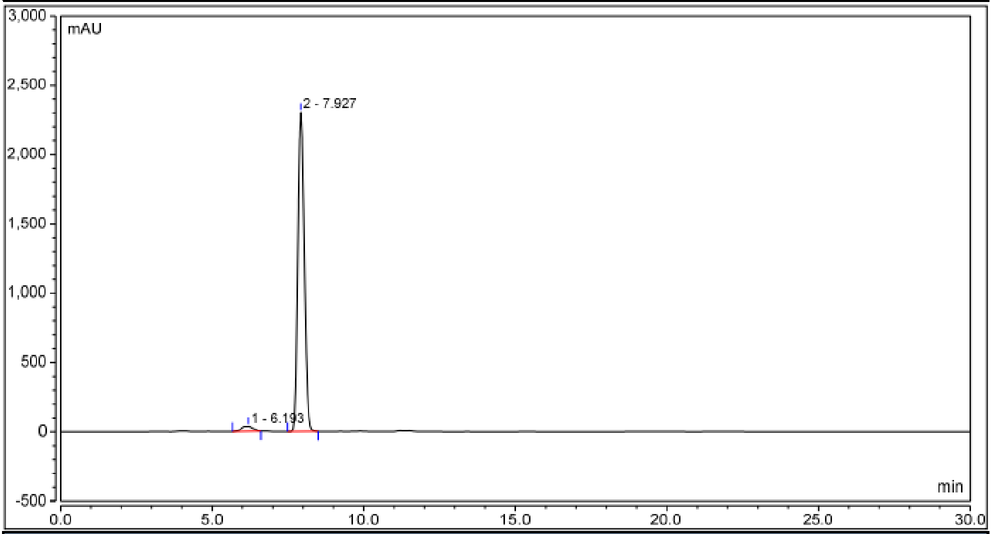

Integration Results

No.	Retention Time min	Area mAu*min	Height mAU	Relative Area %	Relative Height %
1	6.193	15.790	35.517	2.69	1.52
2	7.927	570.781	2303.511	97.31	98.48
**Total:**		**586.571**	**2339.028**	**100.00**	**100.00**

Chromatogram

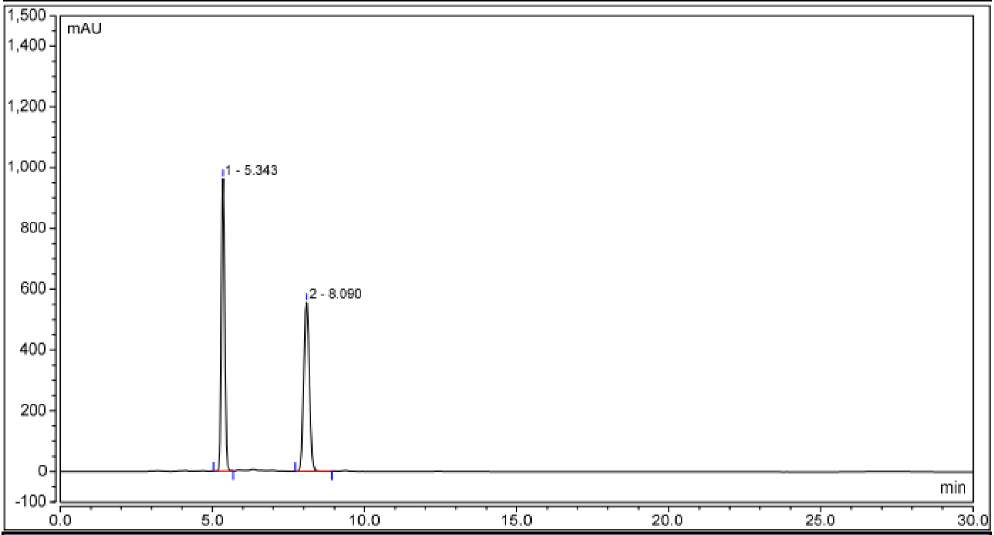

Integration Results

No.	Retention Time min	Area mAu*min	Height mAU	Relative Area %	Relative Height %
1	5.343	115.578	962.338	49.88	63.39
2	8.090	116.125	555.828	50.12	36.61
**Total:**		**231.702**	**1518.167**	**100.00**	**100.00**

methyl (S)-2-(3-(4-acetylbenzyl)-1-benzyl-4,7-dichloro-2-oxoindolin-3-yl)acetate (**5h**)

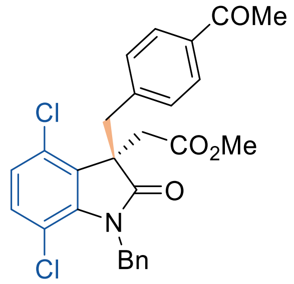

Chromatogram

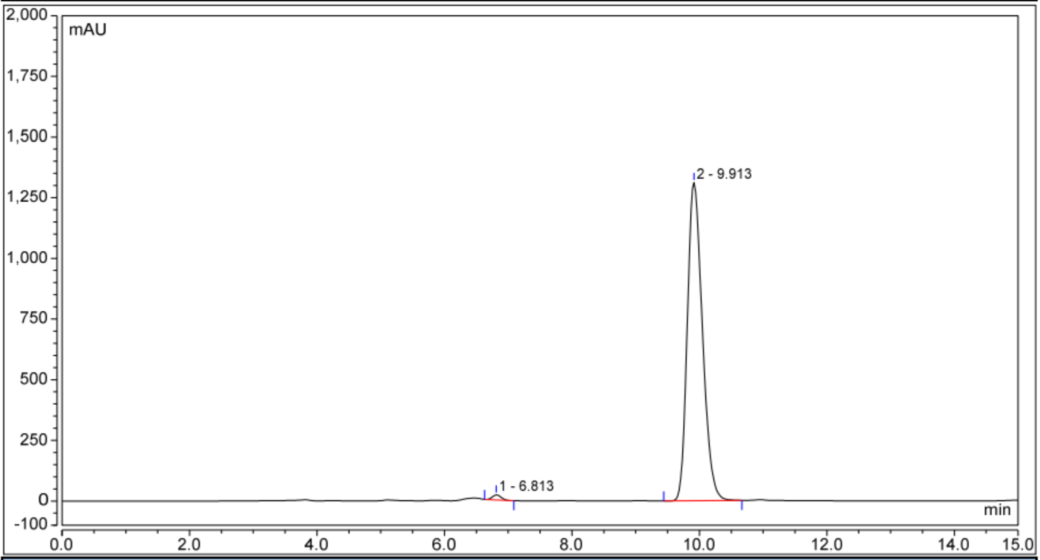

Integration Results

No.	Retention Time<br>min	Area<br>mAu*min	Height<br>mAU	Relative Area<br>%	Relative Height<br>%
1	6.813	3.295	20.350	0.91	1.53
2	9.913	360.185	1311.415	99.09	98.47
**Total:**		**363.479**	**1331.765**	**100.00**	**100.00**

Chromatogram

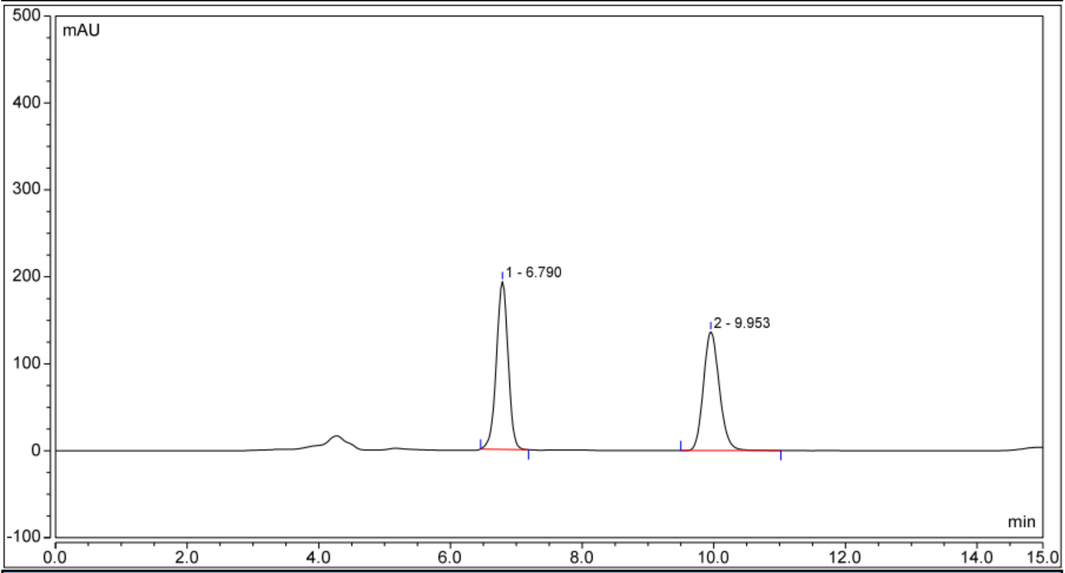

Integration Results

No.	Retention Time<br>min	Area<br>mAu*min	Height<br>mAU	Relative Area<br>%	Relative Height<br>%
1	6.790	39.684	192.805	50.77	58.50
2	9.953	38.480	136.802	49.23	41.50
**Total:**		**78.164**	**329.608**	**100.00**	**100.00**

methyl (S)-2-(1-benzyl-4,7-dichloro-3-(4-(methylsulfonyl)benzyl)-2-oxoindolin-3-yl)acetate (**5i**)

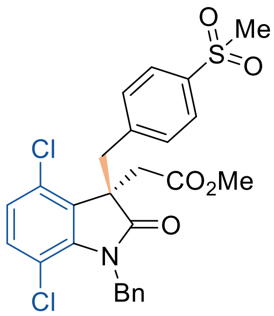

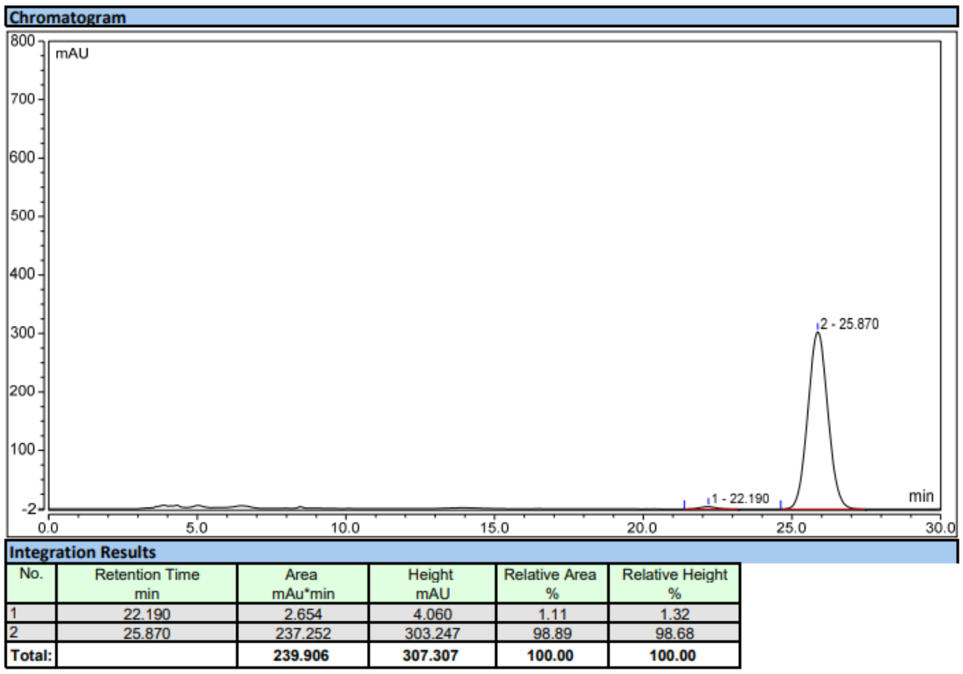

Integration Results

No.	Retention Time min	Area mAu*min	Height mAU	Relative Area %	Relative Height %
1	22.190	2.654	4.060	1.11	1.32
2	25.870	237.252	303.247	98.89	98.68
**Total:**		**239.906**	**307.307**	**100.00**	**100.00**

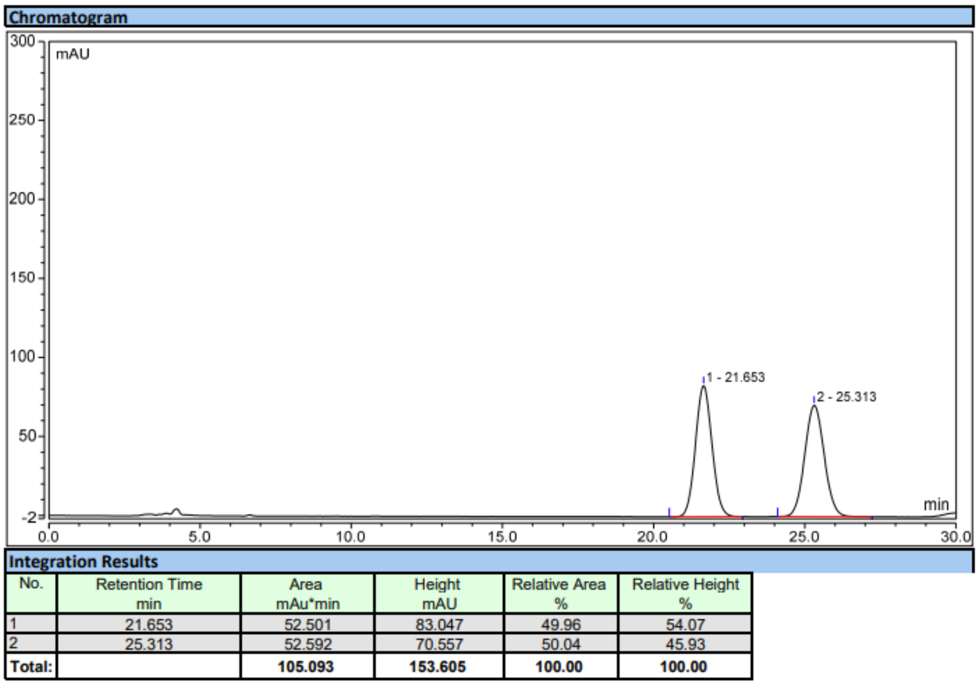

Integration Results

No.	Retention Time min	Area mAu*min	Height mAU	Relative Area %	Relative Height %
1	21.653	52.501	83.047	49.96	54.07
2	25.313	52.592	70.557	50.04	45.93
**Total:**		**105.093**	**153.605**	**100.00**	**100.00**

methyl (S)-2-(3-([1,1'-biphenyl]-4-ylmethyl)-1-benzyl-4,7-dichloro-2-oxoindolin-3-yl)acetate (**5j**)

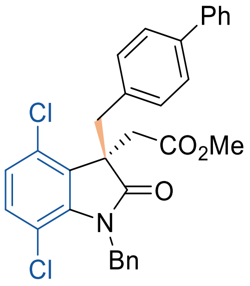

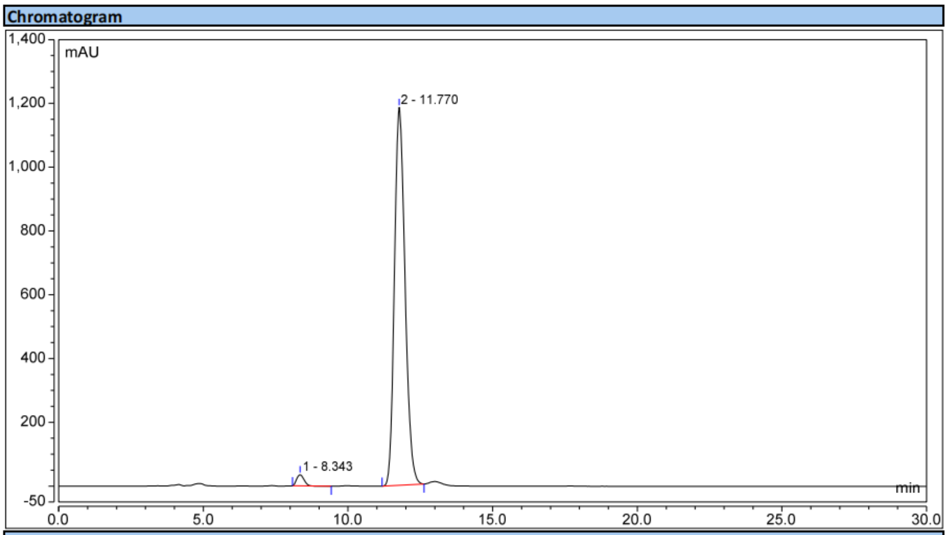

Integration Results

No.	Retention Time min	Area mAu*min	Height mAU	Relative Area %	Relative Height %
1	8.343	10.046	34.936	1.96	2.86
2	11.770	501.786	1184.483	98.04	97.14
**Total:**		**511.832**	**1219.419**	**100.00**	**100.00**

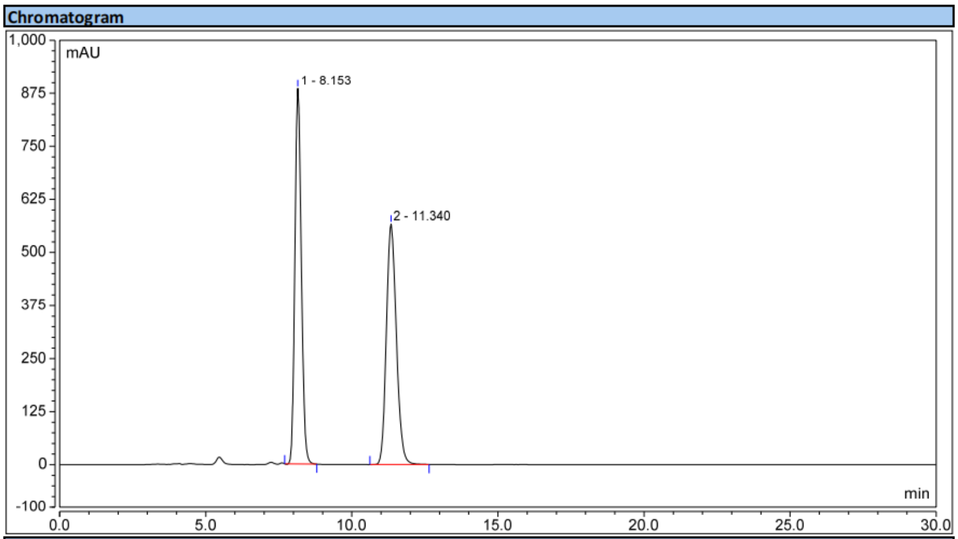

Integration Results

No.	Retention Time min	Area mAu*min	Height mAU	Relative Area %	Relative Height %
1	8.153	224.423	884.550	50.00	60.95
2	11.340	224.413	566.690	50.00	39.05
**Total:**		**448.835**	**1451.240**	**100.00**	**100.00**

methyl (S)-2-(1-benzyl-4,7-dichloro-3-(4-methylbenzyl)-2-oxoindolin-3-yl)acetate (**5k**)

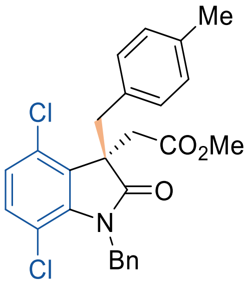

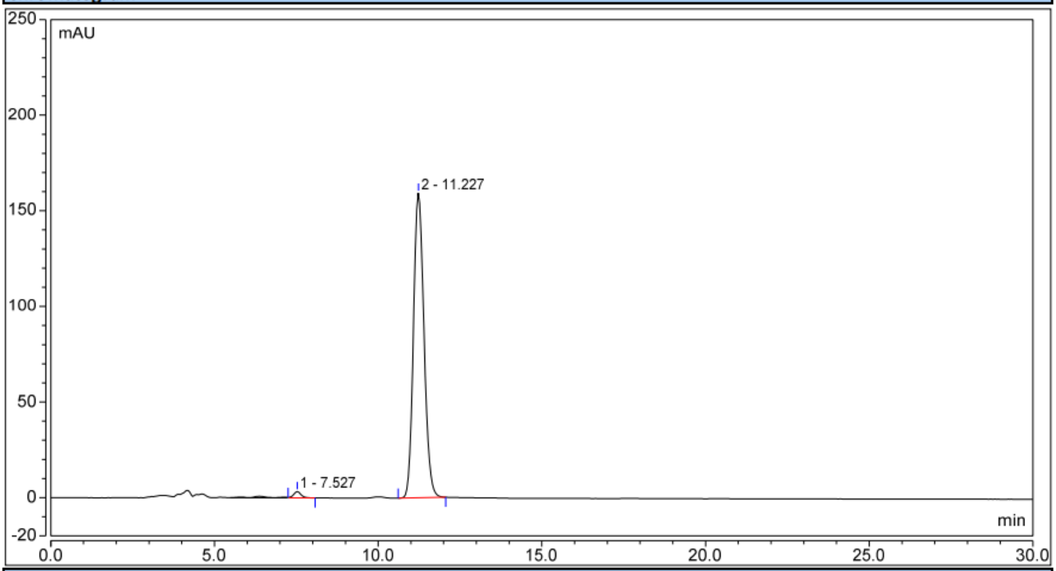

Integration Results

No.	Retention Time<br>min	Area<br>mAu*min	Height<br>mAU	Relative Area<br>%	Relative Height<br>%
1	7.527	0.872	3.307	1.45	2.03
2	11.227	59.315	159.338	98.55	97.97
**Total:**		**60.186**	**162.645**	**100.00**	**100.00**

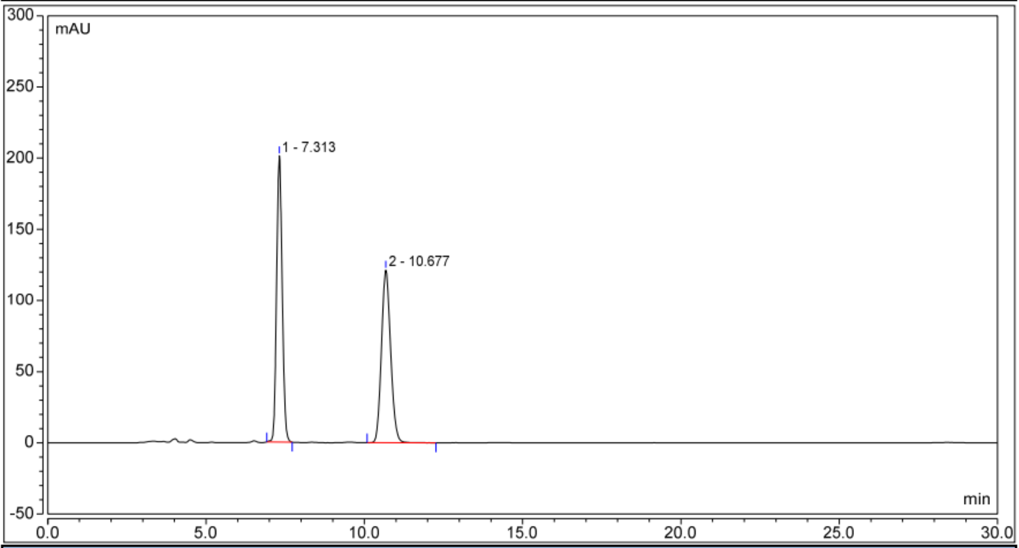

Integration Results

No.	Retention Time<br>min	Area<br>mAu*min	Height<br>mAU	Relative Area<br>%	Relative Height<br>%
1	7.313	40.720	201.167	49.96	62.40
2	10.677	40.781	121.227	50.04	37.60
**Total:**		**81.501**	**322.394**	**100.00**	**100.00**

methyl (S)-2-(1-benzyl-4,6-difluoro-3-(4-methoxybenzyl)-2-oxoindolin-3-yl)acetate (**5l**)

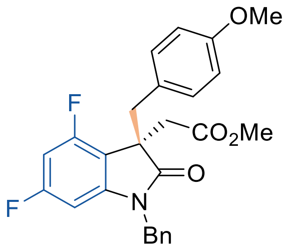

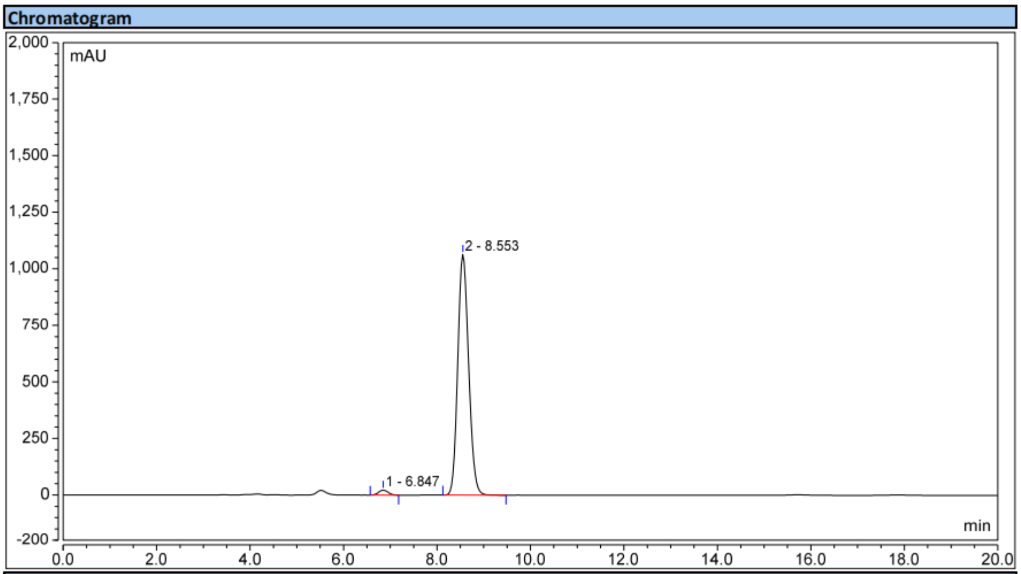

Integration Results

No.	Retention Time min	Area mAu*min	Height mAU	Relative Area %	Relative Height %
1	6.847	5.150	21.780	1.76	2.01
2	8.553	287.136	1064.155	98.24	97.99
**Total:**		**292.286**	**1085.935**	**100.00**	**100.00**

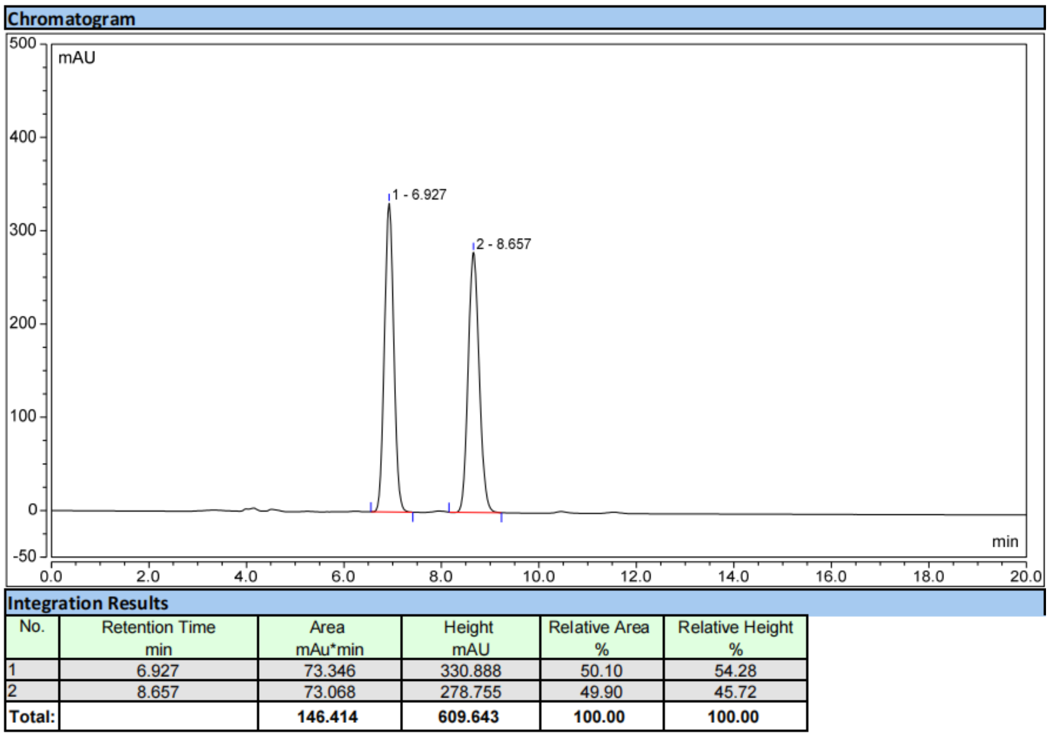

Integration Results

No.	Retention Time min	Area mAu*min	Height mAU	Relative Area %	Relative Height %
1	6.927	73.346	330.888	50.10	54.28
2	8.657	73.068	278.755	49.90	45.72
**Total:**		**146.414**	**609.643**	**100.00**	**100.00**

methyl (S)-2-(1-benzyl-4,7-dichloro-3-(4-(methylthio)benzyl)-2-oxoindolin-3-yl)acetate (**5m**)

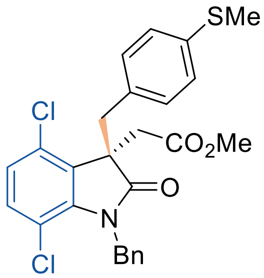

Chromatogram

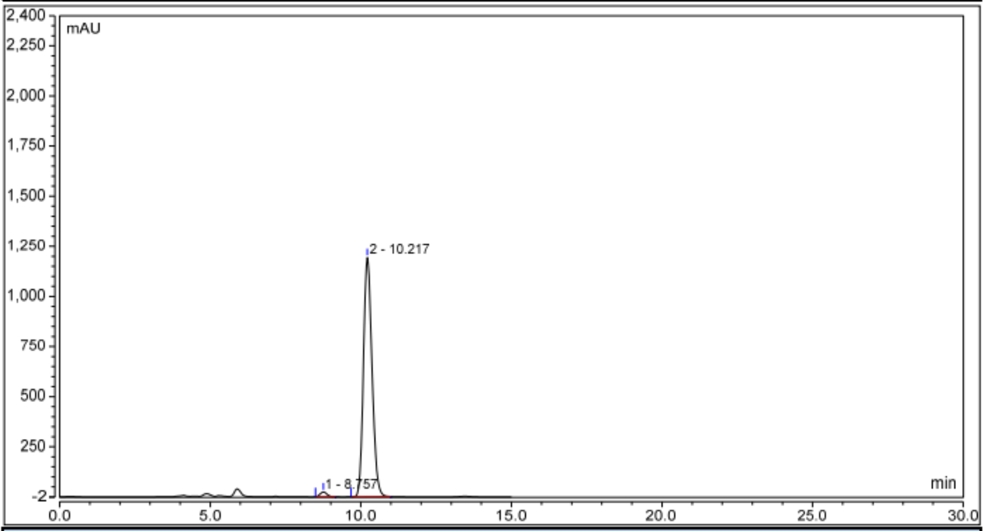

Integration Results

No.	Retention Time min	Area mAu*min	Height mAU	Relative Area %	Relative Height %
1	8.757	6.242	24.191	1.56	1.99
2	10.217	392.873	1192.316	98.44	98.01
**Total:**		**399.115**	**1216.507**	**100.00**	**100.00**

Chromatogram

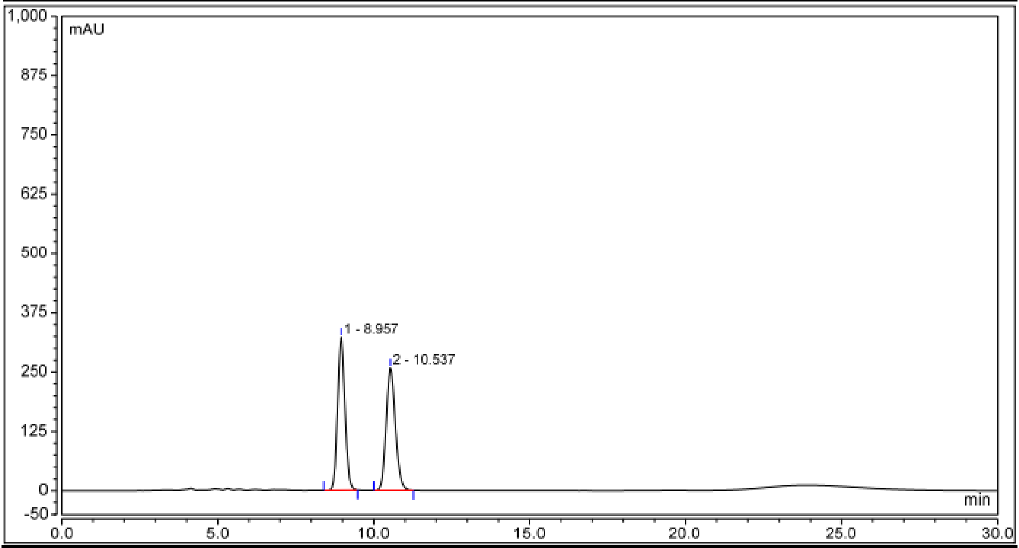

Integration Results

No.	Retention Time min	Area mAu*min	Height mAU	Relative Area %	Relative Height %
1	8.957	89.504	321.879	49.96	55.52
2	10.537	89.636	257.854	50.04	44.48
**Total:**		**179.140**	**579.733**	**100.00**	**100.00**

methyl (S)-2-(1-benzyl-4,6-difluoro-2-oxo-3-(3-(trifluoromethyl)benzyl)indolin-3-yl)acetate (**5n**)

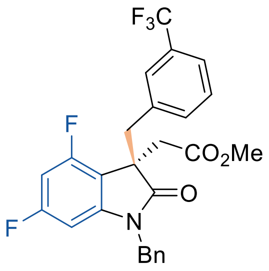

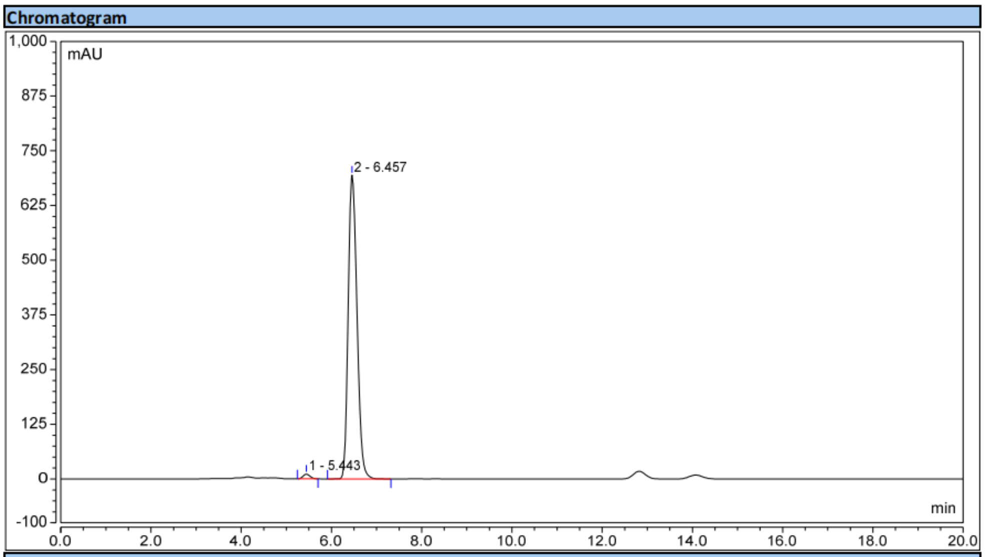

Integration Results

No.	Retention Time min	Area mAu*min	Height mAU	Relative Area %	Relative Height %
1	5.443	1.935	10.768	1.22	1.53
2	6.457	157.332	694.439	98.78	98.47
**Total:**		**159.267**	**705.207**	**100.00**	**100.00**

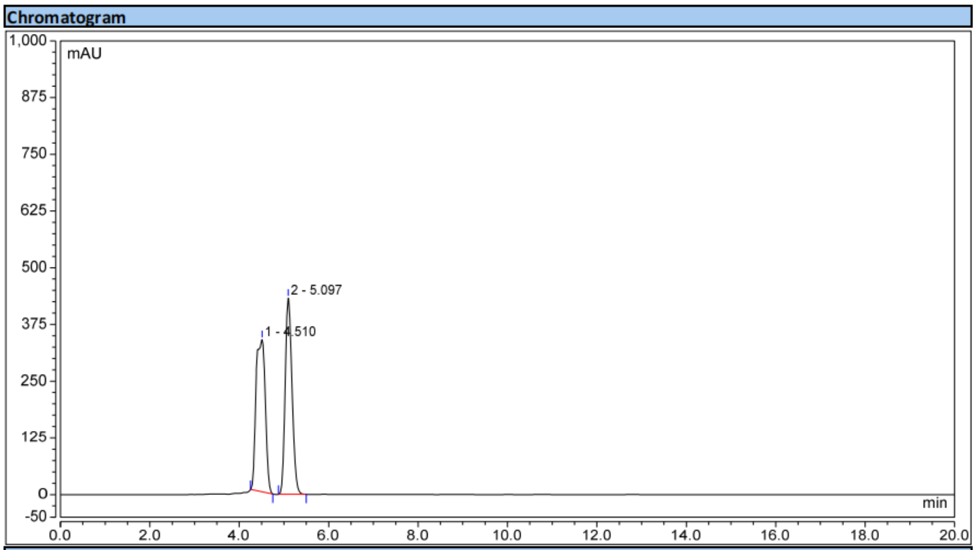

Integration Results

No.	Retention Time min	Area mAu*min	Height mAU	Relative Area %	Relative Height %
1	4.510	80.216	335.349	51.15	43.69
2	5.097	76.619	432.250	48.85	56.31
**Total:**		**156.835**	**767.599**	**100.00**	**100.00**

methyl (S)-2-(1-benzyl-4,7-dichloro-2-oxo-3-((perfluorophenyl)methyl)indolin-3-yl)acetate (**5o**)

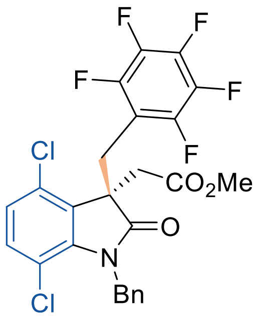

Chromatogram

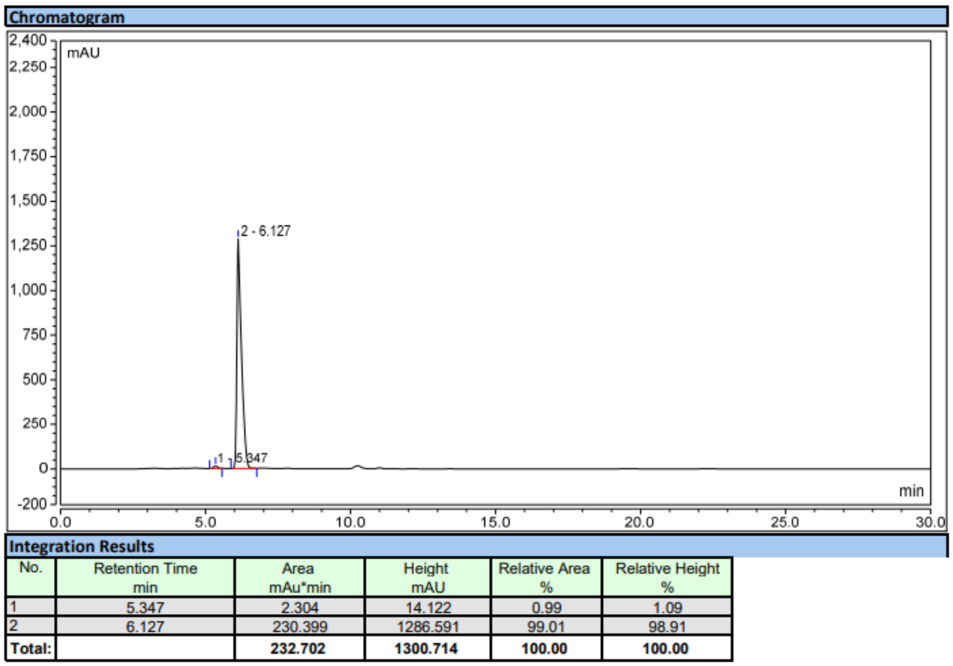

Integration Results

No.	Retention Time min	Area mAu*min	Height mAU	Relative Area %	Relative Height %
1	5.347	2.304	14.122	0.99	1.09
2	6.127	230.399	1286.591	99.01	98.91
**Total:**		**232.702**	**1300.714**	**100.00**	**100.00**

Chromatogram

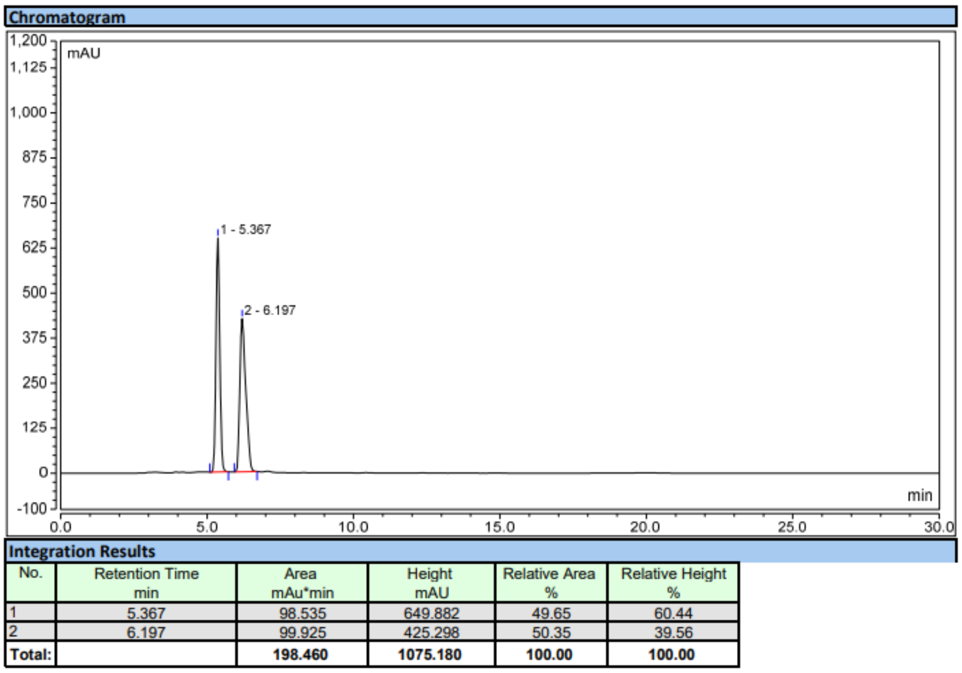

Integration Results

No.	Retention Time min	Area mAu*min	Height mAU	Relative Area %	Relative Height %
1	5.367	98.535	649.882	49.65	60.44
2	6.197	99.925	425.298	50.35	39.56
**Total:**		**198.460**	**1075.180**	**100.00**	**100.00**

methyl methyl (S)-2-(1-benzyl-3-((4-bromonaphthalen-1-yl)methyl)-4,6-difluoro-2-oxoindolin-3-yl)acetate (**5p**)

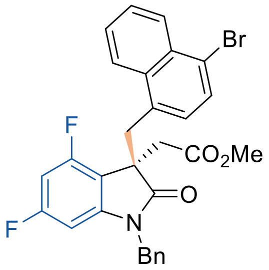

Chromatogram

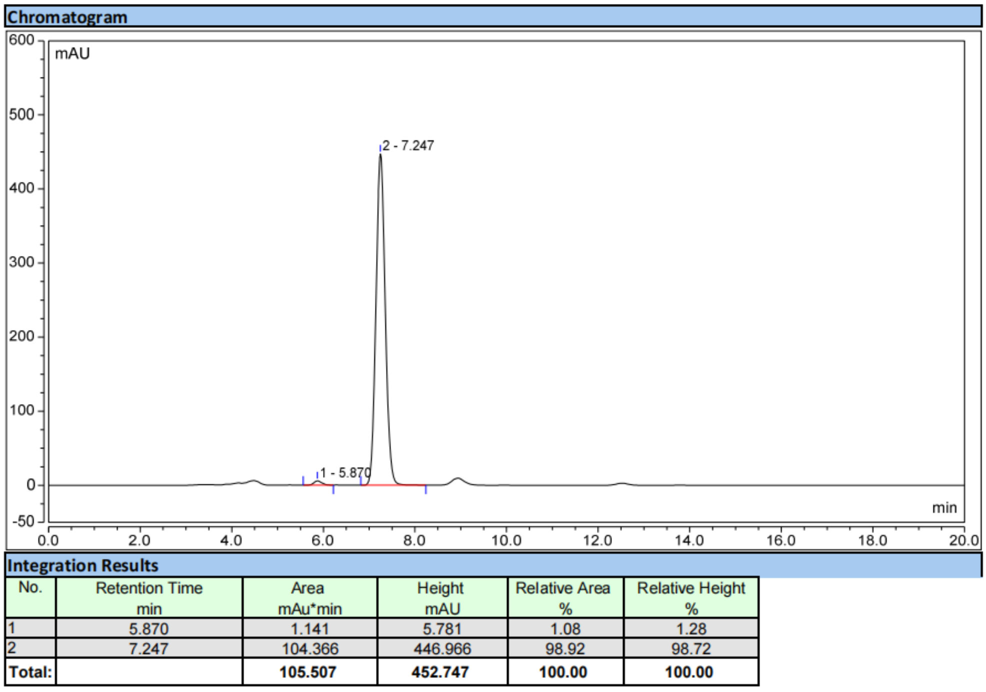

Integration Results

No.	Retention Time min	Area mAu*min	Height mAU	Relative Area %	Relative Height %
1	5.870	1.141	5.781	1.08	1.28
2	7.247	104.366	446.966	98.92	98.72
**Total:**		**105.507**	**452.747**	**100.00**	**100.00**

Chromatogram

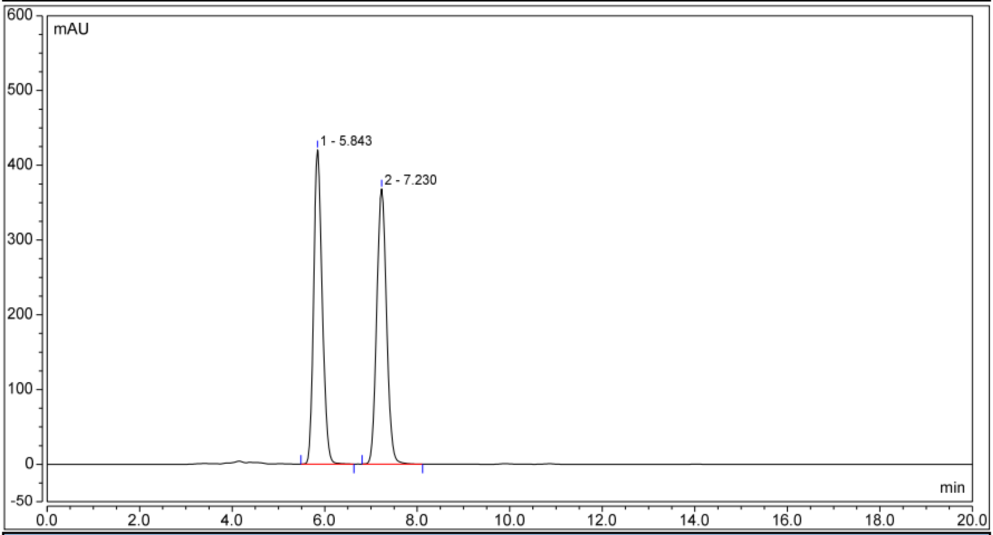

Integration Results

No.	Retention Time min	Area mAu*min	Height mAU	Relative Area %	Relative Height %
1	5.843	89.975	420.457	49.92	53.32
2	7.230	90.268	368.106	50.08	46.68
**Total:**		**180.244**	**788.562**	**100.00**	**100.00**

methyl (S)-2-((1-benzyl-4,7-dichloro-3-(2-methoxy-2-oxoethyl)-2-oxoindolin-3-yl)methyl)furan-3-carboxylate (**5q**)

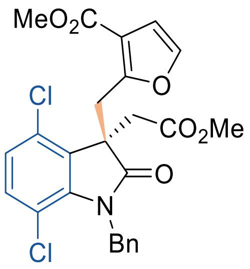

Chromatogram

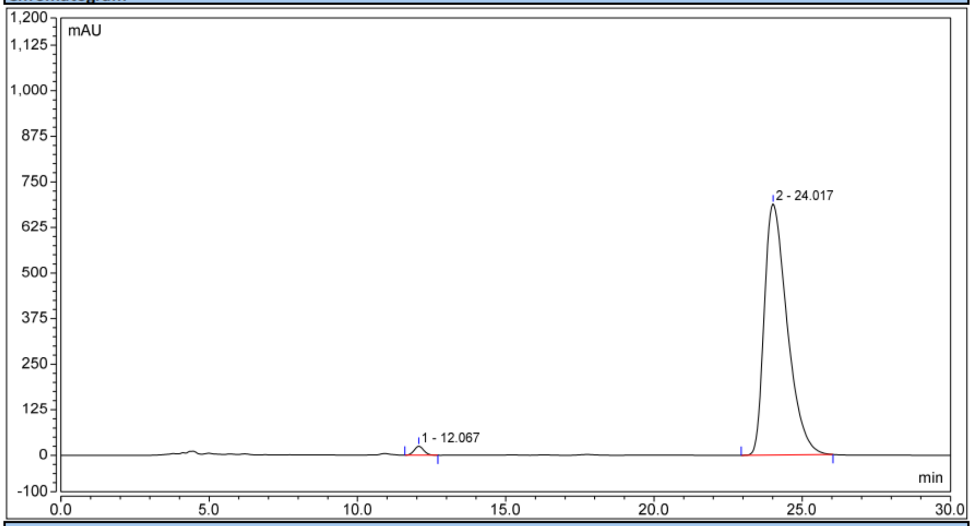

Integration Results

No.	Retention Time min	Area mAu*min	Height mAU	Relative Area %	Relative Height %
1	12.067	9.278	24.429	1.48	3.43
2	24.017	615.703	688.795	98.52	96.57
**Total:**		**624.981**	**713.224**	**100.00**	**100.00**

Chromatogram

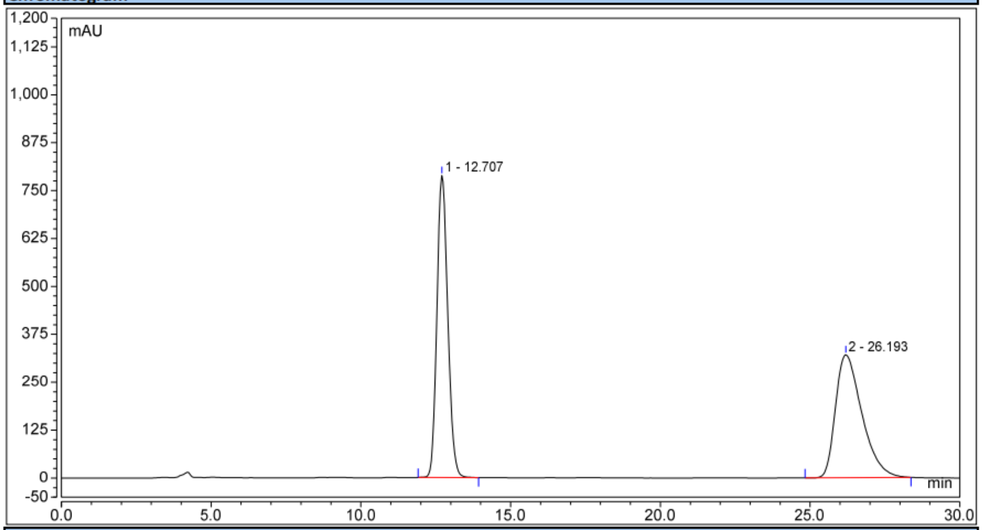

Integration Results

No.	Retention Time min	Area mAu*min	Height mAU	Relative Area %	Relative Height %
1	12.707	331.024	788.050	50.28	71.06
2	26.193	327.358	320.928	49.72	28.94
**Total:**		**658.382**	**1108.978**	**100.00**	**100.00**

methyl (S)-2-(1-benzyl-4,7-dichloro-2-oxo-3-(thiophen-3-ylmethyl)indolin-3-yl)acetate (**5r**)

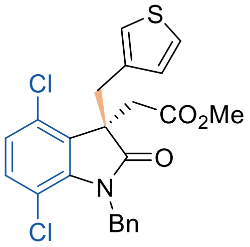

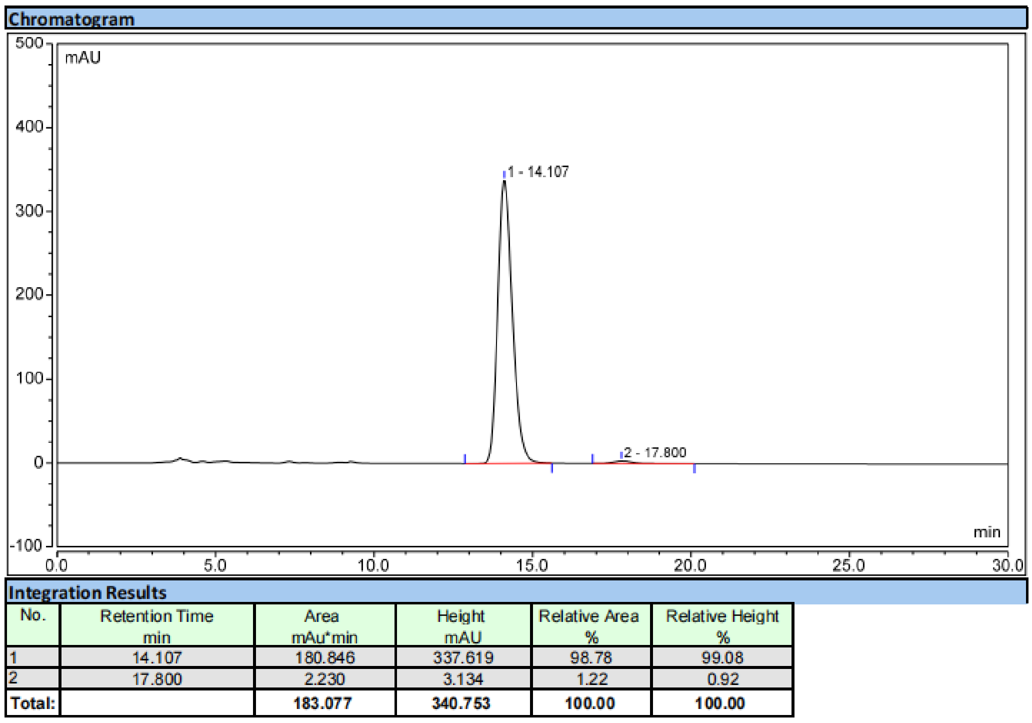

Integration Results

No.	Retention Time min	Area mAu*min	Height mAU	Relative Area %	Relative Height %
1	14.107	180.846	337.619	98.78	99.08
2	17.800	2.230	3.134	1.22	0.92
**Total:**		**183.077**	**340.753**	**100.00**	**100.00**

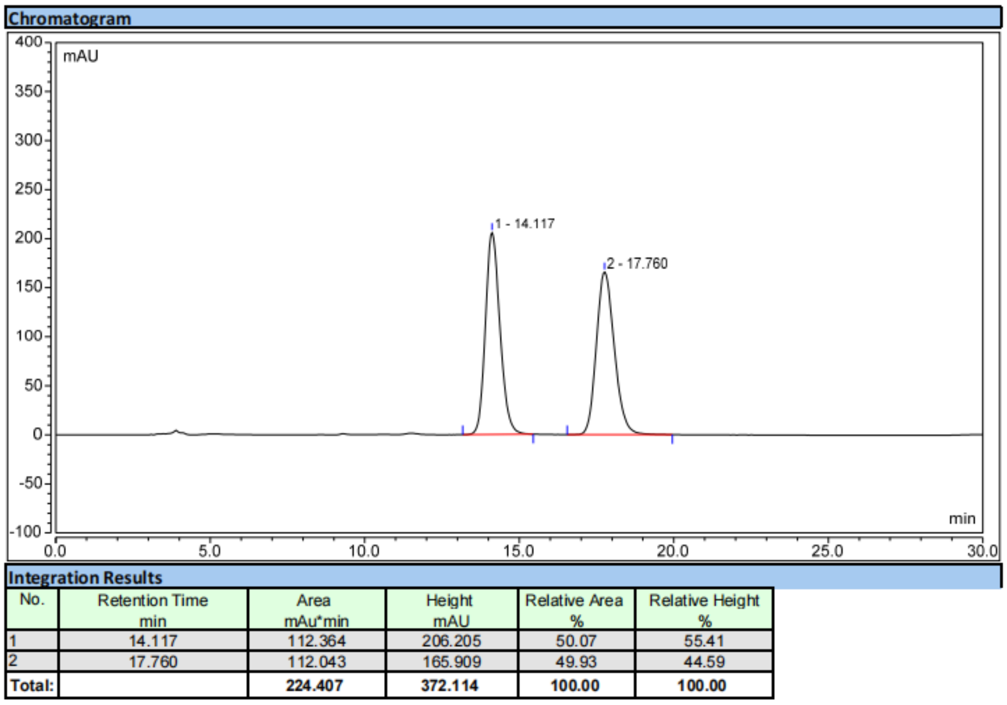

Integration Results

No.	Retention Time min	Area mAu*min	Height mAU	Relative Area %	Relative Height %
1	14.117	112.364	206.205	50.07	55.41
2	17.760	112.043	165.909	49.93	44.59
**Total:**		**224.407**	**372.114**	**100.00**	**100.00**

methyl (R)-2-(1-benzyl-4,7-dichloro-3-((5-cyanothiophen-2-yl)methyl)-2-oxoindolin-3-yl)acetate (**5s**)

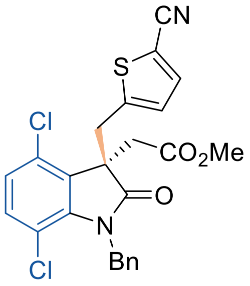

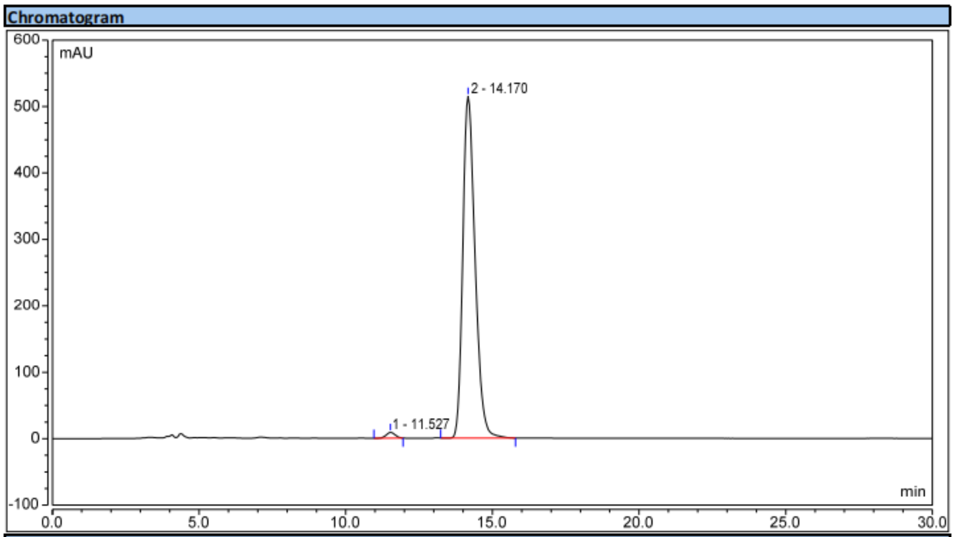

Integration Results

No.	Retention Time<br>min	Area<br>mAu*min	Height<br>mAU	Relative Area<br>%	Relative Height<br>%
1	11.527	3.046	8.693	1.21	1.66
2	14.170	249.574	514.185	98.79	98.34
**Total:**		**252.620**	**522.877**	**100.00**	**100.00**

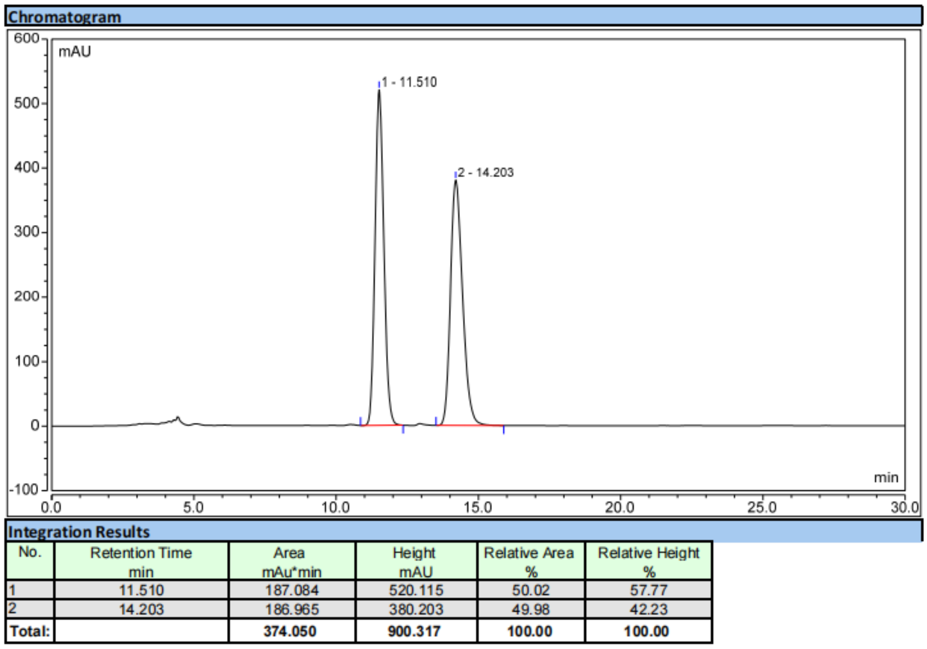

Integration Results

No.	Retention Time<br>min	Area<br>mAu*min	Height<br>mAU	Relative Area<br>%	Relative Height<br>%
1	11.510	187.084	520.115	50.02	57.77
2	14.203	186.965	380.203	49.98	42.23
**Total:**		**374.050**	**900.317**	**100.00**	**100.00**

methyl (S)-2-(3-(benzo[b]thiophen-7-ylmethyl)-1-benzyl-4,7-dichloro-2-oxoindolin-3-yl)acetate (**5t**)

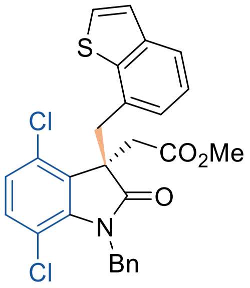

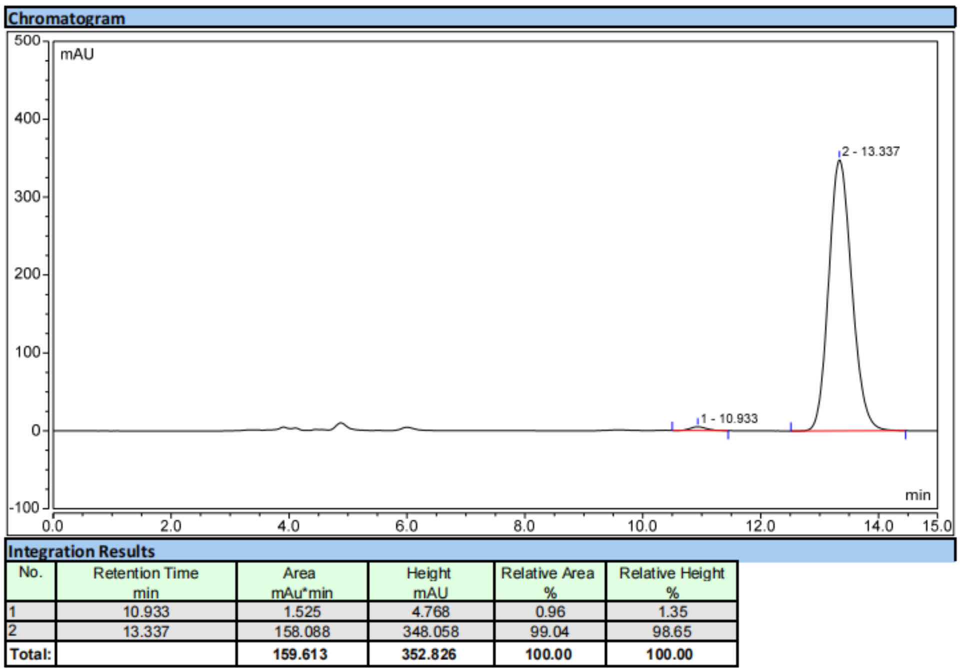

Integration Results

No.	Retention Time min	Area mAu*min	Height mAU	Relative Area %	Relative Height %
1	10.933	1.525	4.768	0.96	1.35
2	13.337	158.088	348.058	99.04	98.65
Total:		159.613	352.826	100.00	100.00

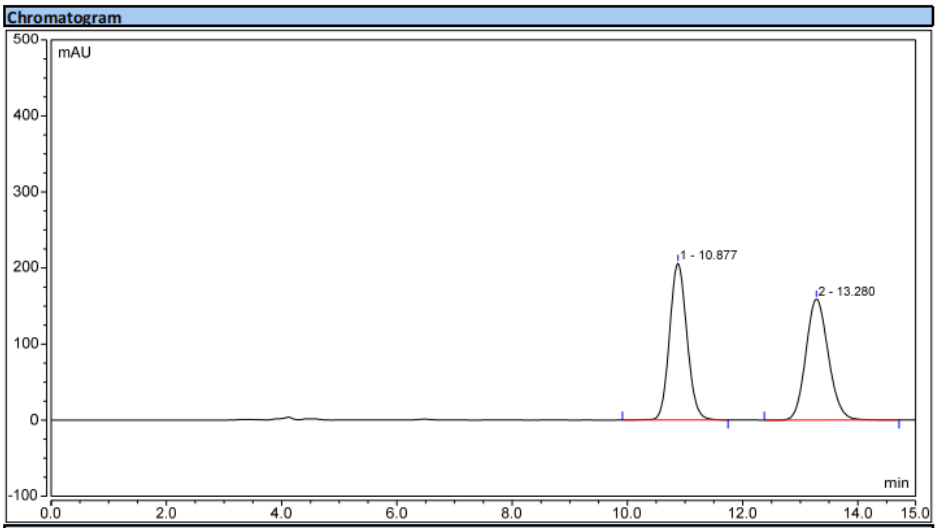

Integration Results

No.	Retention Time min	Area mAu*min	Height mAU	Relative Area %	Relative Height %
1	10.877	71.912	206.184	50.01	56.45
2	13.280	71.887	159.048	49.99	43.55
Total:		143.799	365.232	100.00	100.00

methyl (S)-2-(1-benzyl-4,7-dichloro-3-((2-cyanopyridin-3-yl)methyl)-2-oxoindolin-3-

yl)acetate (**5u**)

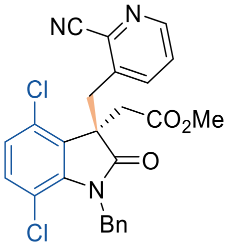

Chromatogram

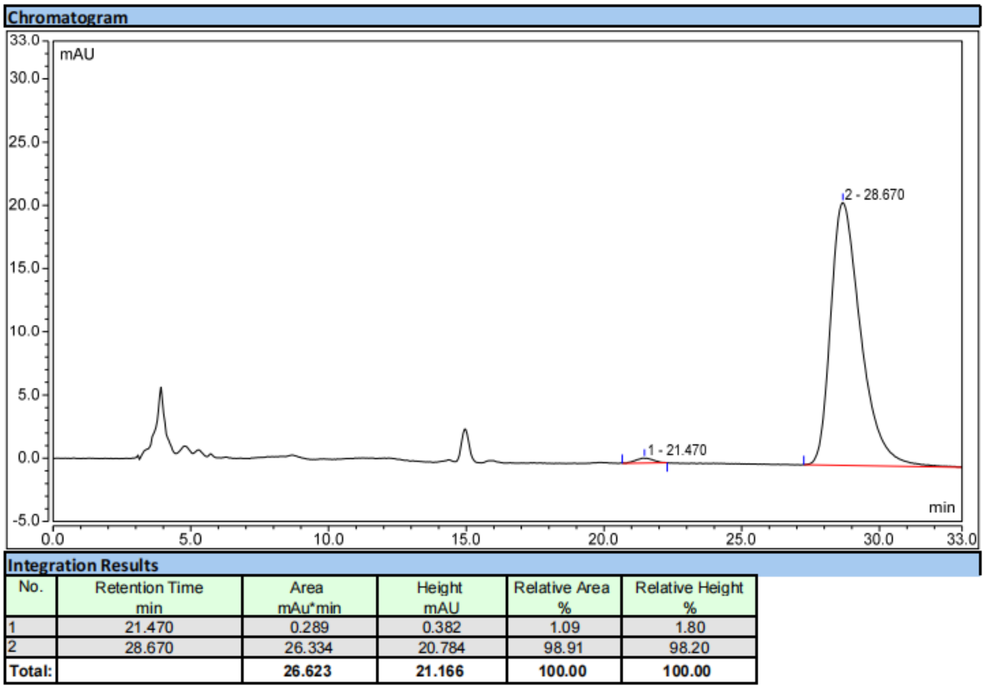

Integration Results

No.	Retention Time min	Area mAu*min	Height mAU	Relative Area %	Relative Height %
1	21.470	0.289	0.382	1.09	1.80
2	28.670	26.334	20.784	98.91	98.20
**Total:**		**26.623**	**21.166**	**100.00**	**100.00**

Chromatogram

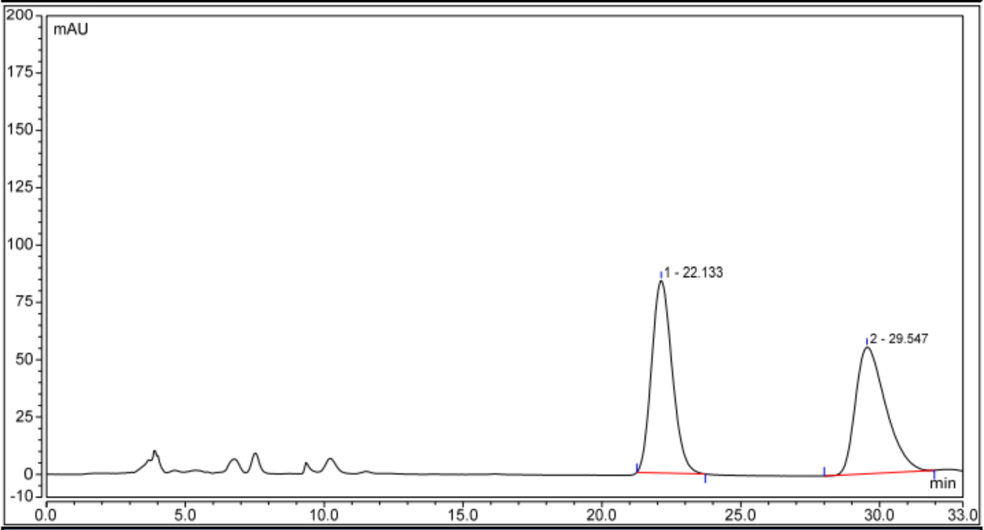

Integration Results

No.	Retention Time min	Area mAu*min	Height mAU	Relative Area %	Relative Height %
1	22.133	71.912	83.938	50.86	60.33
2	29.547	69.486	55.202	49.14	39.67
**Total:**		**141.398**	**139.140**	**100.00**	**100.00**

methyl (S)-2-(1-benzyl-4,7-dichloro-2-oxo-3-(pyrazin-2-ylmethyl)indolin-3-yl)acetate (**5v**)

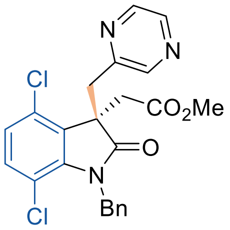

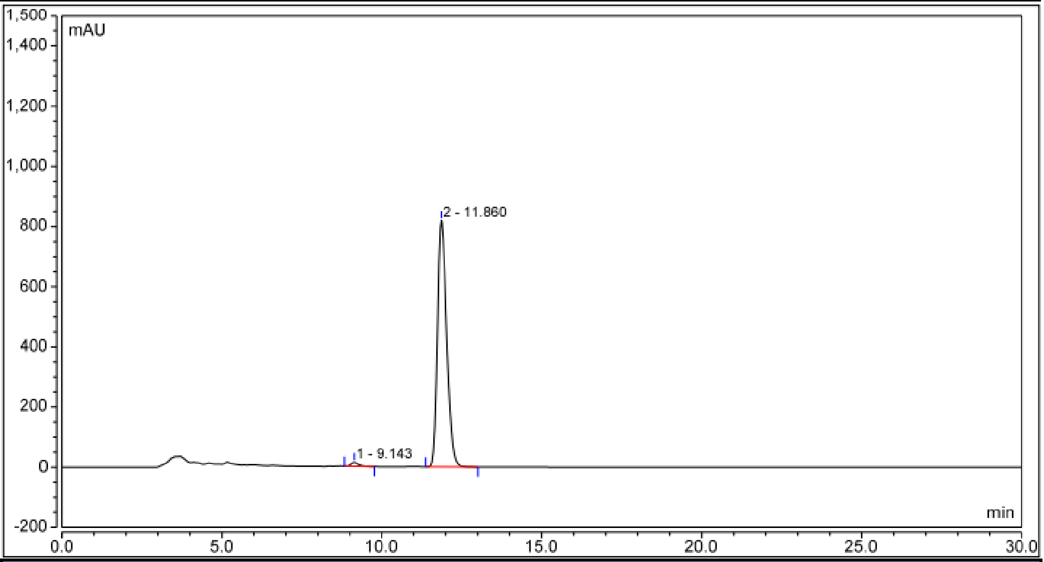

Integration Results

No.	Retention Time min	Area mAu*min	Height mAU	Relative Area %	Relative Height %
1	9.143	3.300	12.497	1.19	1.50
2	11.860	274.360	818.516	98.81	98.50
**Total:**		**277.660**	**831.013**	**100.00**	**100.00**

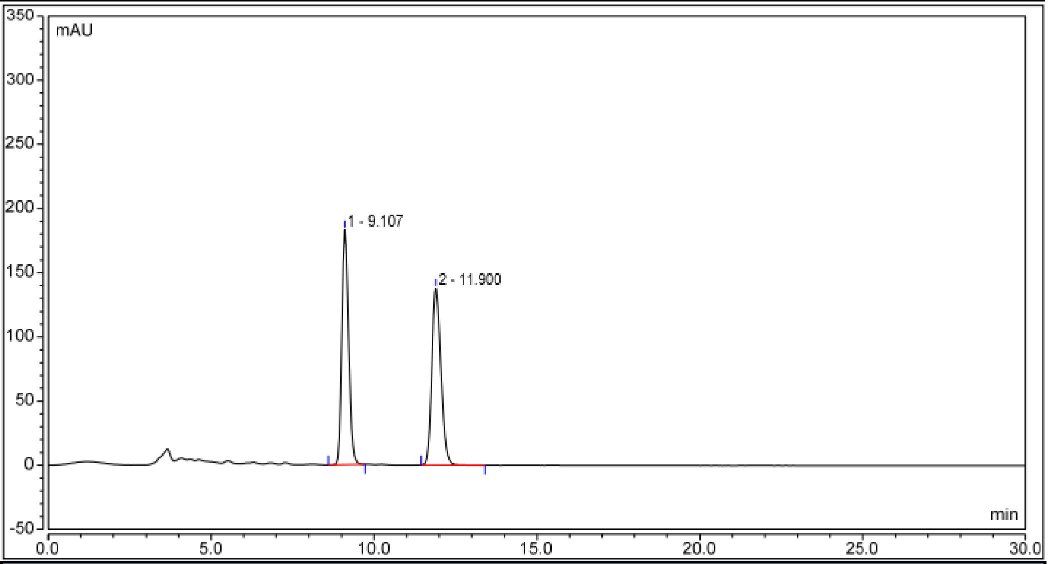

Integration Results

No.	Retention Time min	Area mAu*min	Height mAU	Relative Area %	Relative Height %
1	9.107	44.438	182.825	49.85	57.04
2	11.900	44.704	137.701	50.15	42.96
**Total:**		**89.142**	**320.526**	**100.00**	**100.00**

methyl (S)-2-(1-benzyl-4,7-dichloro-2-oxo-3-(pyrimidin-5-ylmethyl)indolin-3-yl)acetate (**5w**)

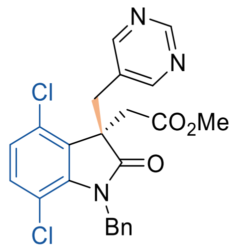

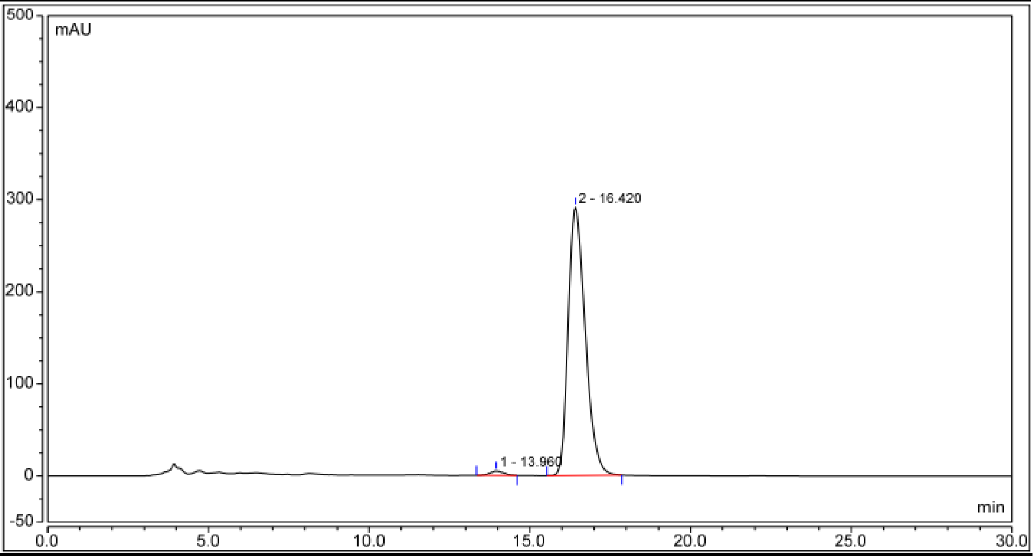

Chromatogram

Integration Results

No.	Retention Time min	Area mAu*min	Height mAU	Relative Area %	Relative Height %
1	13.960	2.369	4.792	1.30	1.62
2	16.420	179.204	291.538	98.70	98.38
**Total:**		**181.573**	**296.329**	**100.00**	**100.00**

Chromatogram

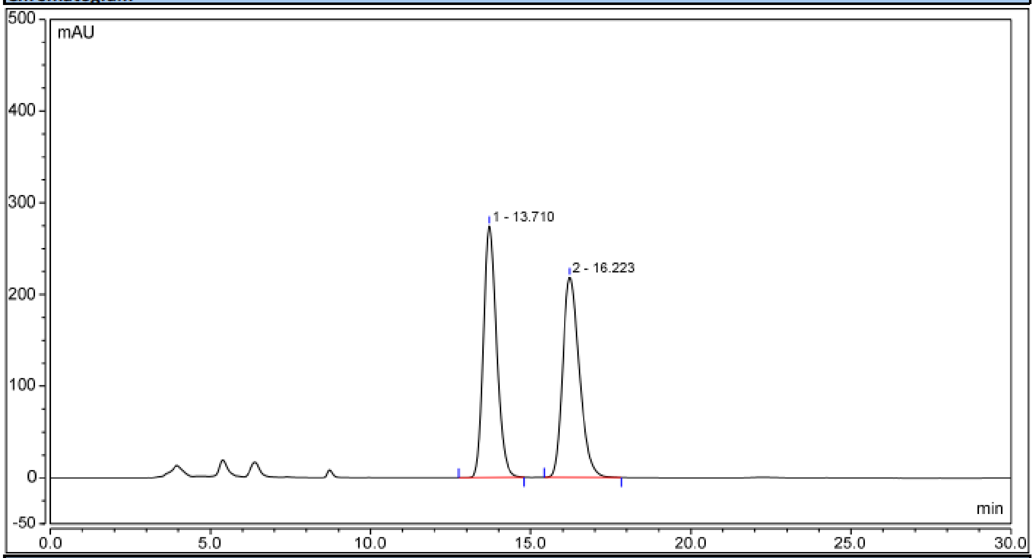

Integration Results

No.	Retention Time min	Area mAu*min	Height mAU	Relative Area %	Relative Height %
1	13.710	131.943	274.273	50.04	55.68
2	16.223	131.755	218.342	49.96	44.32
**Total:**		**263.697**	**492.615**	**100.00**	**100.00**

tert-butyl (S)-3-((1-benzyl-4,7-dichloro-3-(2-methoxy-2-oxoethyl)-2-oxoindolin-3-yl)methyl)-1H-indole-1-carboxylate (**5x**)

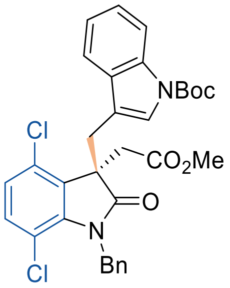

Chromatogram

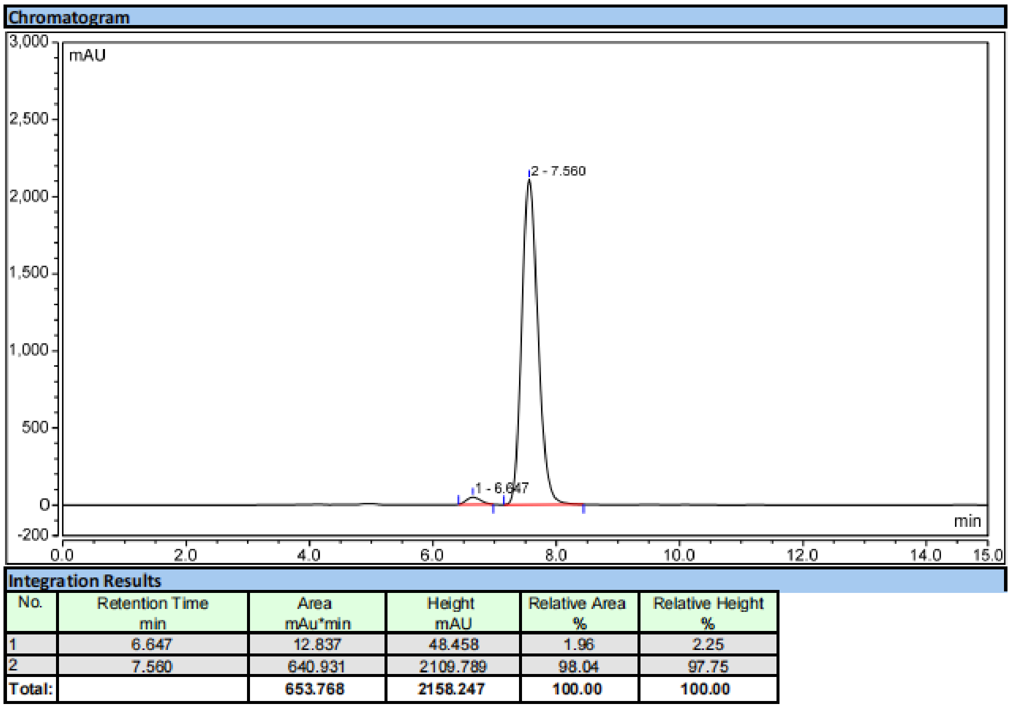

Integration Results

No.	Retention Time min	Area mAu*min	Height mAU	Relative Area %	Relative Height %
1	6.647	12.837	48.458	1.96	2.25
2	7.560	640.931	2109.789	98.04	97.75
**Total:**		**653.768**	**2158.247**	**100.00**	**100.00**

Chromatogram

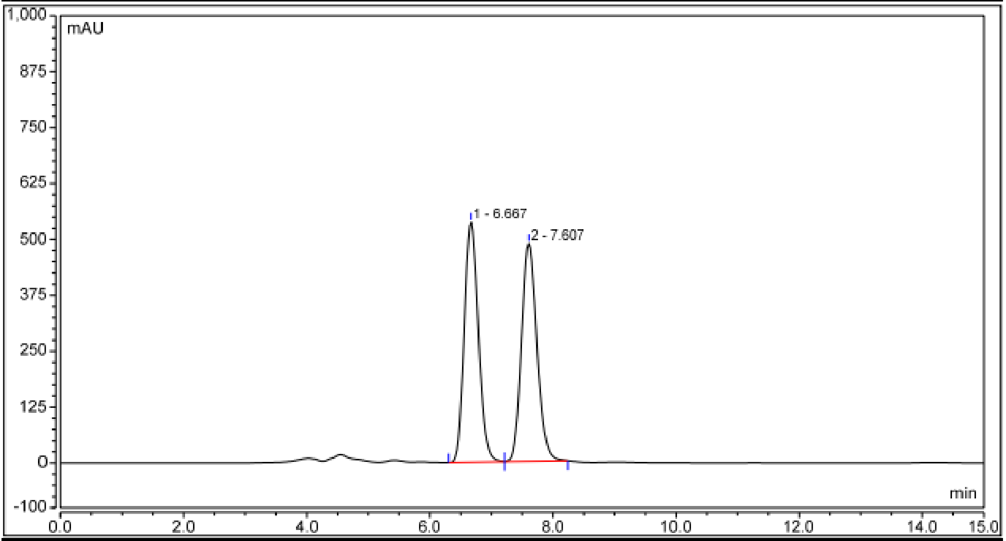

Integration Results

No.	Retention Time min	Area mAu*min	Height mAU	Relative Area %	Relative Height %
1	6.667	142.880	537.222	50.27	52.43
2	7.607	141.348	487.456	49.73	47.57
**Total:**		**284.227**	**1024.678**	**100.00**	**100.00**

methyl (S)-2-(1-benzyl-4,7-dichloro-2-oxo-3-(quinolin-5-ylmethyl)indolin-3-yl)acetate (**5y**)

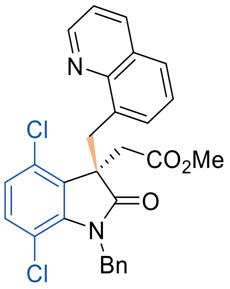

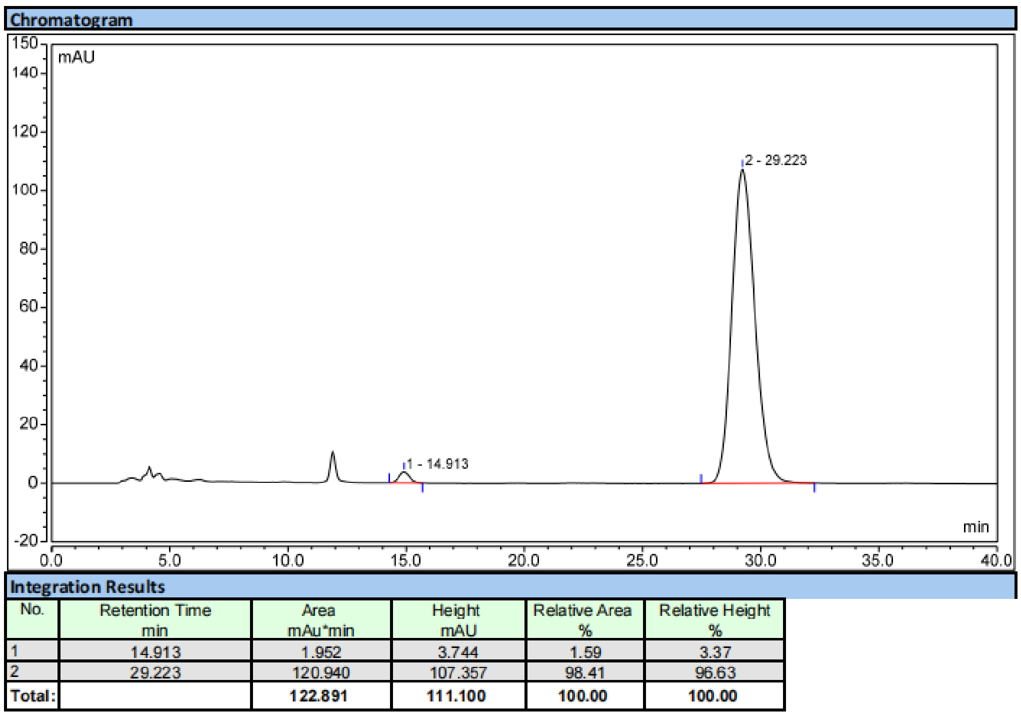

Integration Results

No.	Retention Time min	Area mAu*min	Height mAU	Relative Area %	Relative Height %
1	14.913	1.952	3.744	1.59	3.37
2	29.223	120.940	107.357	98.41	96.63
**Total:**		**122.891**	**111.100**	**100.00**	**100.00**

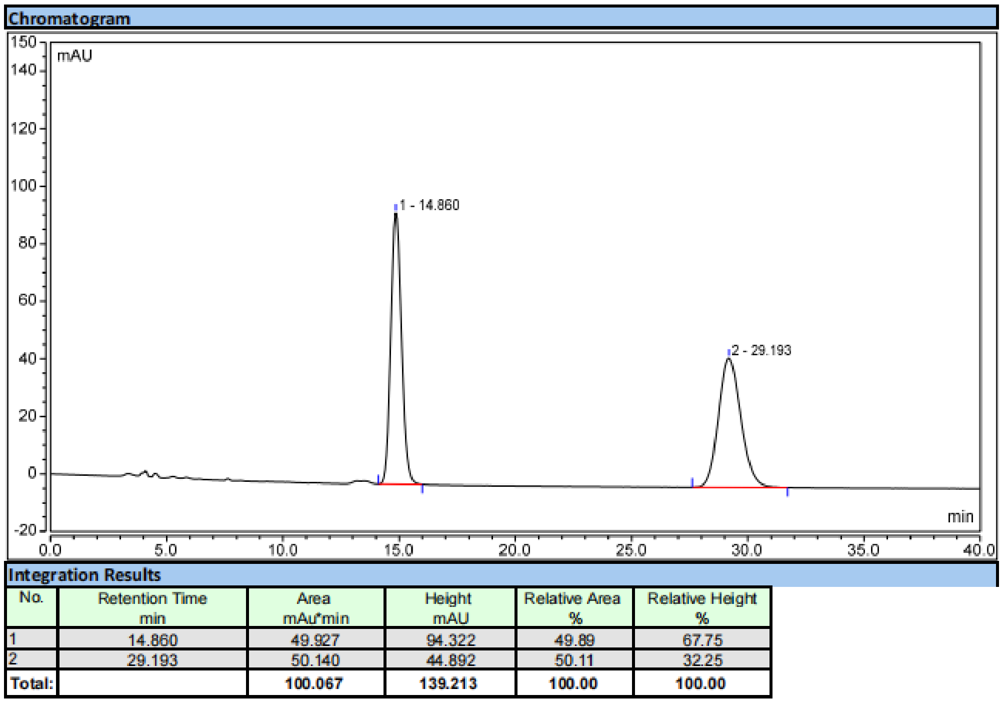

Integration Results

No.	Retention Time min	Area mAu*min	Height mAU	Relative Area %	Relative Height %
1	14.860	49.927	94.322	49.89	67.75
2	29.193	50.140	44.892	50.11	32.25
**Total:**		**100.067**	**139.213**	**100.00**	**100.00**

methyl (S)-2-(1-benzyl-4,7-dichloro-2-oxo-3-(quinoxalin-5-ylmethyl)indolin-3-yl)acetate (**5z**)

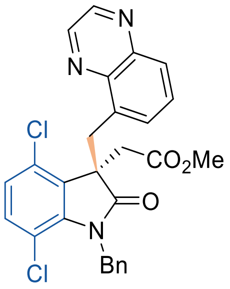

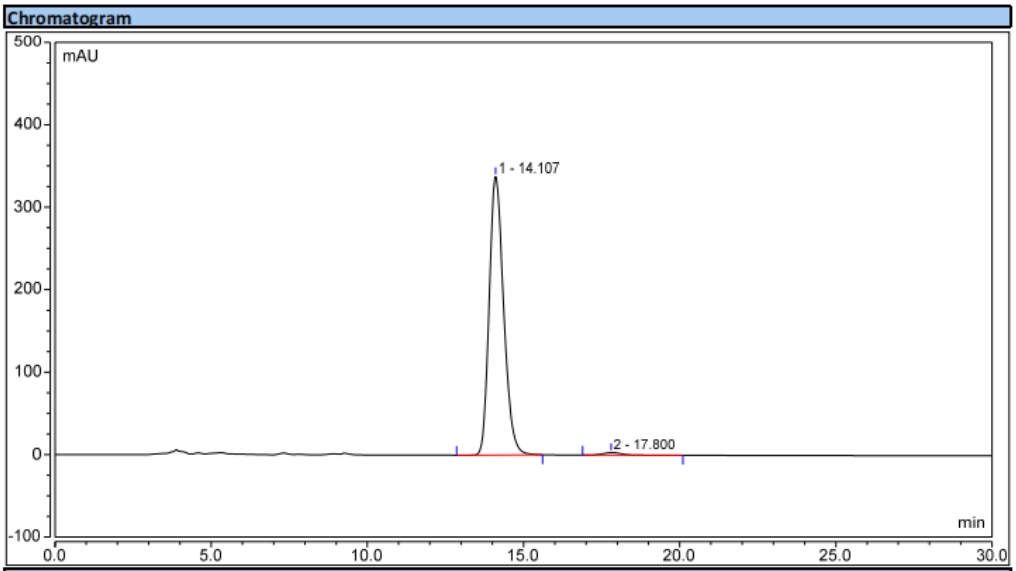

Integration Results

No.	Retention Time min	Area mAu*min	Height mAU	Relative Area %	Relative Height %
1	14.107	180.846	337.619	98.78	99.08
2	17.800	2.230	3.134	1.22	0.92
**Total:**		**183.077**	**340.753**	**100.00**	**100.00**

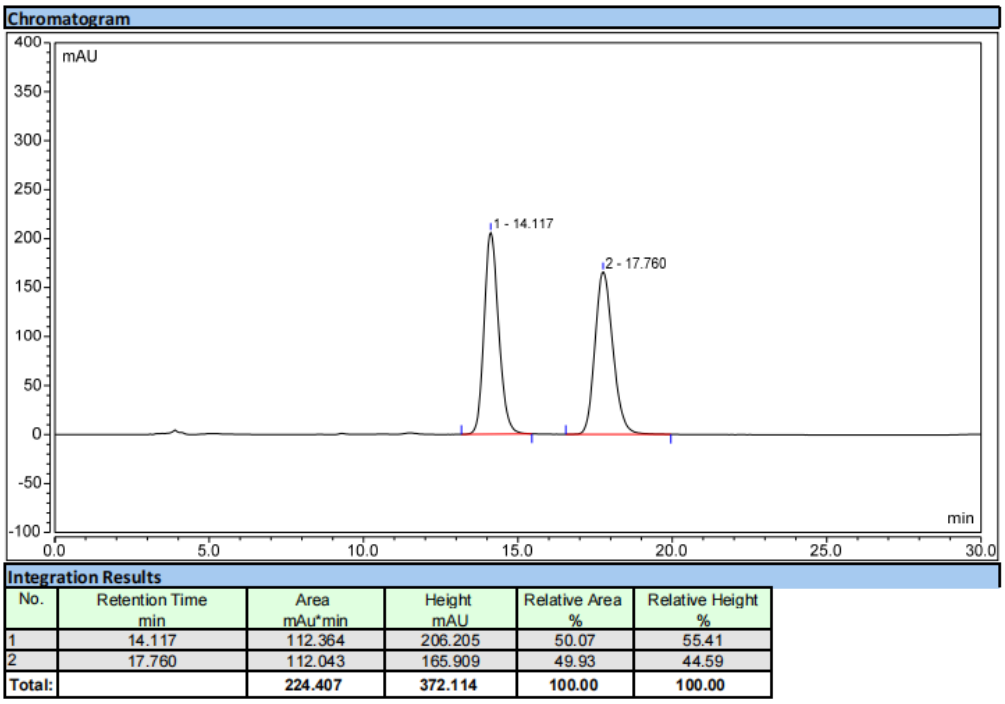

Integration Results

No.	Retention Time min	Area mAu*min	Height mAU	Relative Area %	Relative Height %
1	14.117	112.364	206.205	50.07	55.41
2	17.760	112.043	165.909	49.93	44.59
**Total:**		**224.407**	**372.114**	**100.00**	**100.00**

methyl (S)-2-(1-benzyl-4,7-dichloro-3-((7-methoxy-2-oxo-2H-chromen-4-yl)methyl)-2-oxoindolin-3-yl)acetate (**5aa**)

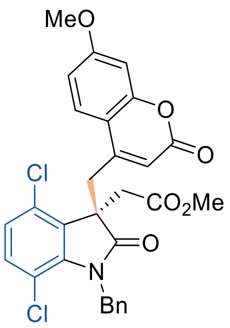

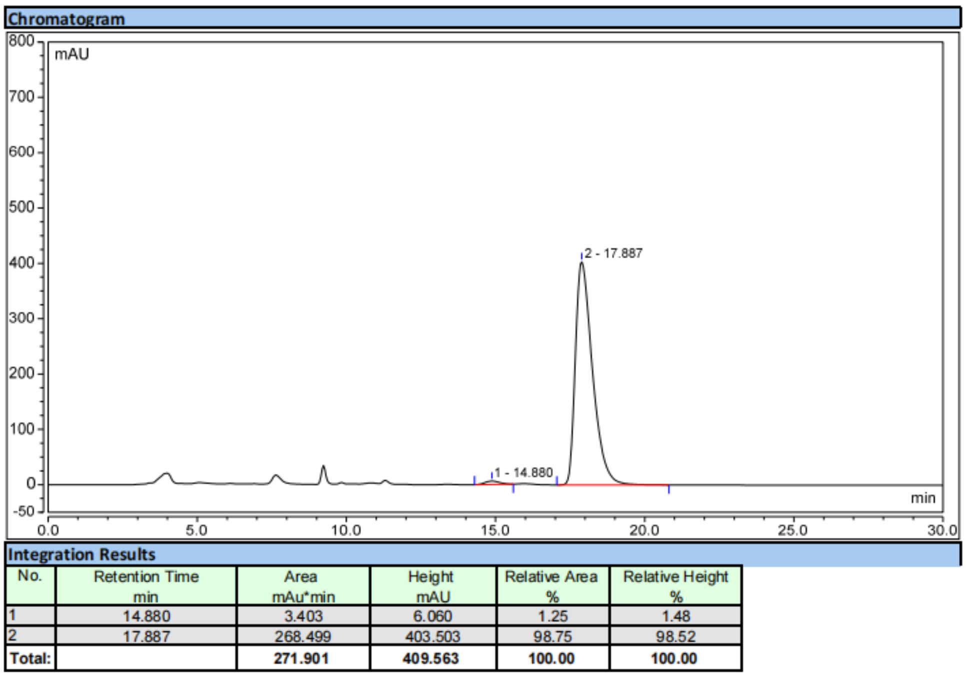

Integration Results

No.	Retention Time min	Area mAu*min	Height mAU	Relative Area %	Relative Height %
1	14.880	3.403	6.060	1.25	1.48
2	17.887	268.499	403.503	98.75	98.52
Total:		271.901	409.563	100.00	100.00

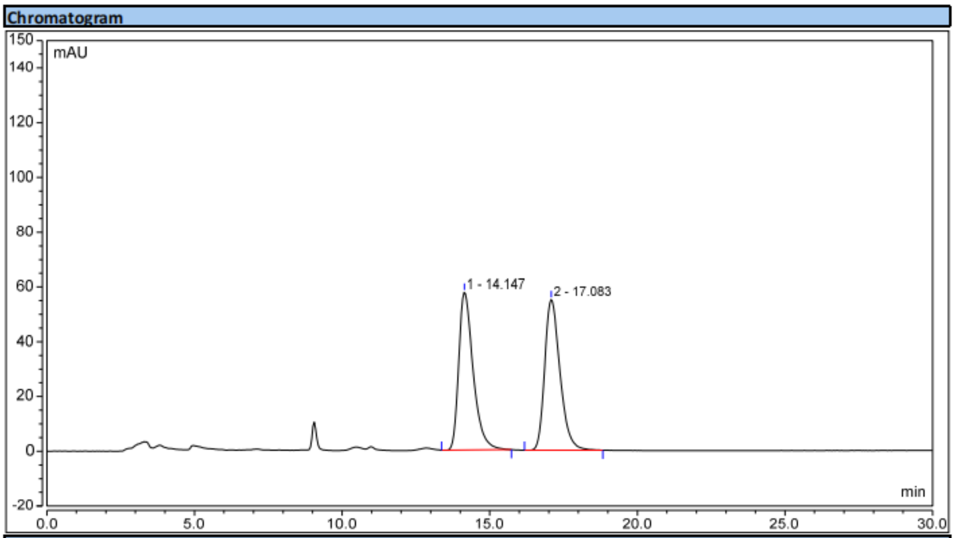

Integration Results

No.	Retention Time min	Area mAu*min	Height mAU	Relative Area %	Relative Height %
1	14.147	32.610	57.647	49.67	51.16
2	17.083	33.049	55.027	50.33	48.84
Total:		65.659	112.674	100.00	100.00

4-(((S)-1-benzyl-4,7-dichloro-3-(2-methoxy-2-oxoethyl)-2-oxoindolin-3-yl)methyl)phenyl (S)-2-(6-methoxynaphthalen-2-yl)propanoate (**5ab**)

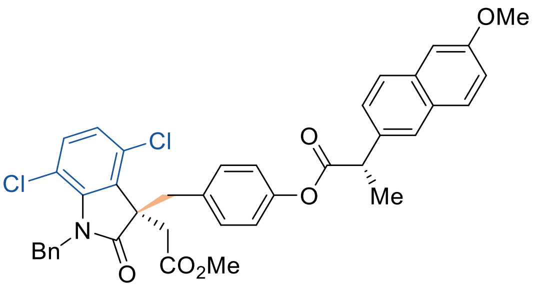

Chromatogram

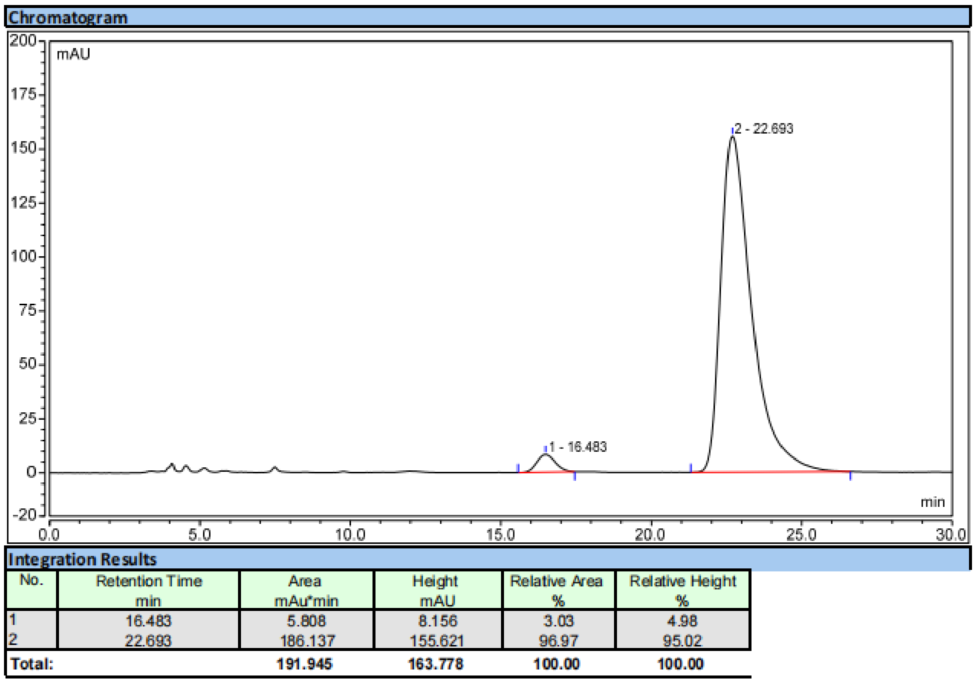

Integration Results

No.	Retention Time<br>min	Area<br>mAu*min	Height<br>mAU	Relative Area<br>%	Relative Height<br>%
1	16.483	5.808	8.156	3.03	4.98
2	22.693	186.137	155.621	96.97	95.02
**Total:**		**191.945**	**163.778**	**100.00**	**100.00**

Chromatogram

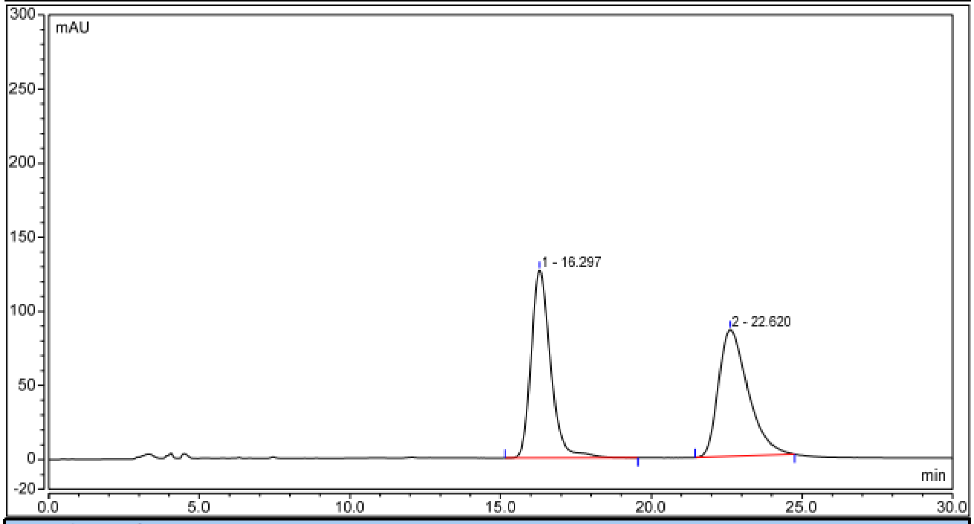

Integration Results

No.	Retention Time<br>min	Area<br>mAu*min	Height<br>mAU	Relative Area<br>%	Relative Height<br>%
1	16.297	95.198	126.540	49.51	59.73
2	22.620	97.068	85.308	50.49	40.27
**Total:**		**192.265**	**211.848**	**100.00**	

methyl (S)-2-(1-benzyl-4,7-dichloro-3-((4-(4-fluorophenyl)-6-isopropyl-2-(N-

methylmethylsulfonamido)pyrimidin-5-yl)methyl)-2-oxoindolin-3-yl)acetate (**5ac**)

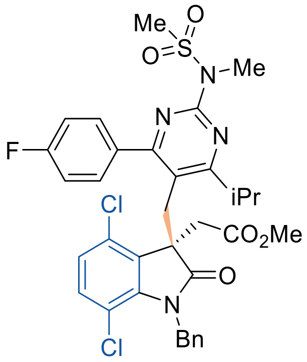

Chromatogram

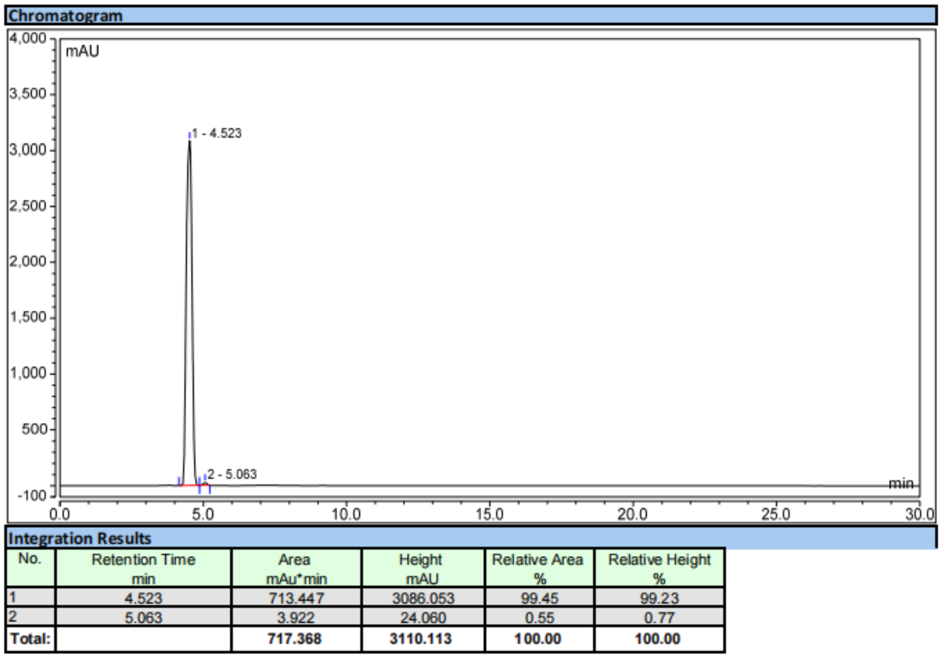

Integration Results

No.	Retention Time min	Area mAu*min	Height mAU	Relative Area %	Relative Height %
1	4.523	713.447	3086.053	99.45	99.23
2	5.063	3.922	24.060	0.55	0.77
**Total:**		**717.368**	**3110.113**	**100.00**	**100.00**

Chromatogram

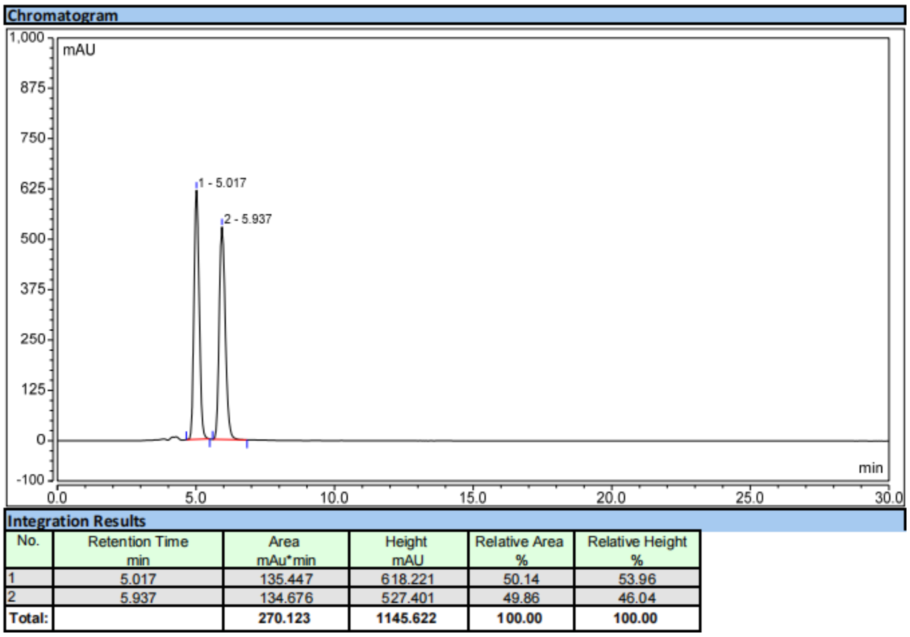

Integration Results

No.	Retention Time min	Area mAu*min	Height mAU	Relative Area %	Relative Height %
1	5.017	135.447	618.221	50.14	53.96
2	5.937	134.676	527.401	49.86	46.04
**Total:**		**270.123**	**1145.622**	**100.00**	**100.00**

(R)-2,5,7,8-tetramethyl-2-((4R,8S)-4,8,11-trimethyldodecyl)chroman-6-yl 4-(((S)-1-benzyl-4,7-dichloro-3-(2-methoxy-2-oxoethyl)-2-oxoindolin-3-yl)methyl)benzoate (**5ad**)

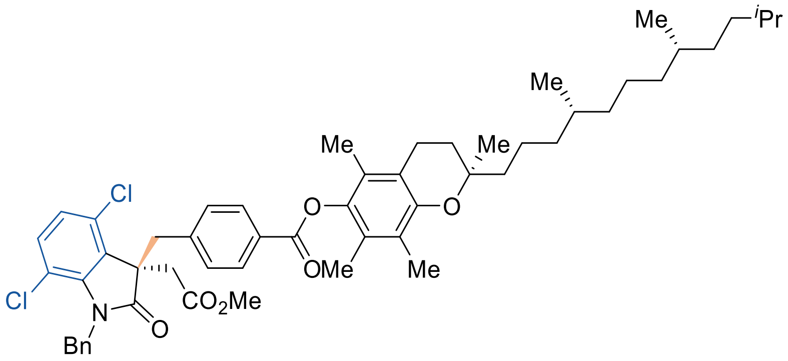

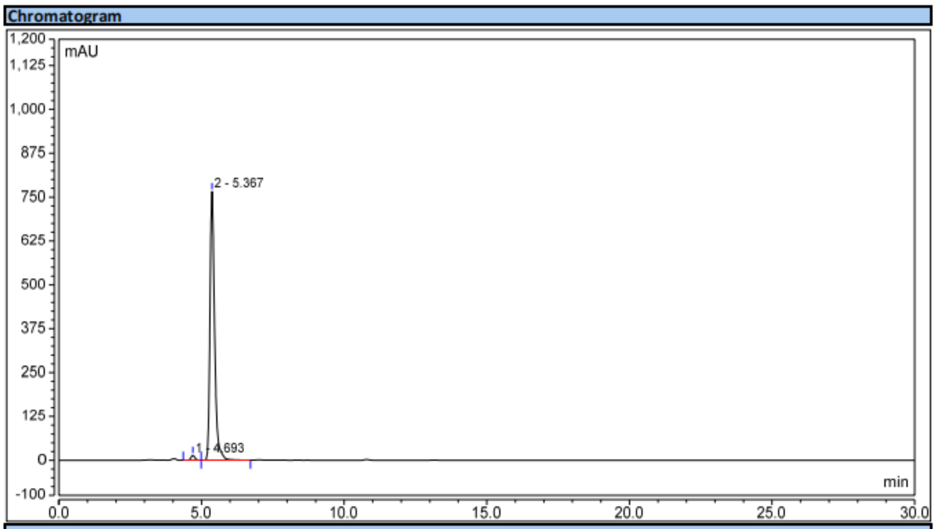

Integration Results

No.	Retention Time min	Area mAu*min	Height mAU	Relative Area %	Relative Height %
1	4.693	2.032	13.835	1.39	1.78
2	5.367	143.884	765.549	98.61	98.22
**Total:**		**145.916**	**779.384**	**100.00**	**100.00**

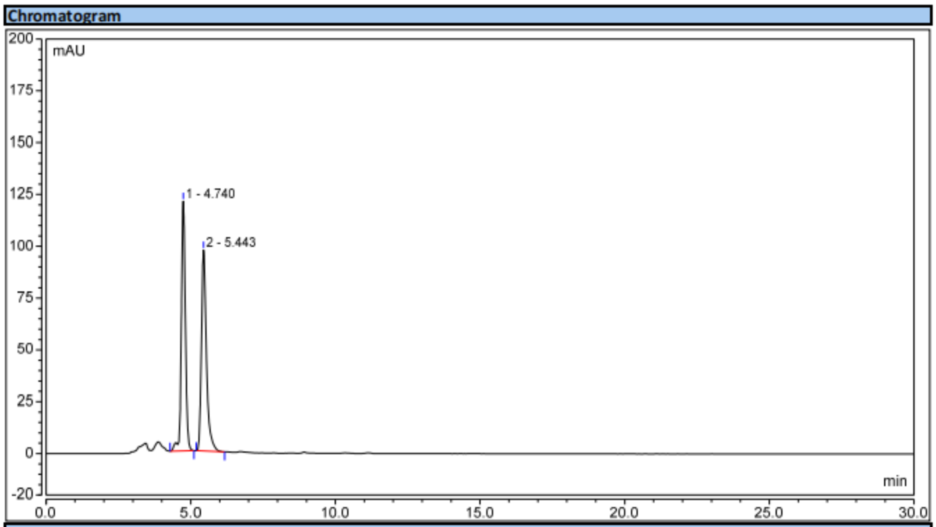

Integration Results

No.	Retention Time min	Area mAu*min	Height mAU	Relative Area %	Relative Height %
1	4.740	19.423	120.426	49.90	55.41
2	5.443	19.504	96.902	50.10	44.59
**Total:**		**38.927**	**217.329**	**100.00**	**100.00**

methyl (S)-2-(1-benzyl-3-cinnamyl-4,6-difluoro-2-oxoindolin-3-yl)acetate (**6a**)

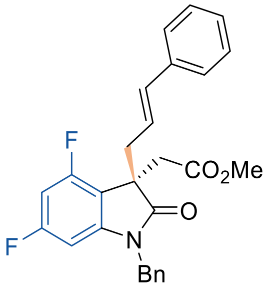

Chromatogram

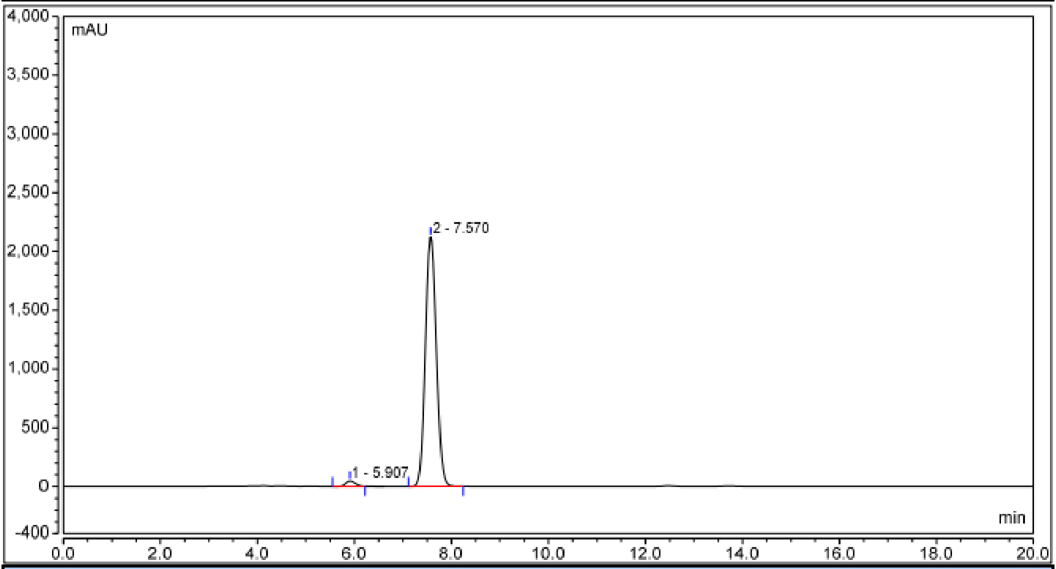

Integration Results

No.	Retention Time<br>min	Area<br>mAu*min	Height<br>mAU	Relative Area<br>%	Relative Height<br>%
1	5.907	10.798	46.978	1.90	2.16
2	7.570	556.358	2123.632	98.10	97.84
**Total:**		**567.156**	**2170.610**	**100.00**	**100.00**

Chromatogram

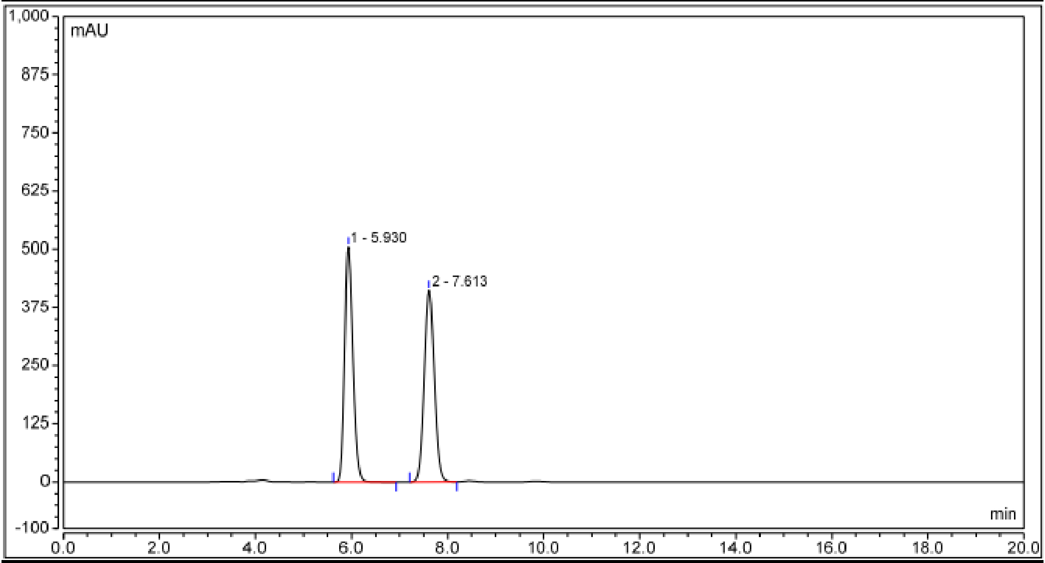

Integration Results

No.	Retention Time<br>min	Area<br>mAu*min	Height<br>mAU	Relative Area<br>%	Relative Height<br>%
1	5.930	100.440	505.262	50.04	55.09
2	7.613	100.261	411.954	49.96	44.91
**Total:**		**200.701**	**917.216**	**100.00**	**100.00**

methyl (S,E)-4-(1-benzyl-4,7-dichloro-3-(2-methoxy-2-oxoethyl)-2-oxoindolin-3-yl)but-2-enoate (**6b**)

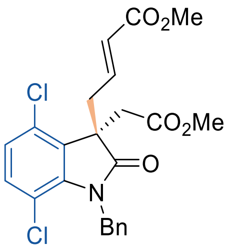

Chromatogram

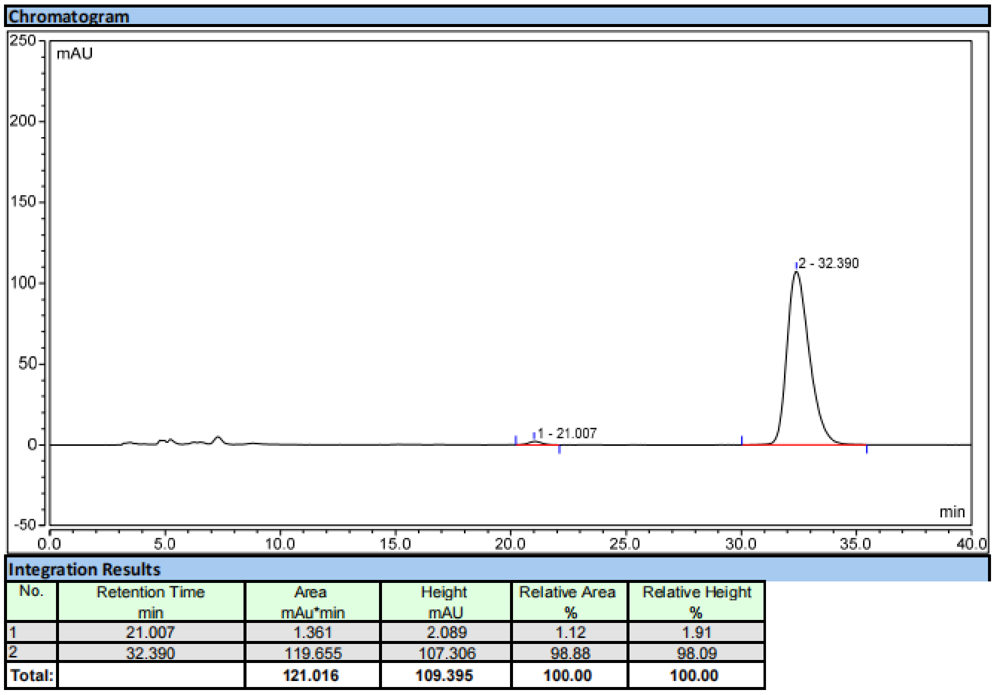

Integration Results

No.	Retention Time min	Area mAu*min	Height mAU	Relative Area %	Relative Height %
1	21.007	1.361	2.089	1.12	1.91
2	32.390	119.655	107.306	98.88	98.09
**Total:**		**121.016**	**109.395**	**100.00**	**100.00**

Chromatogram

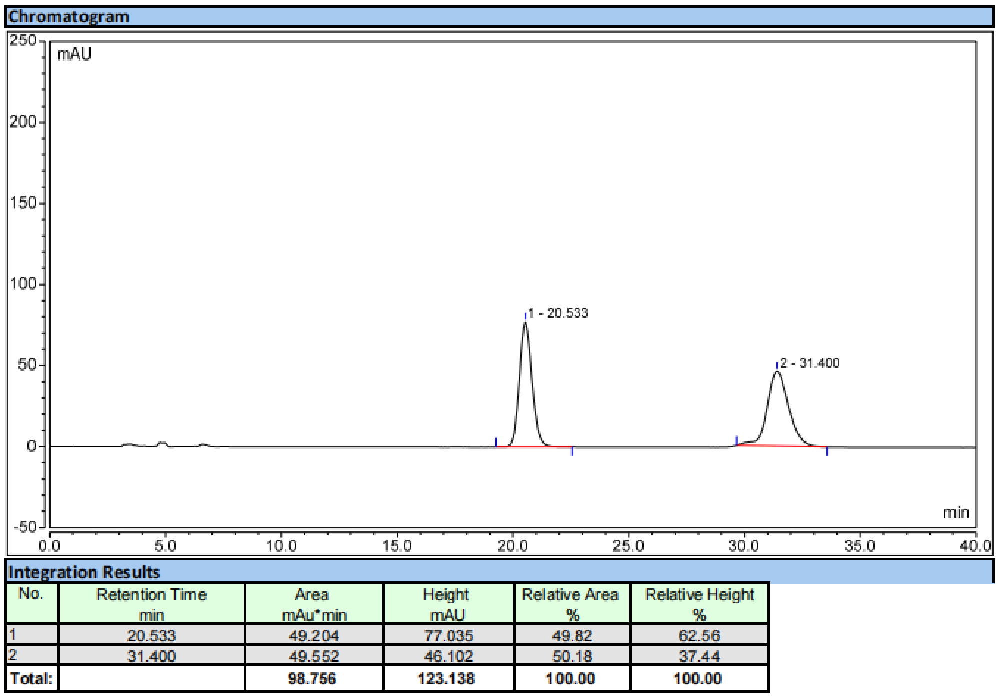

Integration Results

No.	Retention Time min	Area mAu*min	Height mAU	Relative Area %	Relative Height %
1	20.533	49.204	77.035	49.82	62.56
2	31.400	49.552	46.102	50.18	37.44
**Total:**		**98.756**	**123.138**	**100.00**	**100.00**

methyl (S)-2-(1-benzyl-4,7-dichloro-3-(3-methylbut-2-en-1-yl)-2-oxoindolin-3-yl)acetate (**6c**)

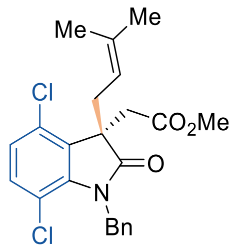

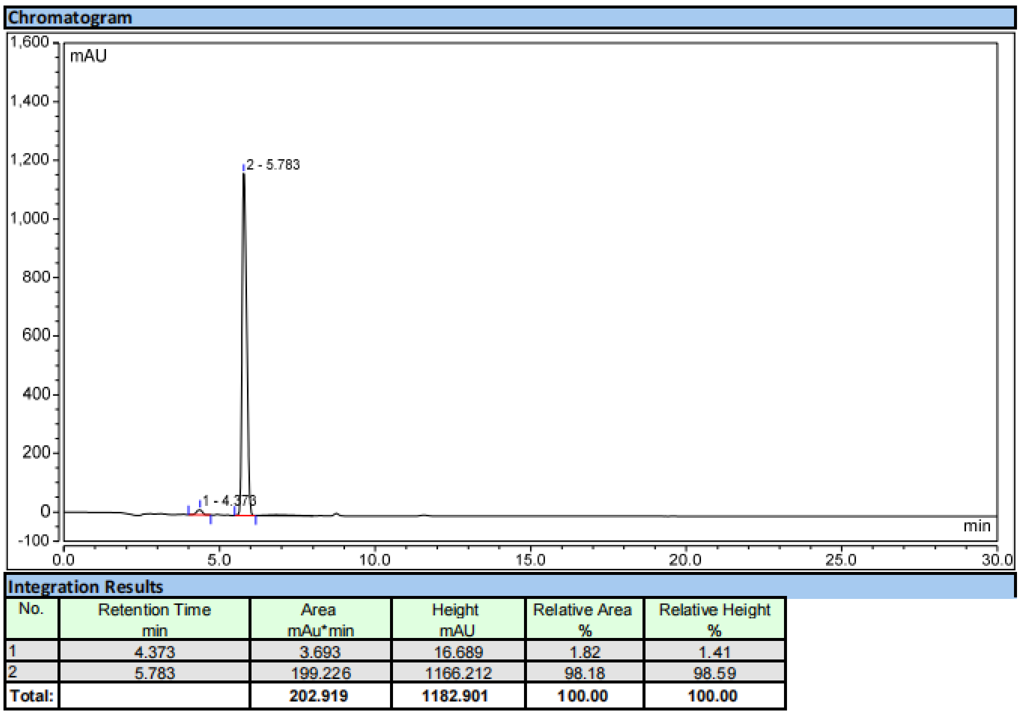

Integration Results

No.	Retention Time min	Area mAu*min	Height mAU	Relative Area %	Relative Height %
1	4.373	3.693	16.689	1.82	1.41
2	5.783	199.226	1166.212	98.18	98.59
**Total:**		**202.919**	**1182.901**	**100.00**	**100.00**

Chromatogram

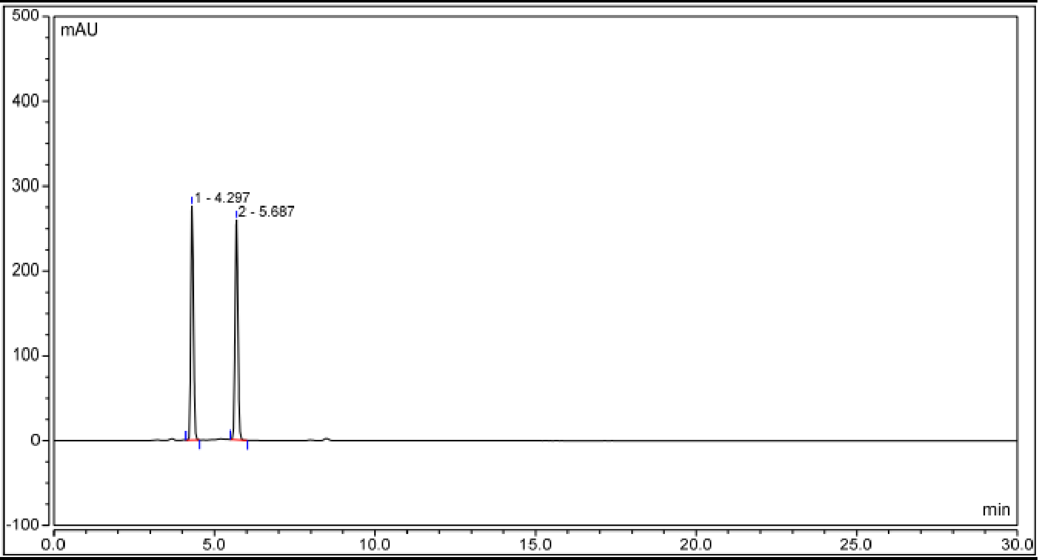

Integration Results

No.	Retention Time min	Area mAu*min	Height mAU	Relative Area %	Relative Height %
1	4.297	27.194	275.447	50.01	51.56
2	5.687	27.186	258.798	49.99	48.44
**Total:**		**54.380**	**534.245**	**100.00**	**100.00**

methyl (S)-2-(1-benzyl-4,7-dichloro-3-(2-methylallyl)-2-oxoindolin-3-yl)acetate (**6d**)

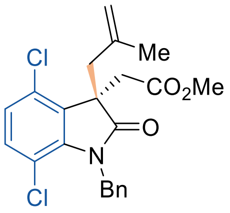

Chromatogram

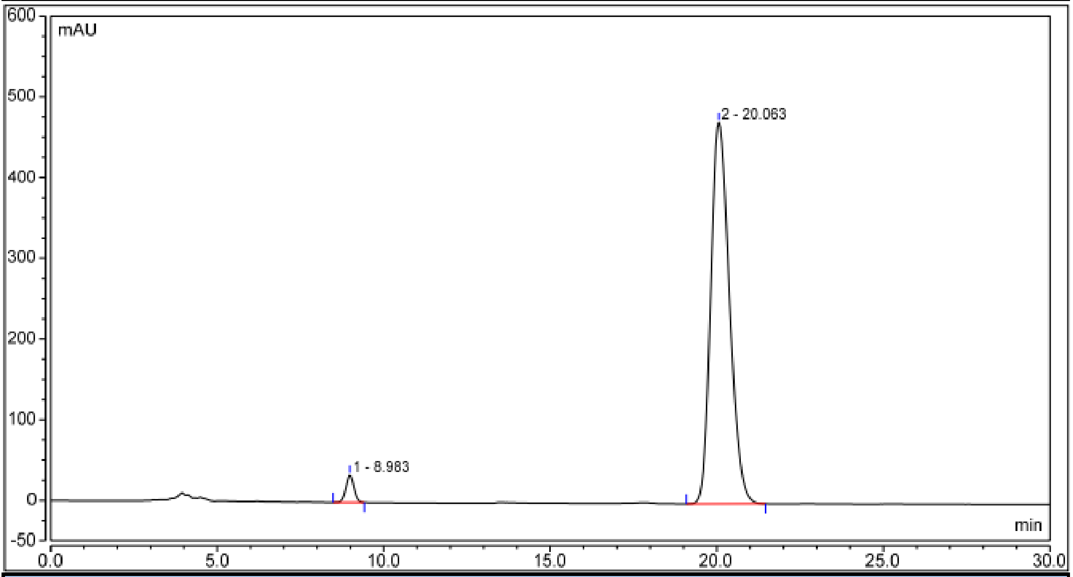

Integration Results

No.	Retention Time min	Area mAu*min	Height mAU	Relative Area %	Relative Height %
1	8.983	9.496	33.863	3.00	6.69
2	20.063	307.273	472.507	97.00	93.31
**Total:**		**316.769**	**506.370**	**100.00**	**100.00**

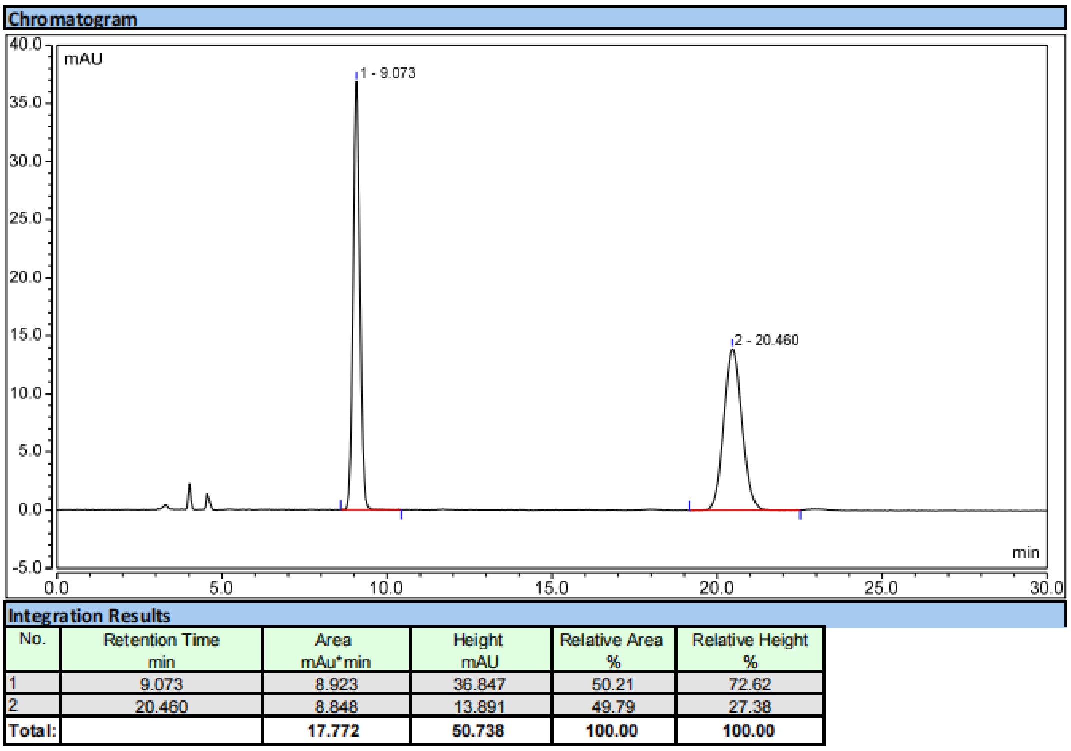

Integration Results

No.	Retention Time min	Area mAu*min	Height mAU	Relative Area %	Relative Height %
1	9.073	8.923	36.847	50.21	72.62
2	20.460	8.848	13.891	49.79	27.38
**Total:**		**17.772**	**50.738**	**100.00**	**100.00**

methyl (S)-2-(1-benzyl-4,6-difluoro-2-oxo-3-(3-phenylprop-2-yn-1-yl)indolin-3-yl)acetate (**6e**)

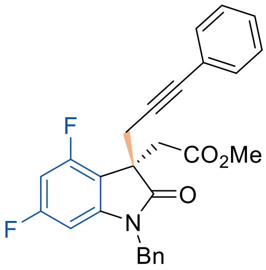

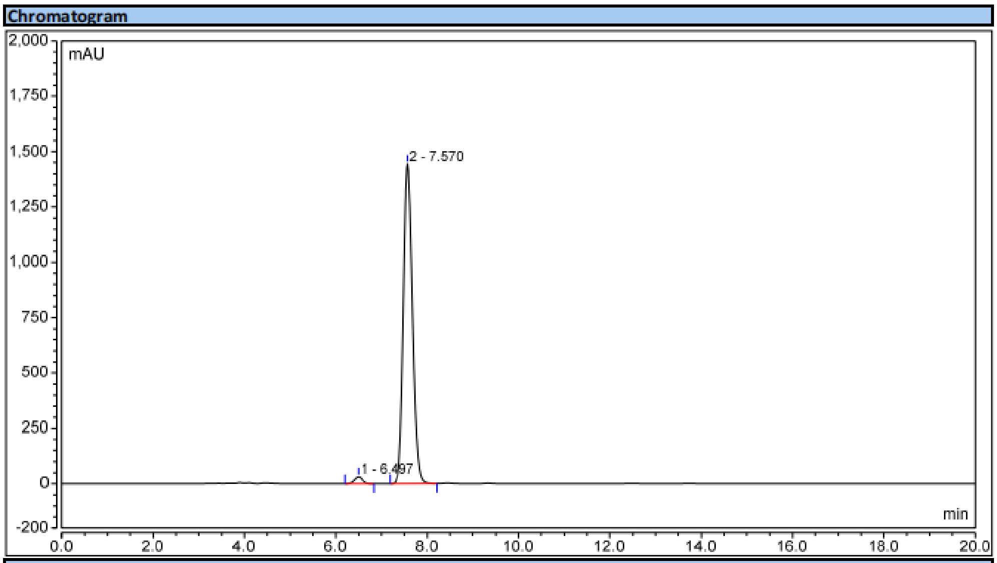

Integration Results

No.	Retention Time min	Area mAu*min	Height mAU	Relative Area %	Relative Height %
1	6.497	6.136	30.466	1.82	2.07
2	7.570	330.710	1443.169	98.18	97.93
**Total:**		**336.846**	**1473.635**	**100.00**	**100.00**

Chromatogram

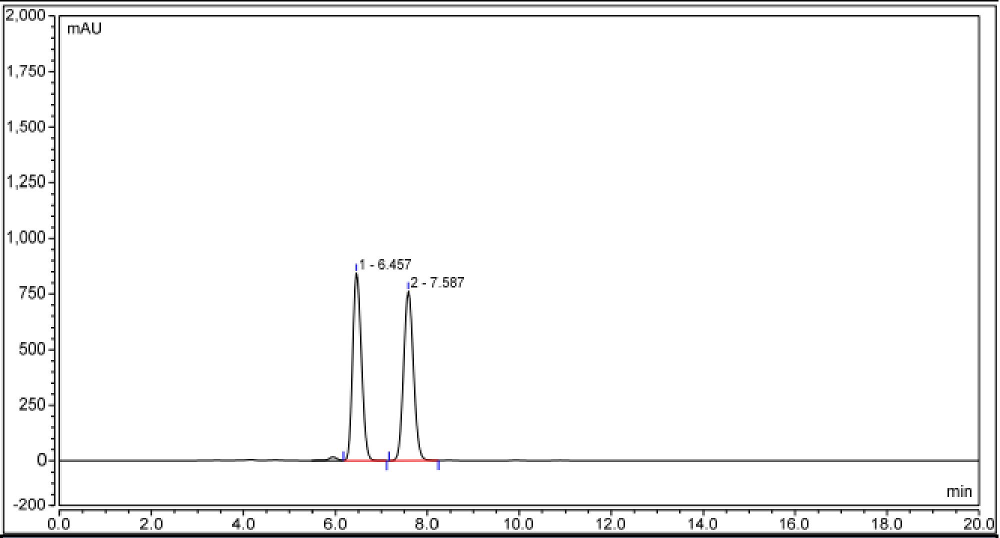

Integration Results

No.	Retention Time min	Area mAu*min	Height mAU	Relative Area %	Relative Height %
1	6.457	187.842	843.213	49.58	52.57
2	7.587	191.045	760.783	50.42	47.43
**Total:**		**378.887**	**1603.996**	**100.00**	**100.00**

(S)-2-(1,3-bis(3-methylbut-2-en-1-yl)-2-oxoindolin-3-yl)-N-methylacetamide (**7a**)

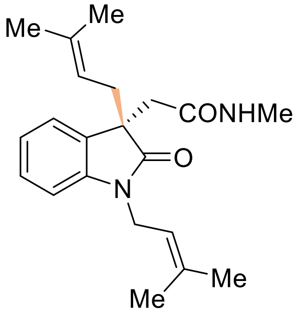

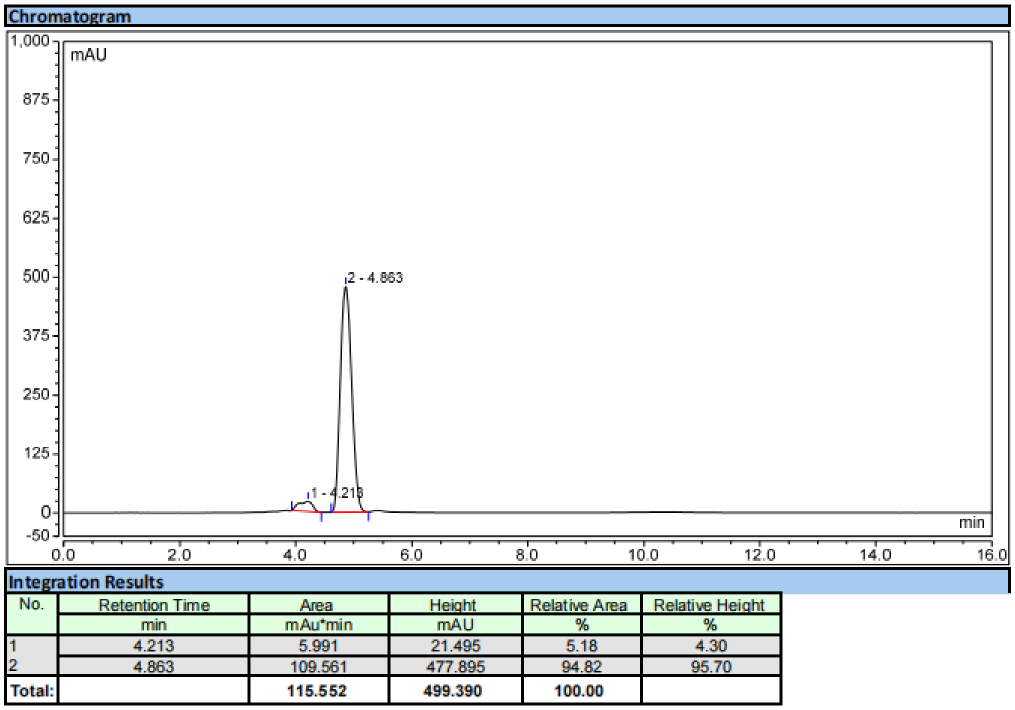

Integration Results

No.	Retention Time	Area	Height	Relative Area	Relative Height
	min	mAu*min	mAU	%	%
1	4.213	5.991	21.495	5.18	4.30
2	4.863	109.561	477.895	94.82	95.70
**Total:**		**115.552**	**499.390**	**100.00**	

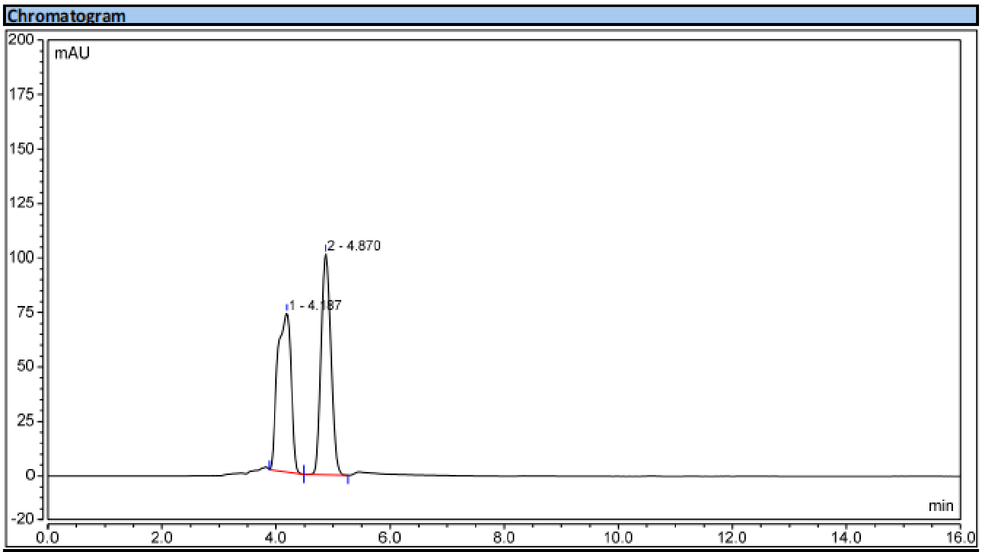

Integration Results

No.	Retention Time	Area	Height	Relative Area	Relative Height
	min	mAu*min	mAU	%	%
1	4.187	19.781	72.941	49.23	41.84
2	4.870	20.398	101.398	50.77	58.16
**Total:**		**40.180**	**174.339**	**100.00**	

(S)-2-(6-bromo-1,3-bis(3-methylbut-2-en-1-yl)-2-oxoindolin-3-yl)-N-methylacetamide (**7b**)

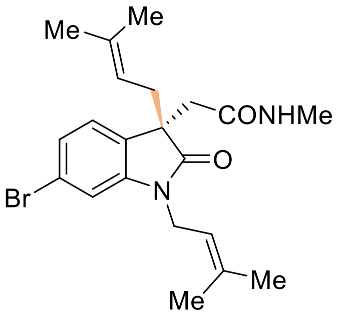

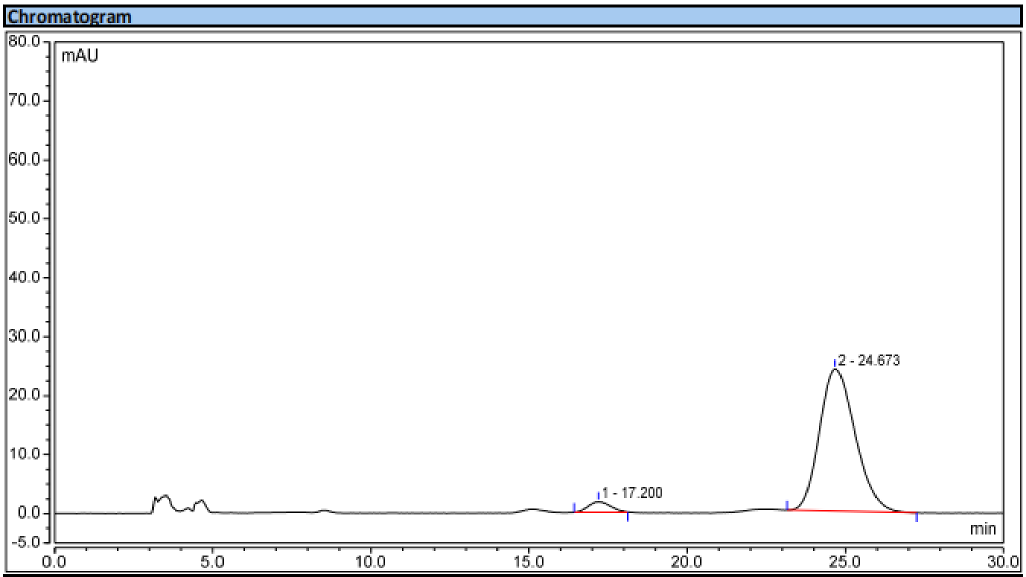

Integration Results

No.	Retention Time min	Area mAu*min	Height mAU	Relative Area %	Relative Height %
1	17.200	1.449	1.777	4.39	6.87
2	24.673	31.533	24.099	95.61	93.13
**Total:**		**32.982**	**25.876**	**100.00**	**100.00**

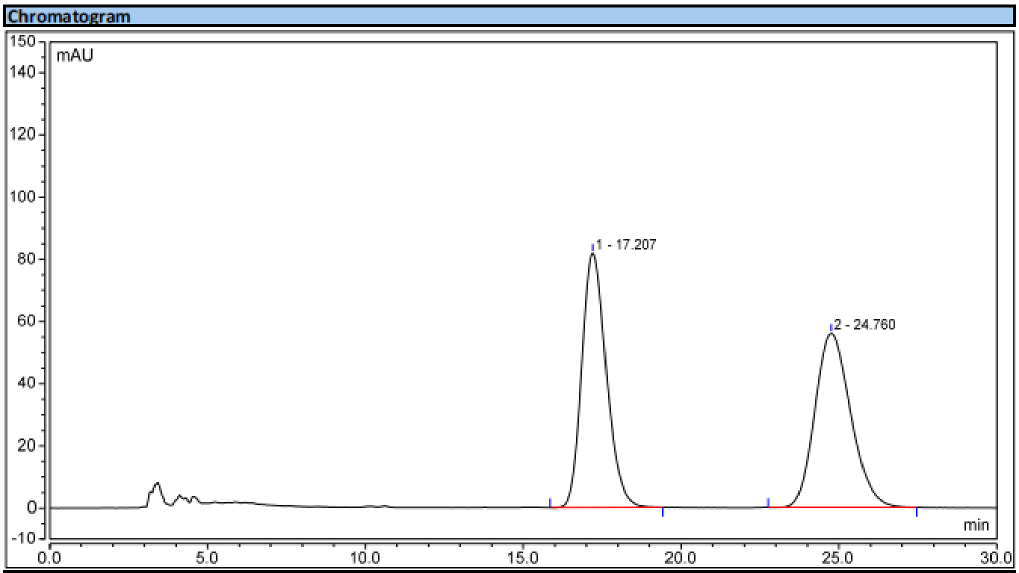

Integration Results

No.	Retention Time min	Area mAu*min	Height mAU	Relative Area %	Relative Height %
1	17.207	73.512	81.801	49.86	59.40
2	24.760	73.920	55.907	50.14	40.60
**Total:**		**147.432**	**137.708**	**100.00**	**100.00**

Methyl (S,E)-2-methyl-4-(3-(2-(methylamino)-2-oxoethyl)-2-oxoindolin-3-yl)but-2-enoate (**7c**)

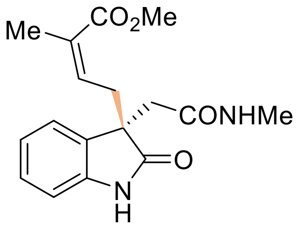

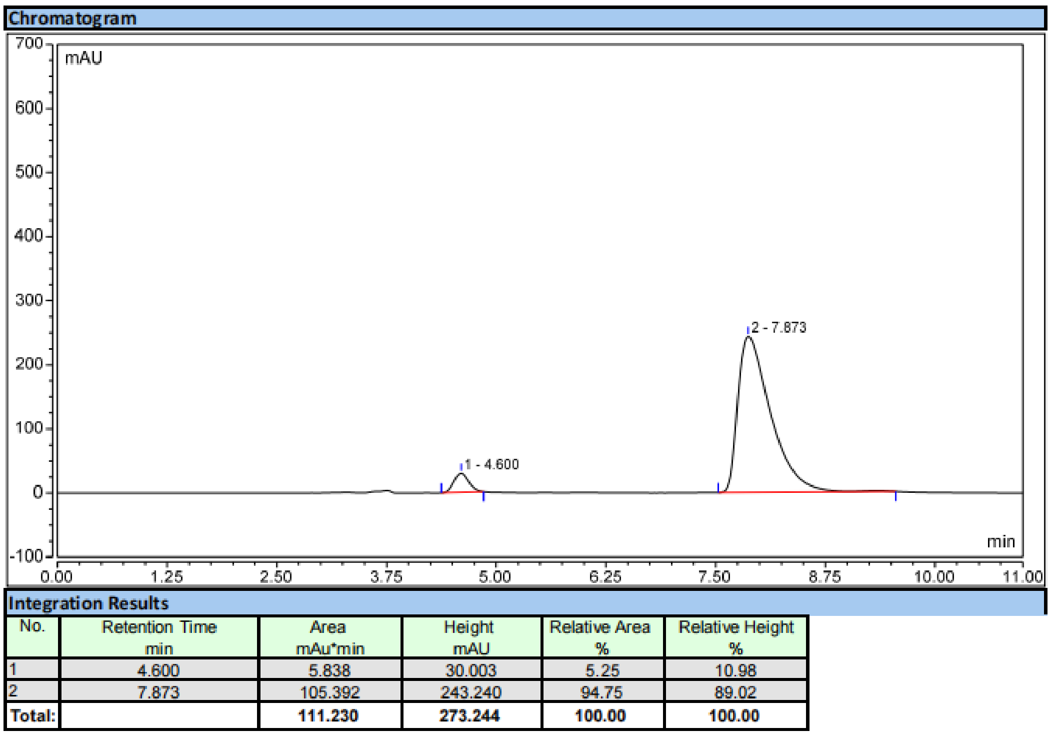

Integration Results

No.	Retention Time min	Area mAu*min	Height mAU	Relative Area %	Relative Height %
1	4.600	5.838	30.003	5.25	10.98
2	7.873	105.392	243.240	94.75	89.02
**Total:**		**111.230**	**273.244**	**100.00**	**100.00**

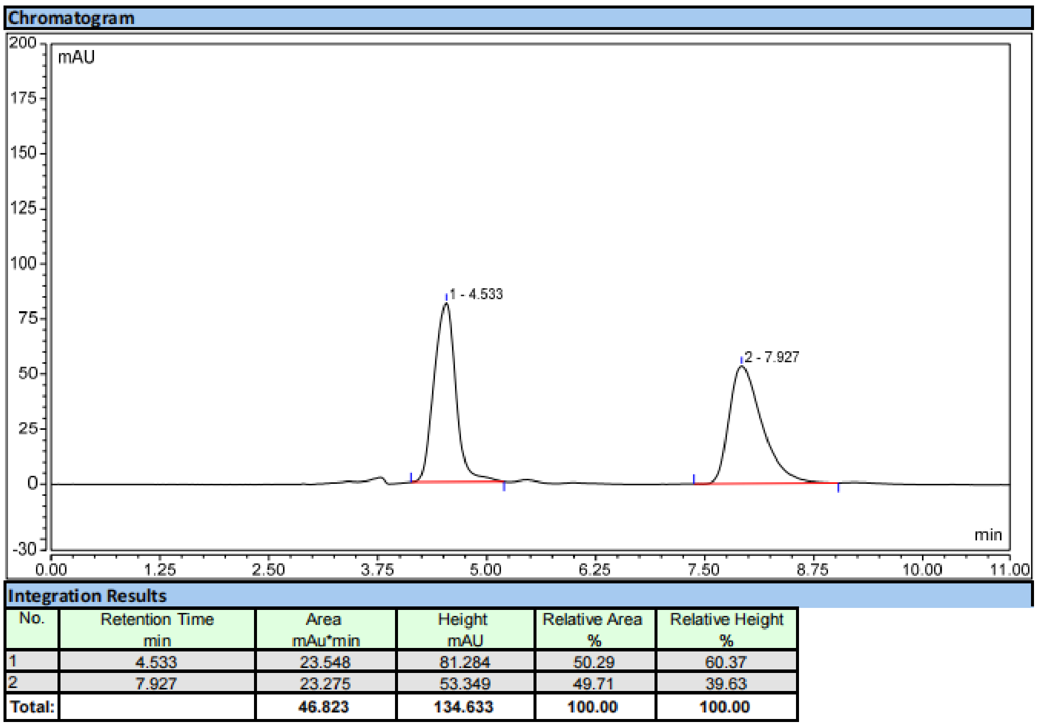

Integration Results

No.	Retention Time min	Area mAu*min	Height mAU	Relative Area %	Relative Height %
1	4.533	23.548	81.284	50.29	60.37
2	7.927	23.275	53.349	49.71	39.63
**Total:**		**46.823**	**134.633**	**100.00**	**100.00**

NMR spectra:

4-(bromomethyl)phenyl (S)-2-(6-methoxynaphthalen-2-yl)propanoate

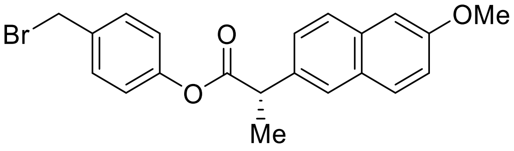

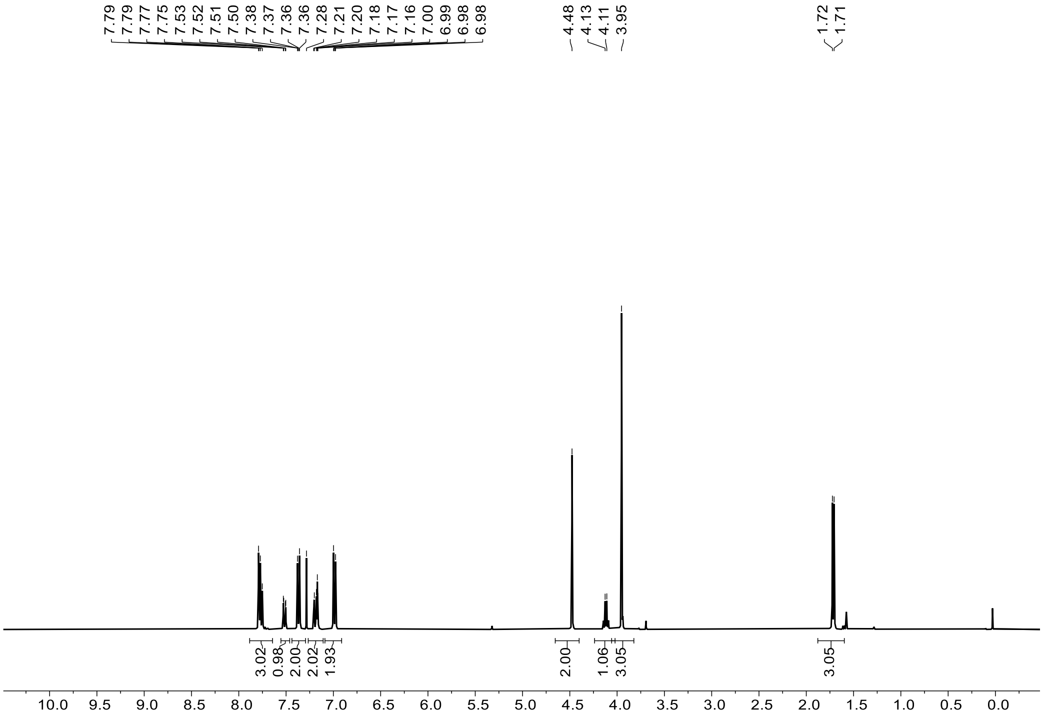

^{1}H NMR (400 MHz, $CDCl_3$)

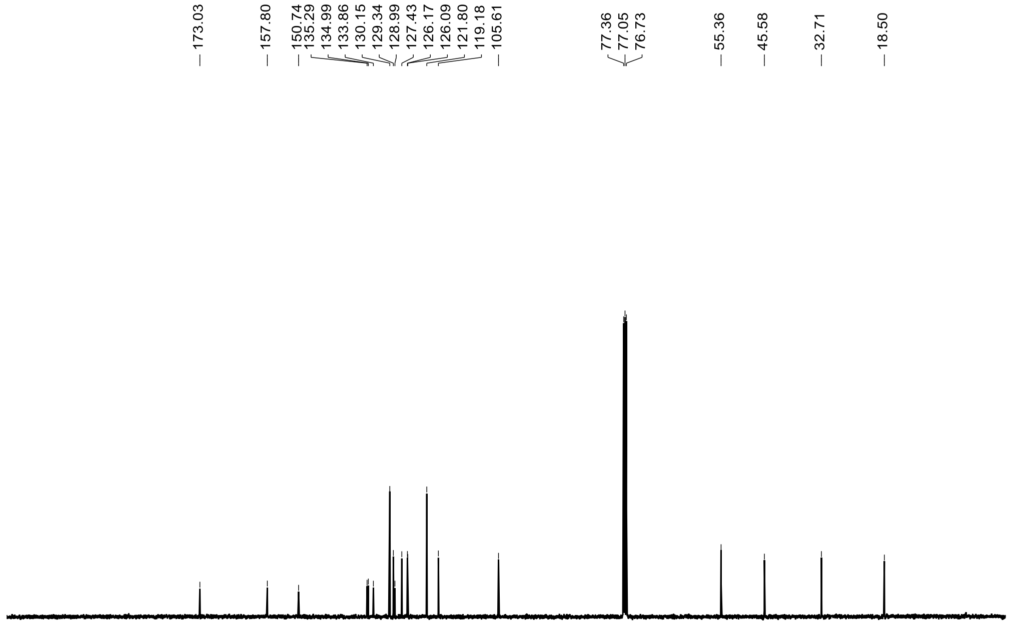

^{13}C NMR (101 MHz, $CDCl_3$)

(4-chlorophenyl)(3-(2-hydroxyethyl)-5-methoxy-2-methyl-1H-indol-1-yl)methanone

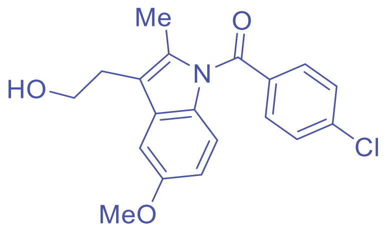

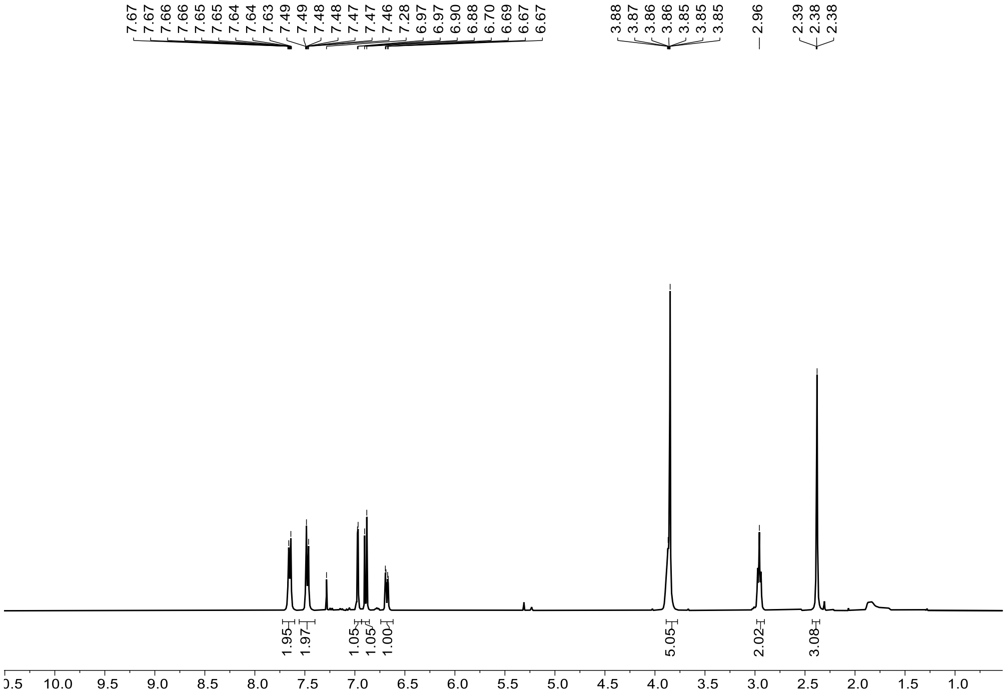

^{1}H NMR (400 MHz, $CDCl_3$)

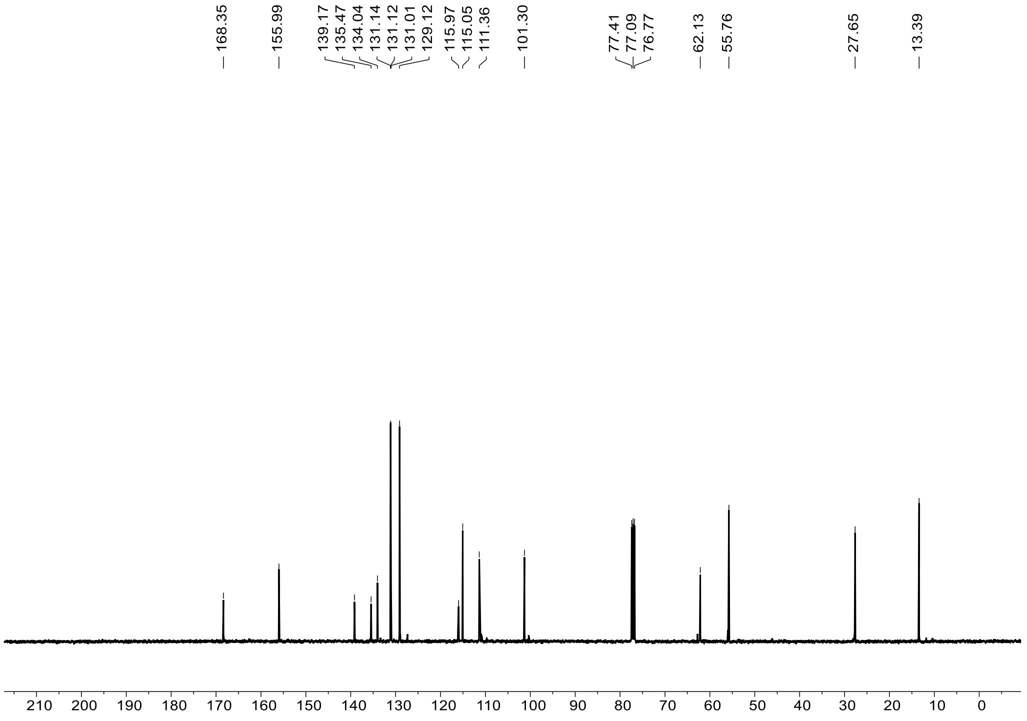

^{13}C NMR (101 MHz, $CDCl_3$)

(R)-2,5,7,8-tetramethyl-2-((4R,8R)-4,8,12-trimethyltridecyl)chroman-6-yl 4-(bromomethyl)benzoate

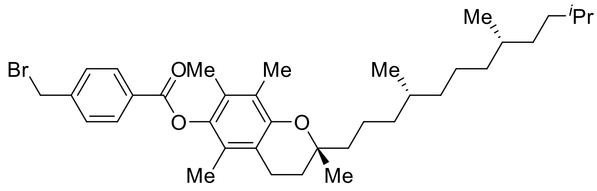

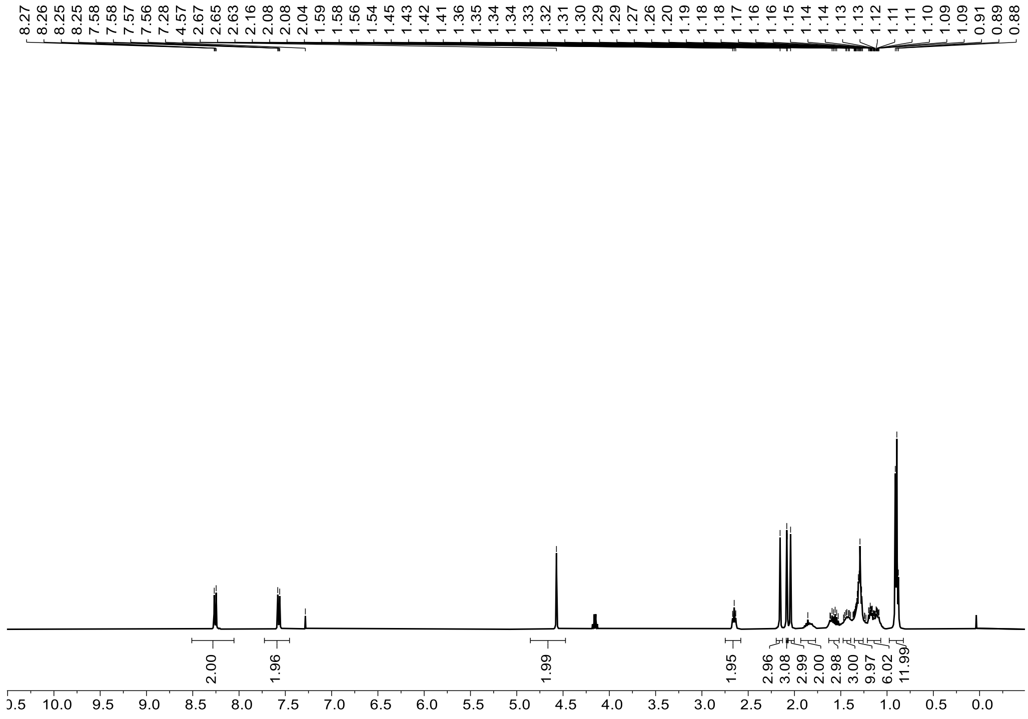

^{1}H NMR (400 MHz, $CDCl_3$)

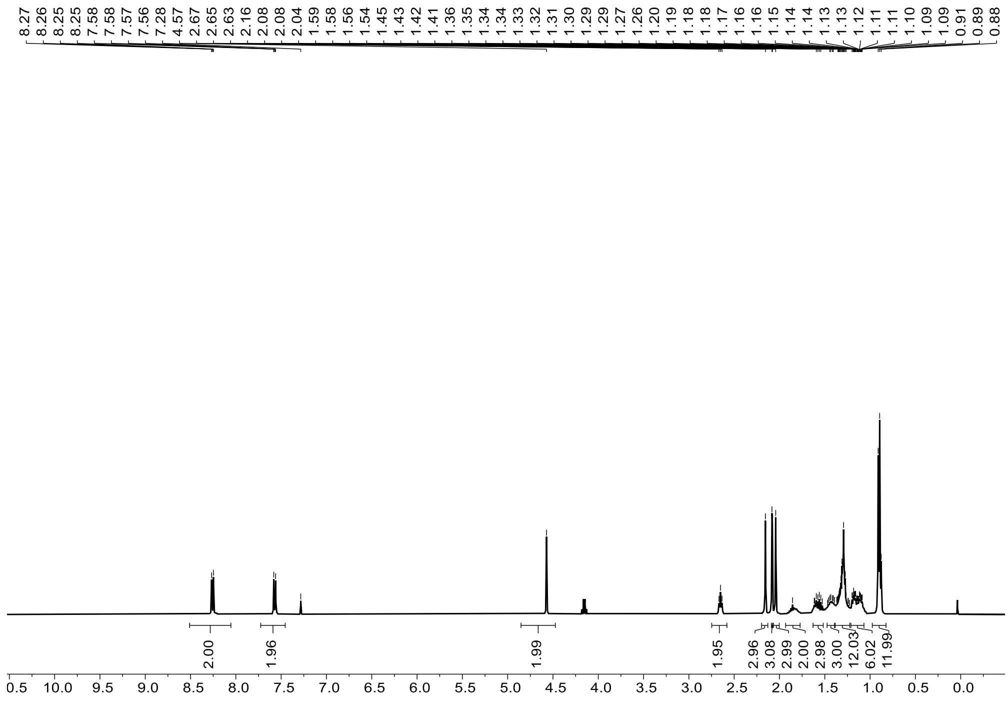

^{13}C NMR (101 MHz, $CDCl_3$)

methyl (S)-2-(1,3-dibenzyl-2-oxoindolin-3-yl)acetate (**4a**)

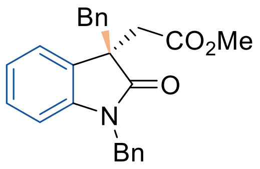

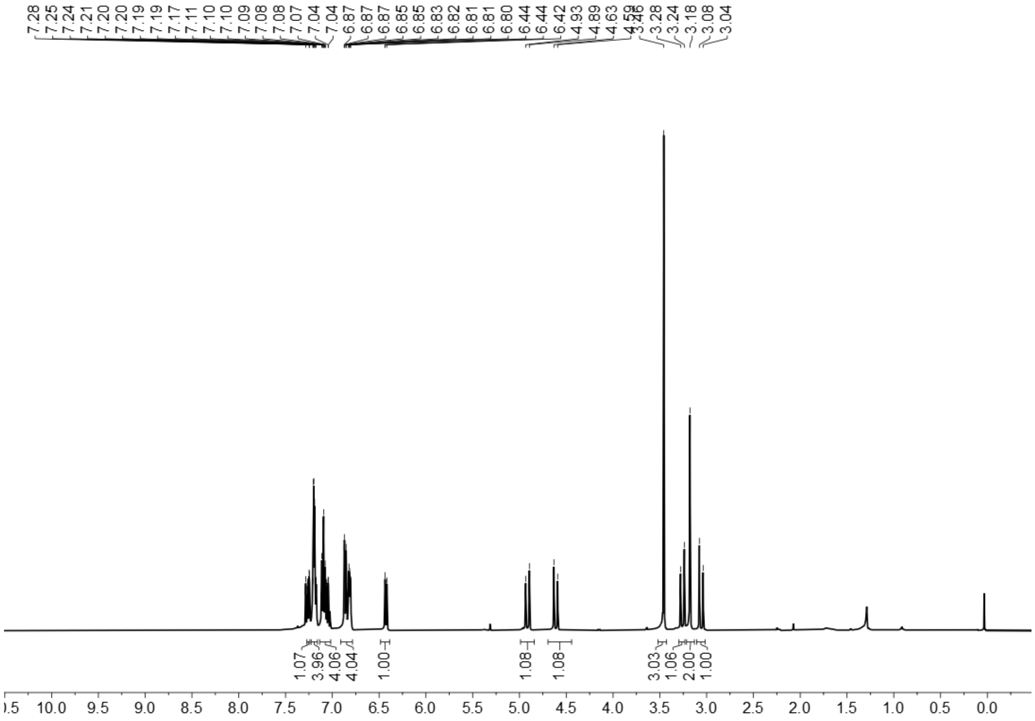

^{1}H NMR (400 MHz, $CDCl_3$)

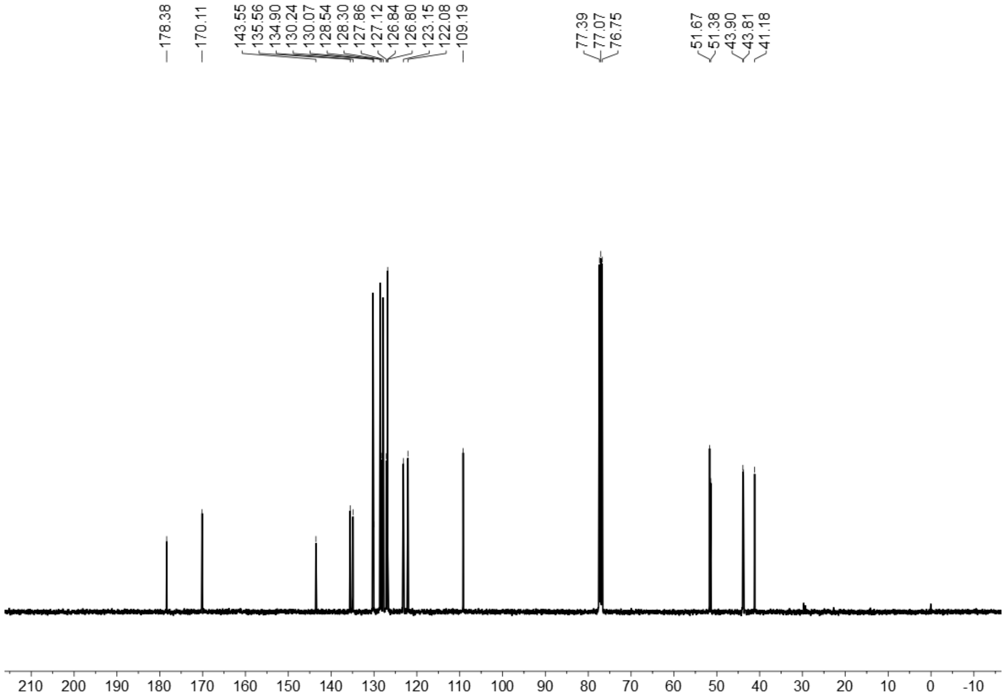

^{13}C NMR (101 MHz, $CDCl_3$)

methyl (S)-2-(1,3-dibenzyl-5-fluoro-2-oxoindolin-3-yl)acetate (**4b**)

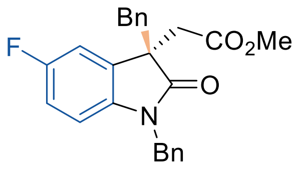

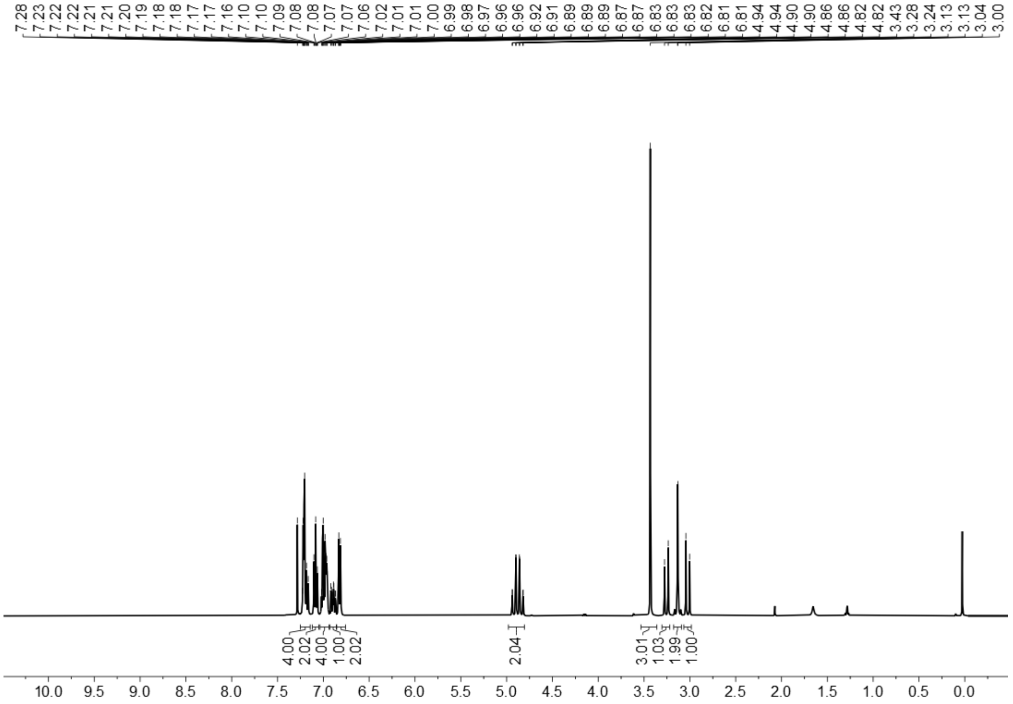

^{1}H NMR (400 MHz, $CDCl_3$)

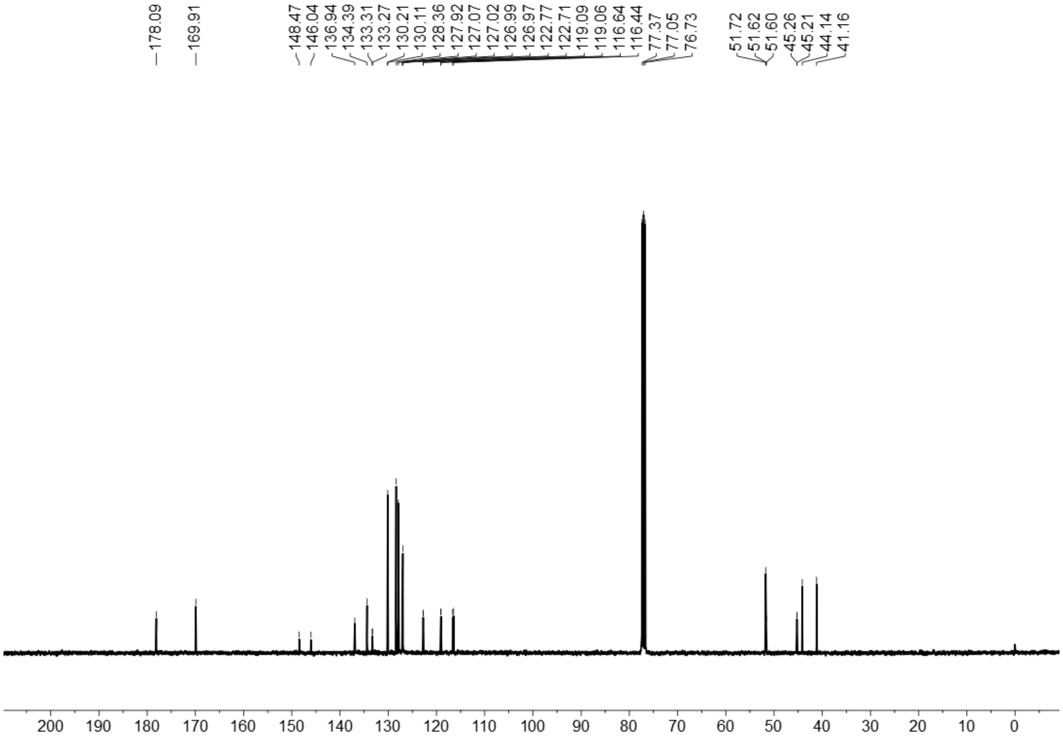

^{13}C NMR (101 MHz, $CDCl_3$)

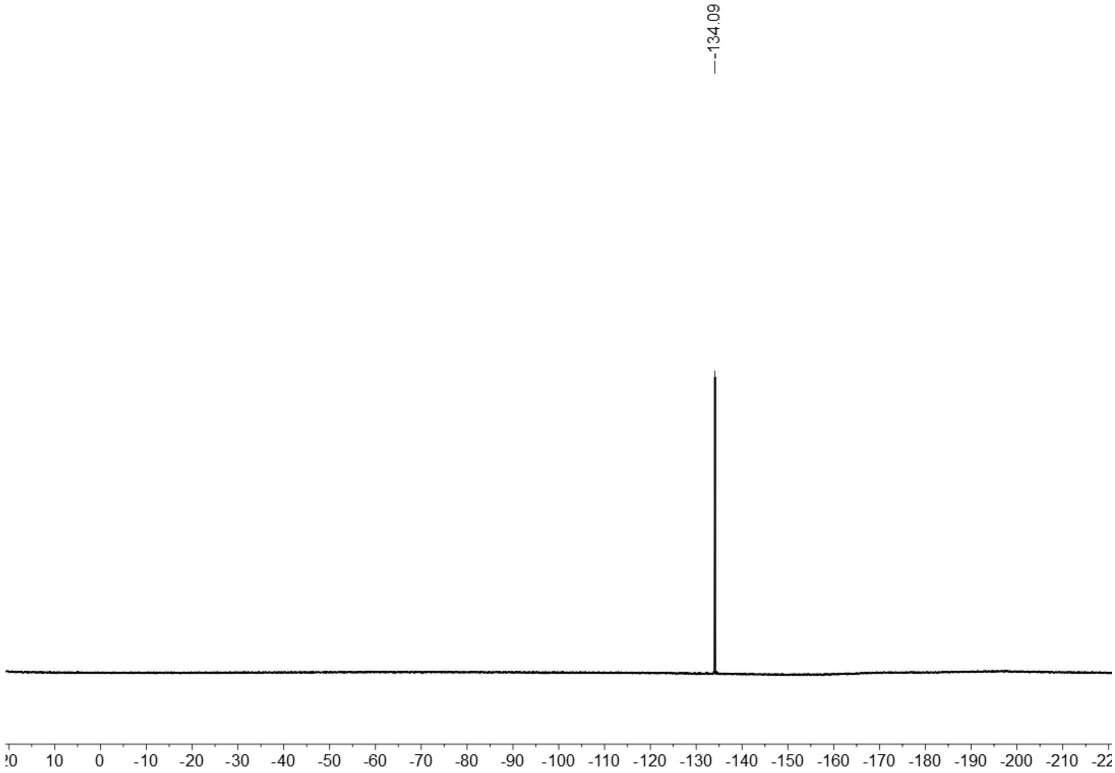

^{19}F NMR (376 MHz, $CDCl_3$)

methyl (S)-2-(1,3-dibenzyl-5-chloro-2-oxoindolin-3-yl)acetate (**4c**)

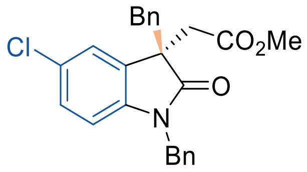

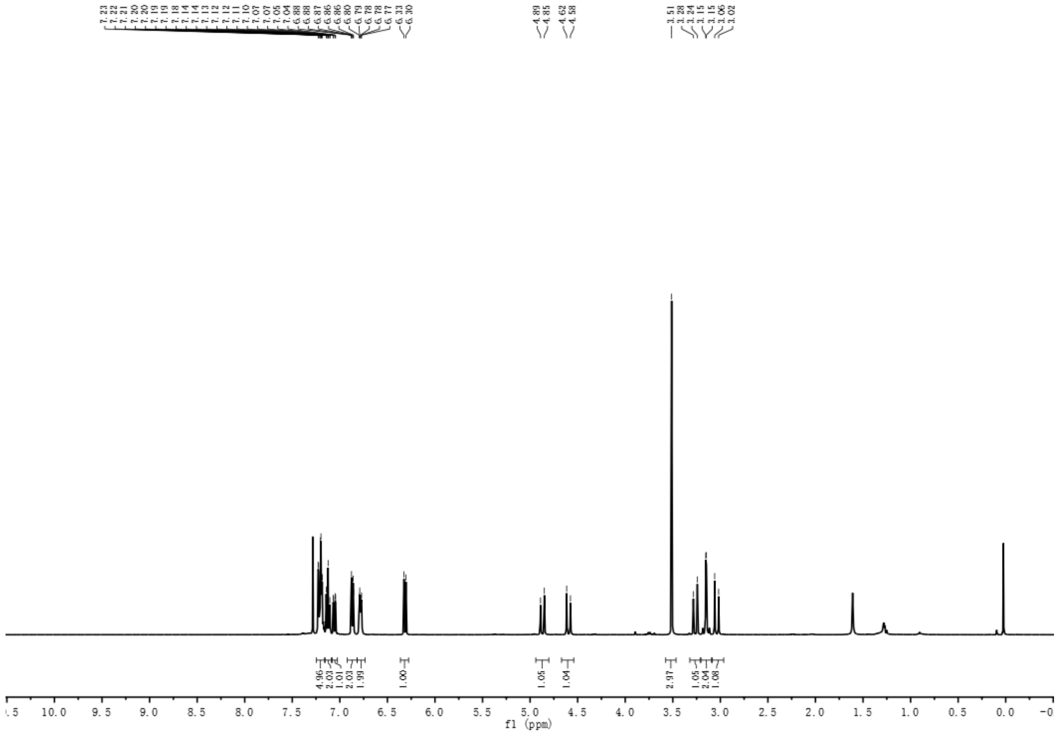

^{1}H NMR (400 MHz, $CDCl_3$)

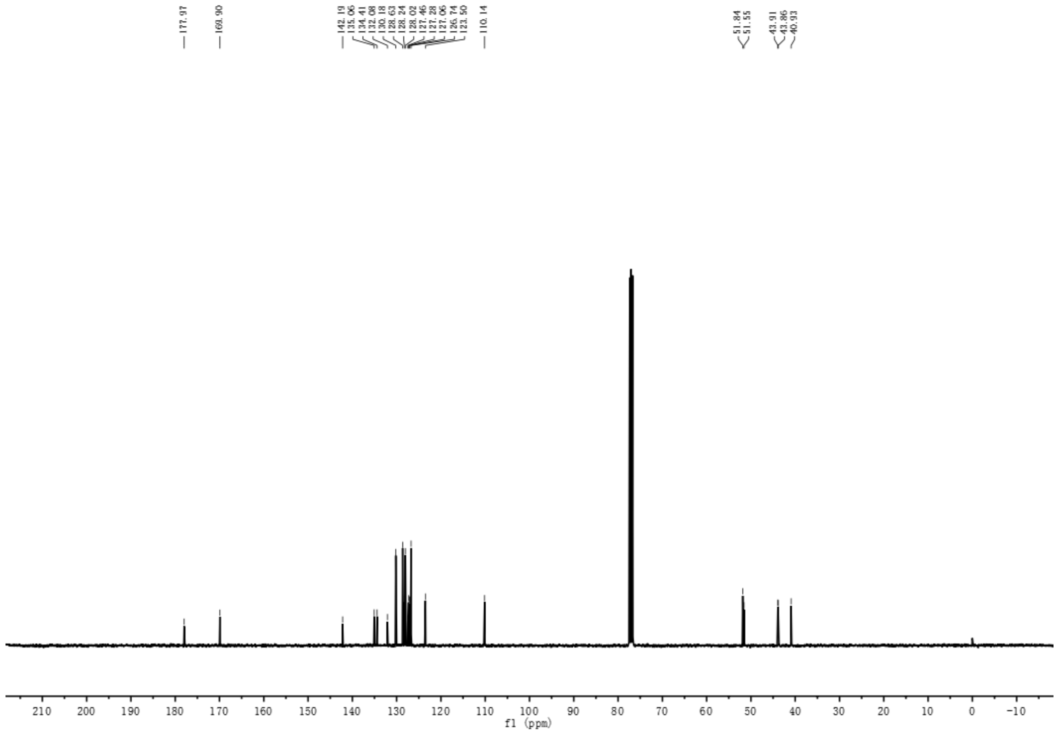

^{13}C NMR (101 MHz, $CDCl_3$)

methyl (S)-2-(1,3-dibenzyl-5-bromo-2-oxoindolin-3-yl)acetate (**4d**)

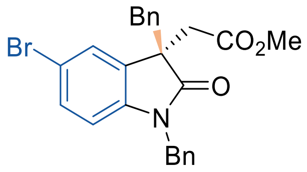

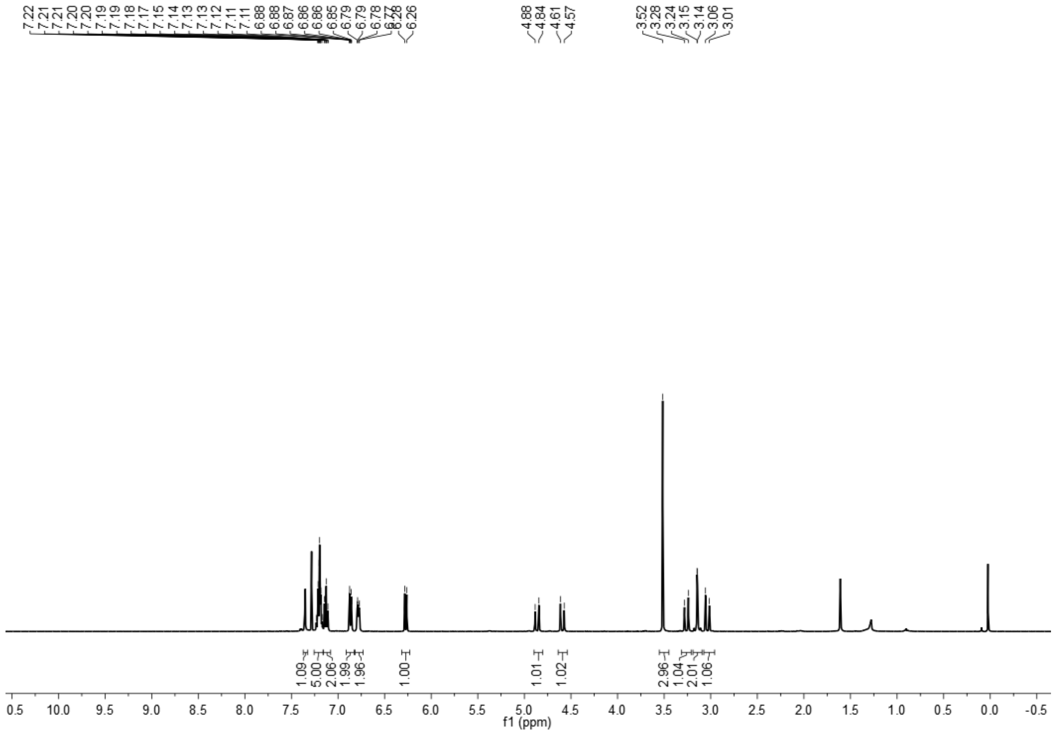

^{1}**H NMR** (400 MHz, $CDCl_3$)

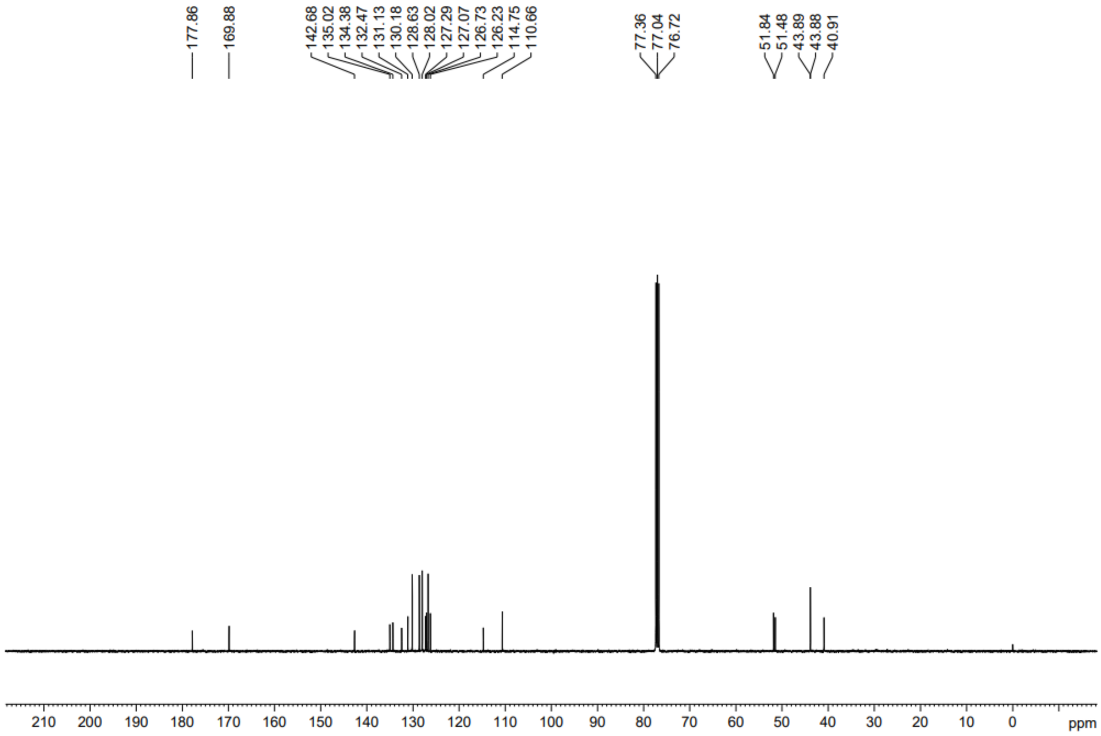

^{13}**C NMR** (101 MHz, $CDCl_3$)

methyl (S)-2-(1,3-dibenzyl-2-oxo-5-(trifluoromethoxy)indolin-3-yl)acetate (**4e**)

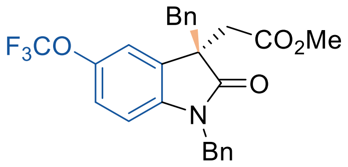

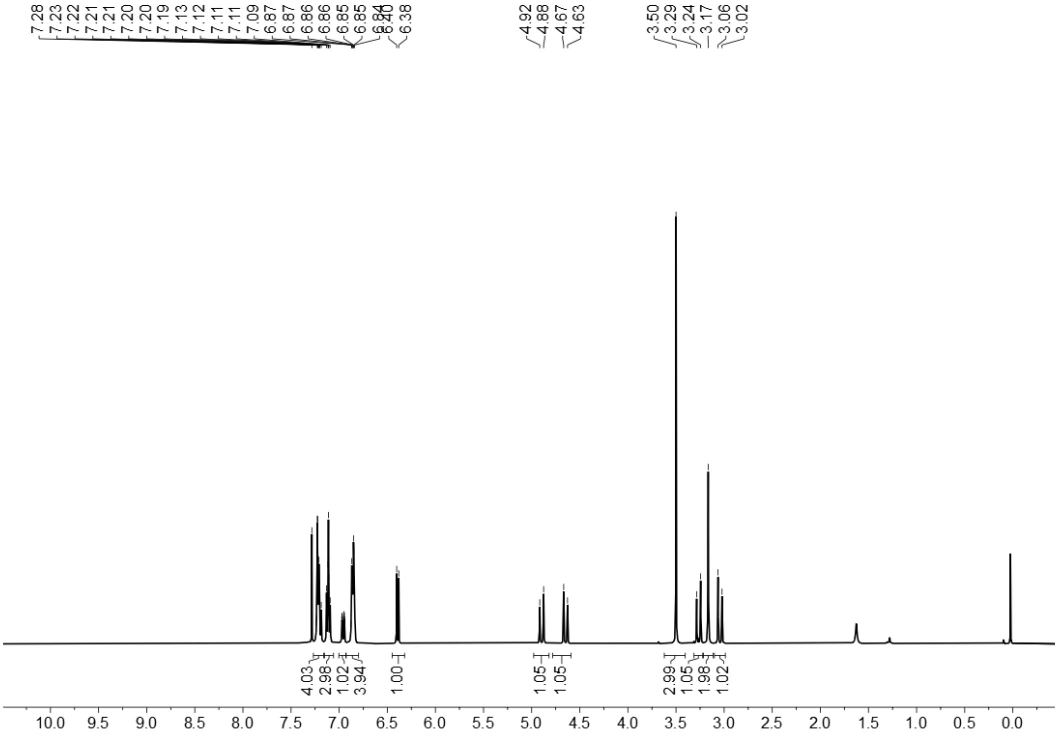

^{1}H NMR (400 MHz, $CDCl_3$)

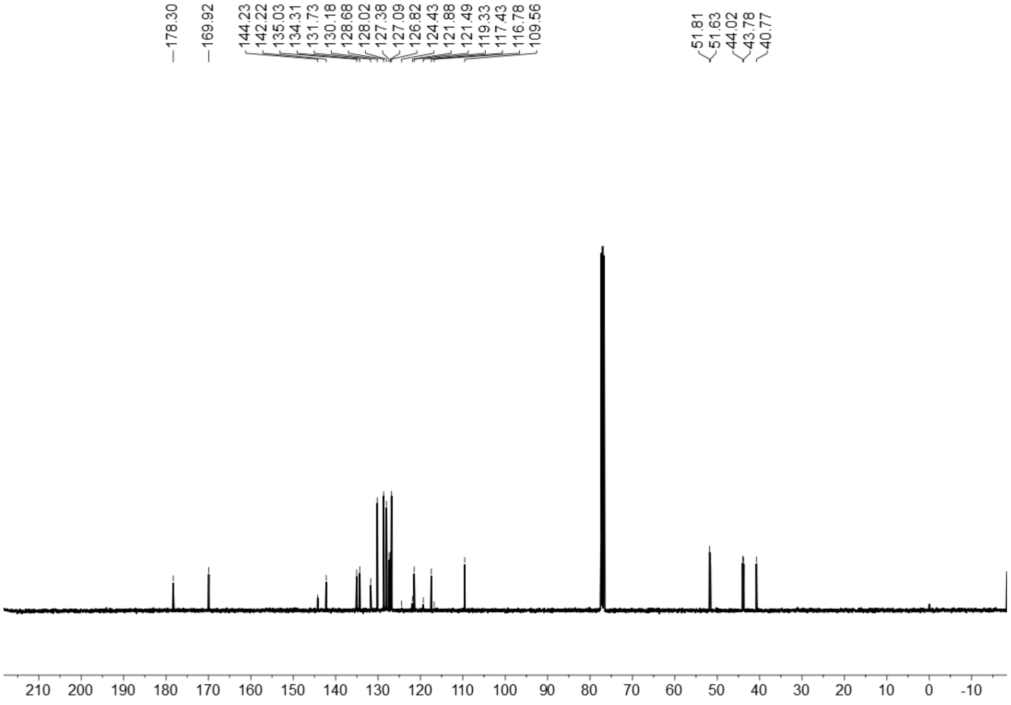

^{13}C NMR (101 MHz, $CDCl_3$)

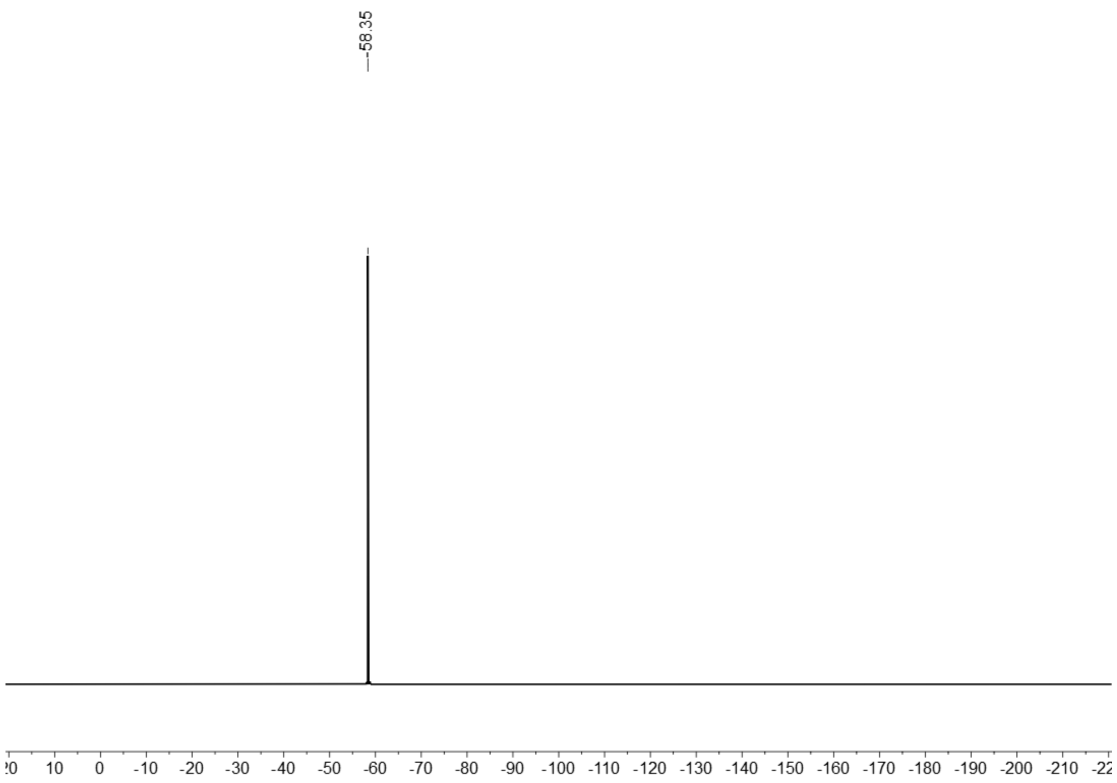

^{19}F NMR (376 MHz, $CDCl_3$)

methyl (S)-2-(1,3-dibenzyl-5-methoxy-2-oxoindolin-3-yl)acetate (**4f**)

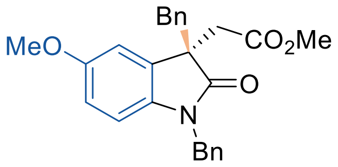

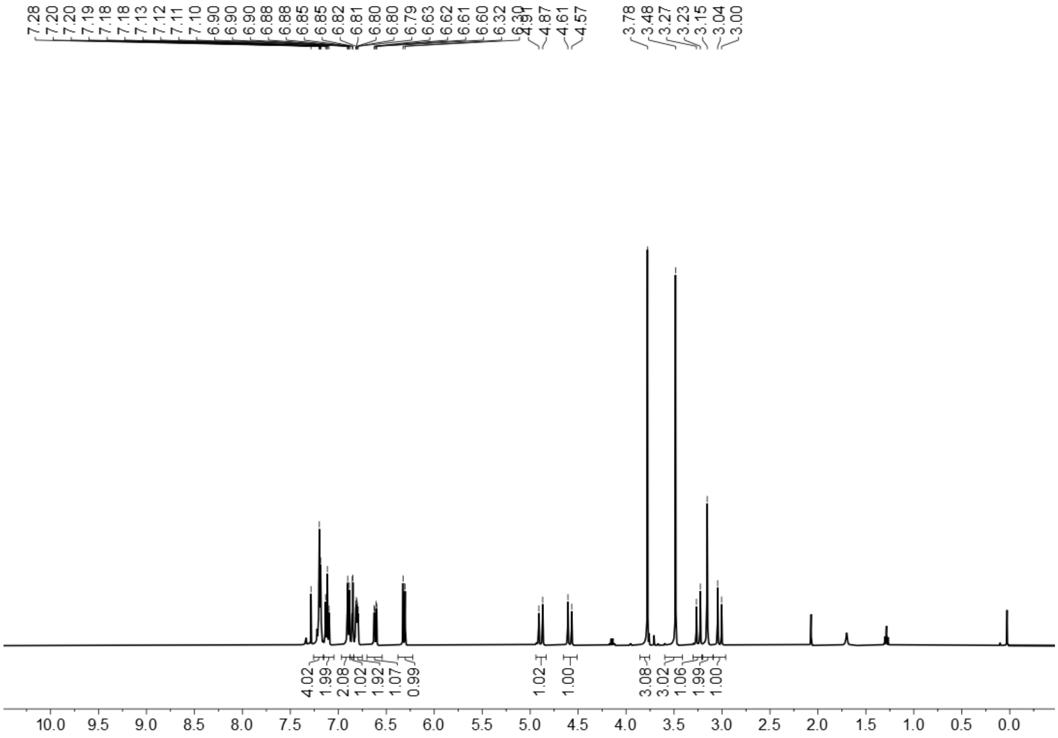

^{1}H NMR (400 MHz, CDCl$_3$)

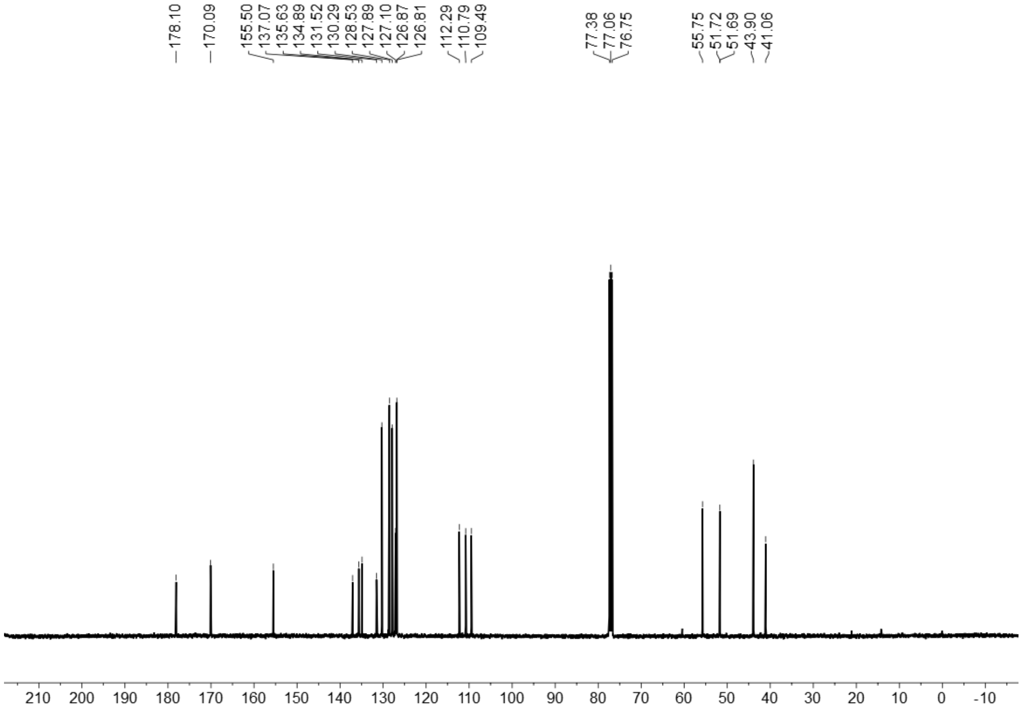

^{13}C NMR (101 MHz, CDCl$_3$)

methyl (S)-2-(1,3-dibenzyl-5-methyl-2-oxoindolin-3-yl)acetate (**4g**)

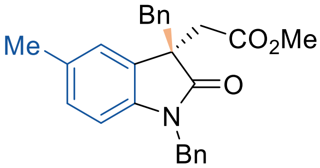

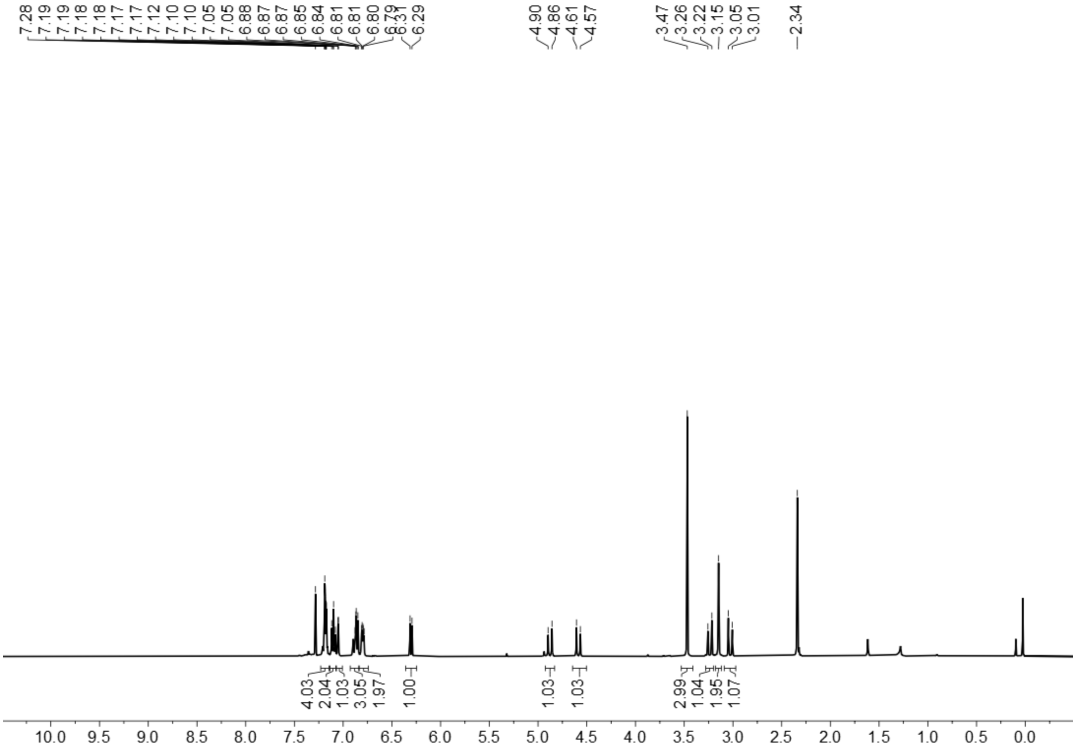

^{1}H NMR (400 MHz, $CDCl_3$)

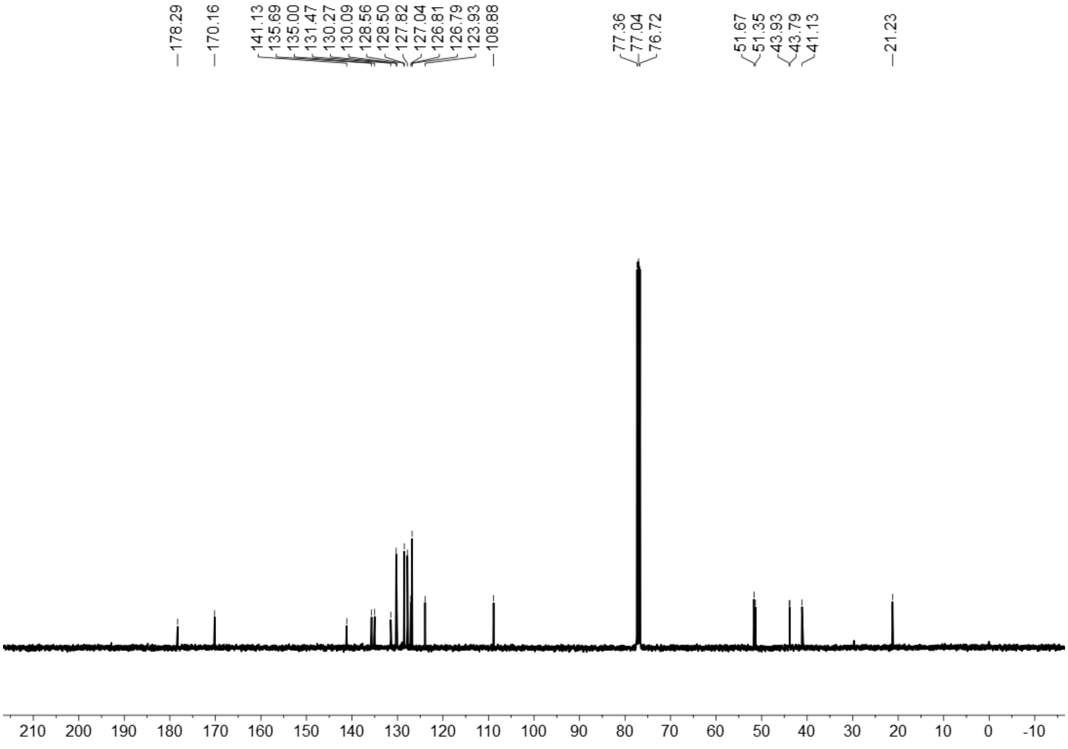

^{13}C NMR (101 MHz, $CDCl_3$)

methyl (S)-2-(1,3-dibenzyl-7-methyl-2-oxoindolin-3-yl)acetate (**4h**)

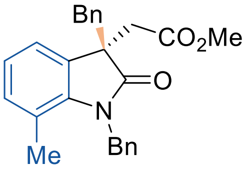

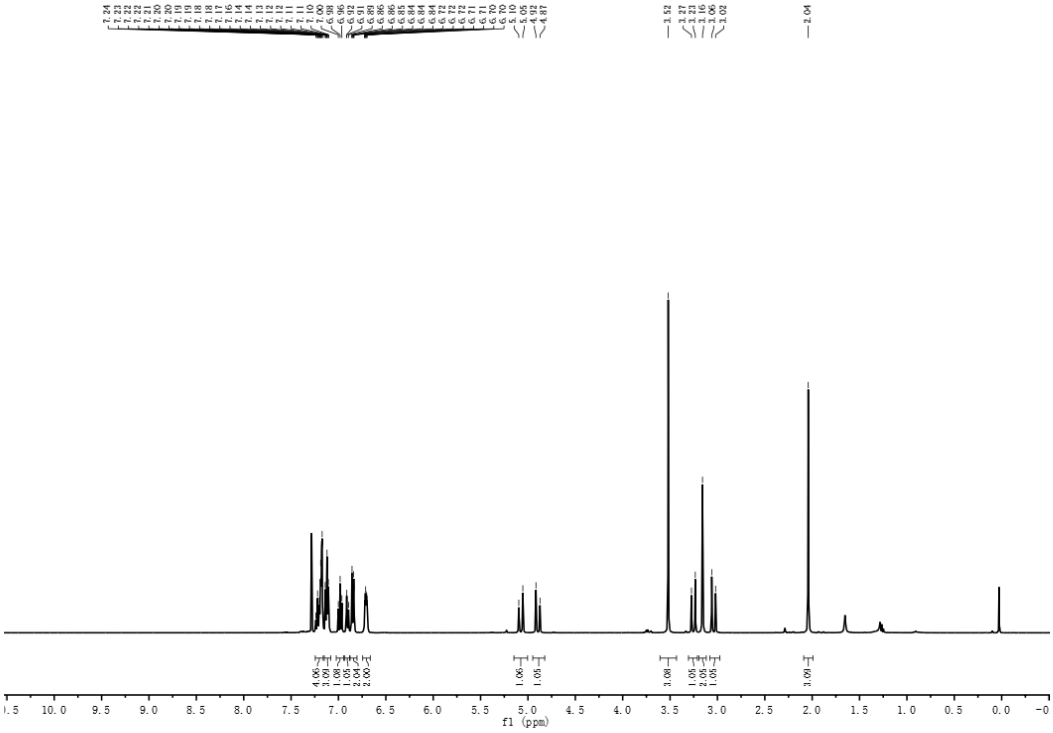

^{1}H NMR (400 MHz, $CDCl_3$)

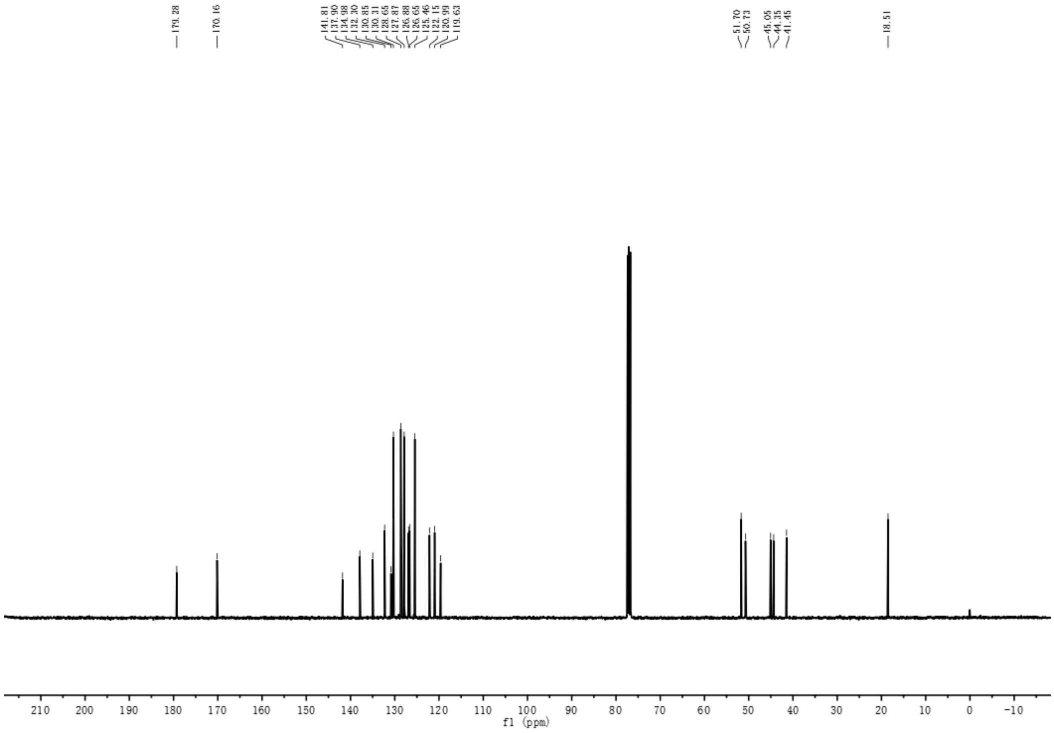

^{13}C NMR (101 MHz, $CDCl_3$)

methyl (S)-2-(1,3-dibenzyl-7-fluoro-2-oxoindolin-3-yl)acetate (**4i**)

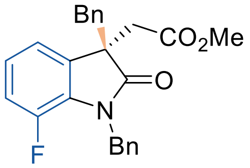

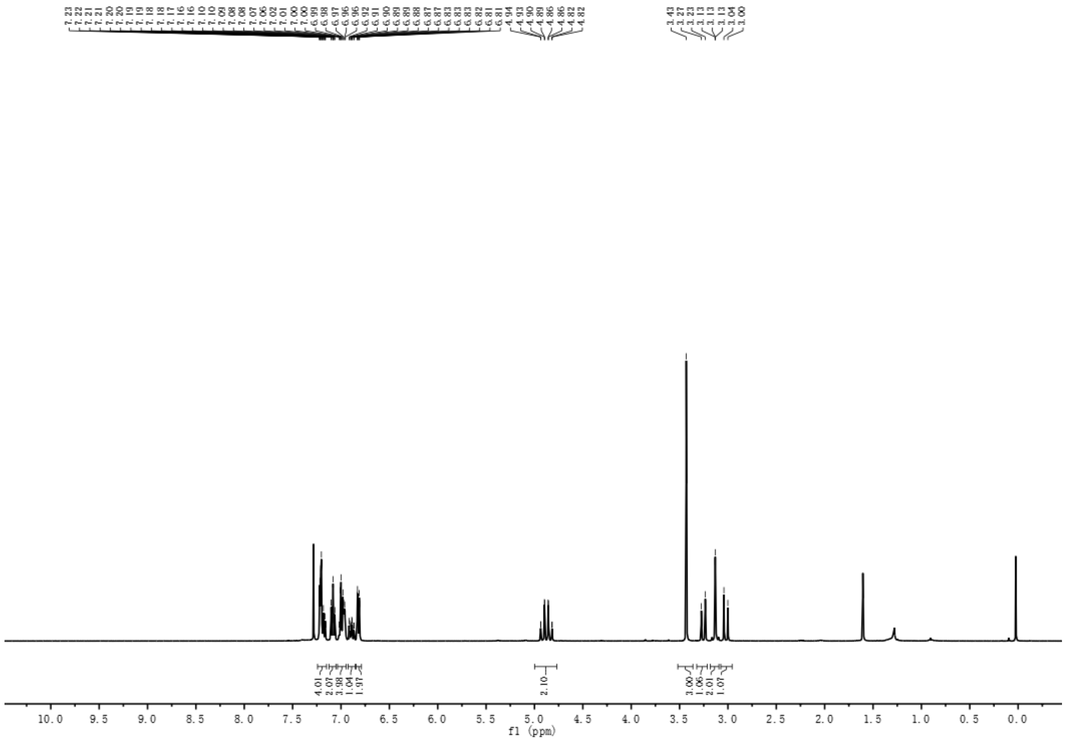

^{1}H NMR (400 MHz, $CDCl_3$)

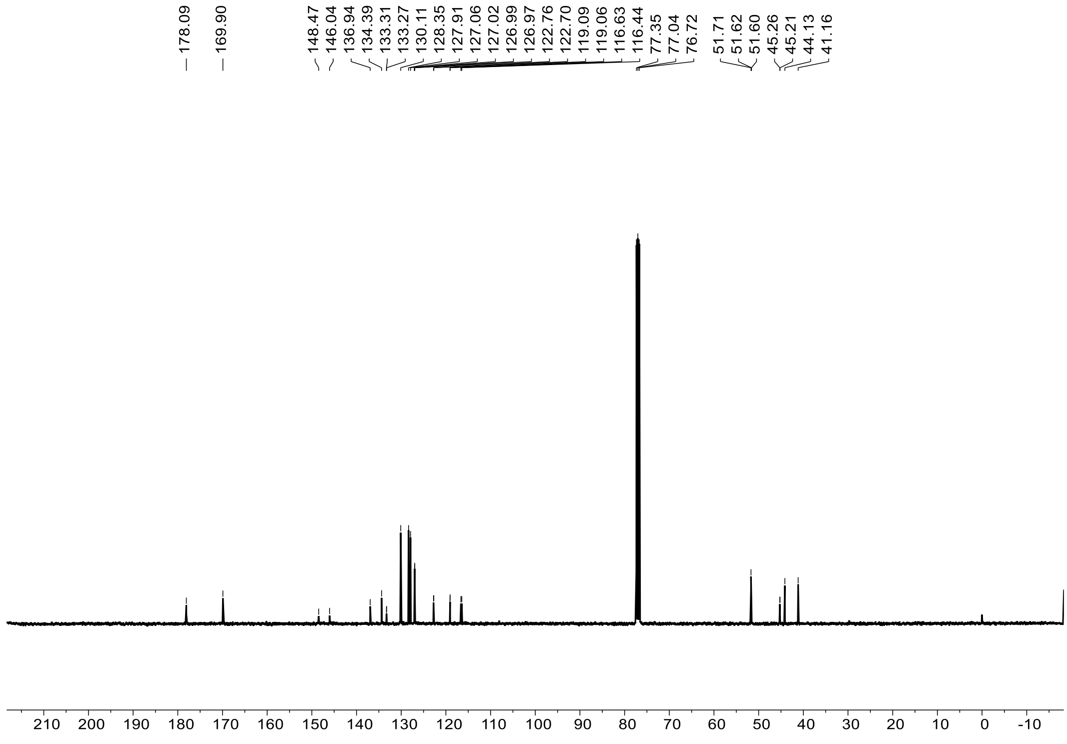

^{13}C NMR (101 MHz, $CDCl_3$)

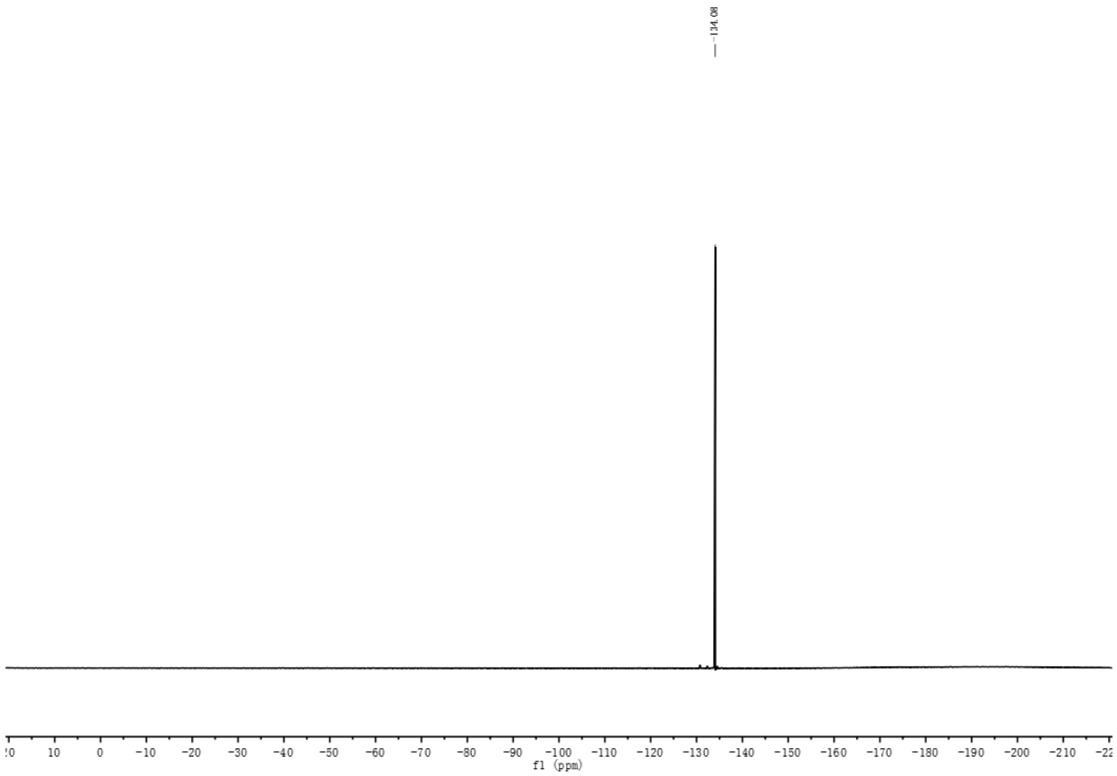

^{19}F NMR (376 MHz, $CDCl_3$)

methyl (S)-2-(1,3-dibenzyl-6-chloro-2-oxoindolin-3-yl)acetate (**4j**)

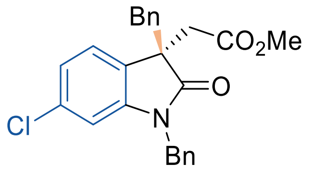

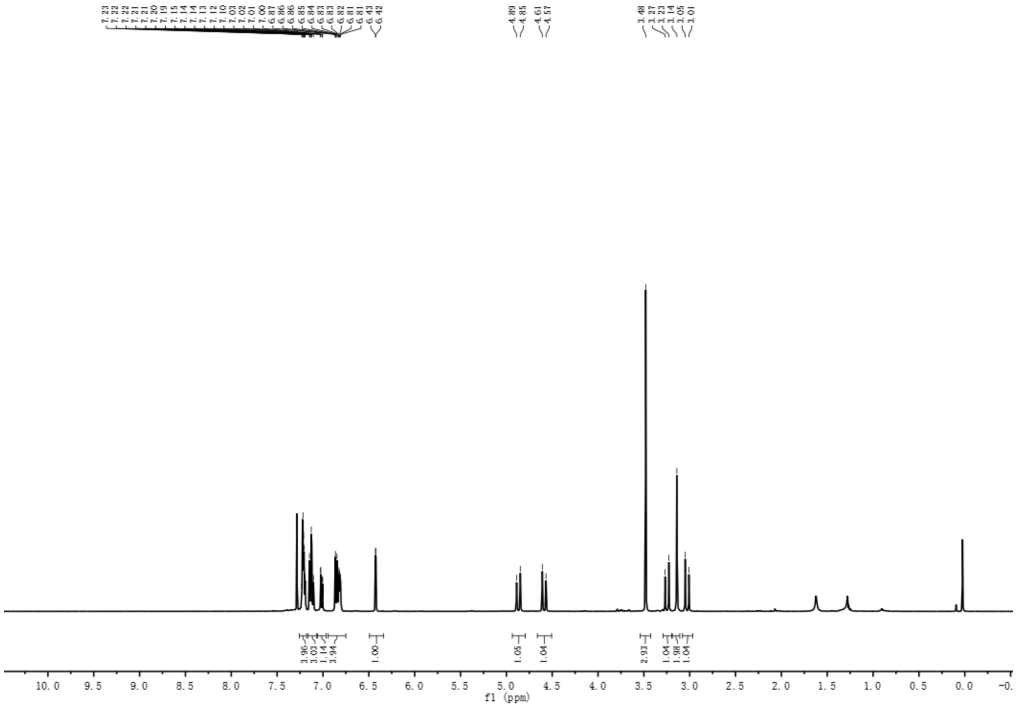

^{1}H NMR (400 MHz, $CDCl_3$)

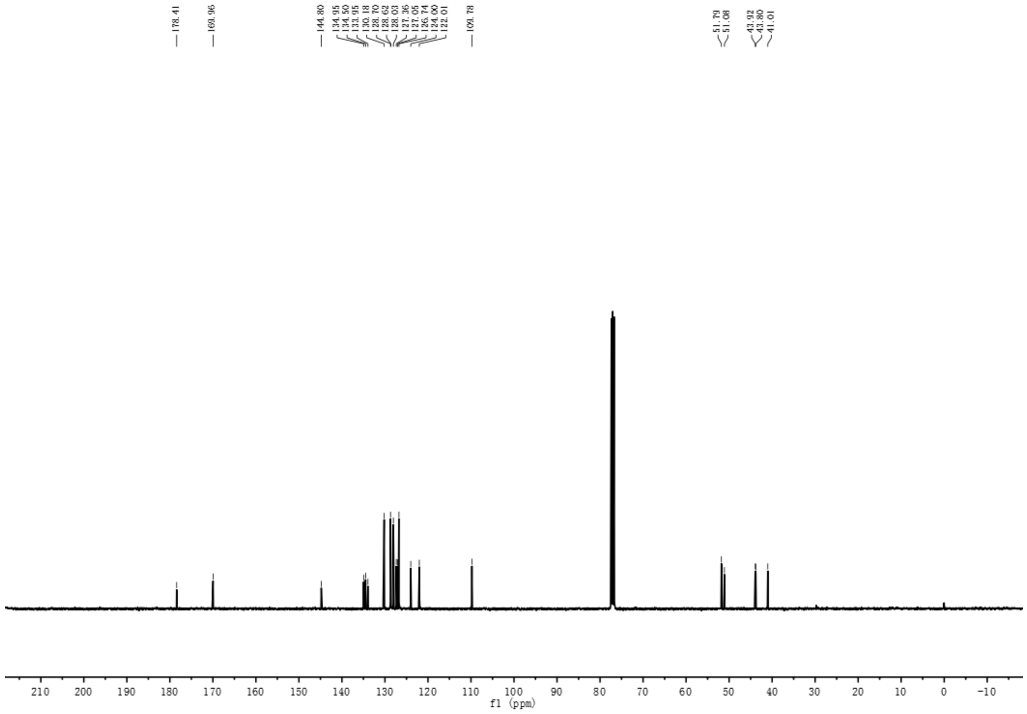

^{13}C NMR (101 MHz, $CDCl_3$)

methyl (S)-2-(1,3-dibenzyl-7-bromo-2-oxoindolin-3-yl)acetate (**4k**)

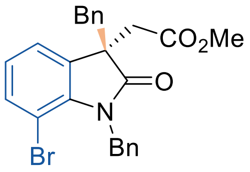

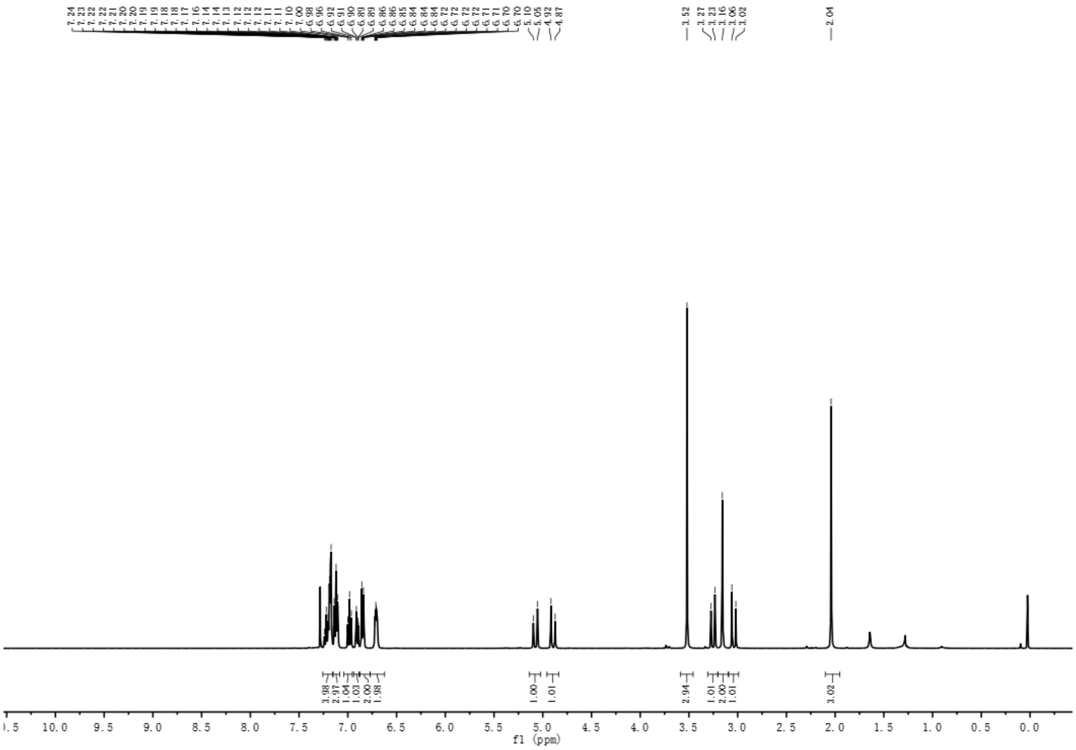

^{1}H NMR (400 MHz, $CDCl_3$)

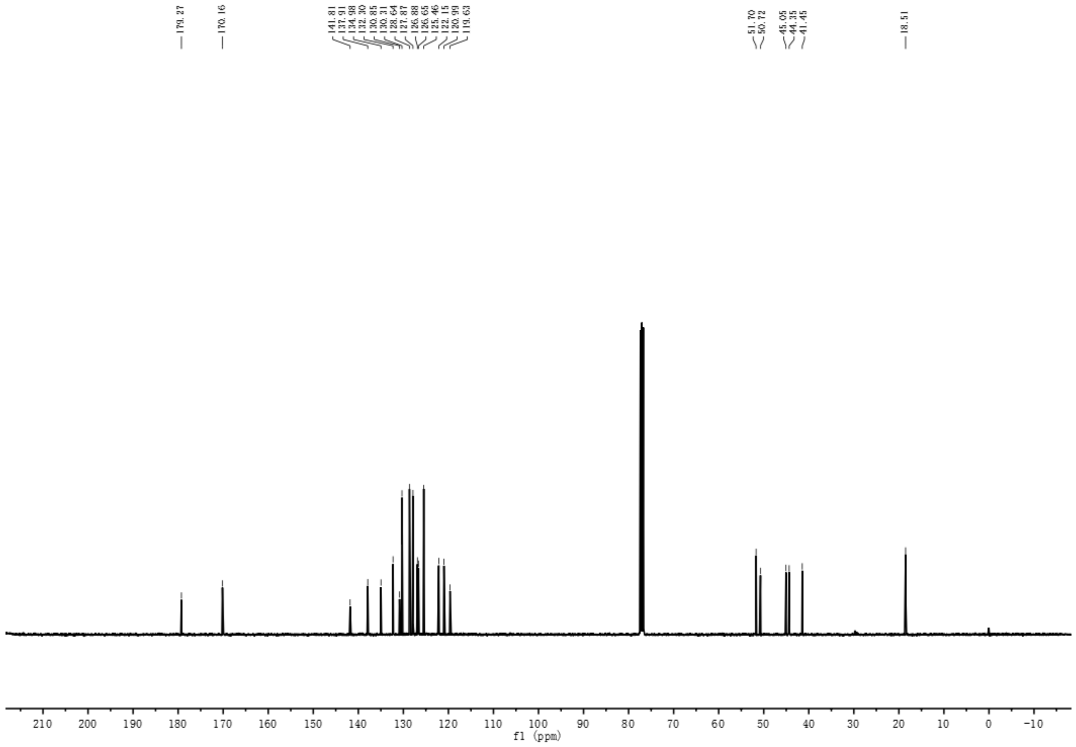

^{13}C NMR (101 MHz, $CDCl_3$)

methyl (S)-2-(1,3-dibenzyl-4,6-difluoro-2-oxoindolin-3-yl)acetate (**4l**)

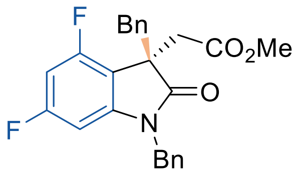

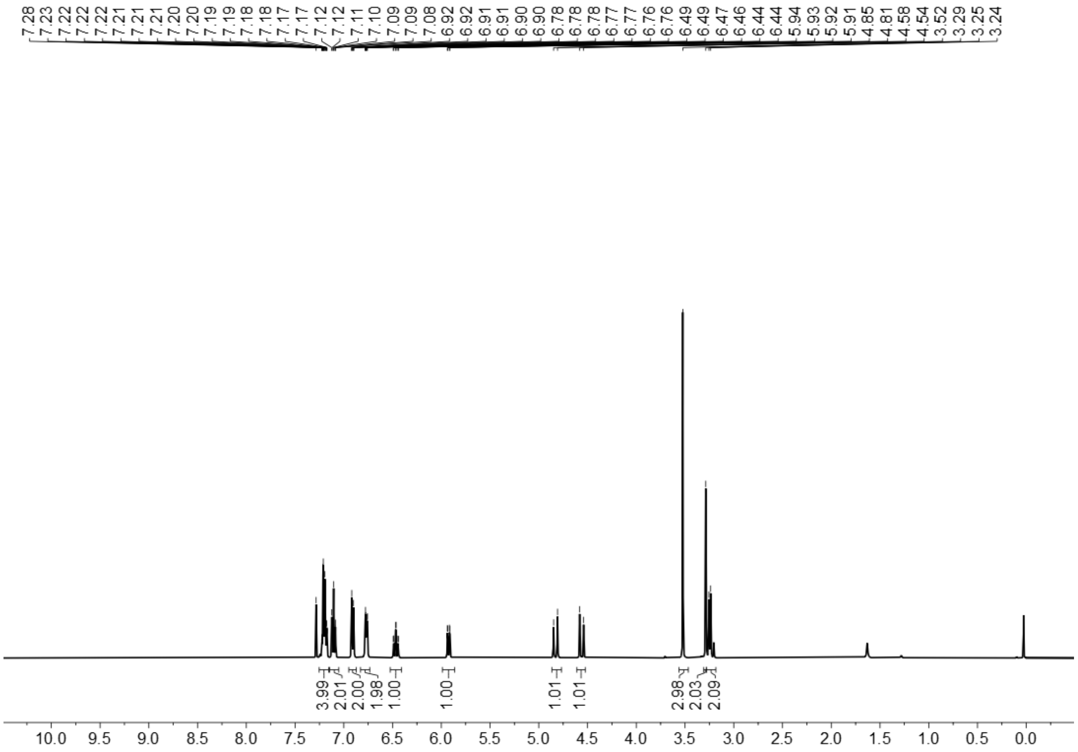

^{1}H NMR (400 MHz, $CDCl_3$)

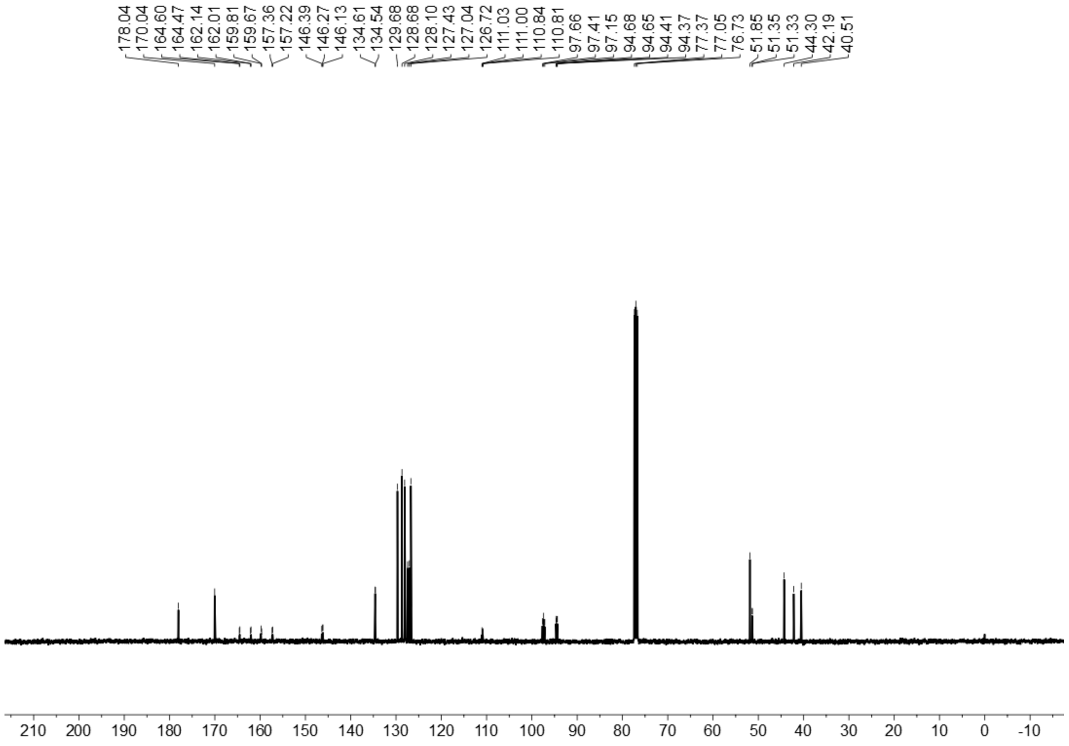

^{13}C NMR (101 MHz, $CDCl_3$)

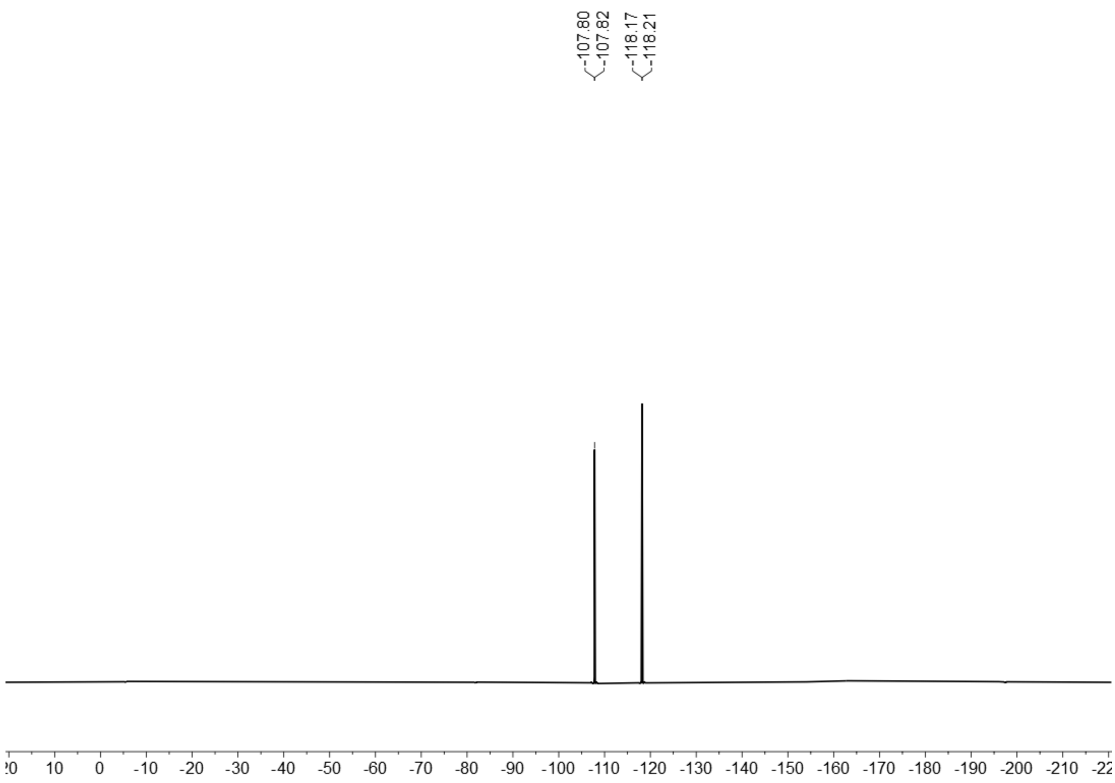

^{19}F NMR (376 MHz, $CDCl_3$)

methyl (S)-2-(1,3-dibenzyl-4,7-dichloro-2-oxoindolin-3-yl)acetate (**4m**)

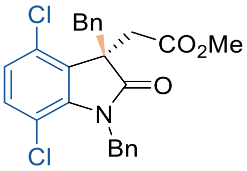

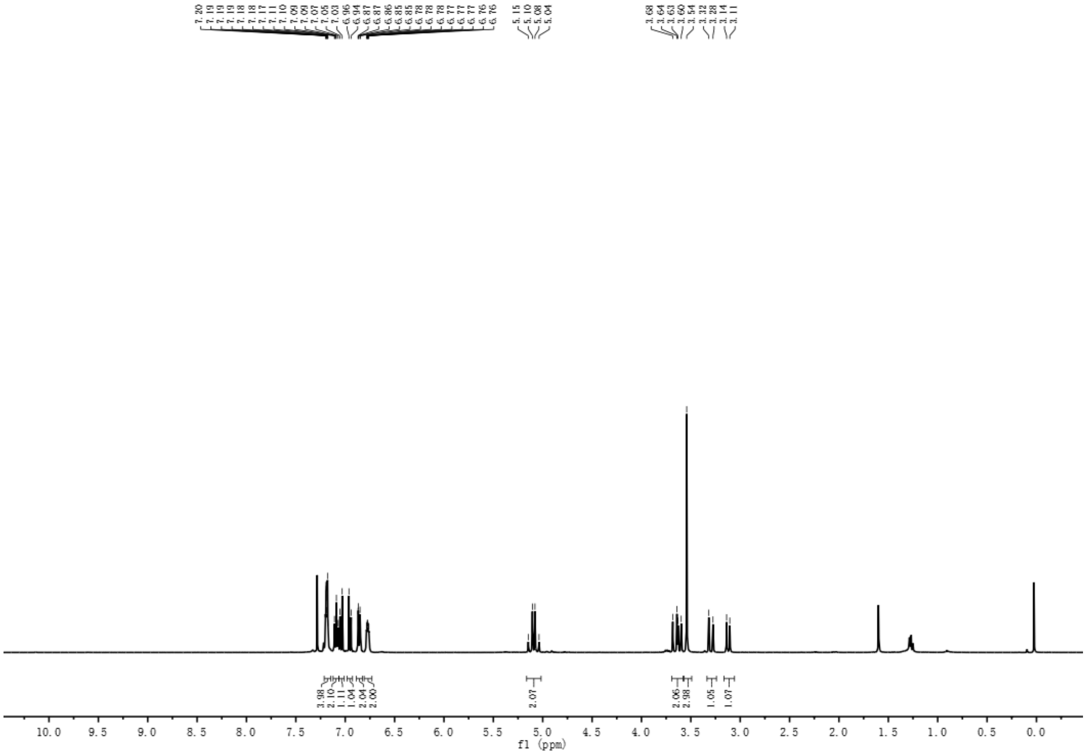

^{1}H NMR (400 MHz, $CDCl_3$)

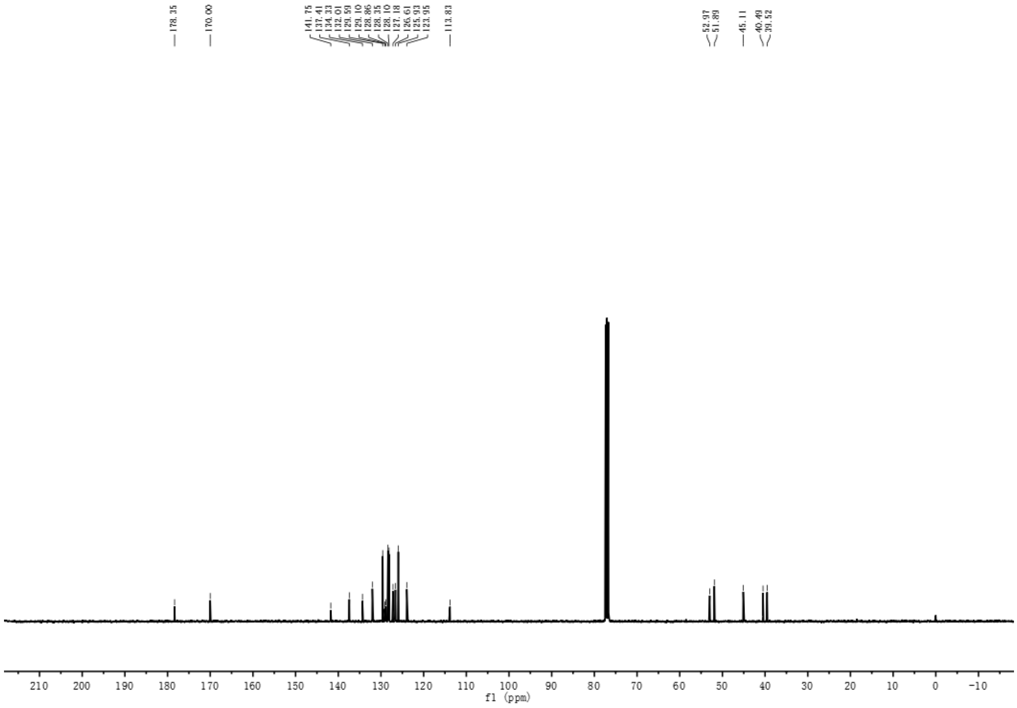

^{13}C NMR (101 MHz, $CDCl_3$)

methyl (S)-2-(1,3-dibenzyl-5,7-dimethyl-2-oxoindolin-3-yl)acetate (**4n**)

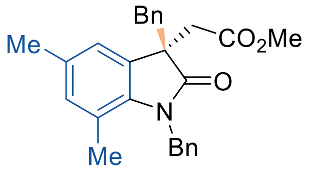

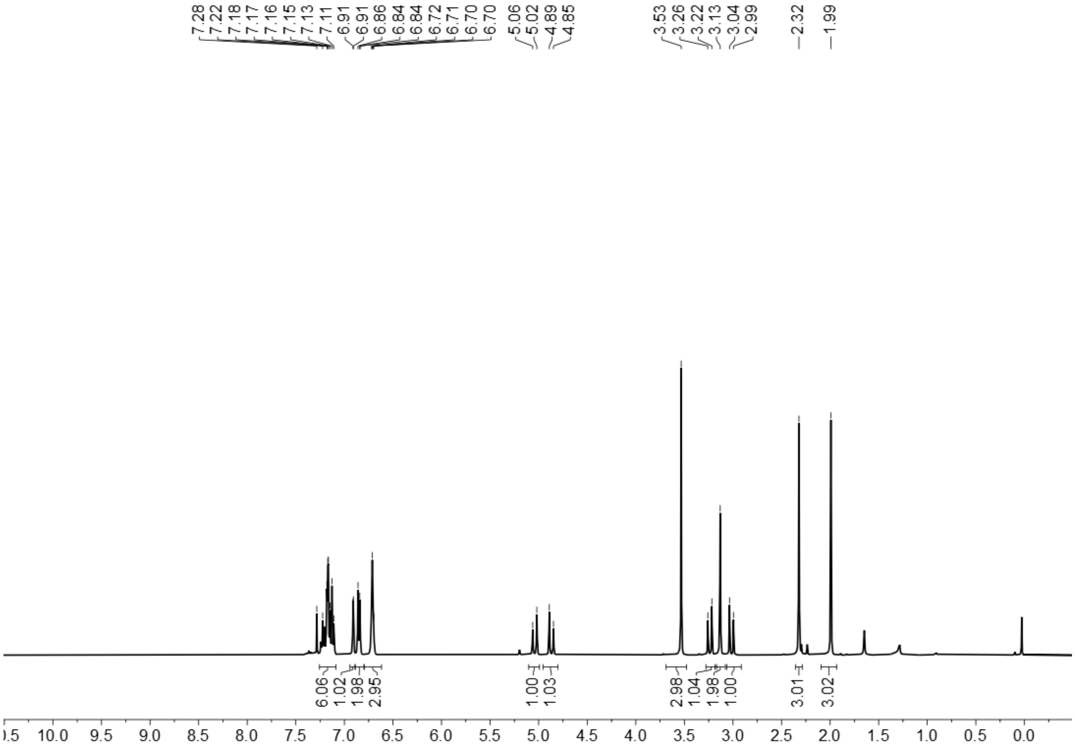

1H NMR (400 MHz, $CDCl_3$)

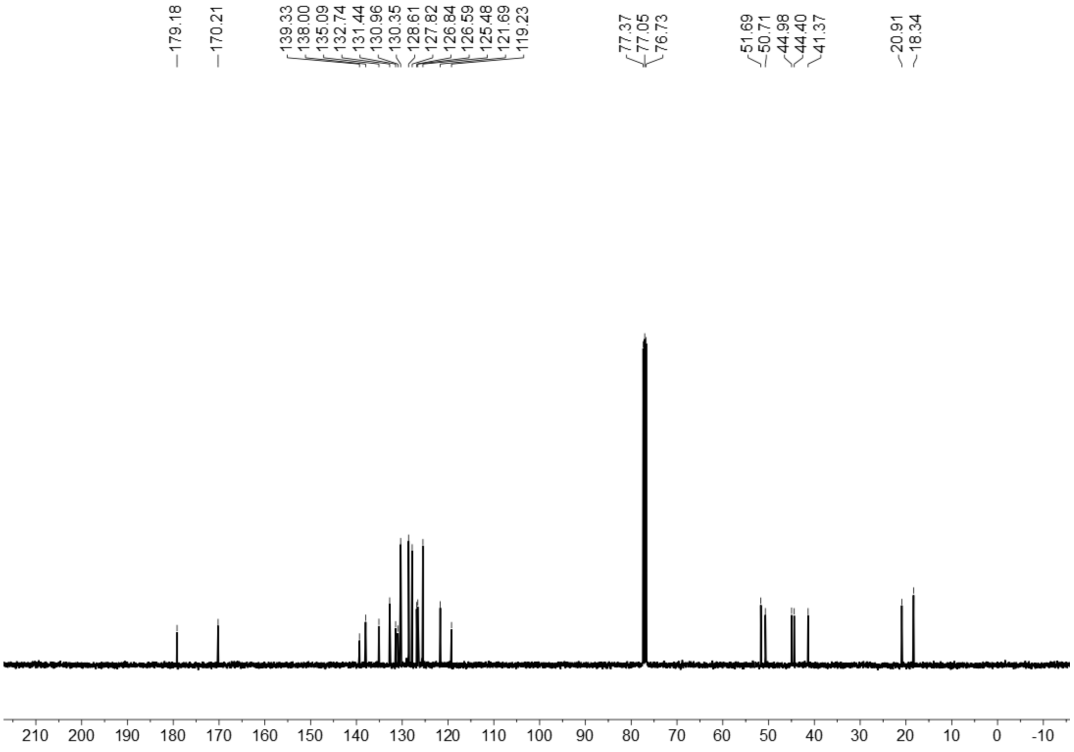

13C NMR (101 MHz, $CDCl_3$)

methyl (S)-2-(1,3-dibenzyl-5-chloro-7-methyl-2-oxoindolin-3-yl)acetate (**4o**)

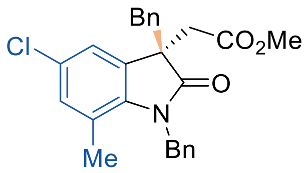

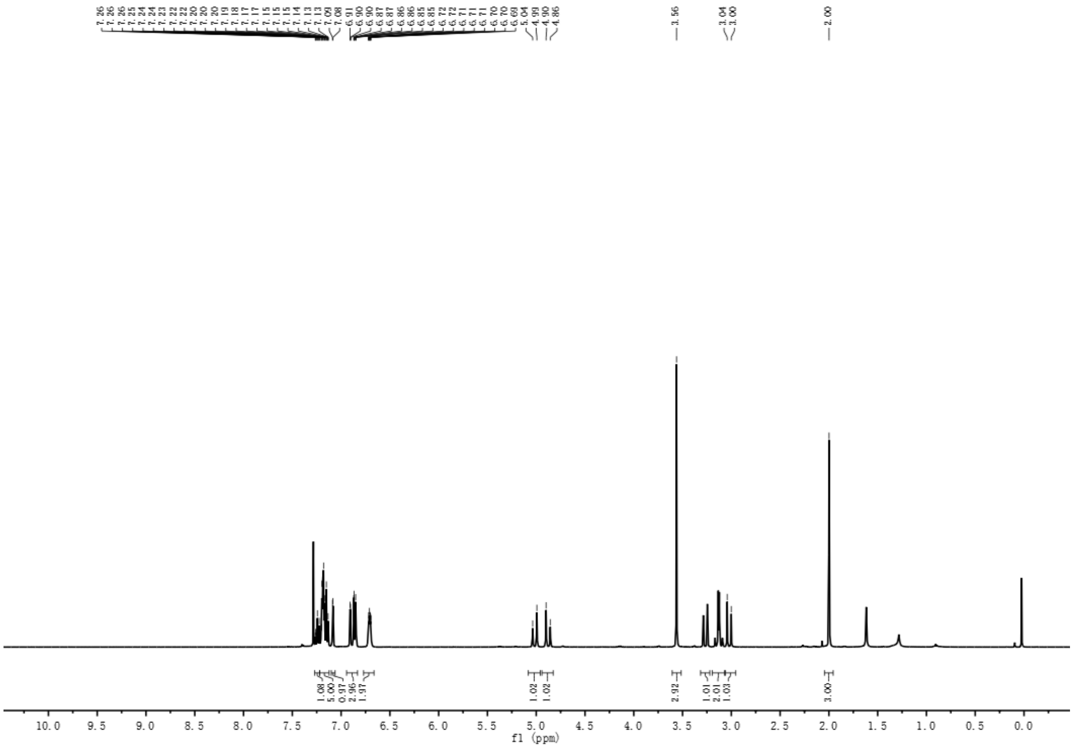

^{1}H NMR (400 MHz, $CDCl_3$)

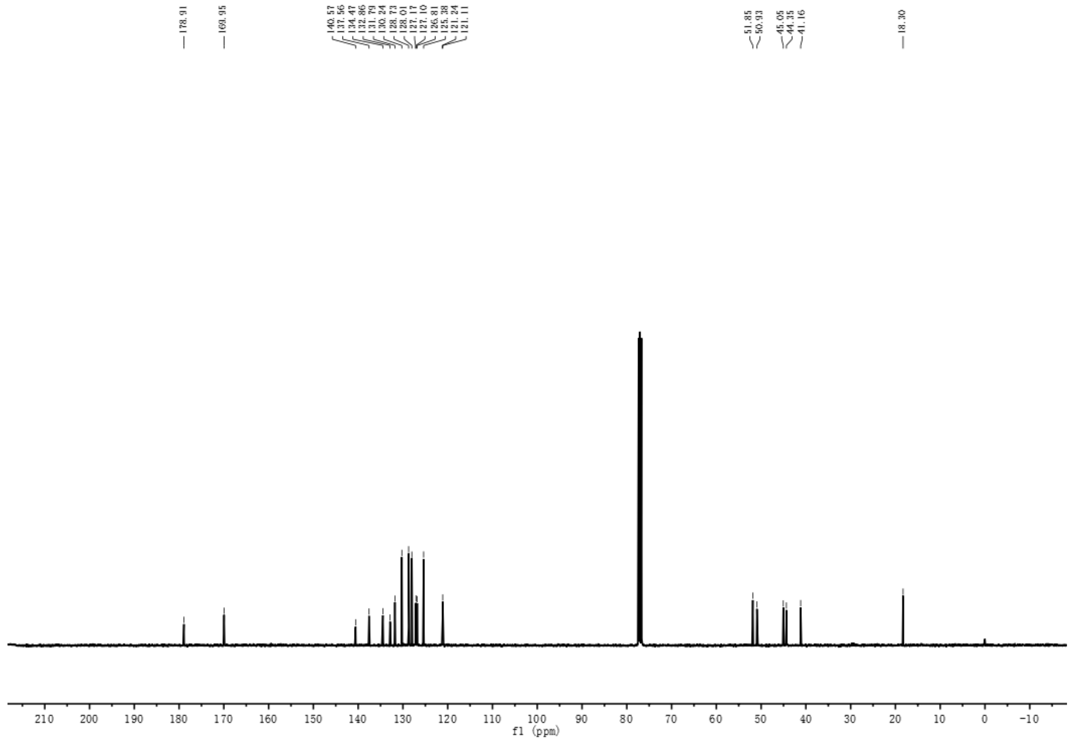

^{13}C NMR (101 MHz, $CDCl_3$)

methyl (S)-2-(3-benzyl-1-methyl-2-oxoindolin-3-yl)acetate (**4p**)

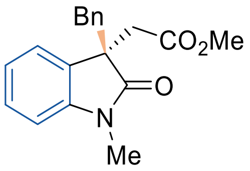

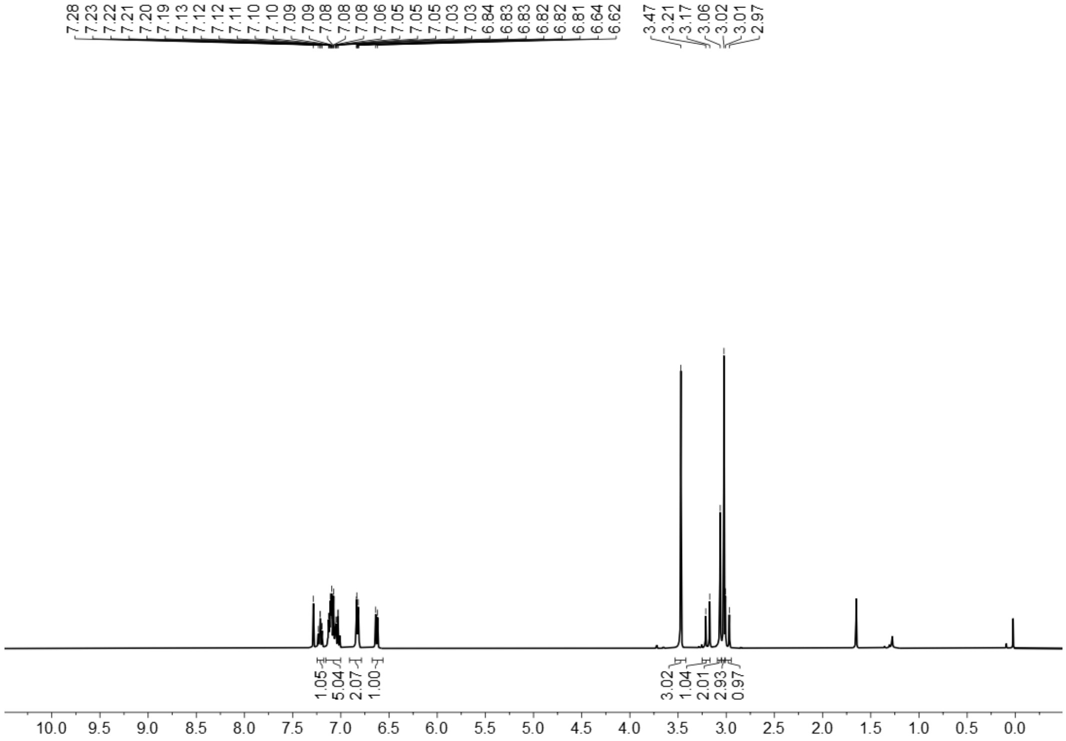

^{1}H NMR (400 MHz, $CDCl_3$)

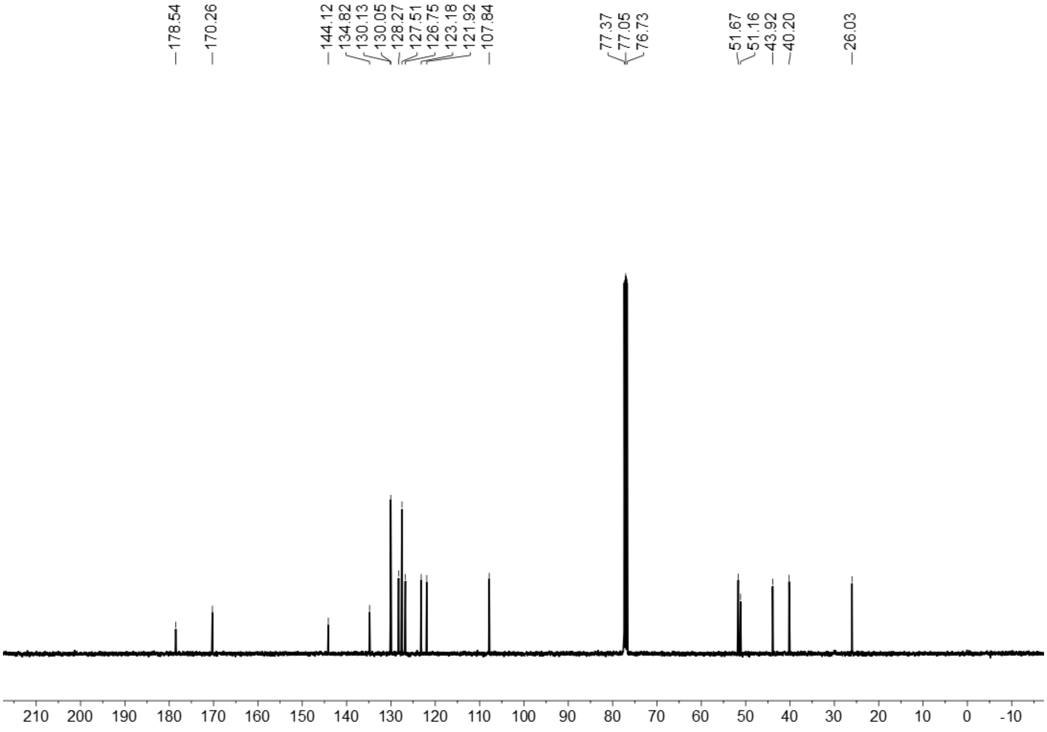

^{13}C NMR (101 MHz, $CDCl_3$)

ethyl (S)-2-(1,3-dibenzyl-4,7-dichloro-2-oxoindolin-3-yl)acetate (**4q**)

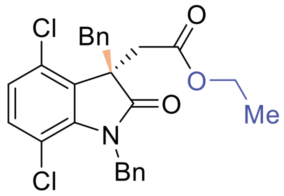

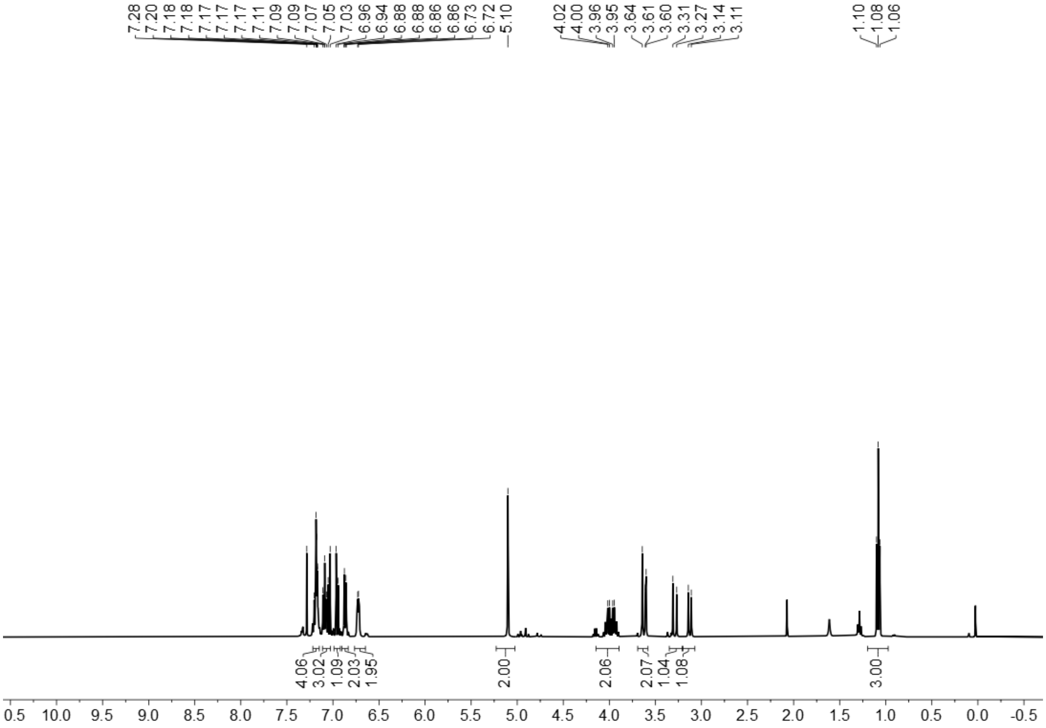

^{1}H NMR (400 MHz, $CDCl_3$)

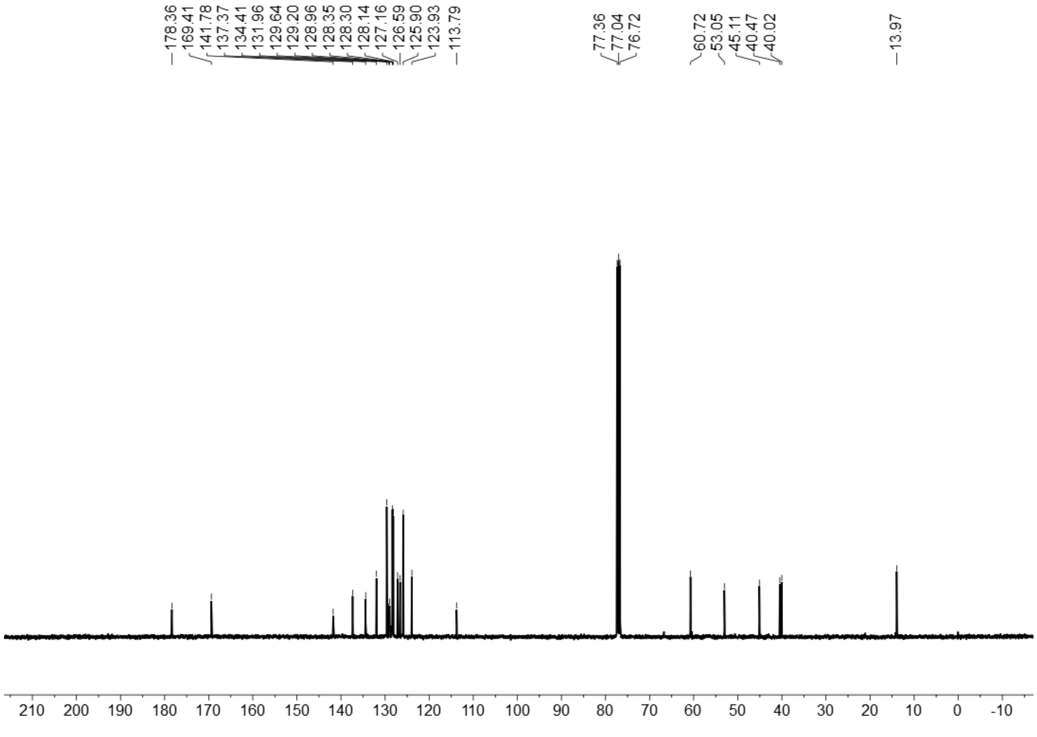

^{13}C NMR (101 MHz, $CDCl_3$)

benzyl (S)-2-(1,3-dibenzyl-4,7-dichloro-2-oxoindolin-3-yl)acetate (**4r**)

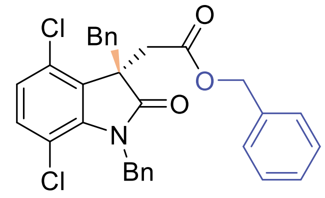

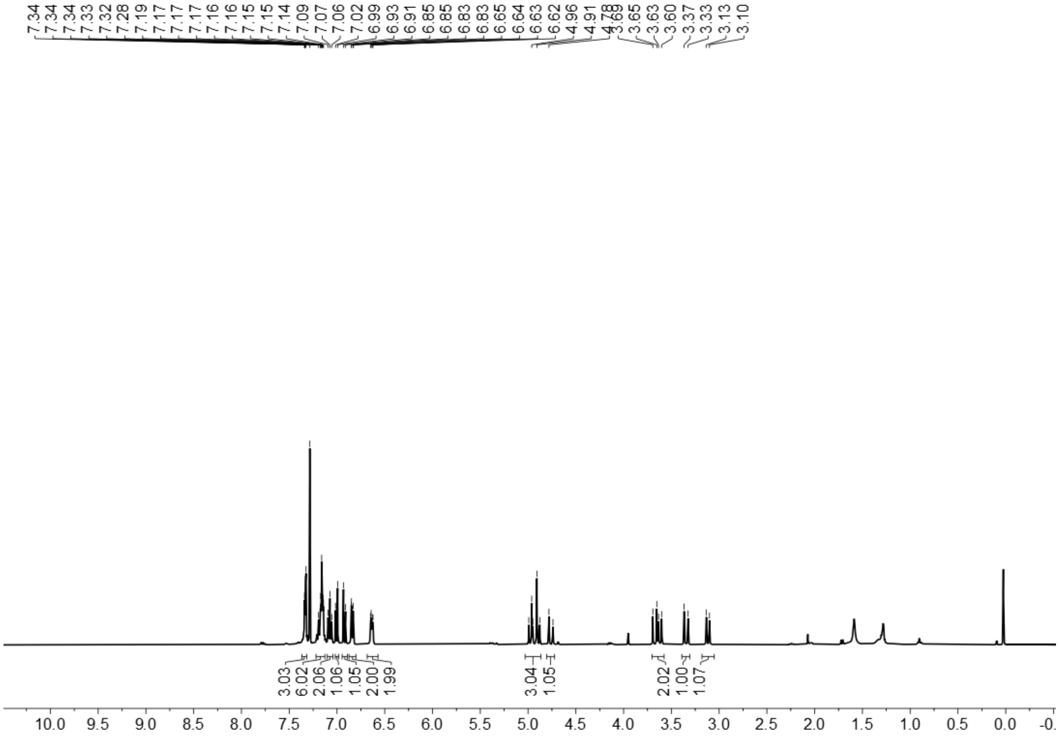

^{1}H NMR (400 MHz, $CDCl_3$)

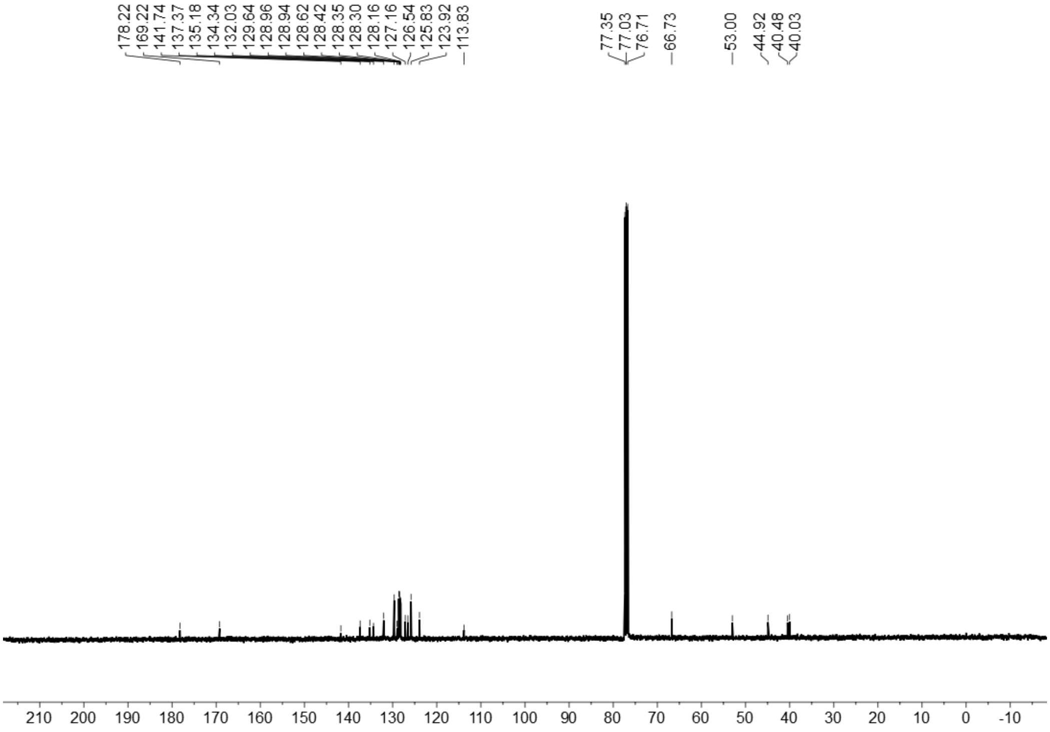

^{13}C NMR (101 MHz, $CDCl_3$)

(S)-1,3-dibenzyl-4,7-dichloro-3-(2-oxo-2-(1H-pyrazol-1-yl)ethyl)indolin-2-one (**4s**)

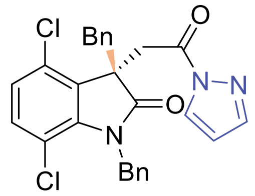

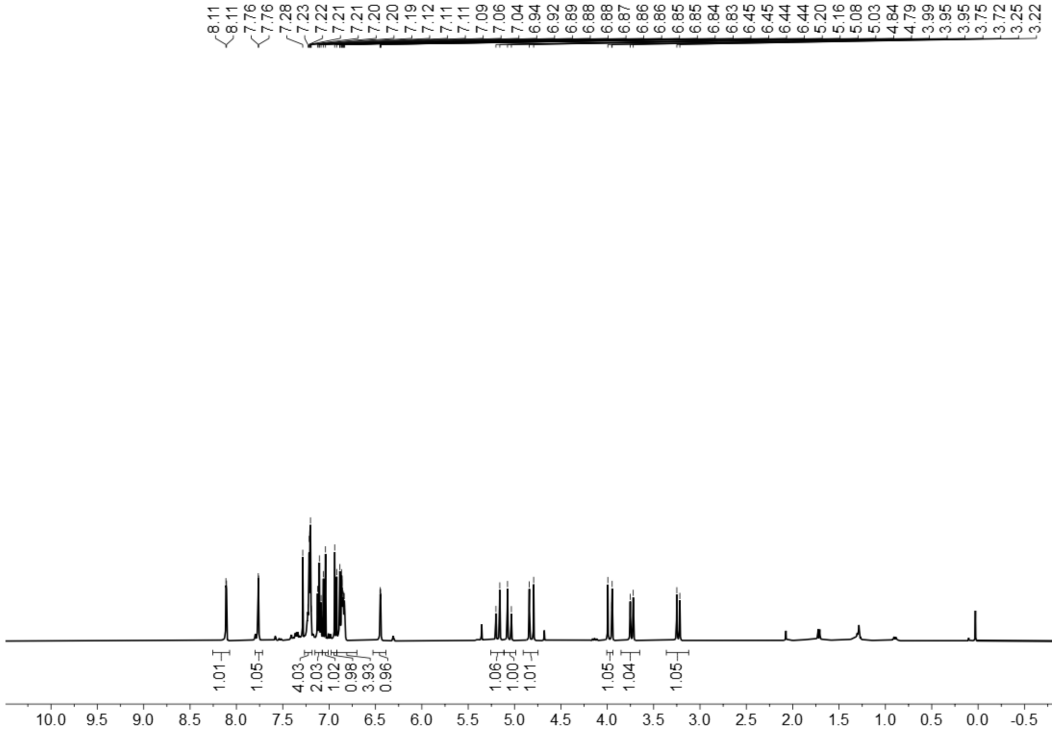

^{1}H NMR (400 MHz, $CDCl_3$)

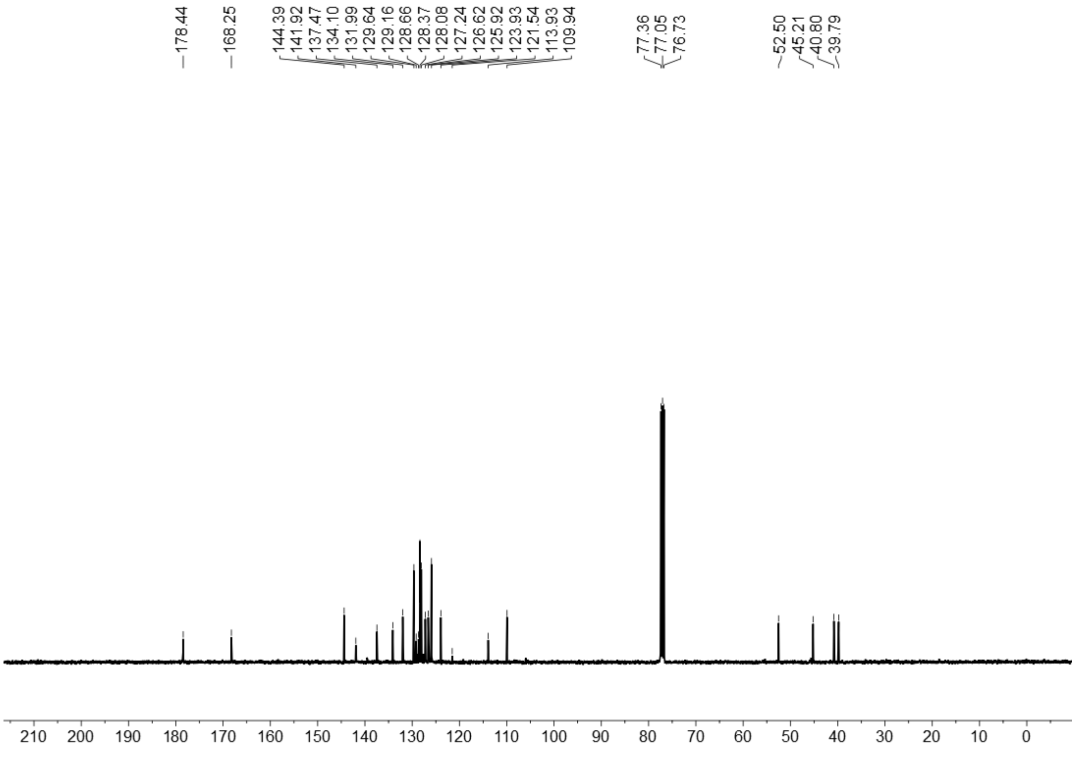

^{13}C NMR (101 MHz, $CDCl_3$)

2-(1-(4-chlorobenzoyl)-5-methoxy-2-methyl-1H-indol-3-yl)ethyl (S)-2-(1,3-dibenzyl-4,7-dichloro-2-oxoindolin-3-yl)acetate (**4t**)

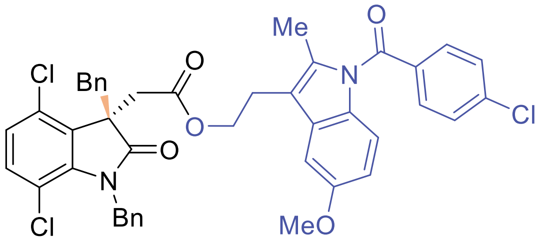

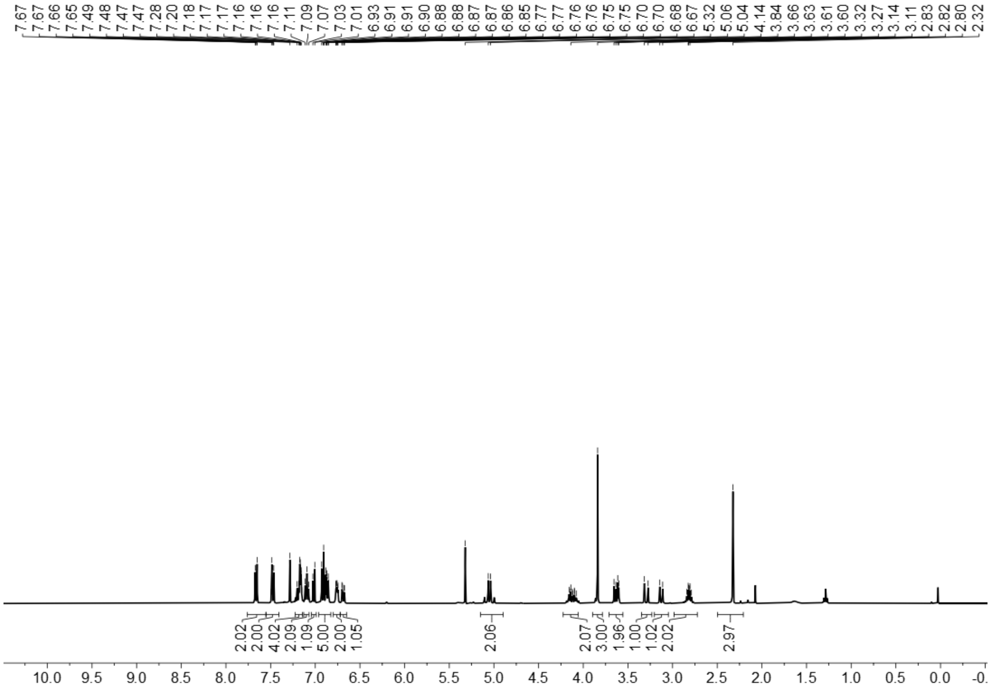

^{1}H NMR (400 MHz, $CDCl_3$)

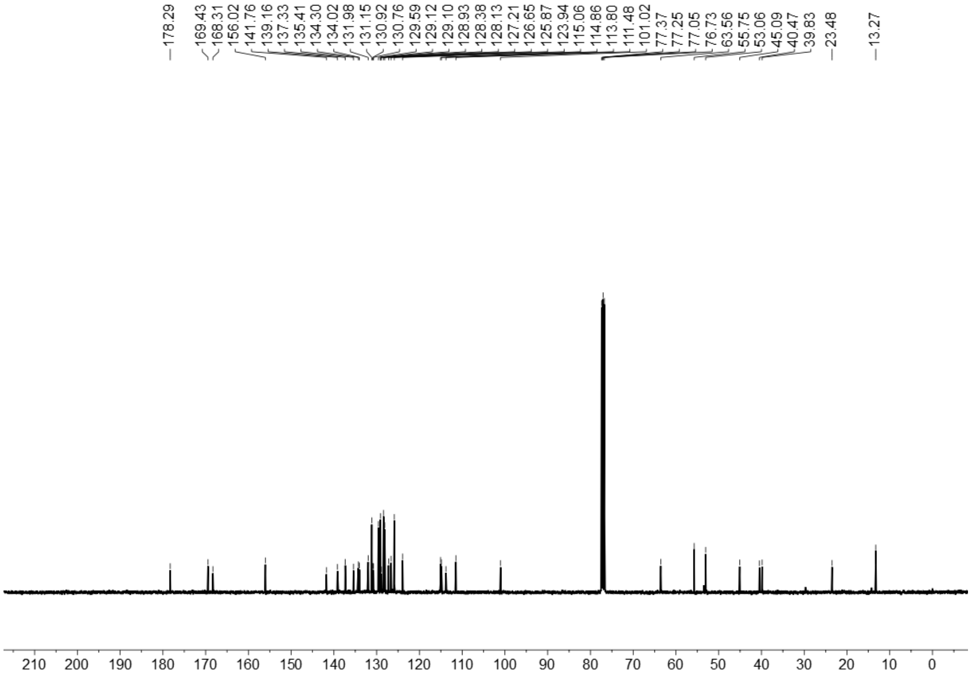

^{13}C NMR (101 MHz, $CDCl_3$)

S-benzyl (S)-2-(1,3-dibenzyl-4,7-dichloro-2-oxoindolin-3-yl)ethanethioate (**4u**)

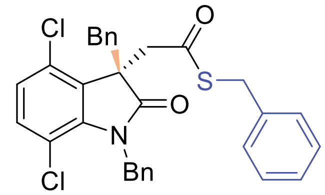

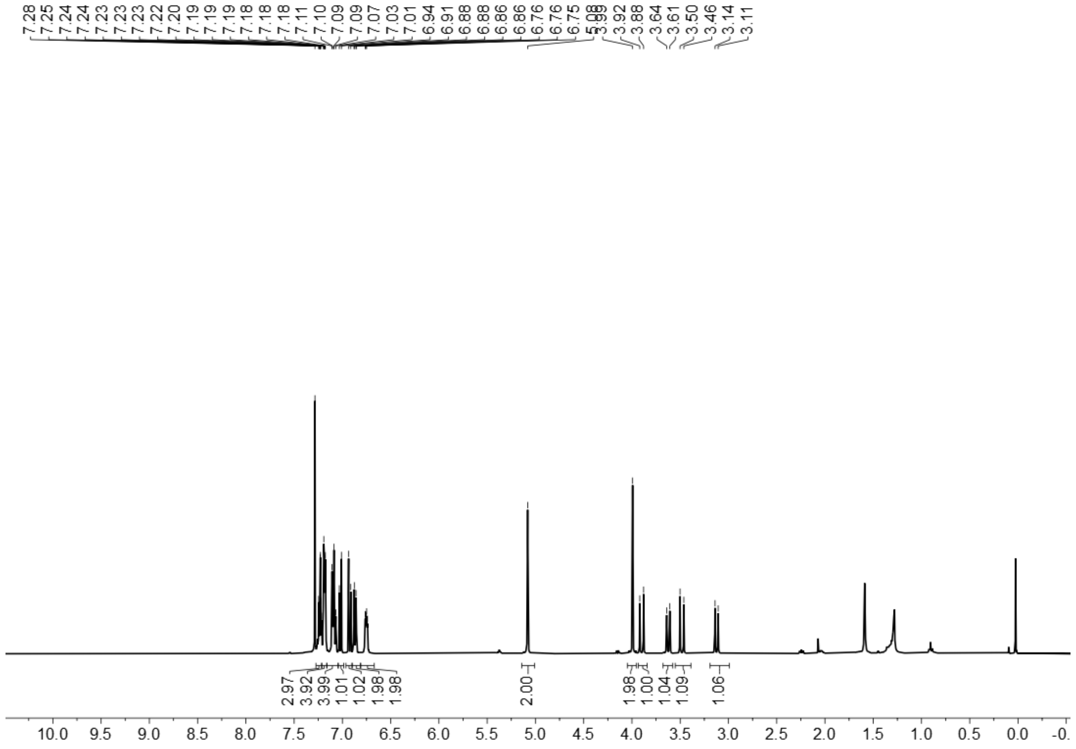

¹H NMR (400 MHz, $CDCl_3$)

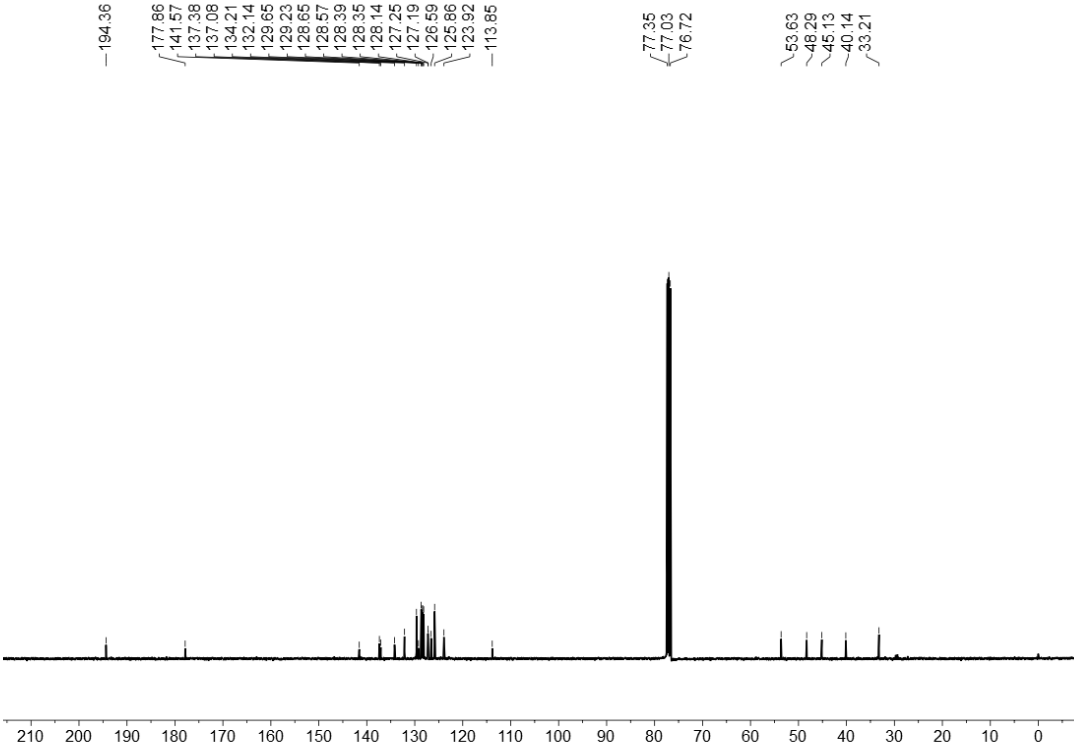

¹³C NMR (101 MHz, $CDCl_3$)

methyl (S)-2-(1-benzyl-4,7-dichloro-3-(4-fluorobenzyl)-2-oxoindolin-3-yl)acetate (**5a**)

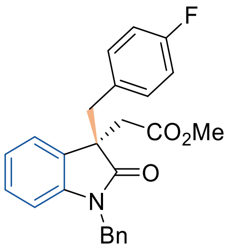

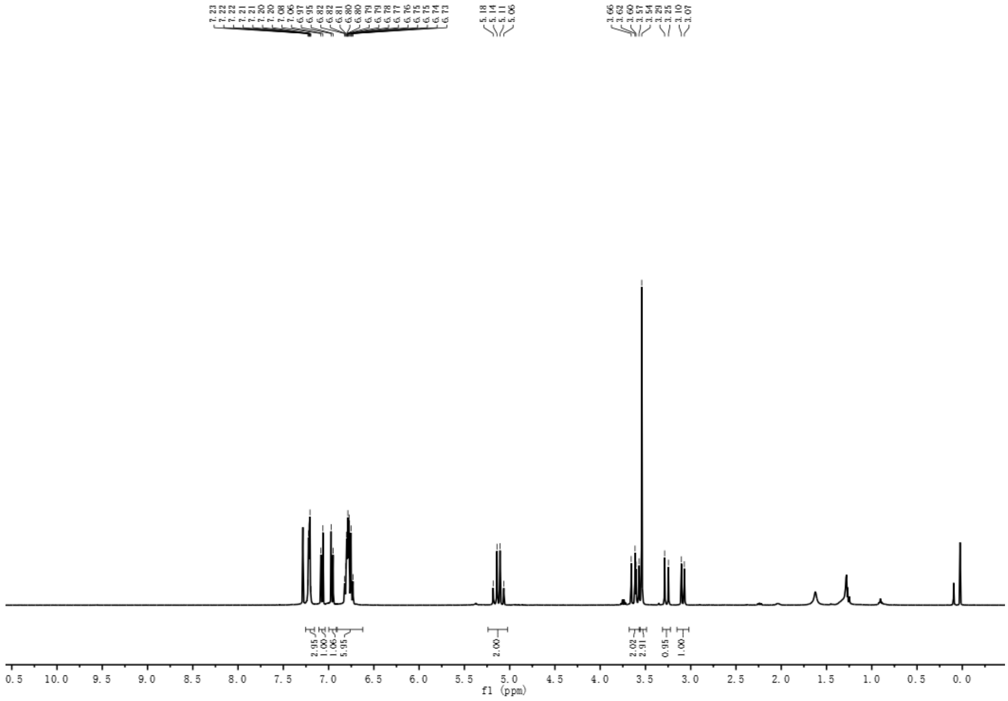

^{1}H NMR (400 MHz, $CDCl_3$)

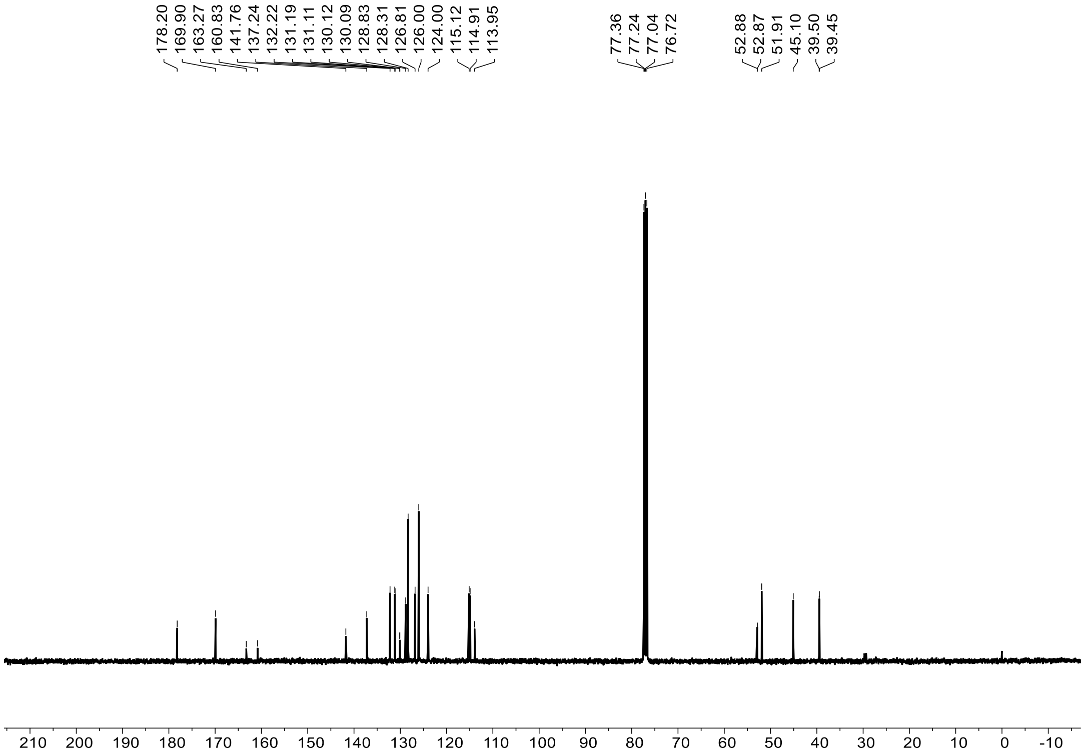

^{13}C NMR (101 MHz, $CDCl_3$)

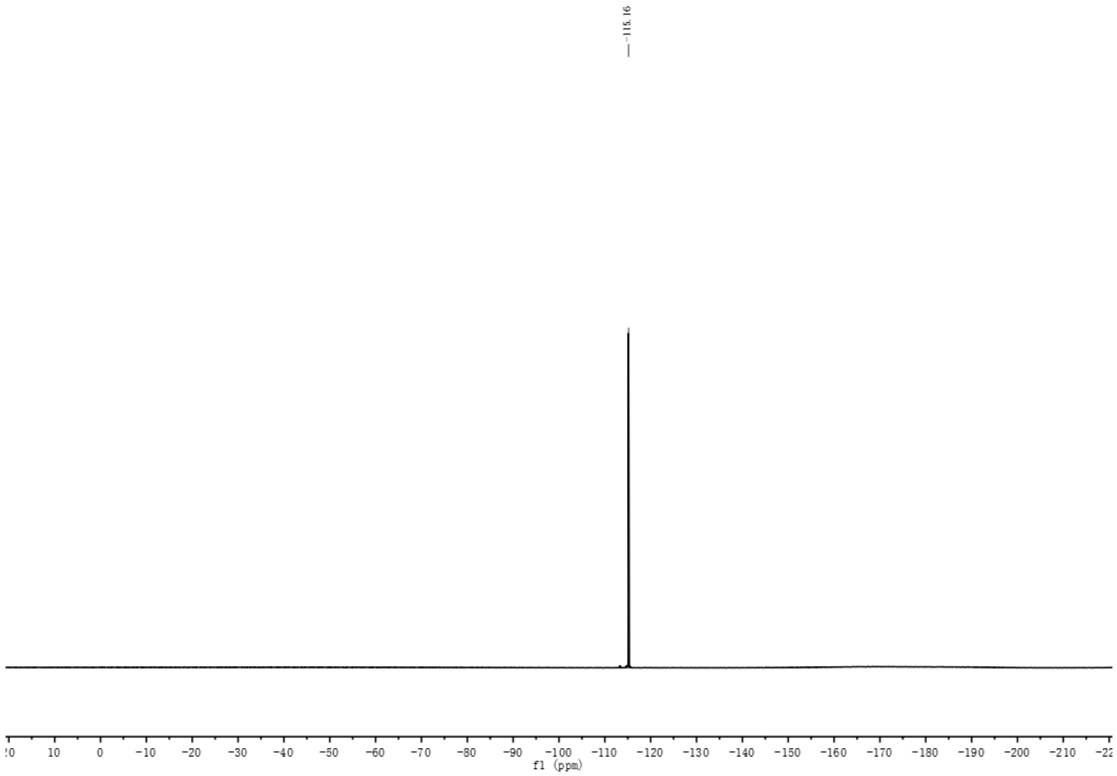

^{19}F NMR (376 MHz, $CDCl_3$)

methyl (S)-2-(1-benzyl-3-(4-chlorobenzyl)-4,6-difluoro-2-oxoindolin-3-yl)acetate (**5b**)

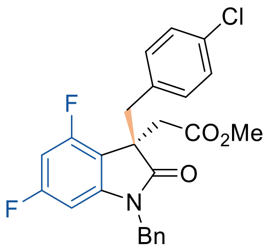

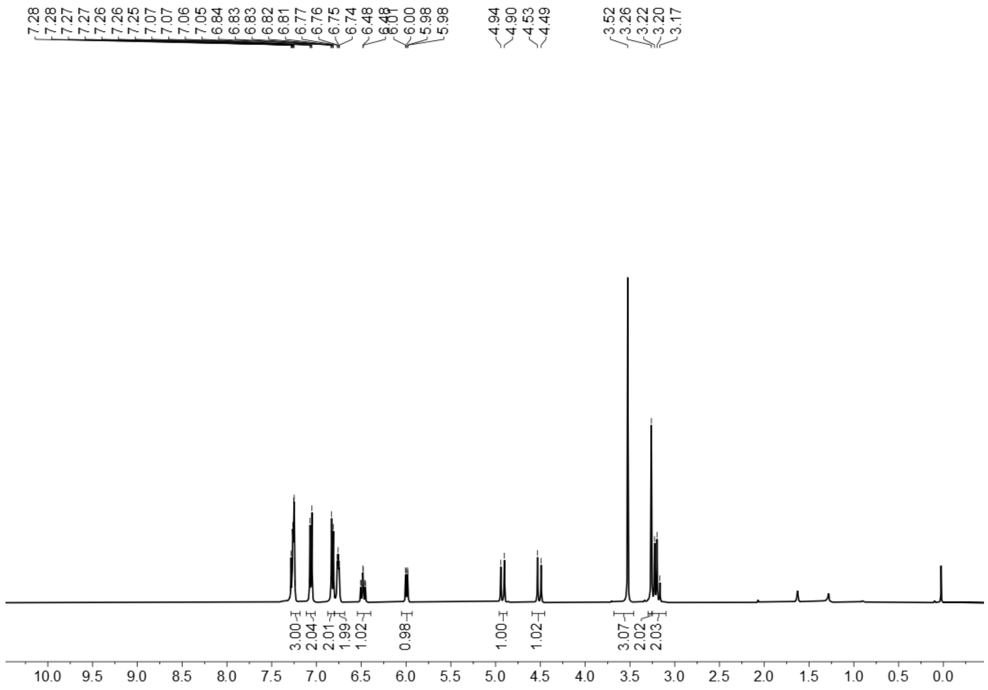

^{1}H NMR (400 MHz, $CDCl_3$)

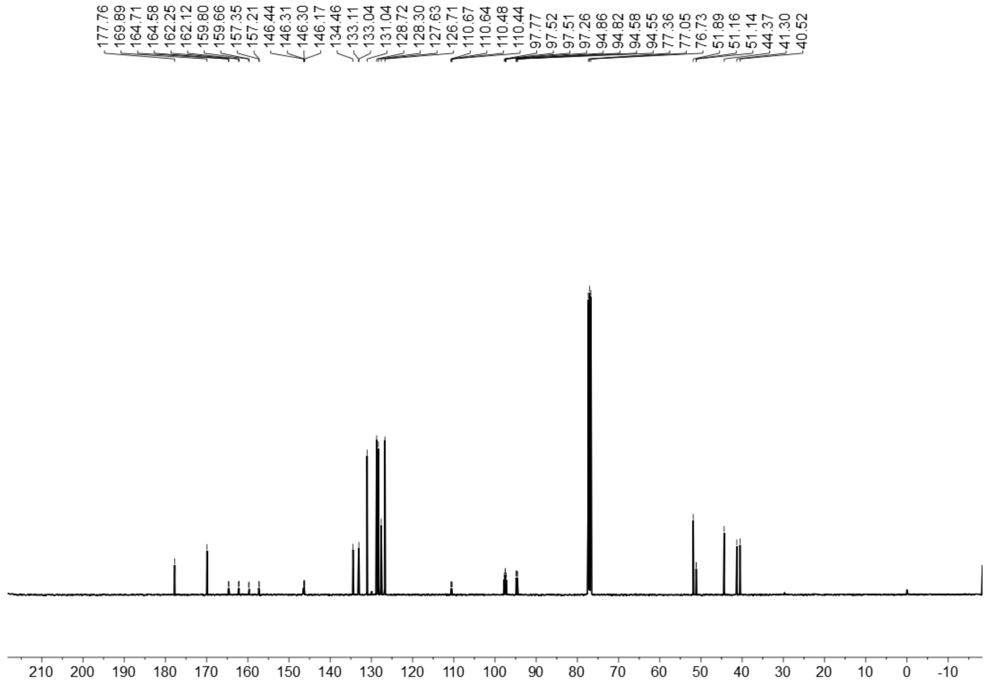

^{13}C NMR (101 MHz, $CDCl_3$)

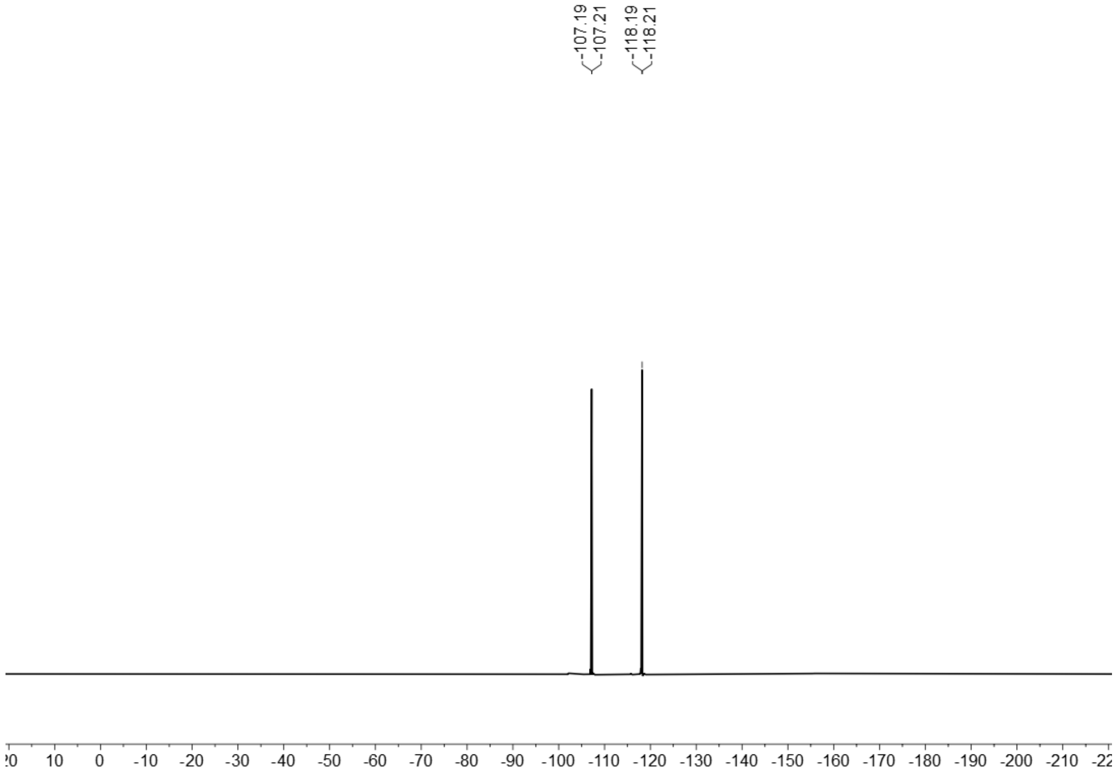

^{19}F NMR (376 MHz, $CDCl_3$)

methyl (S)-2-(1-benzyl-3-(4-bromobenzyl)-4,7-dichloro-2-oxoindolin-3-yl)acetate (**5c**)

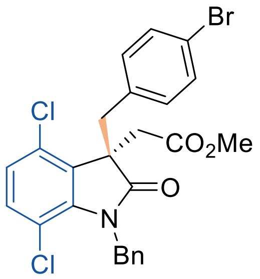

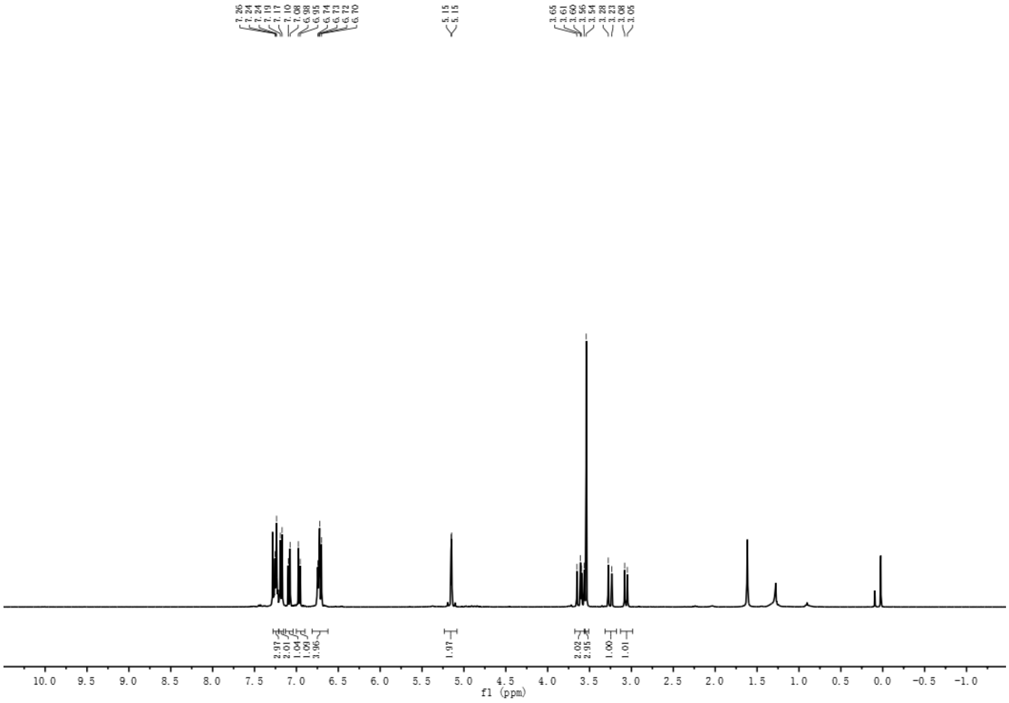

^{1}H NMR (400 MHz, $CDCl_3$)

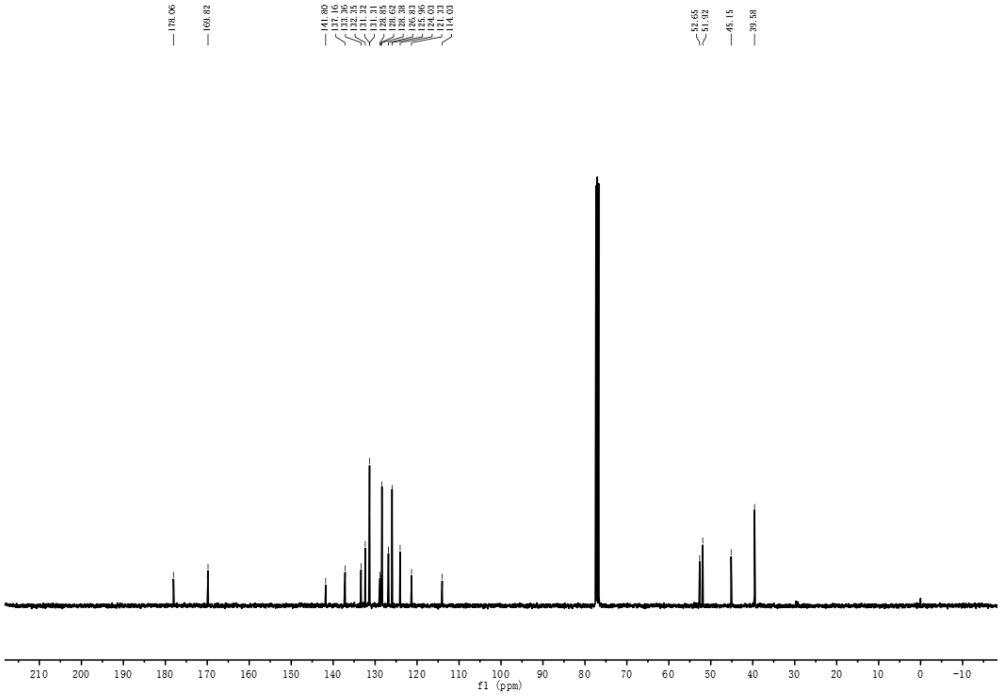

^{13}C NMR (101 MHz, $CDCl_3$)

methyl (S)-2-(1-benzyl-4,7-dichloro-3-(4-iodobenzyl)-2-oxoindolin-3-yl)acetate (**5d**)

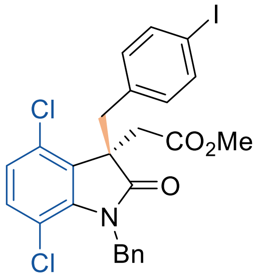

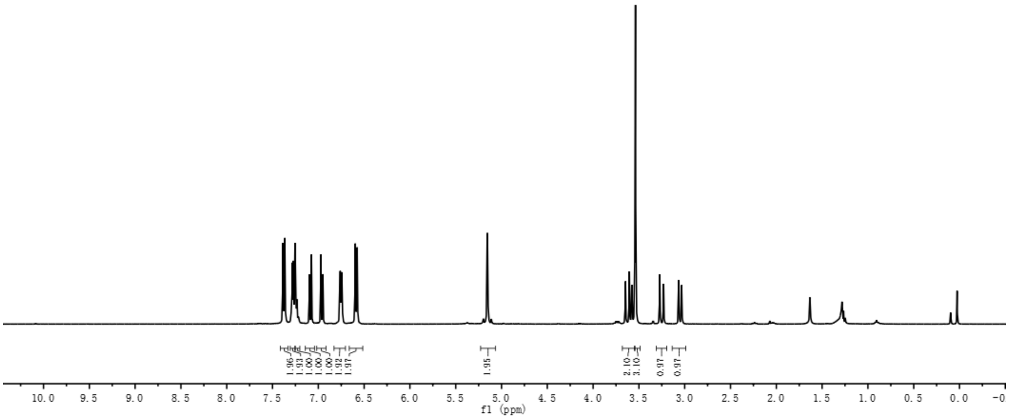

^{1}H NMR (400 MHz, $CDCl_3$)

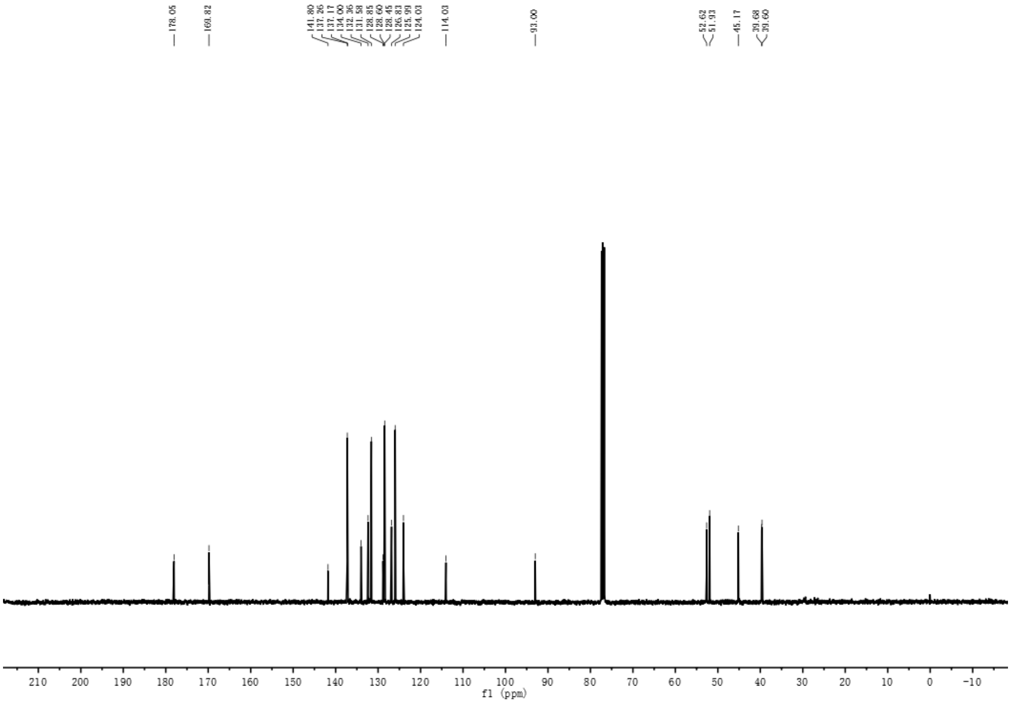

^{13}C NMR (101 MHz, $CDCl_3$)

methyl (S)-2-(1-benzyl-3-(4-cyanobenzyl)-4,6-difluoro-2-oxoindolin-3-yl)acetate (**5e**)

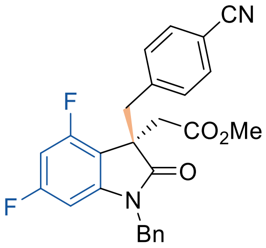

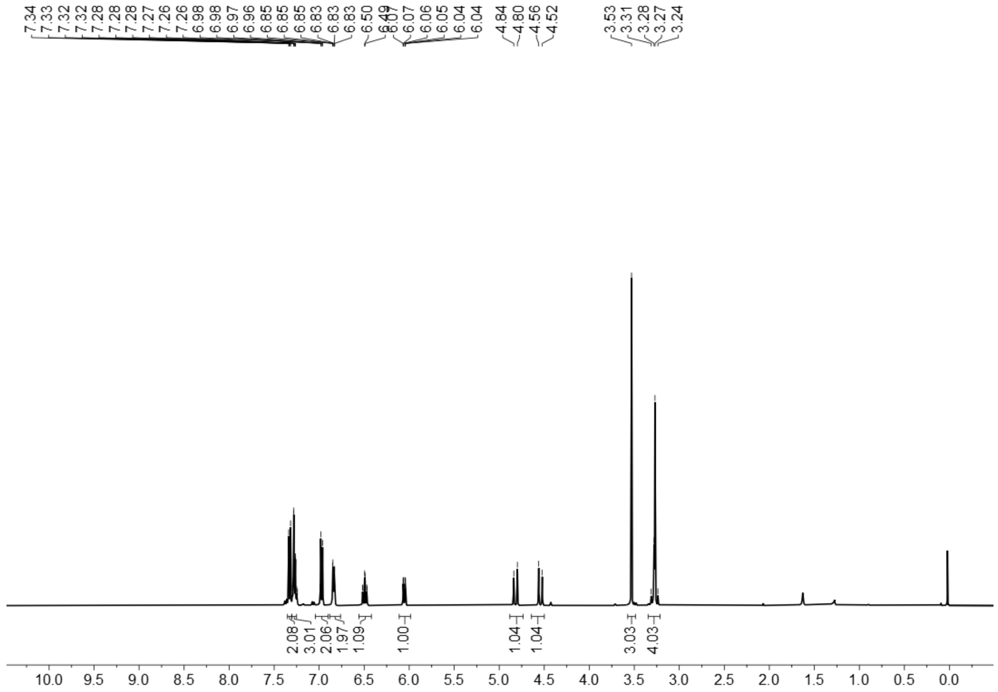

^{1}H NMR (400 MHz, $CDCl_3$)

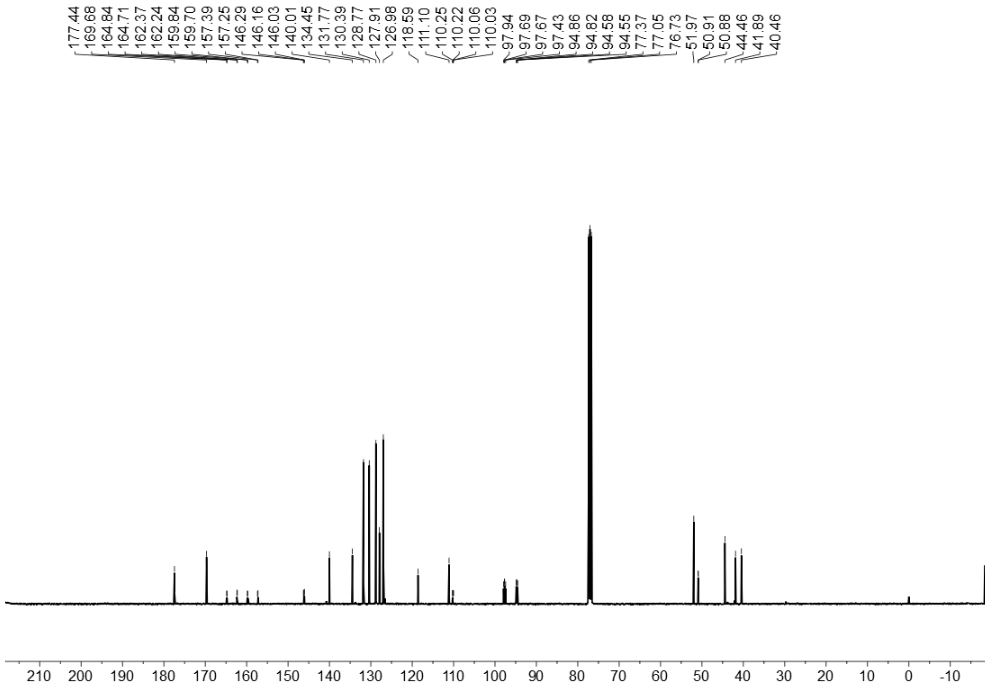

^{13}**C NMR** (101 MHz, $CDCl_3$)

methyl (S)-2-(1-benzyl-4,7-dichloro-3-(4-nitrobenzyl)-2-oxoindolin-3-yl)acetate (**5f**)

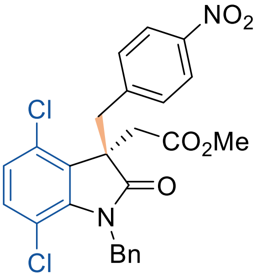

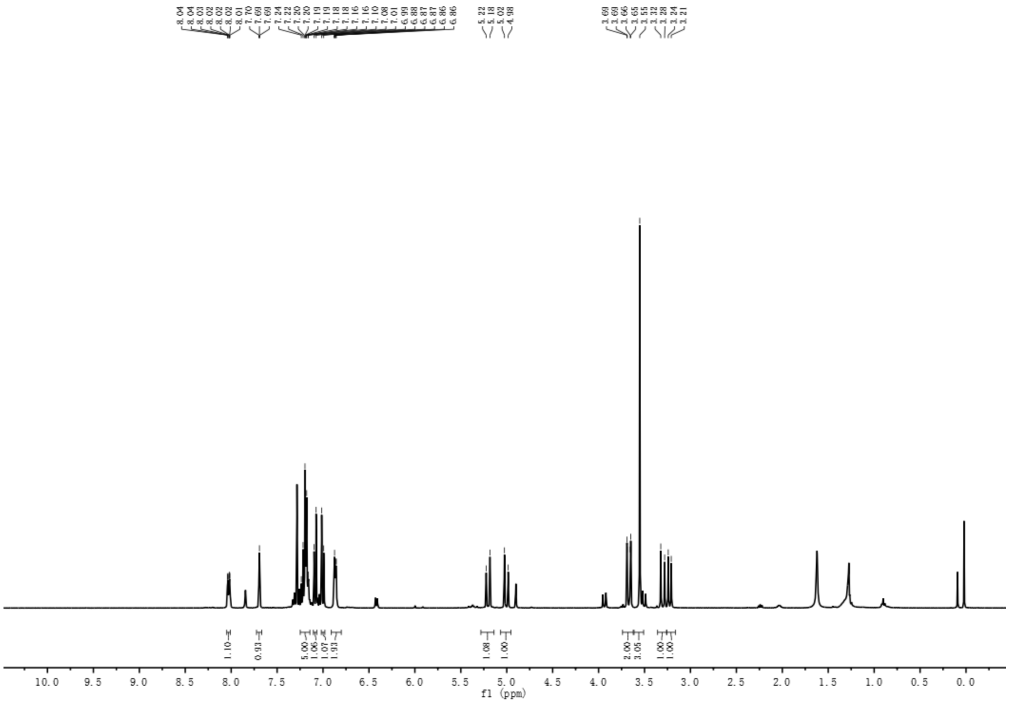

^{1}**H NMR** (400 MHz, $CDCl_3$)

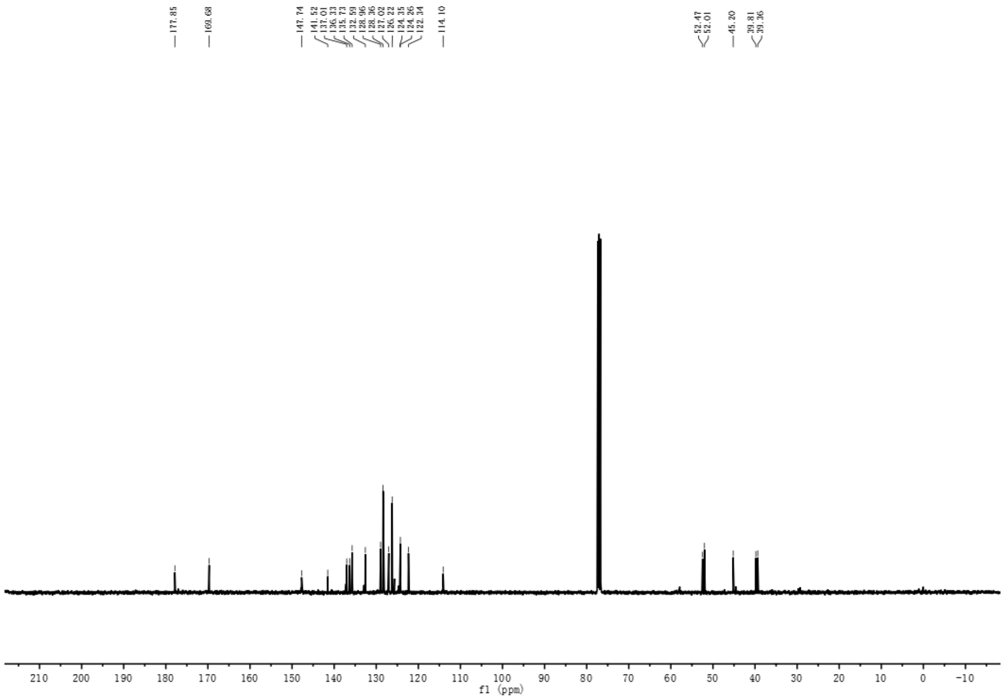

^{13}C NMR (101 MHz, $CDCl_3$)

methyl (S)-4-((1-benzyl-4,7-dichloro-3-(2-methoxy-2-oxoethyl)-2-oxoindolin-3-yl)methyl)benzoate (**5g**)

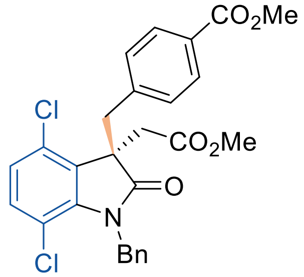

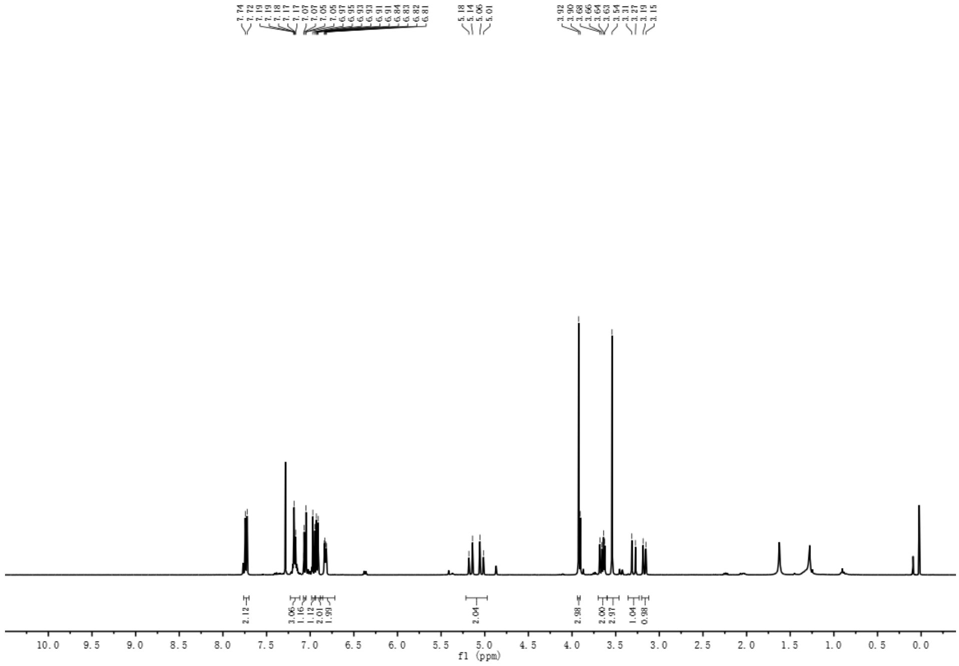

^{1}**H NMR** (400 MHz, $CDCl_3$)

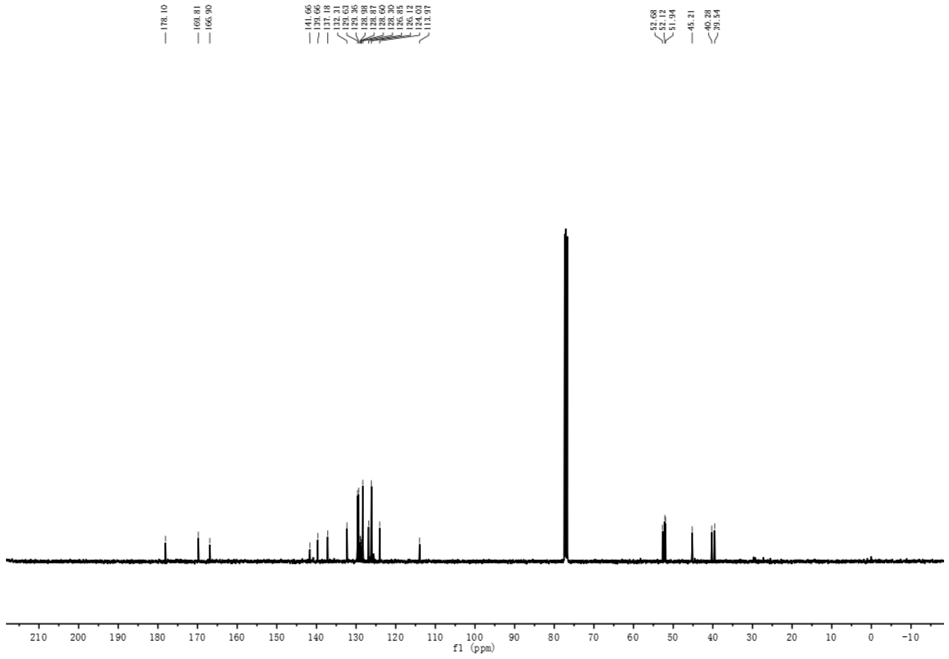

^{13}**C NMR** (101 MHz, $CDCl_3$)

methyl (S)-2-(3-(4-acetylbenzyl)-1-benzyl-4,7-dichloro-2-oxoindolin-3-yl)acetate (**5h**)

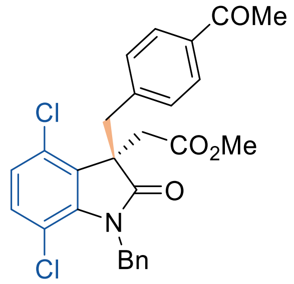

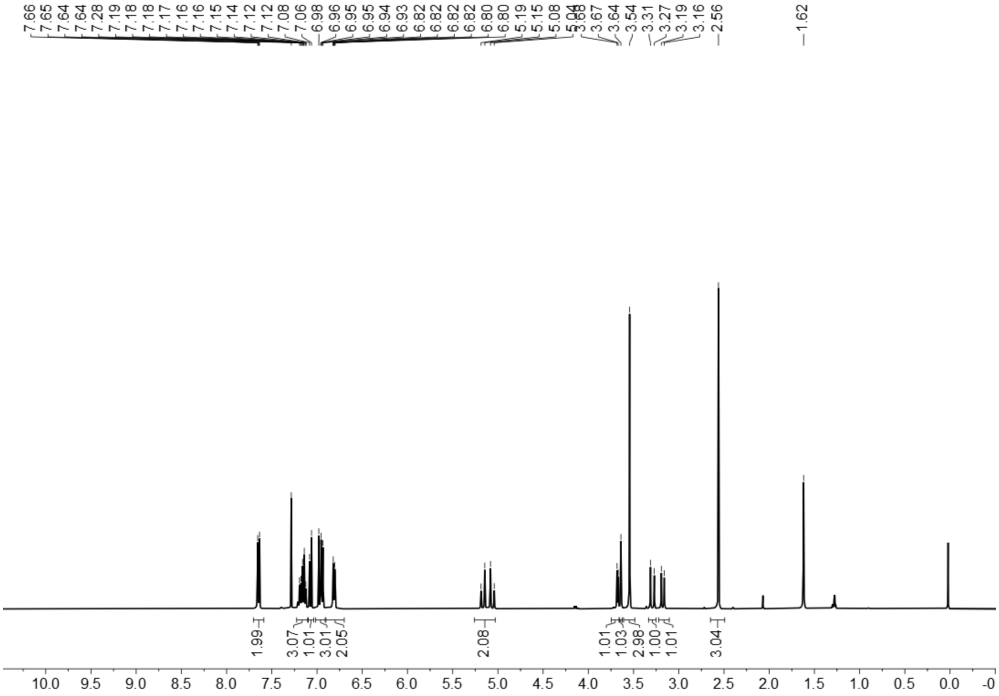

^{1}H NMR (400 MHz, $CDCl_3$)

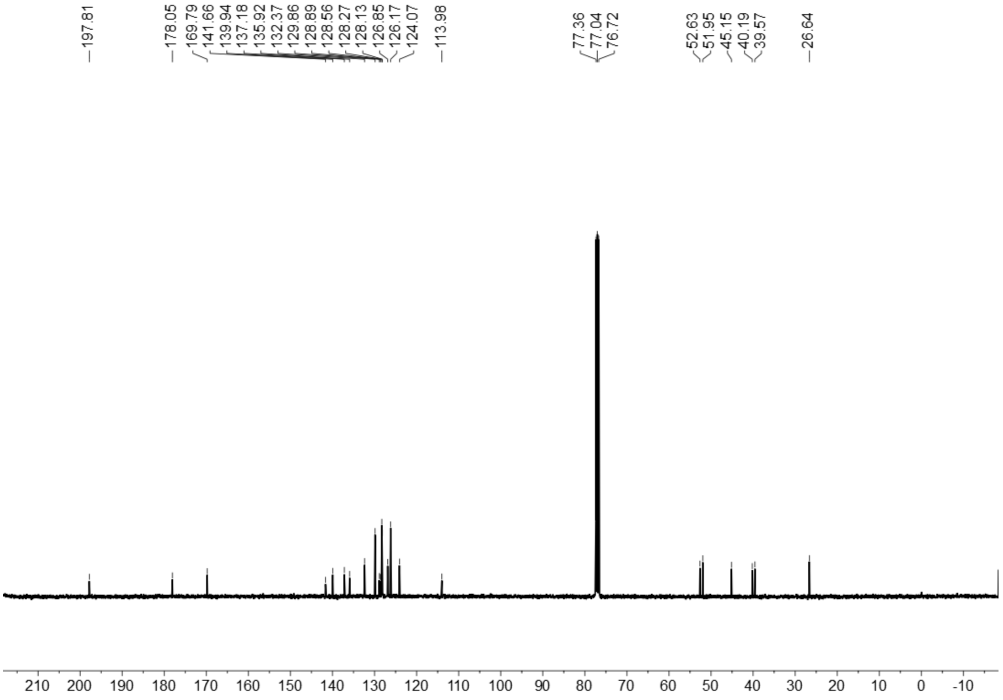

^{13}C NMR (101 MHz, $CDCl_3$)

methyl (S)-2-(1-benzyl-4,7-dichloro-3-(4-(methylsulfonyl)benzyl)-2-oxoindolin-3-yl)acetate (**5i**)

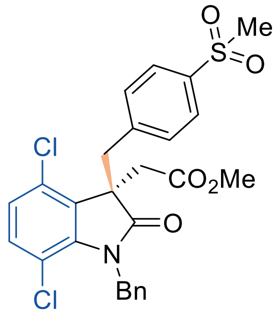

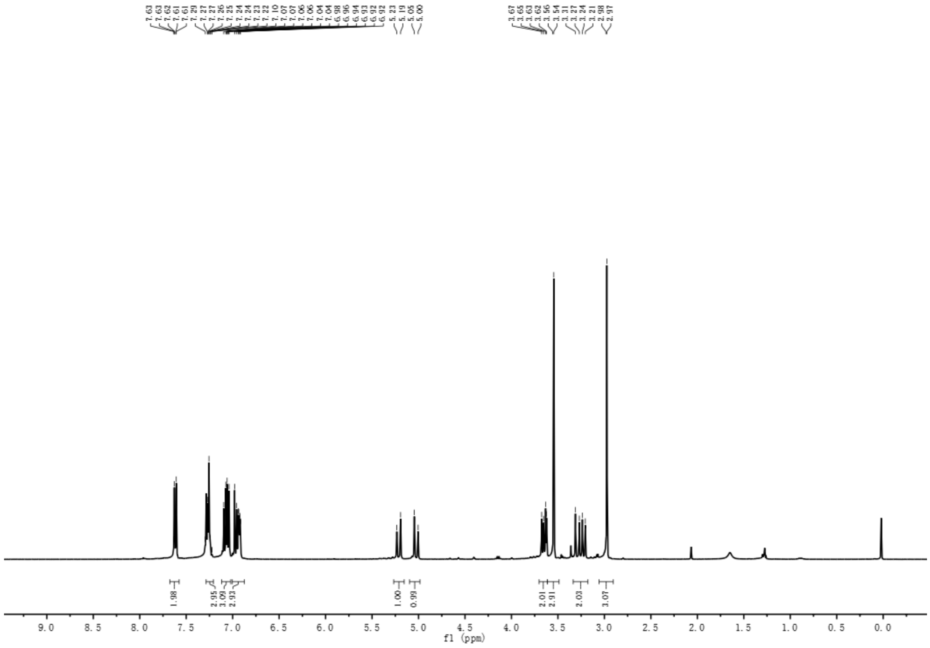

¹H NMR (400 MHz, CDCl₃)

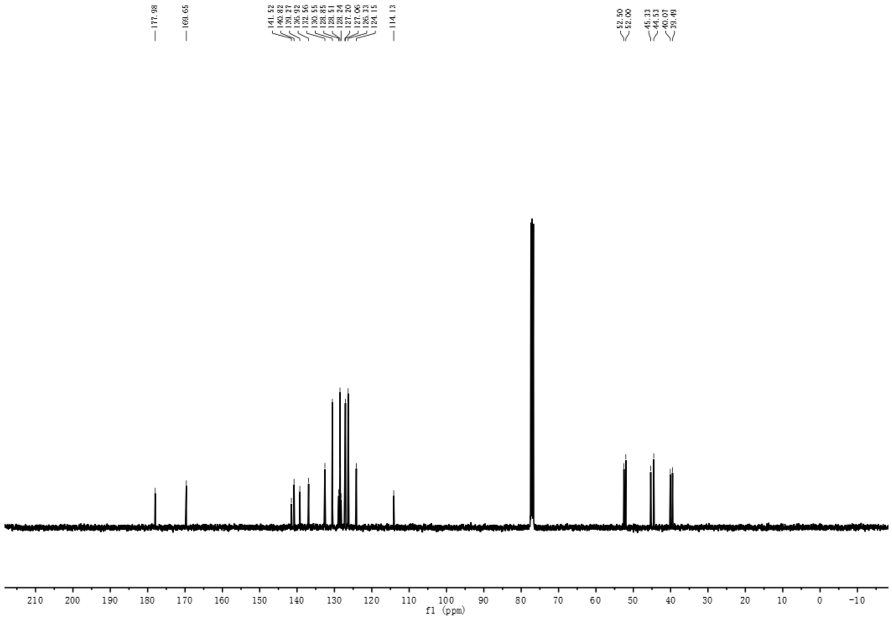

¹³C NMR (101 MHz, CDCl₃)

methyl (S)-2-(3-([1,1'-biphenyl]-4-ylmethyl)-1-benzyl-4,7-dichloro-2-oxoindolin-3-yl)acetate (**5j**)

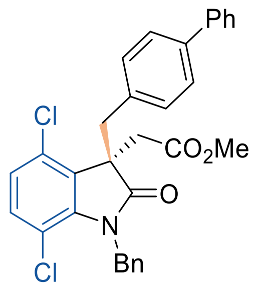

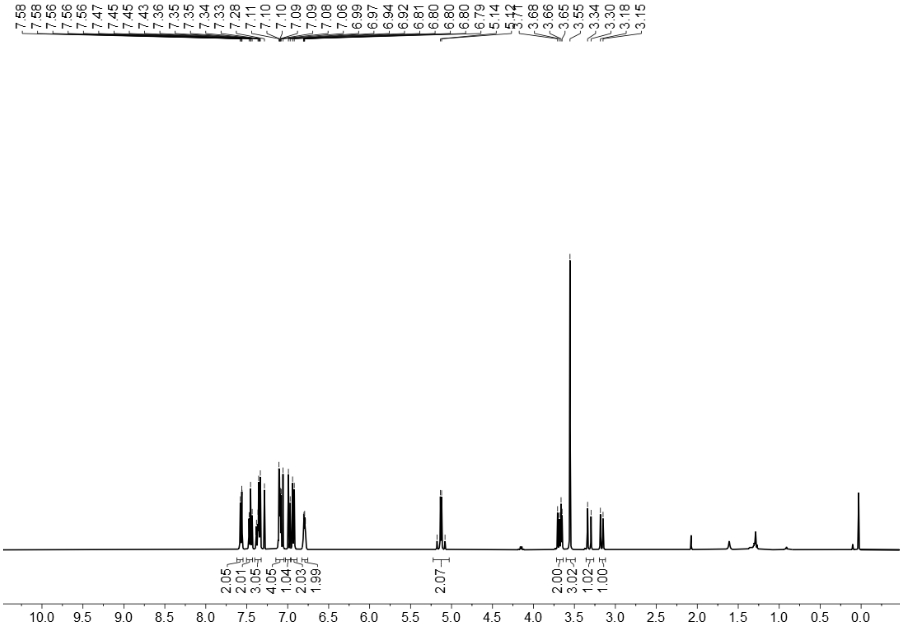

^{1}H NMR (400 MHz, $CDCl_3$)

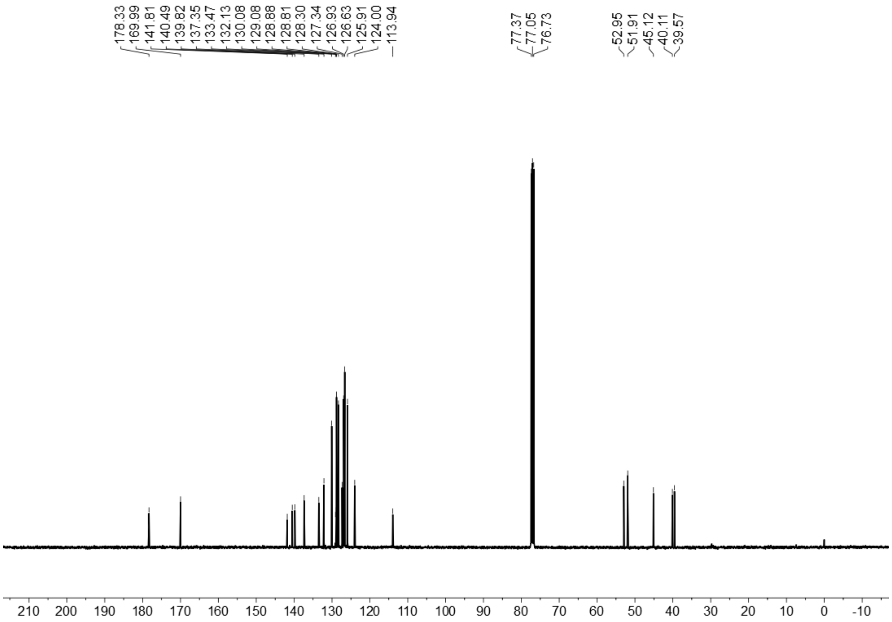

^{13}C NMR (101 MHz, $CDCl_3$)

methyl (S)-2-(1-benzyl-4,7-dichloro-3-(4-methylbenzyl)-2-oxoindolin-3-yl)acetate (**5k**)

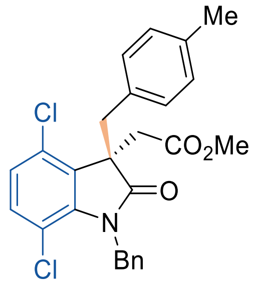

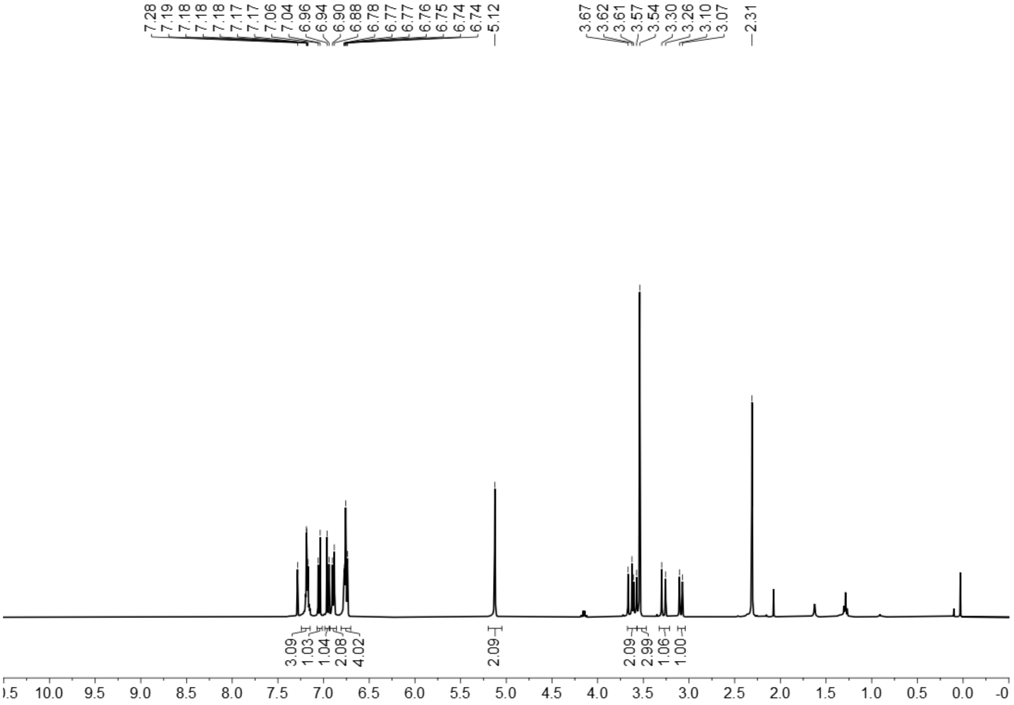

^{1}H NMR (400 MHz, $CDCl_3$)

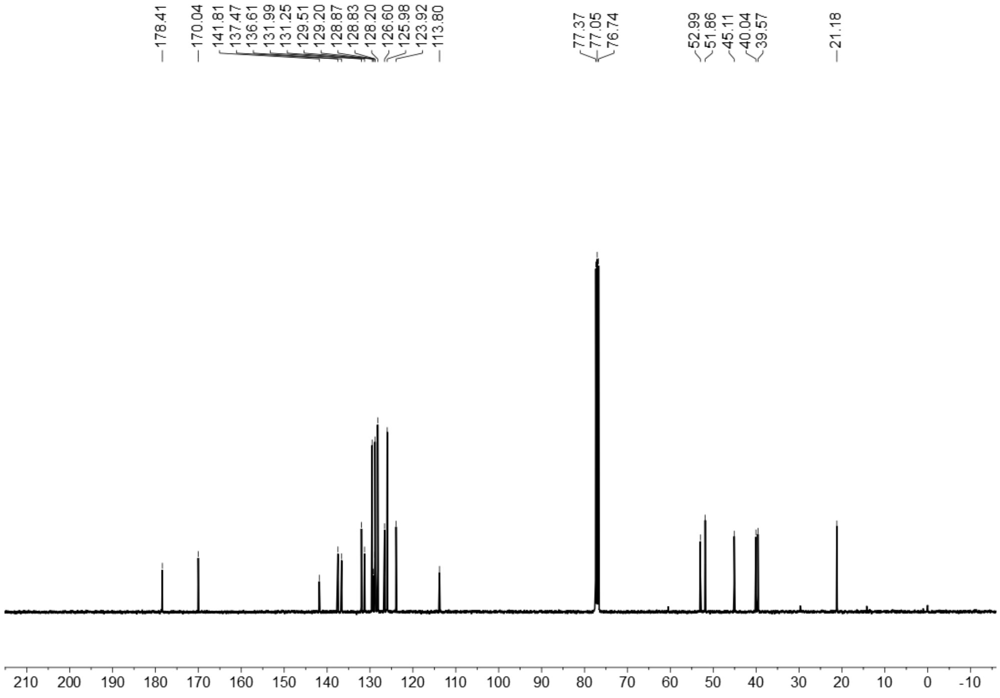

^{13}C NMR (101 MHz, $CDCl_3$)

methyl (S)-2-(1-benzyl-4,6-difluoro-3-(4-methoxybenzyl)-2-oxoindolin-3-yl)acetate (**5l**)

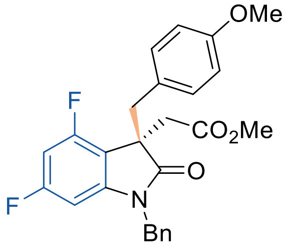

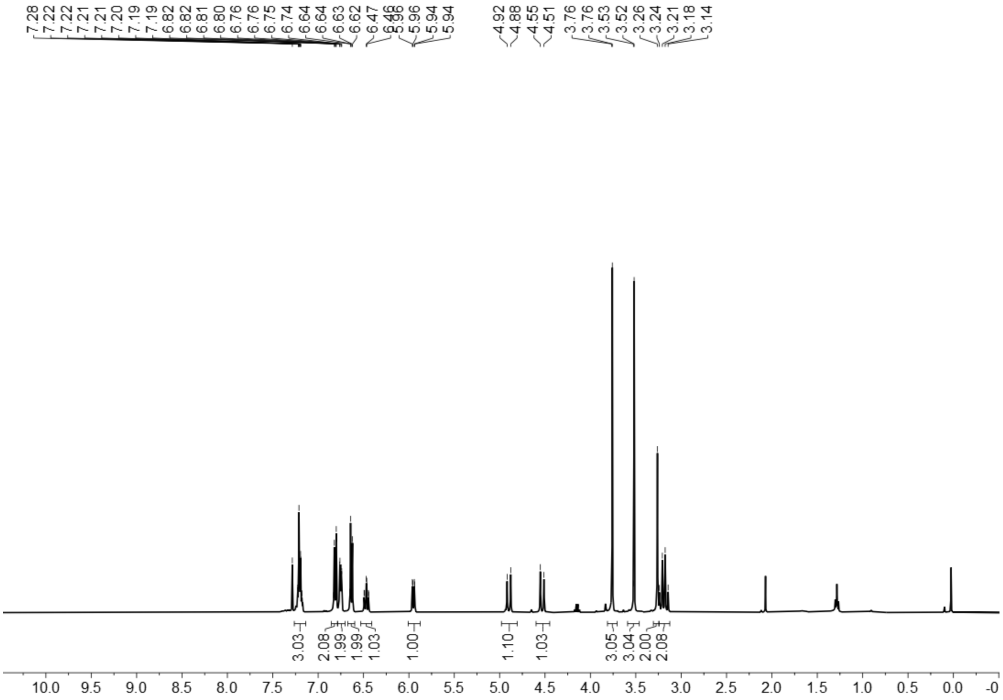

^{1}H NMR (400 MHz, $CDCl_3$)

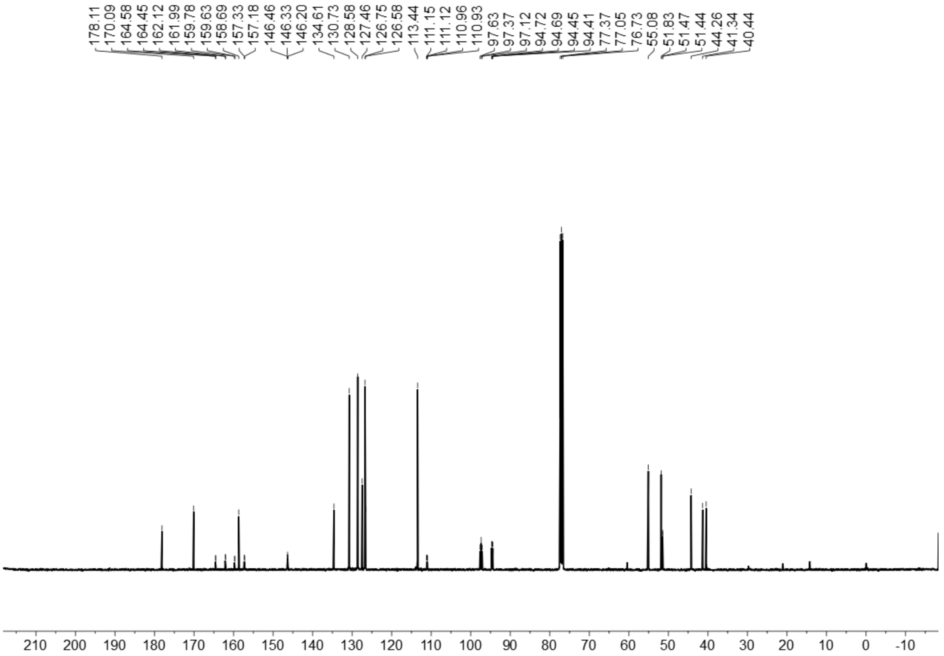

^{13}C NMR (101 MHz, $CDCl_3$)

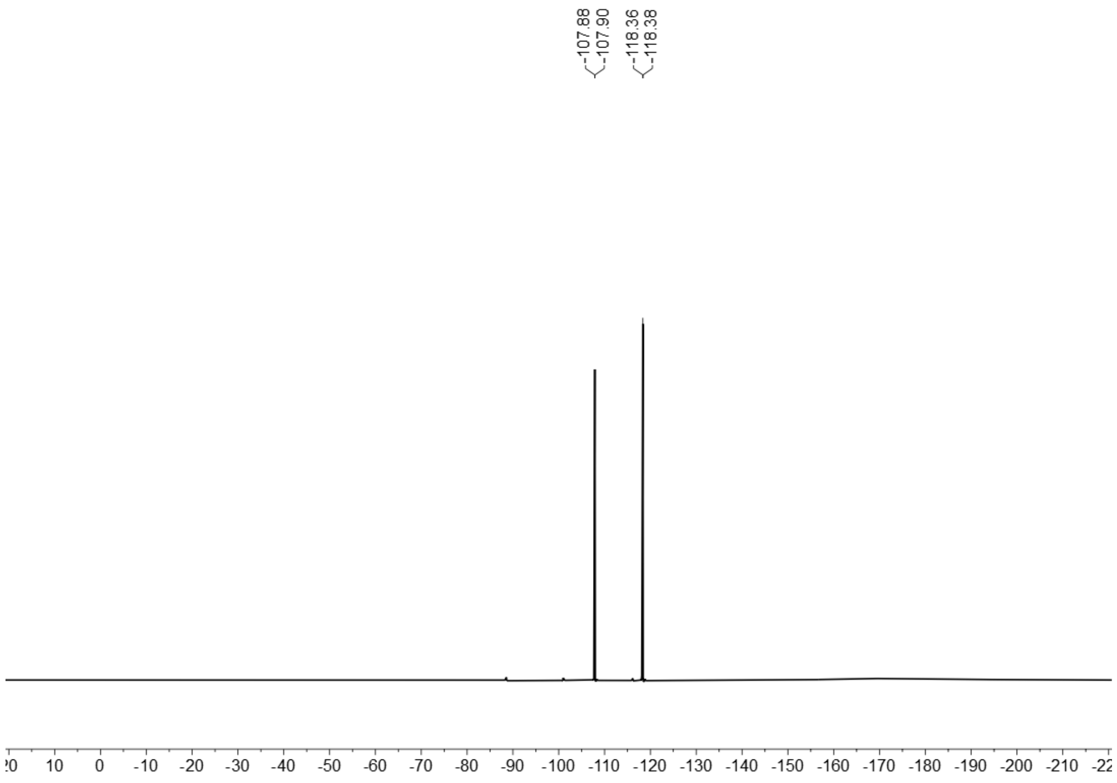

^{19}F NMR (376 MHz, $CDCl_3$)

methyl (S)-2-(1-benzyl-4,7-dichloro-3-(4-(methylthio)benzyl)-2-oxoindolin-3-yl)acetate (**5m**)

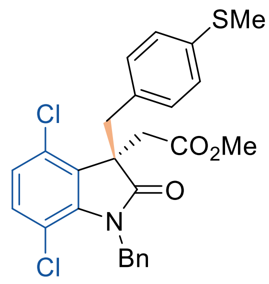

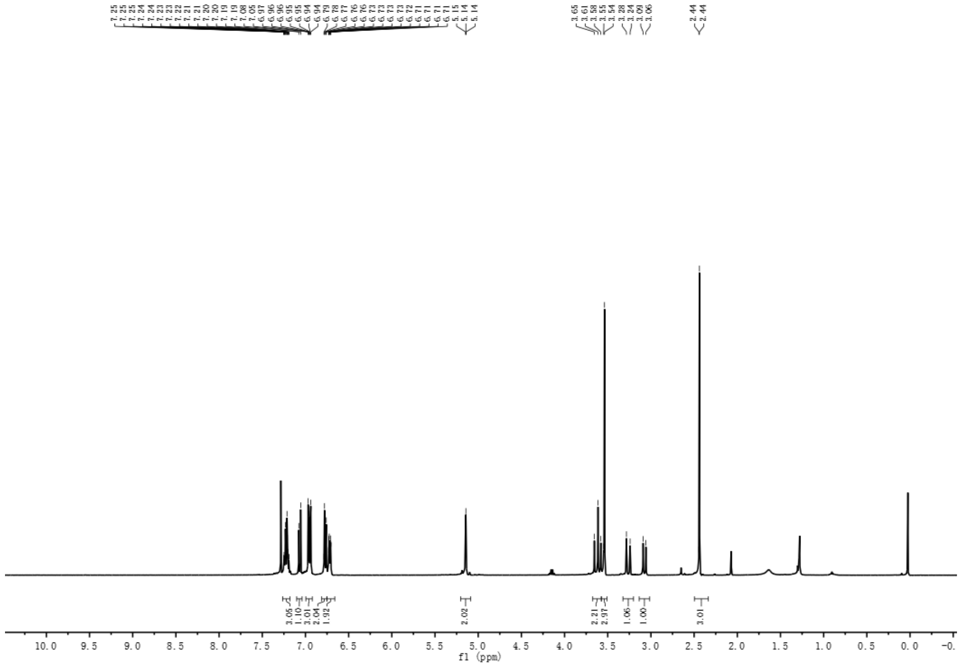

¹H NMR (400 MHz, $CDCl_3$)

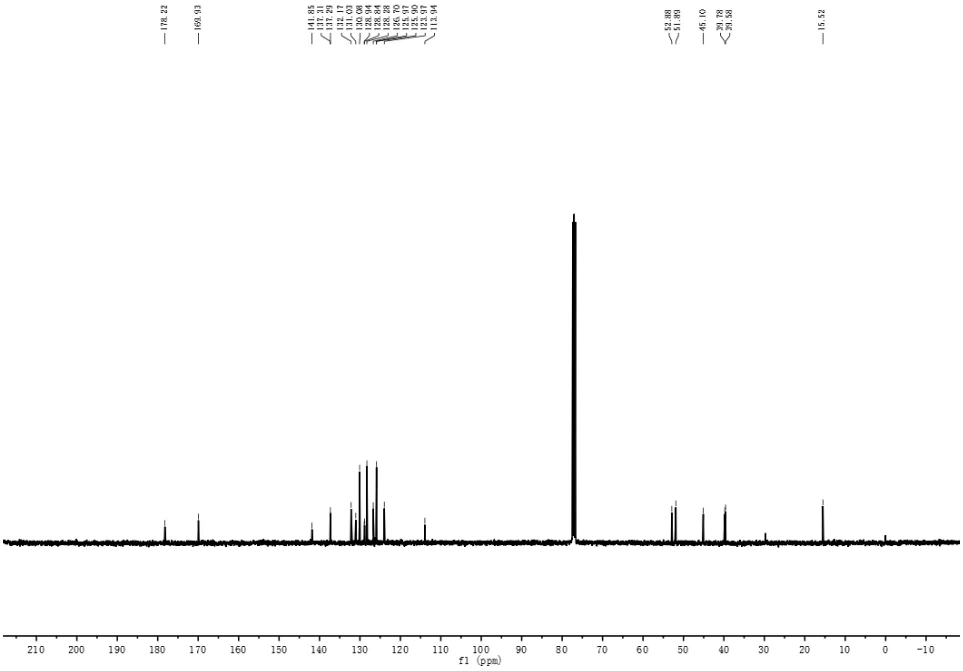

¹³C NMR (101 MHz, $CDCl_3$)

methyl (S)-2-(1-benzyl-4,6-difluoro-2-oxo-3-(3-(trifluoromethyl)benzyl)indolin-3-yl)acetate (**5n**)

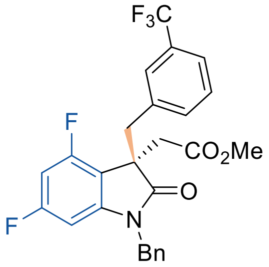

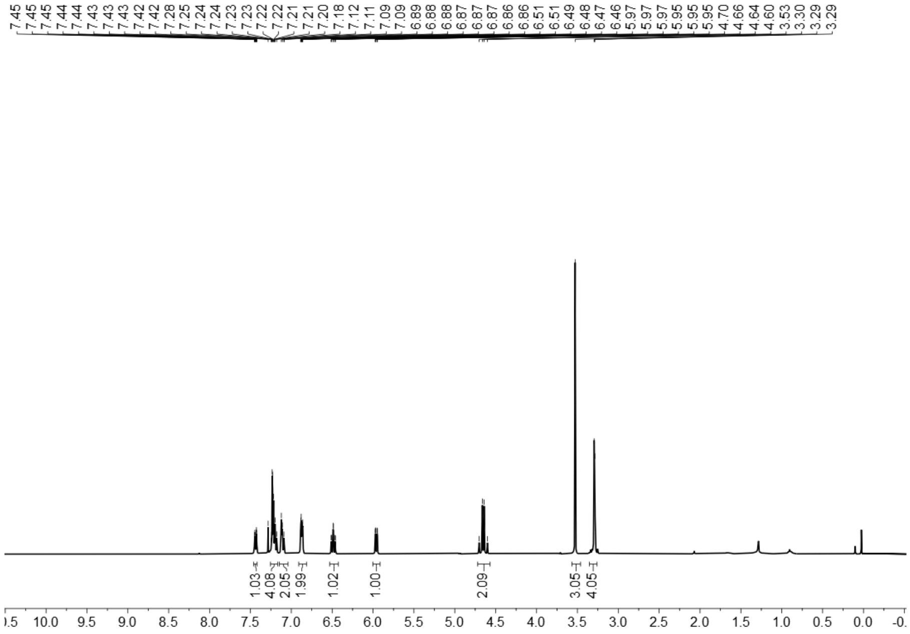

^{1}H NMR (400 MHz, $CDCl_3$)

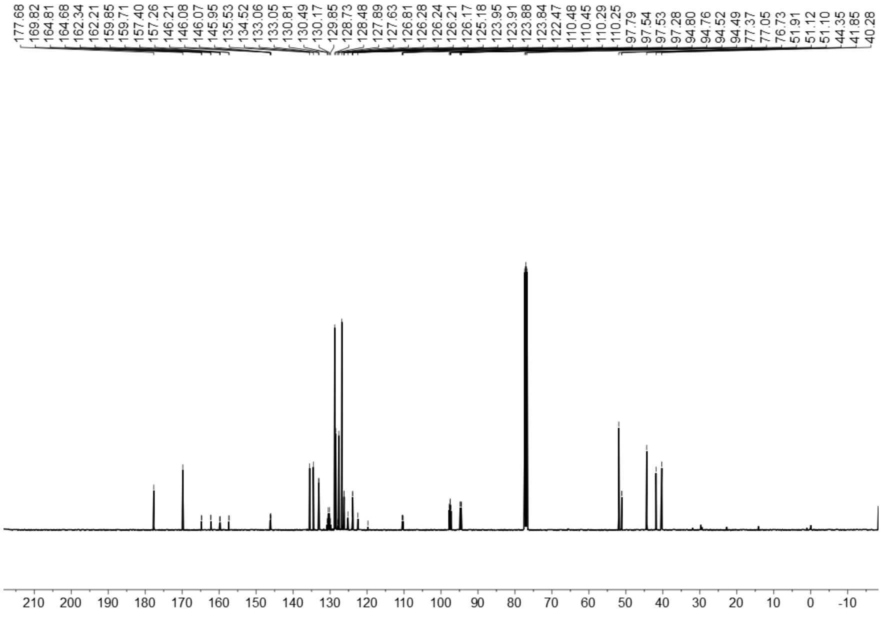

^{13}C NMR (101 MHz, $CDCl_3$)

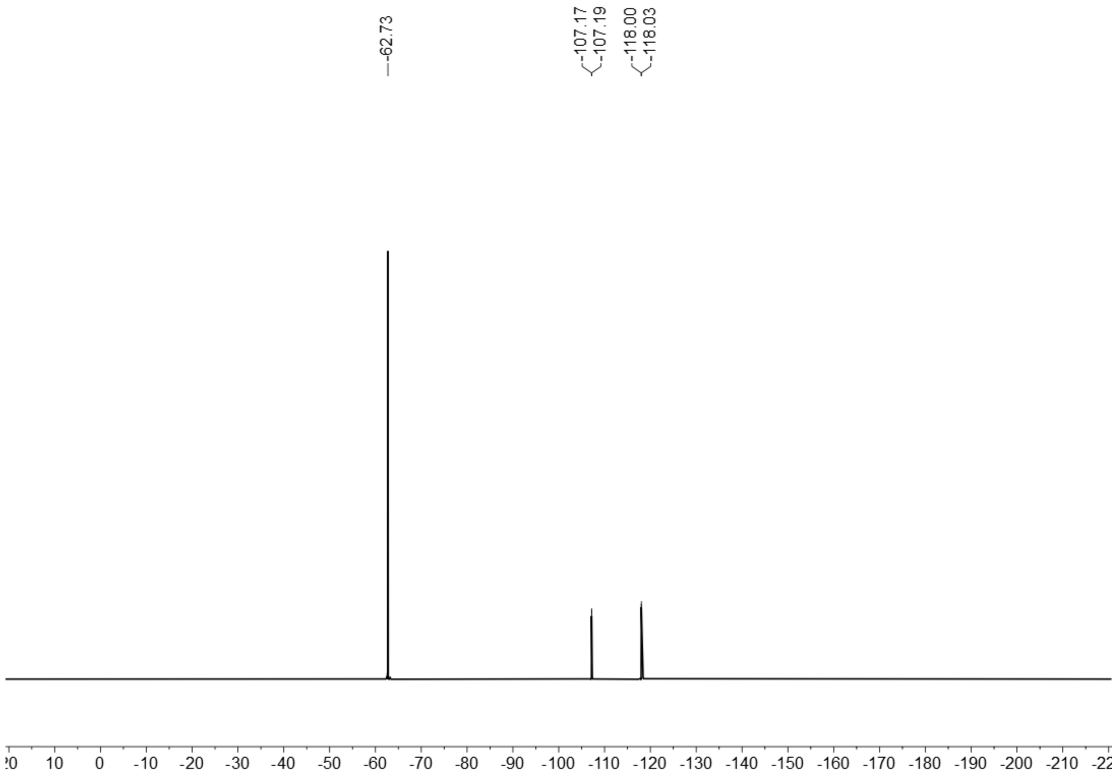

^{19}F NMR (101 MHz, $CDCl_3$)

methyl (S)-2-(1-benzyl-4,7-dichloro-2-oxo-3-((perfluorophenyl)methyl)indolin-3-yl)acetate (**5o**)

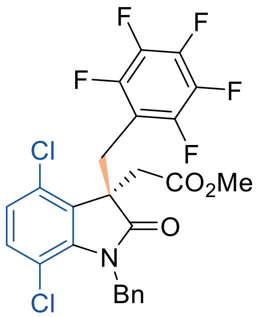

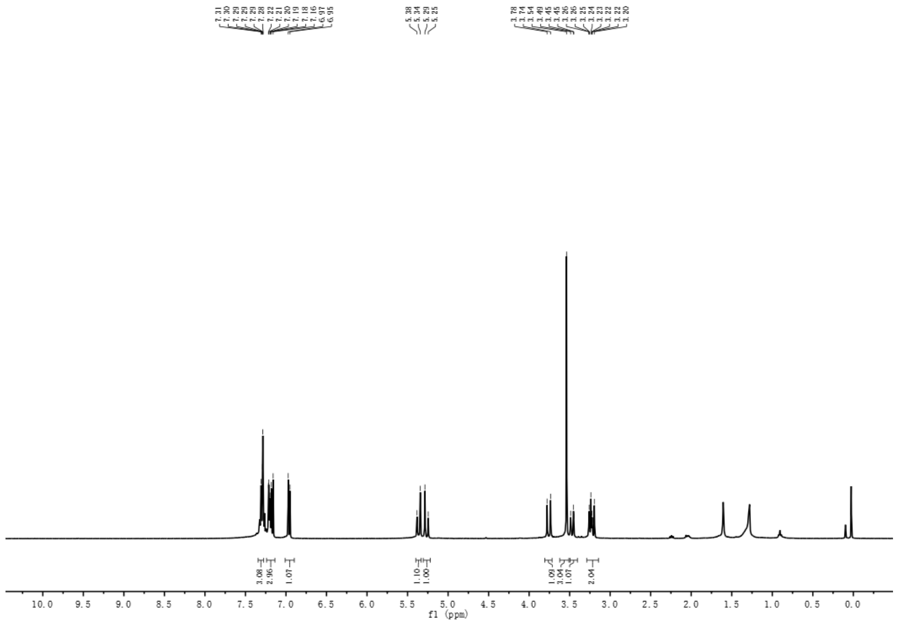

^{1}H NMR (400 MHz, $CDCl_3$)

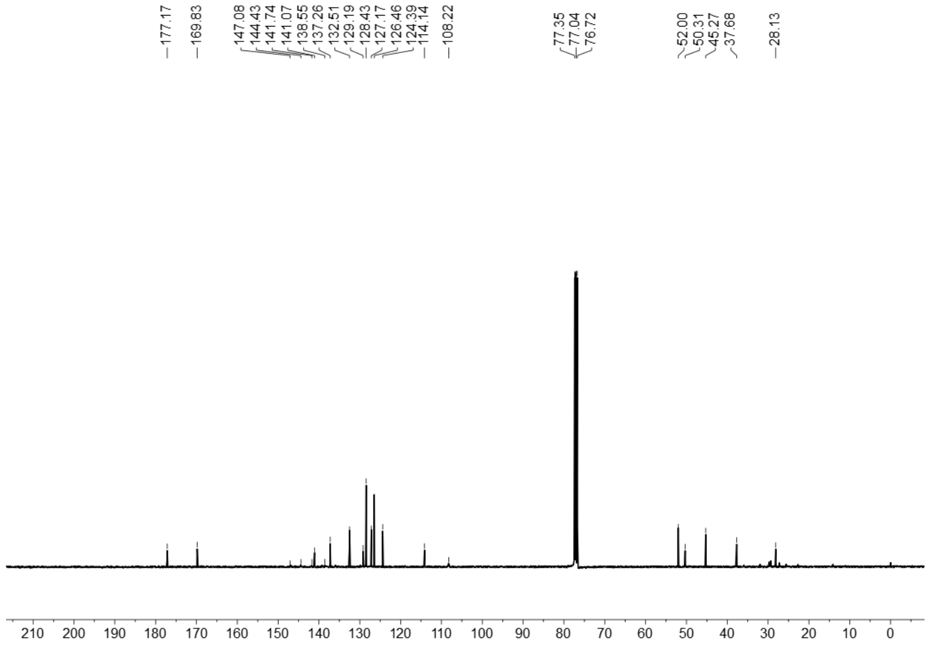

^{13}C NMR (101 MHz, $CDCl_3$)

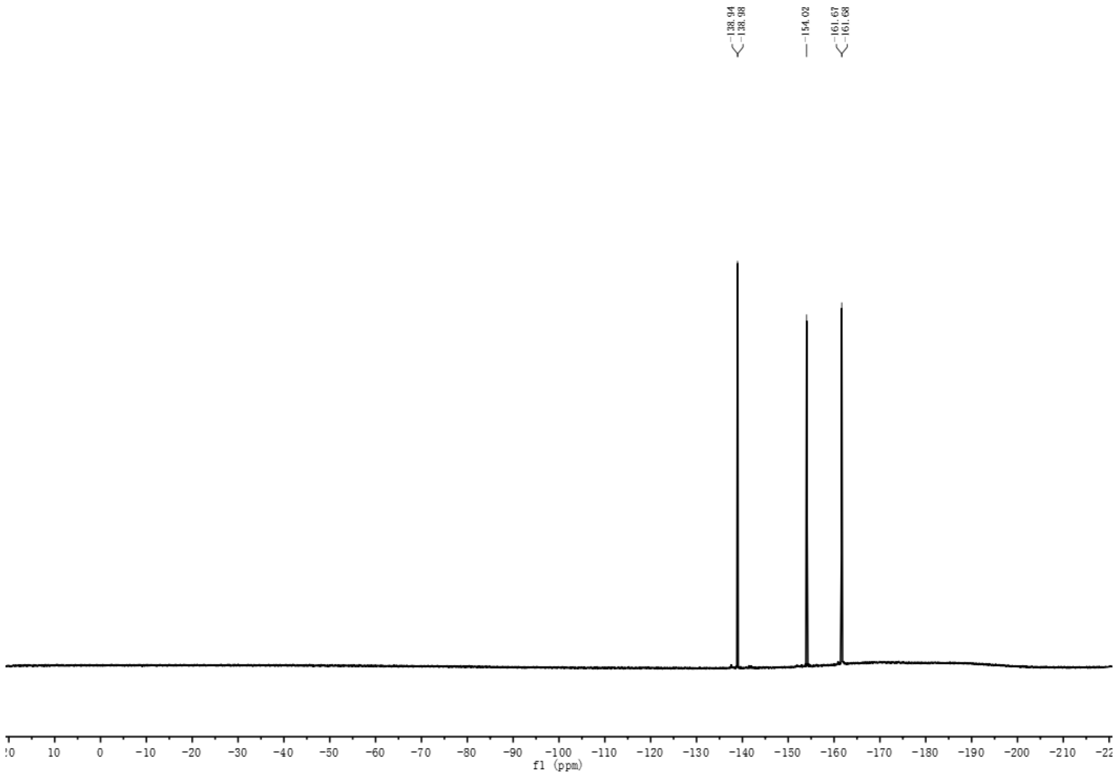

^{19}F NMR (376 MHz, $CDCl_3$)

methyl methyl (S)-2-(1-benzyl-3-((4-bromonaphthalen-1-yl)methyl)-4,6-difluoro-2-oxoindolin-3-yl)acetate (**5p**)

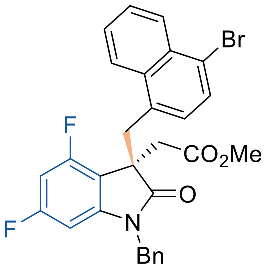

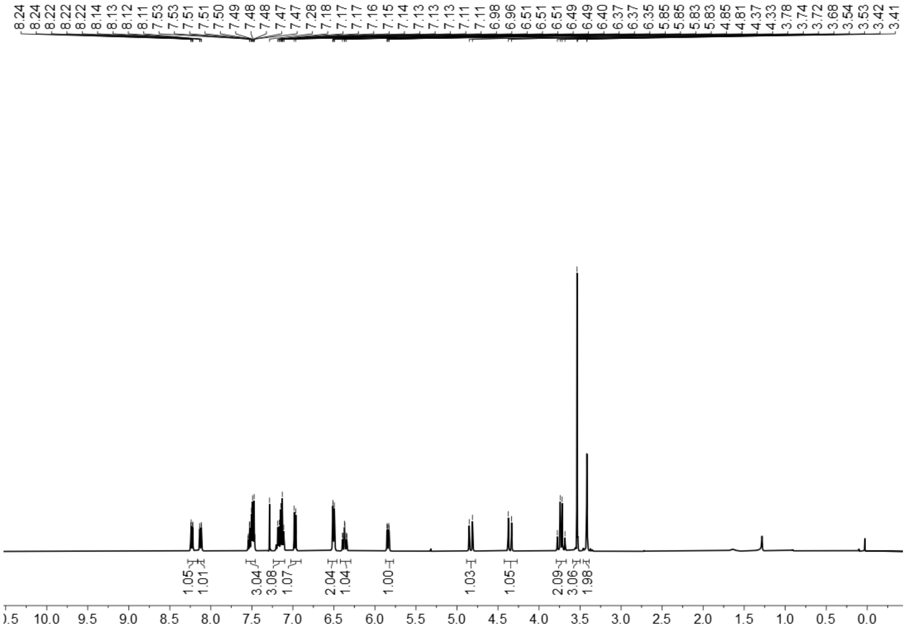

^{1}H NMR (400 MHz, $CDCl_3$)

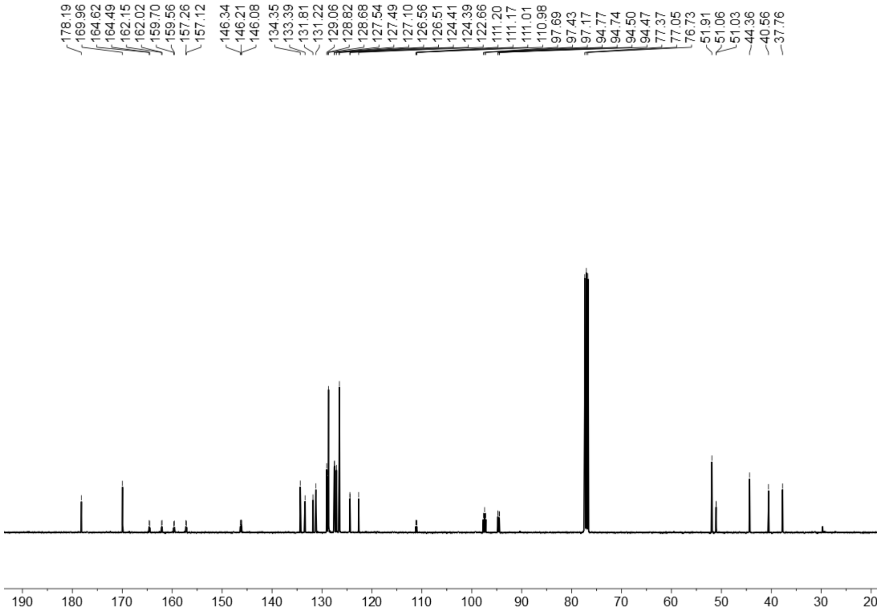

^{13}C NMR (101 MHz, $CDCl_3$)

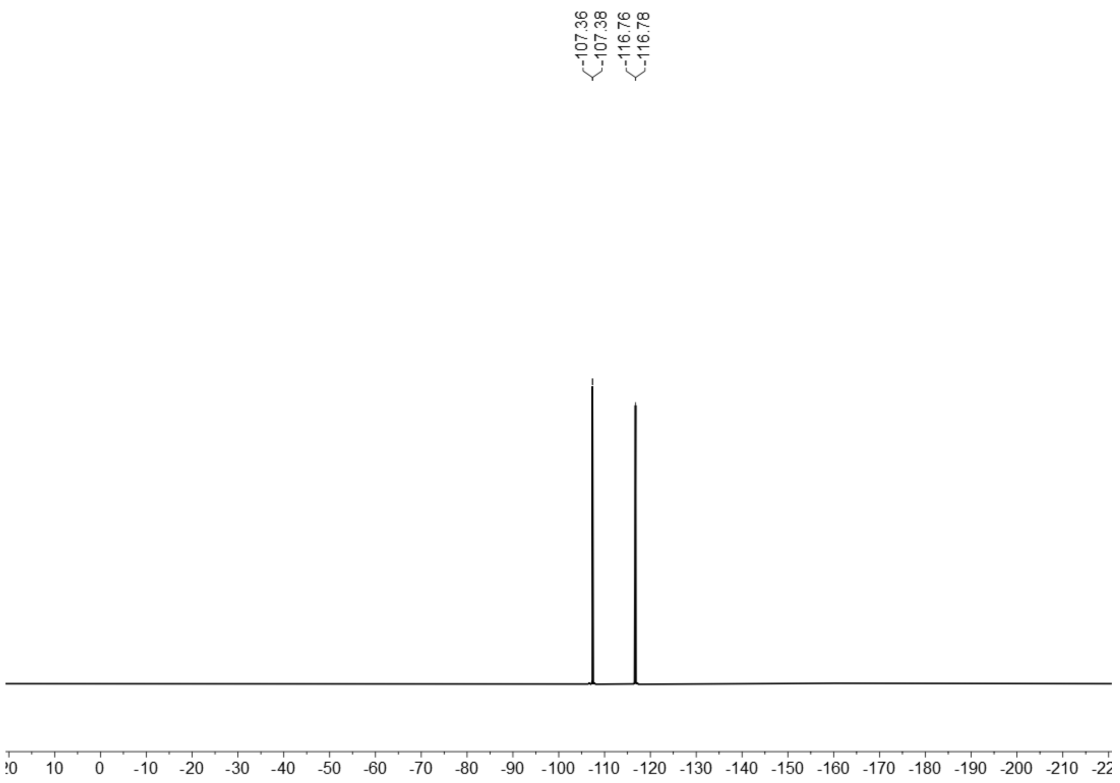

^{19}F NMR (376 MHz, $CDCl_3$)

methyl (S)-2-((1-benzyl-4,7-dichloro-3-(2-methoxy-2-oxoethyl)-2-oxoindolin-3-yl)methyl)furan-3-carboxylate (**5q**)

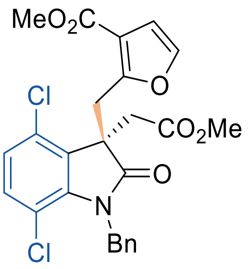

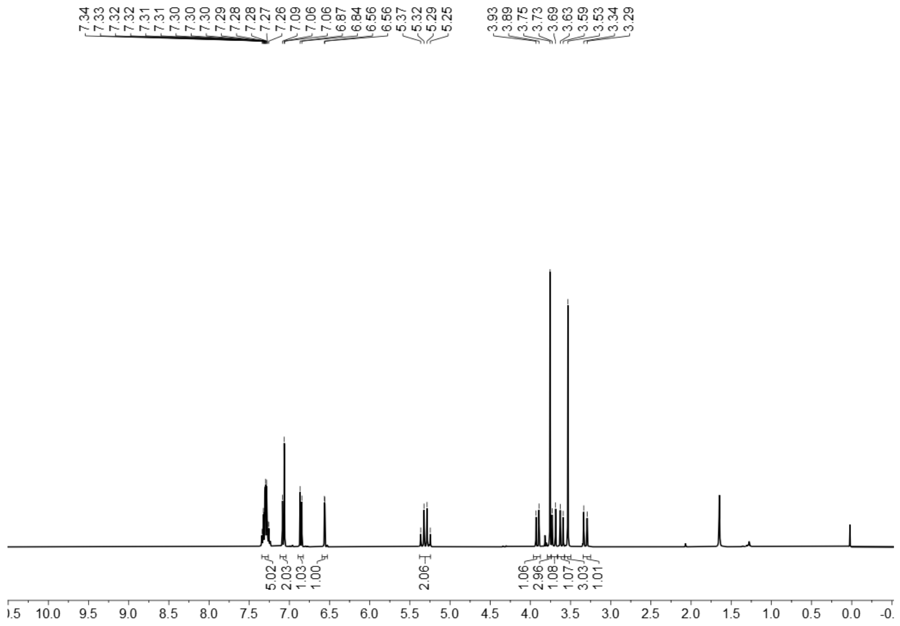

¹H NMR (400 MHz, $CDCl_3$)

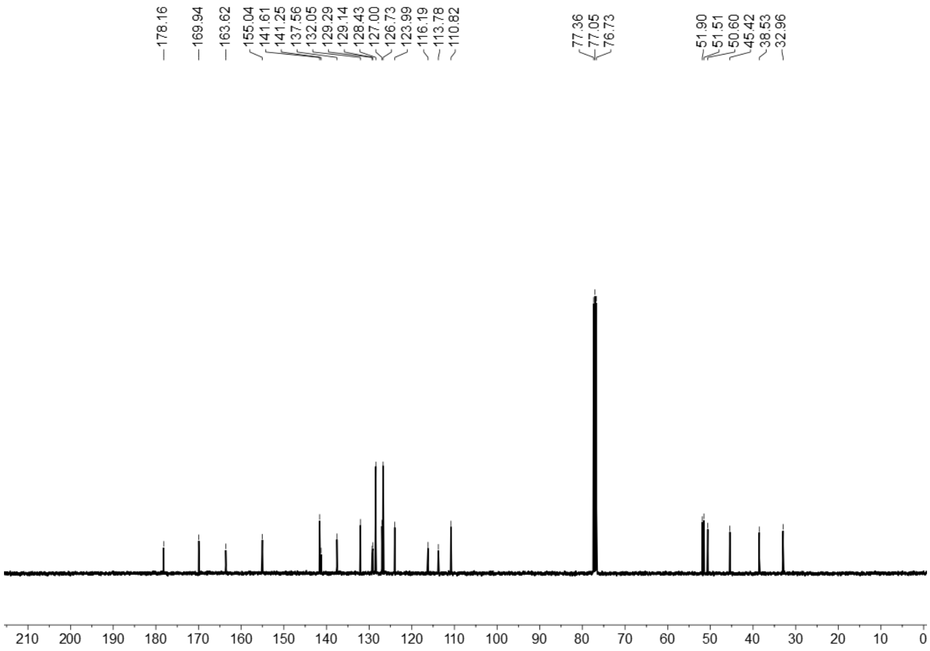

¹³C NMR (101 MHz, $CDCl_3$)

methyl (S)-2-(1-benzyl-4,7-dichloro-2-oxo-3-(thiophen-3-ylmethyl)indolin-3-yl)acetate (**5r**)

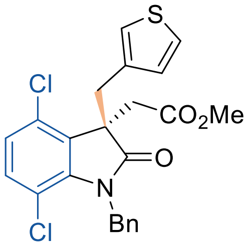

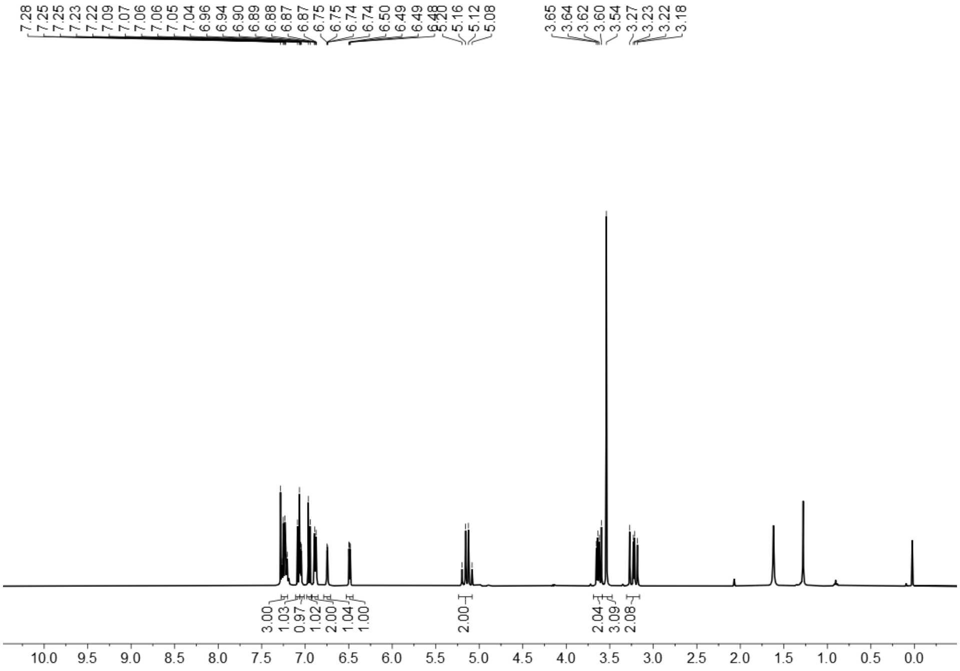

^{1}H NMR (400 MHz, $CDCl_3$)

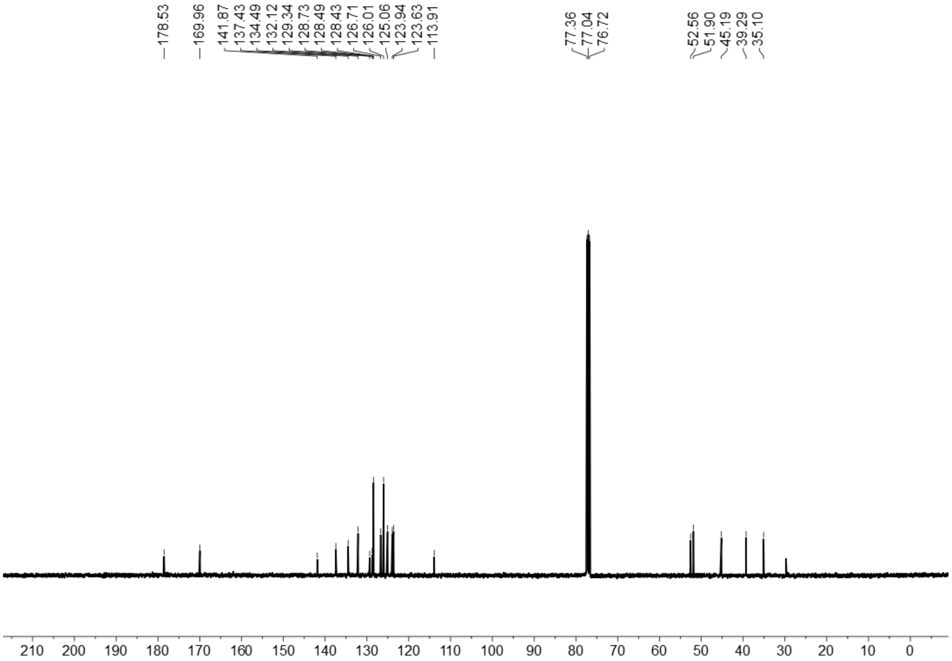

^{13}C NMR (101 MHz, $CDCl_3$)

methyl (R)-2-(1-benzyl-4,7-dichloro-3-((5-cyanothiophen-2-yl)methyl)-2-oxoindolin-3-yl)acetate (**5s**)

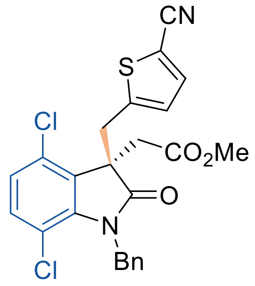

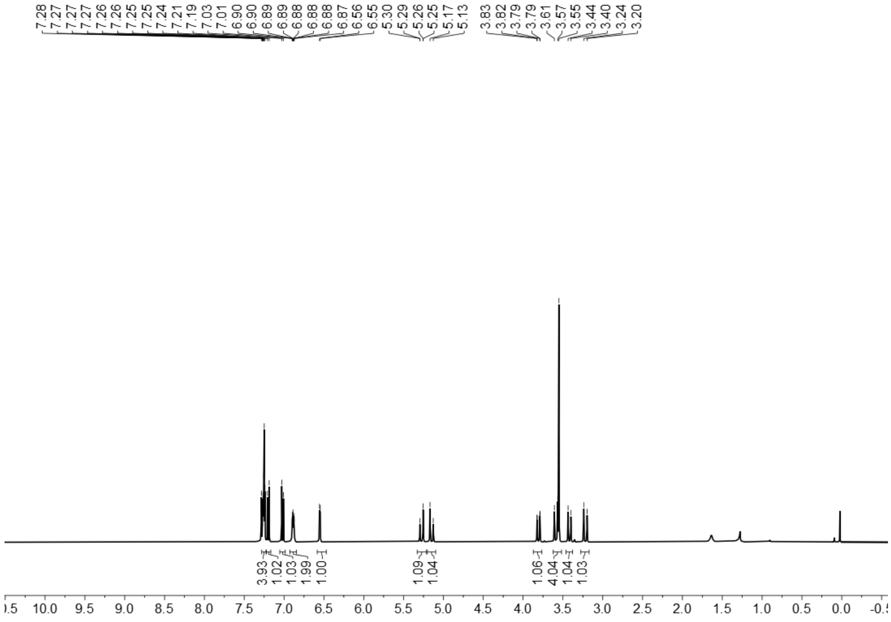

^{1}H NMR (400 MHz, $CDCl_3$)

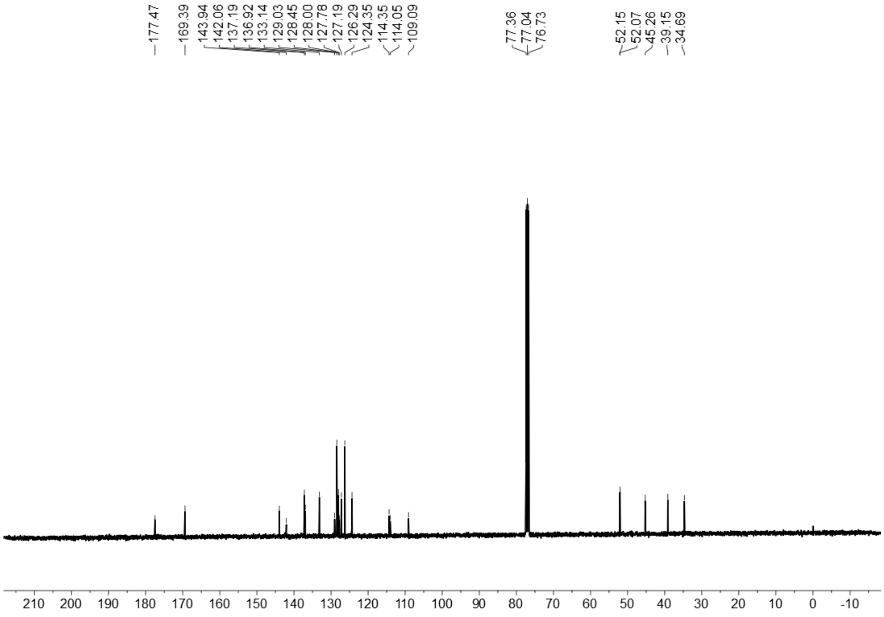

^{13}C NMR (101 MHz, $CDCl_3$)

methyl (S)-2-(3-(benzo[b]thiophen-7-ylmethyl)-1-benzyl-4,7-dichloro-2-oxoindolin-3-yl)acetate (**5t**)

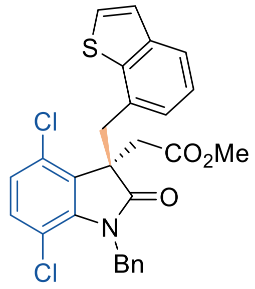

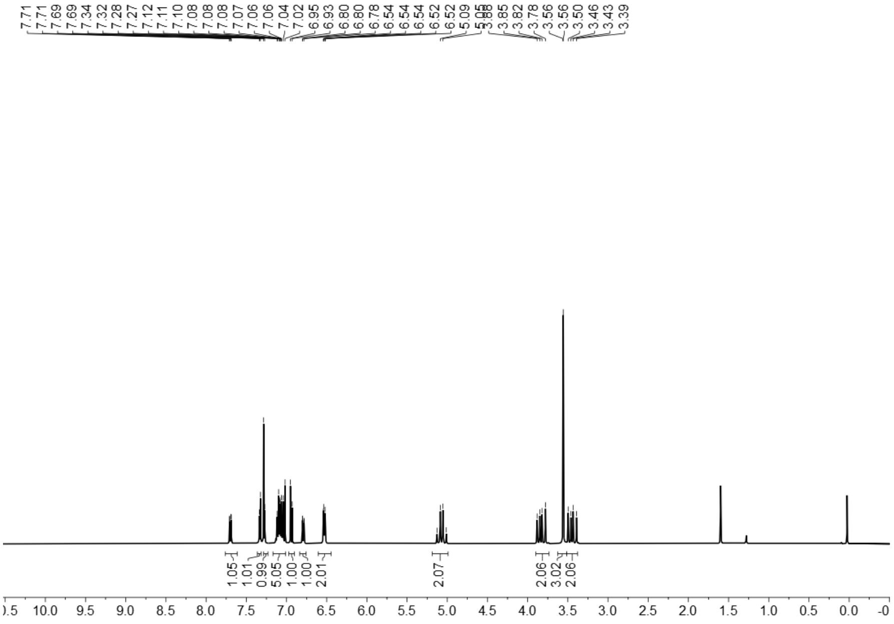

^{1}H NMR (400 MHz, $CDCl_3$)

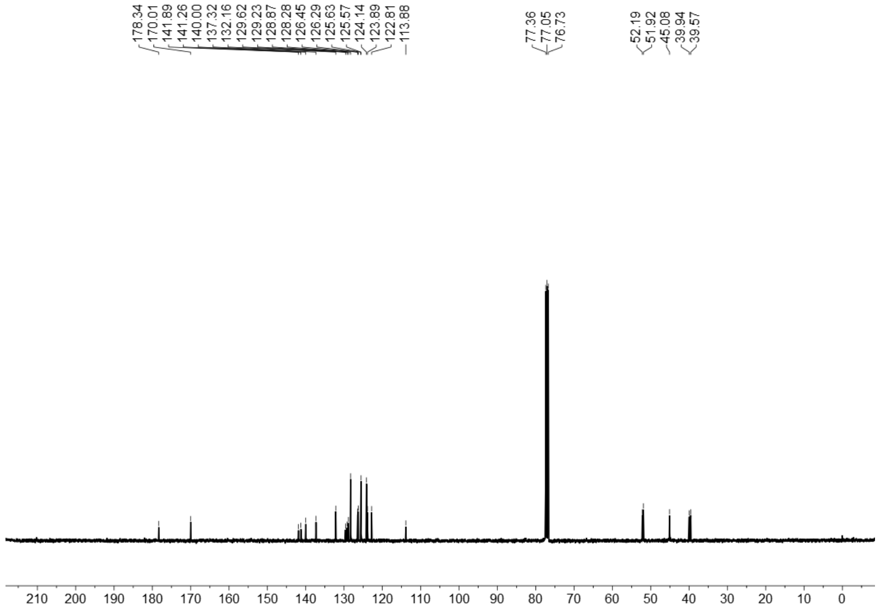

^{13}C NMR (101 MHz, $CDCl_3$)

methyl (S)-2-(1-benzyl-4,7-dichloro-3-((2-cyanopyridin-3-yl)methyl)-2-oxoindolin-3-yl)acetate (**5u**)

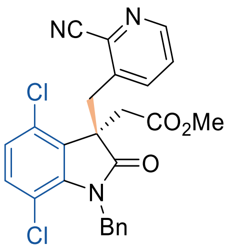

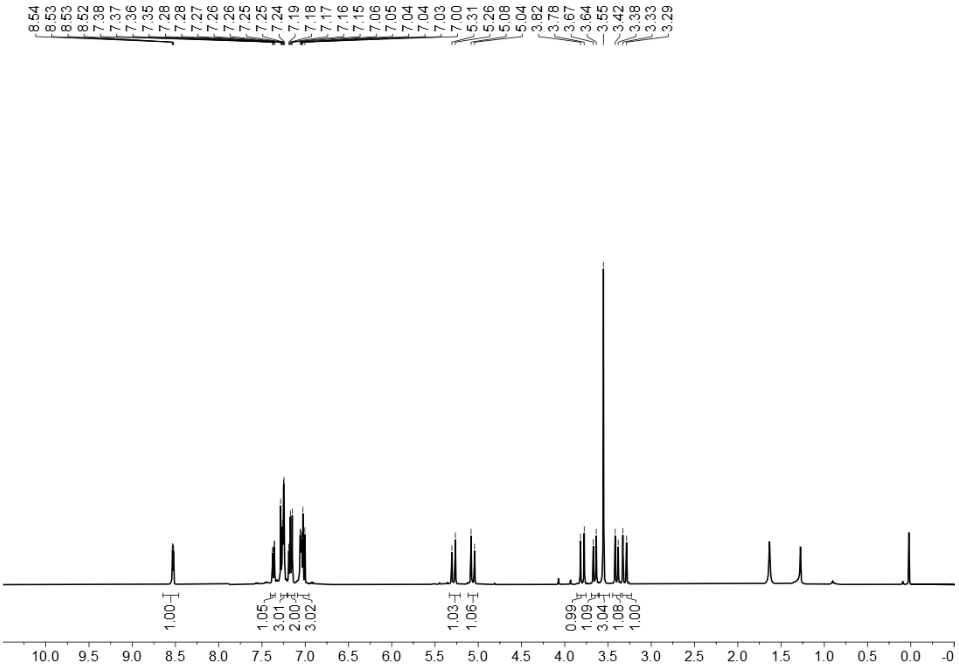

^{1}H NMR (400 MHz, $CDCl_3$)

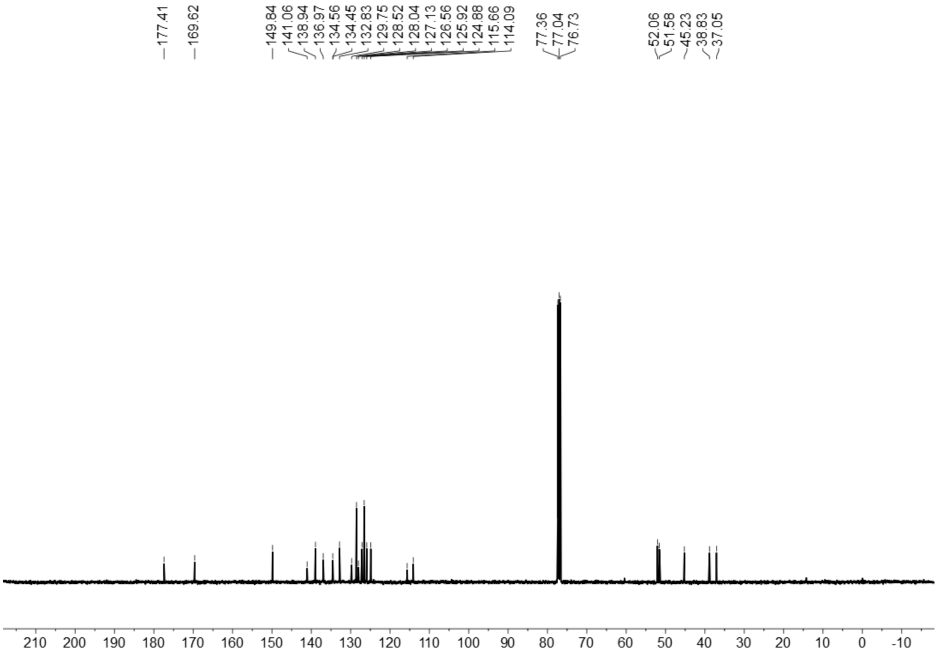

^{13}C NMR (101 MHz, $CDCl_3$)

methyl (S)-2-(1-benzyl-4,7-dichloro-2-oxo-3-(pyrazin-2-ylmethyl)indolin-3-yl)acetate (**5v**)

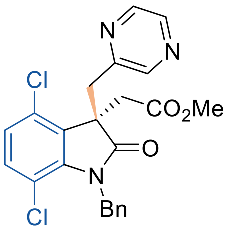

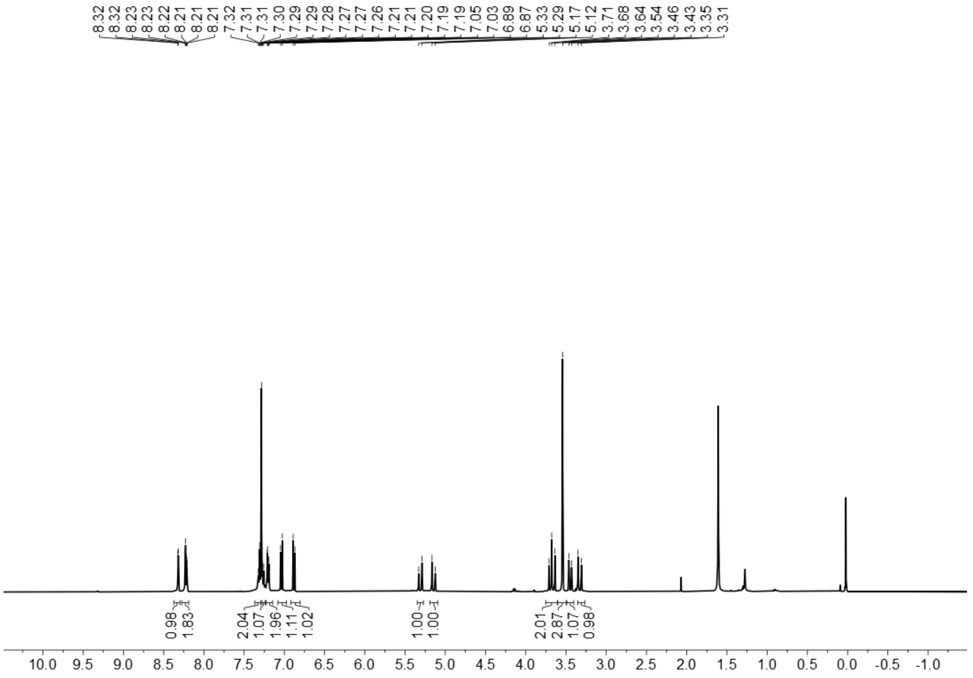

^{1}H NMR (400 MHz, $CDCl_3$)

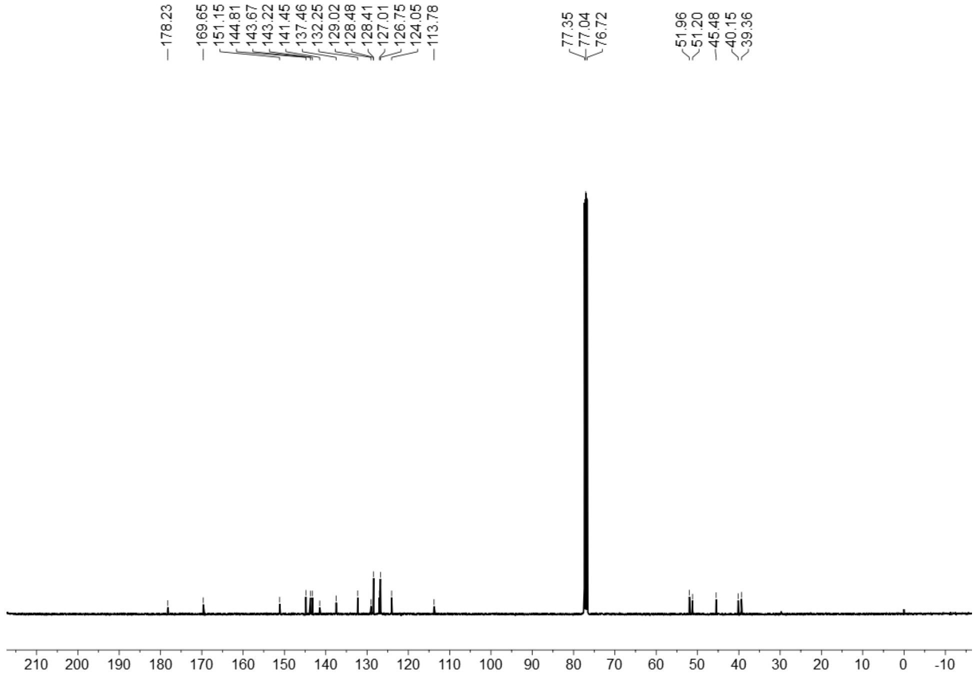

^{13}C NMR (101 MHz, $CDCl_3$)

methyl (S)-2-(1-benzyl-4,7-dichloro-2-oxo-3-(pyrimidin-5-ylmethyl)indolin-3-yl)acetate (**5w**)

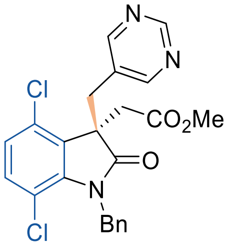

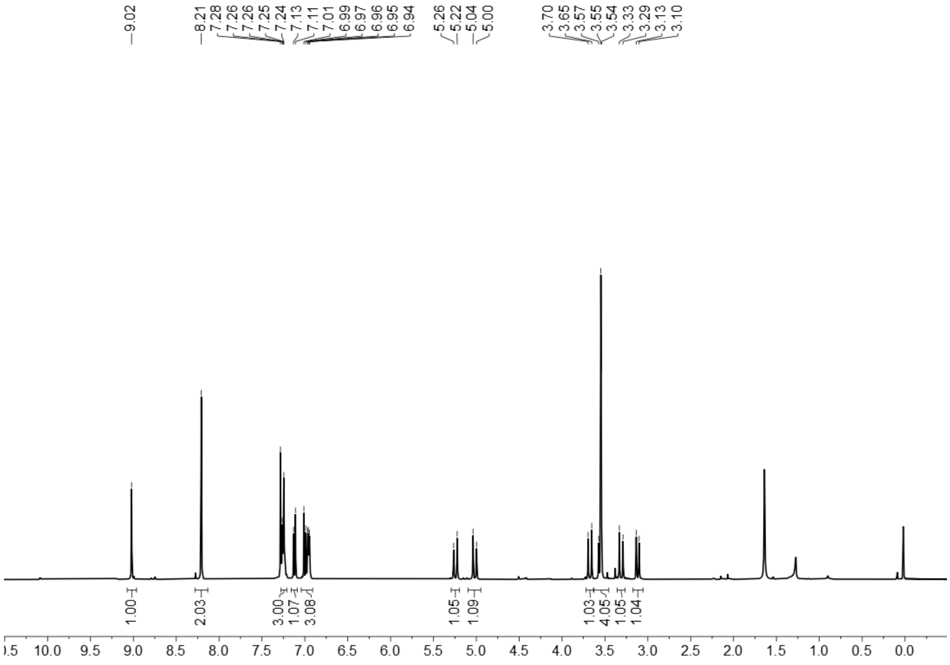

^{1}H NMR (400 MHz, $CDCl_3$)

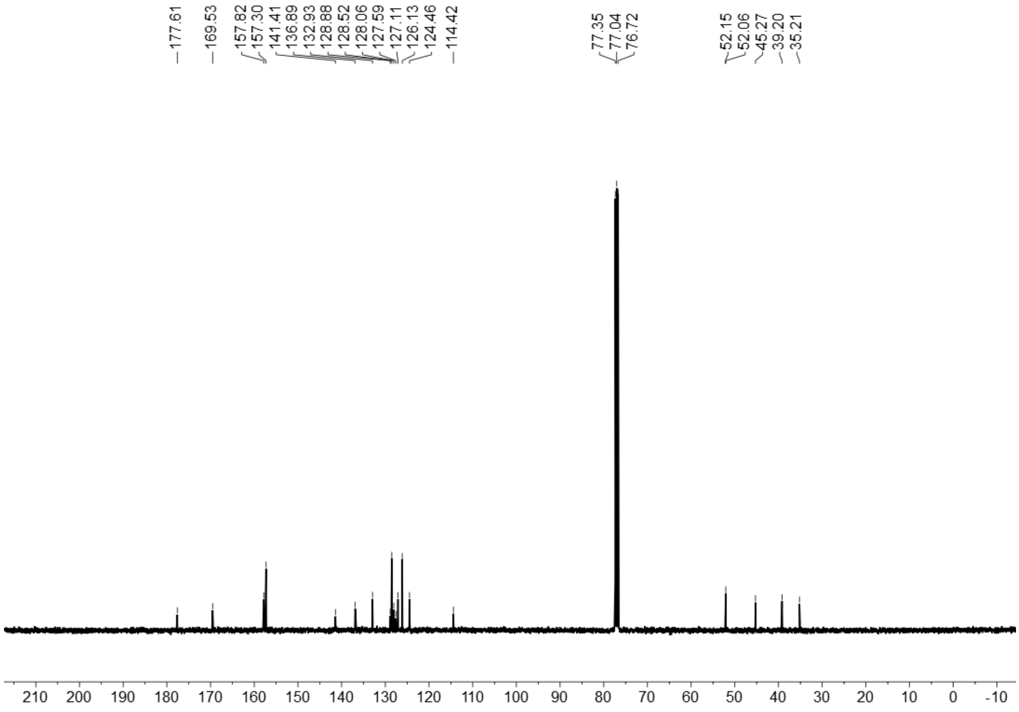

^{13}**C NMR** (101 MHz, $CDCl_3$)

tert-butyl (S)-3-((1-benzyl-4,7-dichloro-3-(2-methoxy-2-oxoethyl)-2-oxoindolin-3-yl)methyl)-1H-indole-1-carboxylate (**5x**)

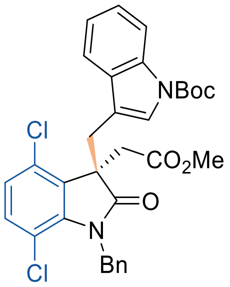

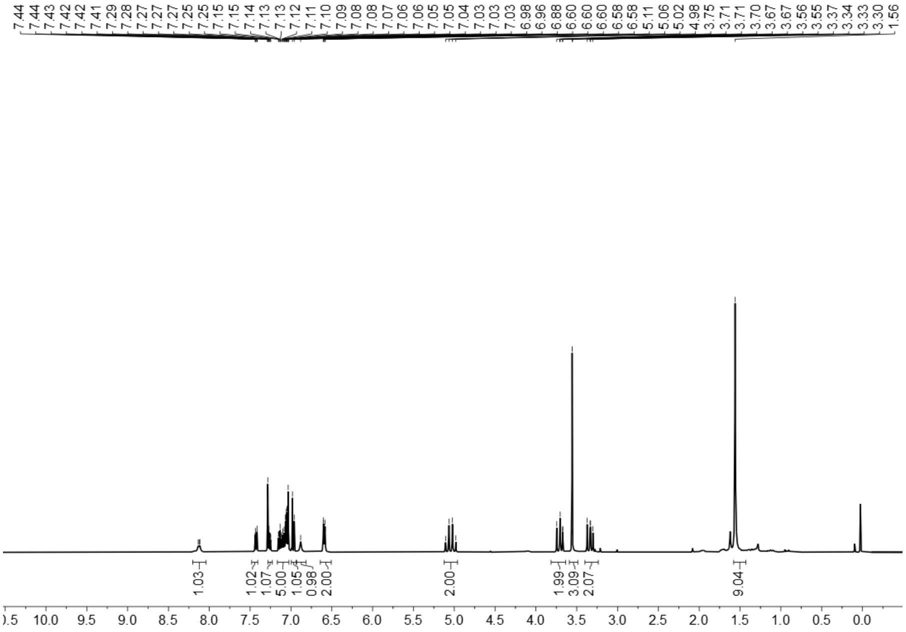

^{1}**H NMR** (400 MHz, $CDCl_3$)

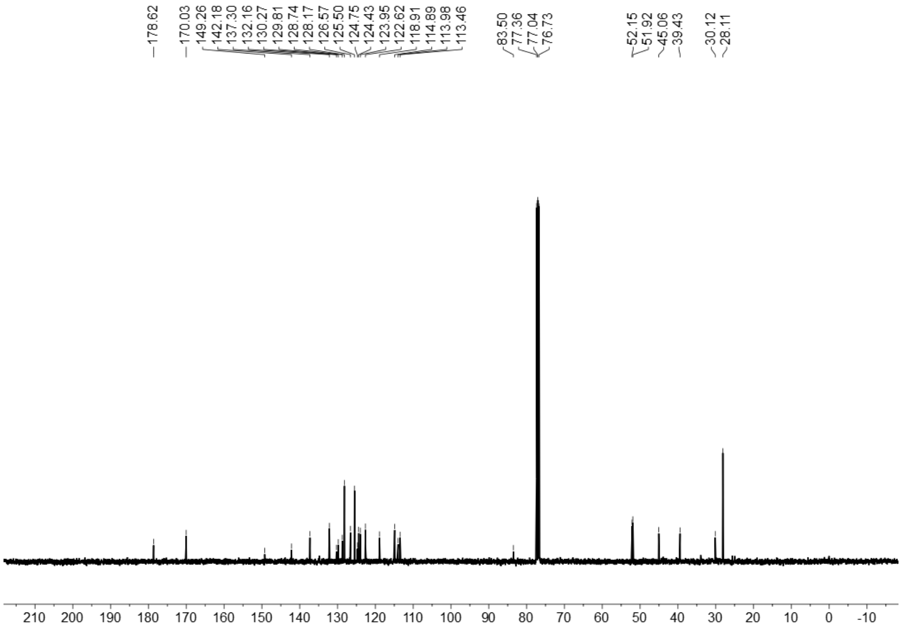

^{13}C NMR (101 MHz, $CDCl_3$)

methyl (S)-2-(1-benzyl-4,7-dichloro-2-oxo-3-(quinolin-5-ylmethyl)indolin-3-yl)acetate (**5y**)

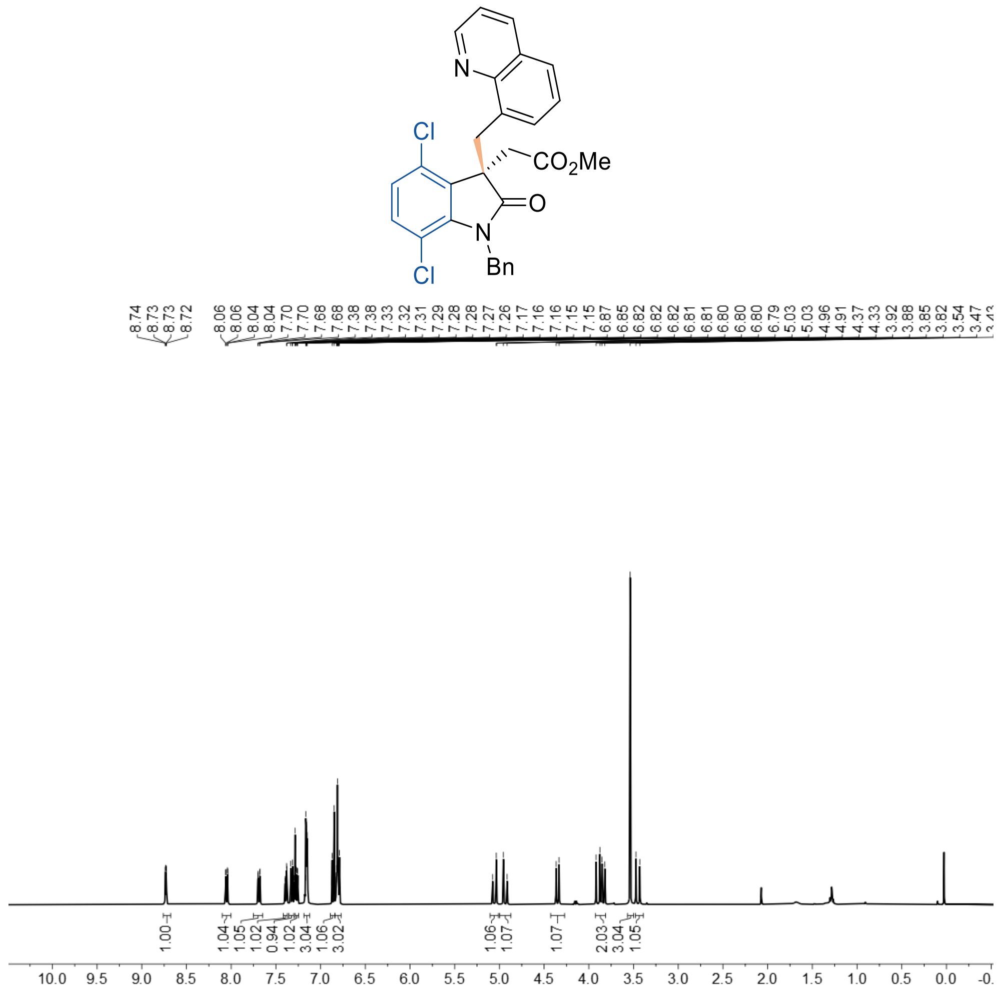

^{1}H NMR (400 MHz, $CDCl_3$)

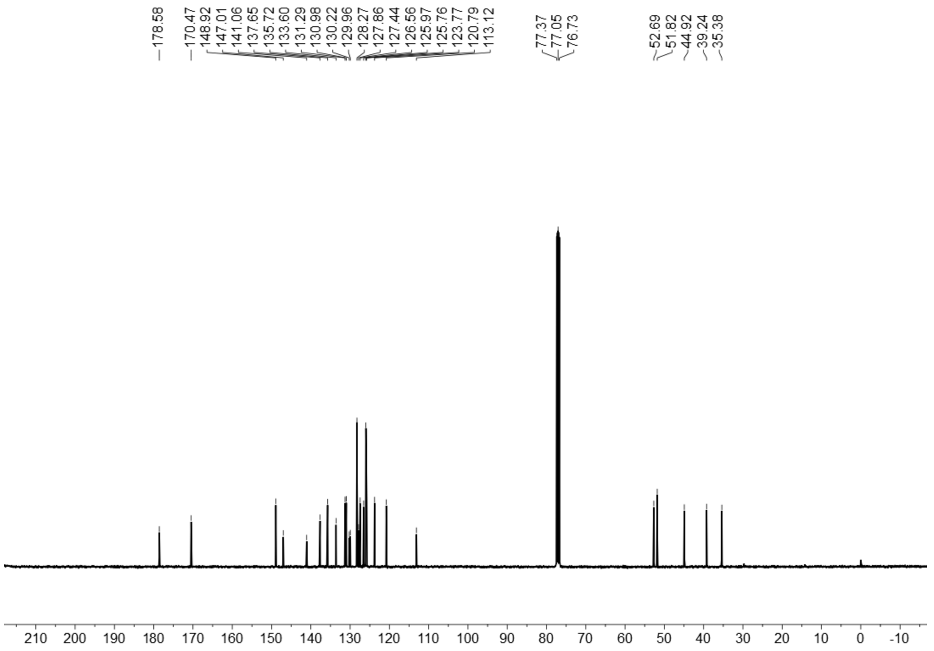

^{13}C NMR (101 MHz, $CDCl_3$)

methyl (S)-2-(1-benzyl-4,7-dichloro-2-oxo-3-(quinoxalin-5-ylmethyl)indolin-3-yl)acetate (**5z**)

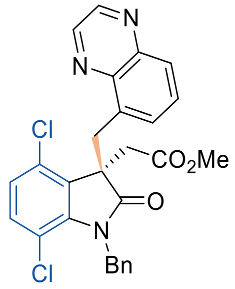

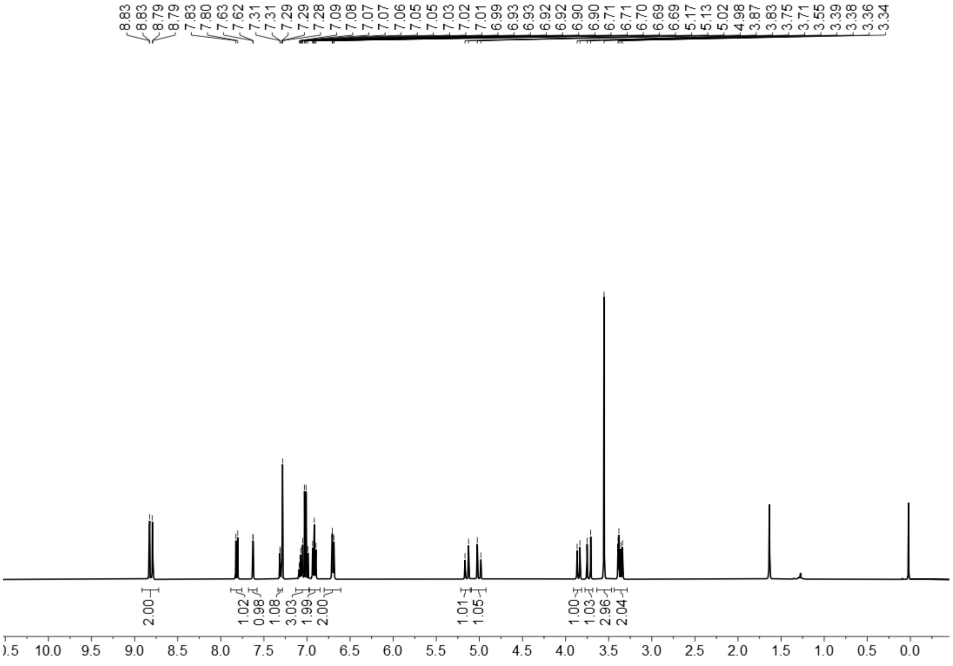

^{1}H NMR (400 MHz, $CDCl_3$)

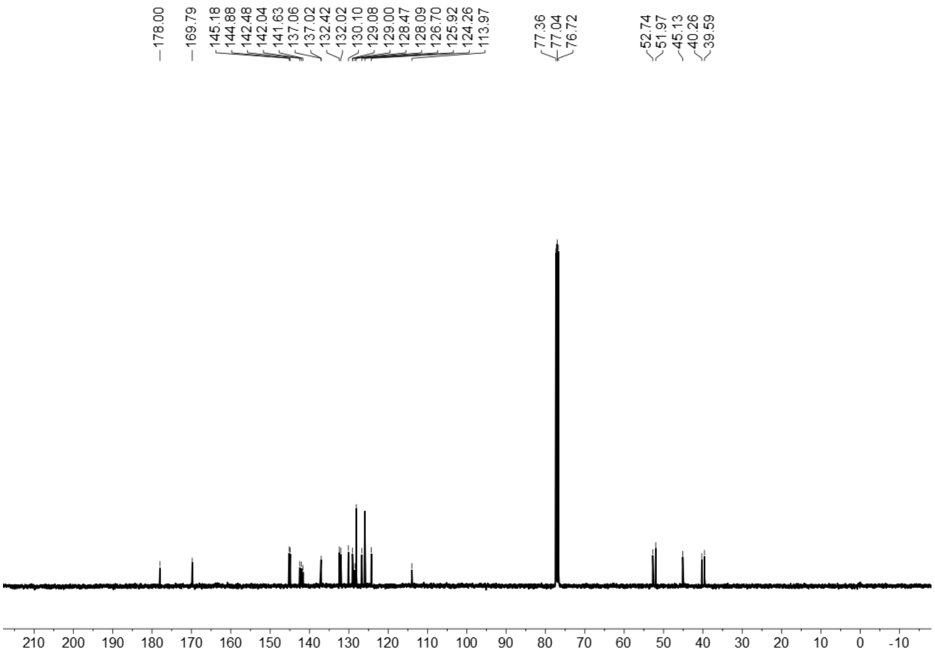

^{13}C NMR (101 MHz, $CDCl_3$)

methyl (S)-2-(1-benzyl-4,7-dichloro-3-((7-methoxy-2-oxo-2H-chromen-4-yl)methyl)-2-oxoindolin-3-yl)acetate (**5aa**)

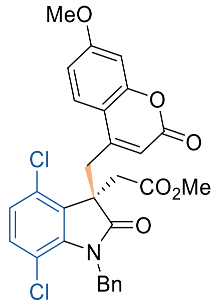

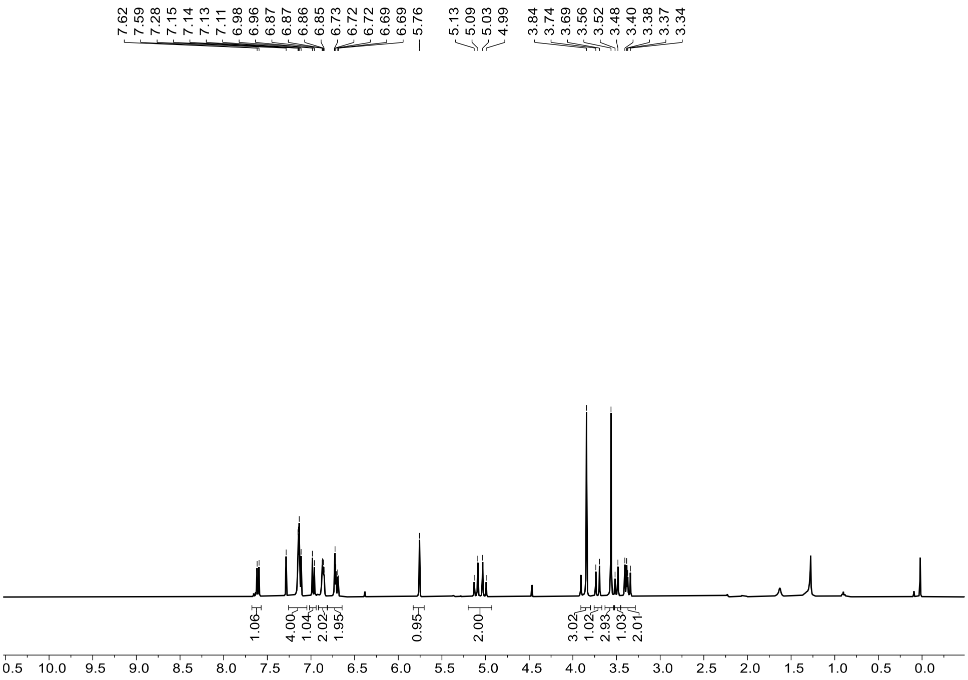

^{1}H NMR (400 MHz, $CDCl_3$)

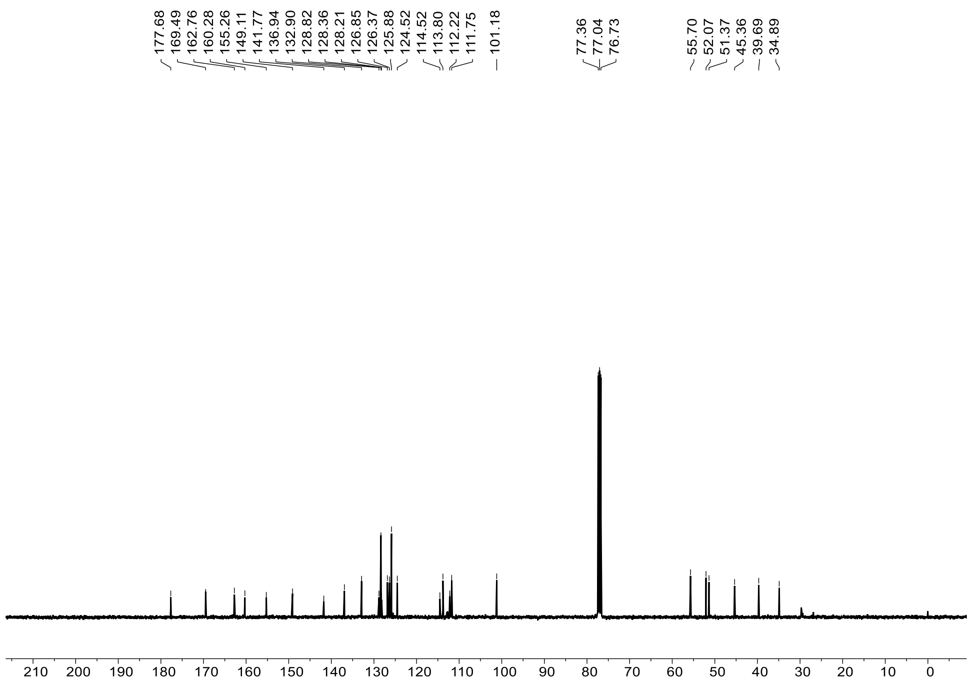

^{13}C NMR (101 MHz, $CDCl_3$)

4-(((S)-1-benzyl-4,7-dichloro-3-(2-methoxy-2-oxoethyl)-2-oxoindolin-3-yl)methyl)phenyl (S)-2-(6-methoxynaphthalen-2-yl)propanoate (**5ab**)

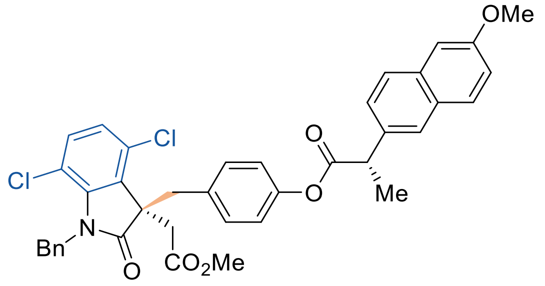

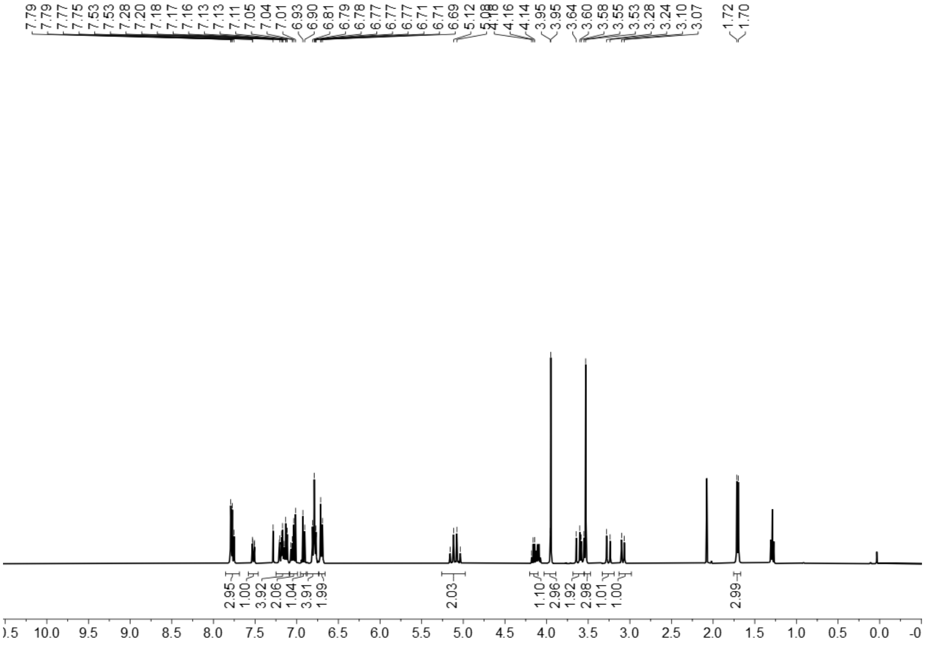

^{1}H NMR (400 MHz, $CDCl_3$)

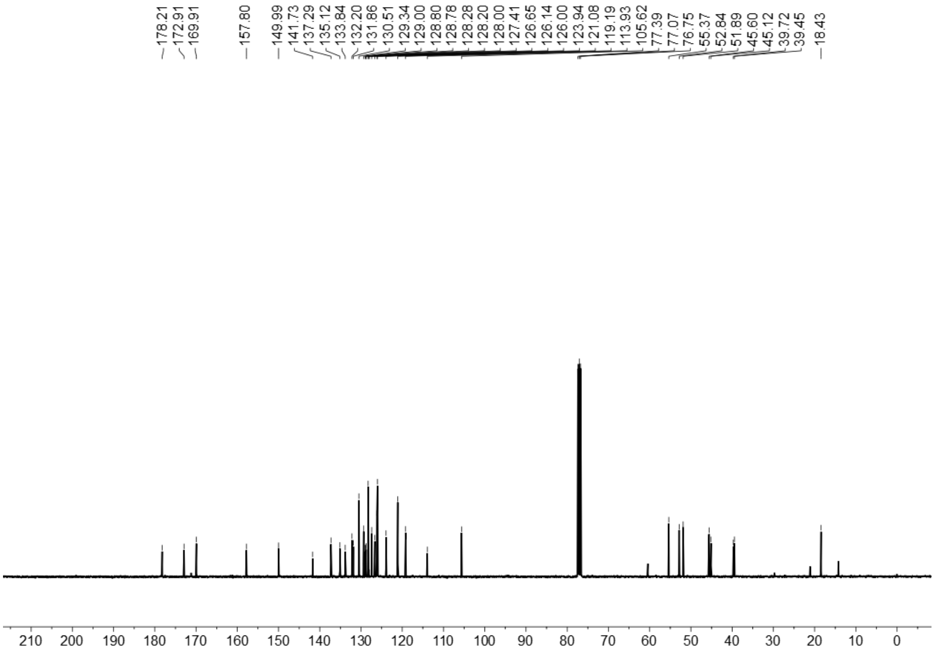

^{13}C NMR (101 MHz, $CDCl_3$)

methyl (S)-2-(1-benzyl-4,7-dichloro-3-((4-(4-fluorophenyl)-6-isopropyl-2-(N-methylmethylsulfonamido)pyrimidin-5-yl)methyl)-2-oxoindolin-3-yl)acetate (**5ac**)

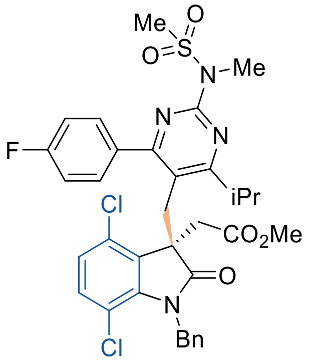

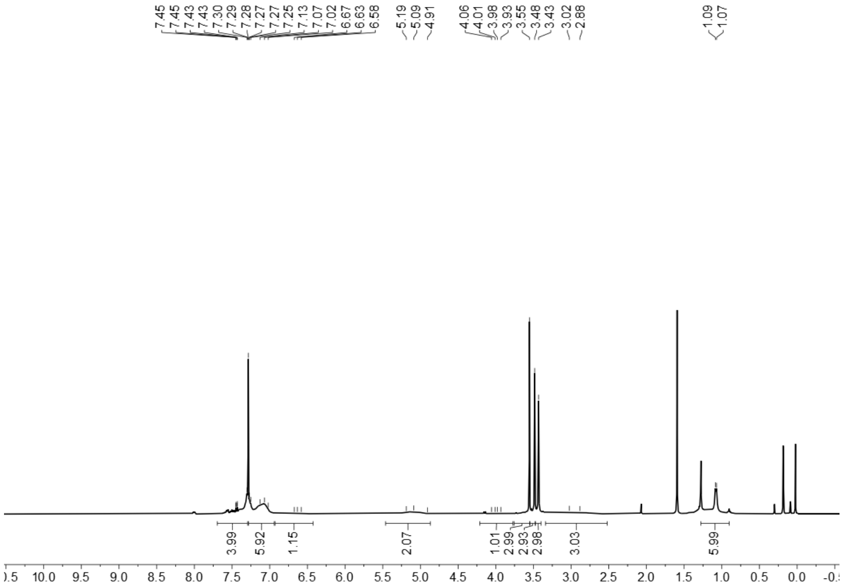

^{1}H NMR (400 MHz, $CDCl_3$)

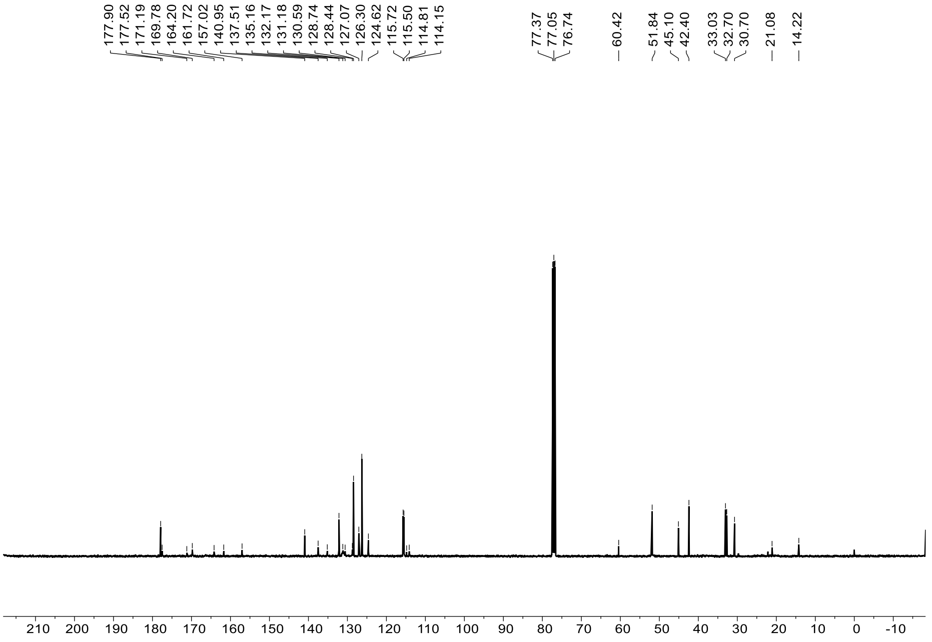

^{13}C NMR (101 MHz, $CDCl_3$)

(R)-2,5,7,8-tetramethyl-2-((4R,8S)-4,8,11-trimethyldodecyl)chroman-6-yl 4-(((S)-1-benzyl-4,7-dichloro-3-(2-methoxy-2-oxoethyl)-2-oxoindolin-3-yl)methyl)benzoate (**5ad**)

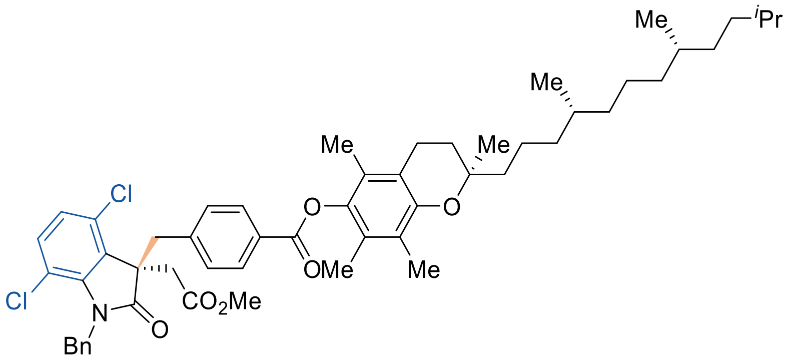

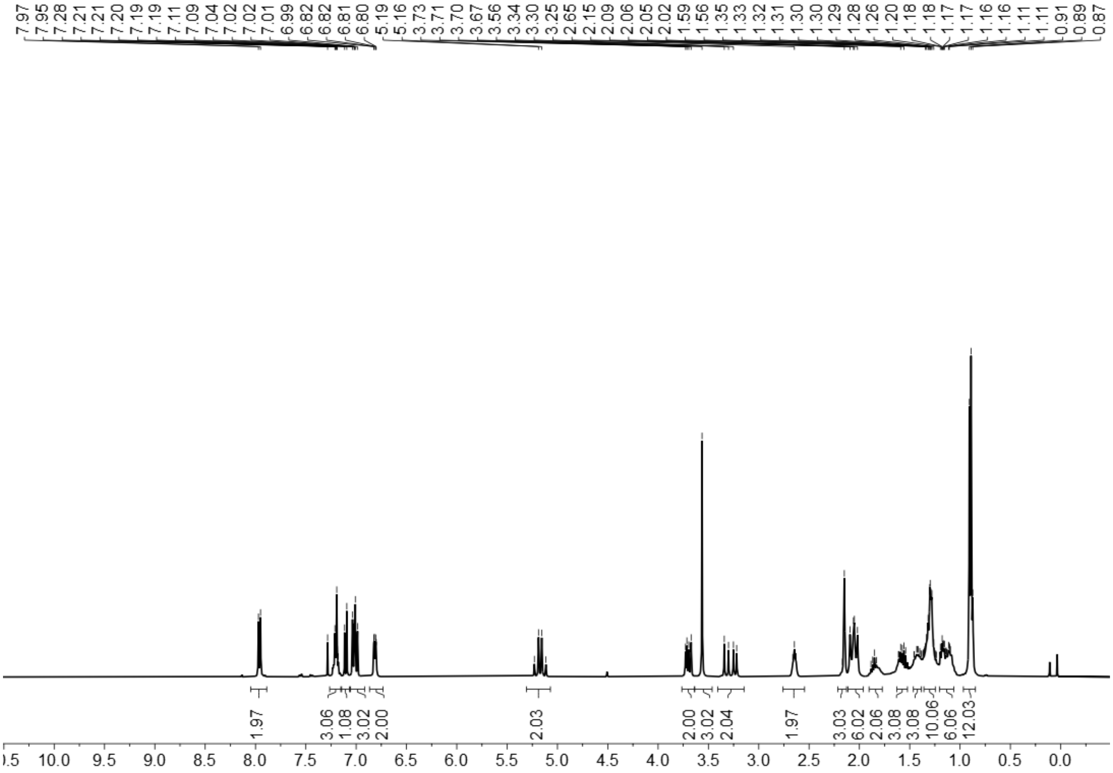

^{1}H NMR (400 MHz, $CDCl_3$)

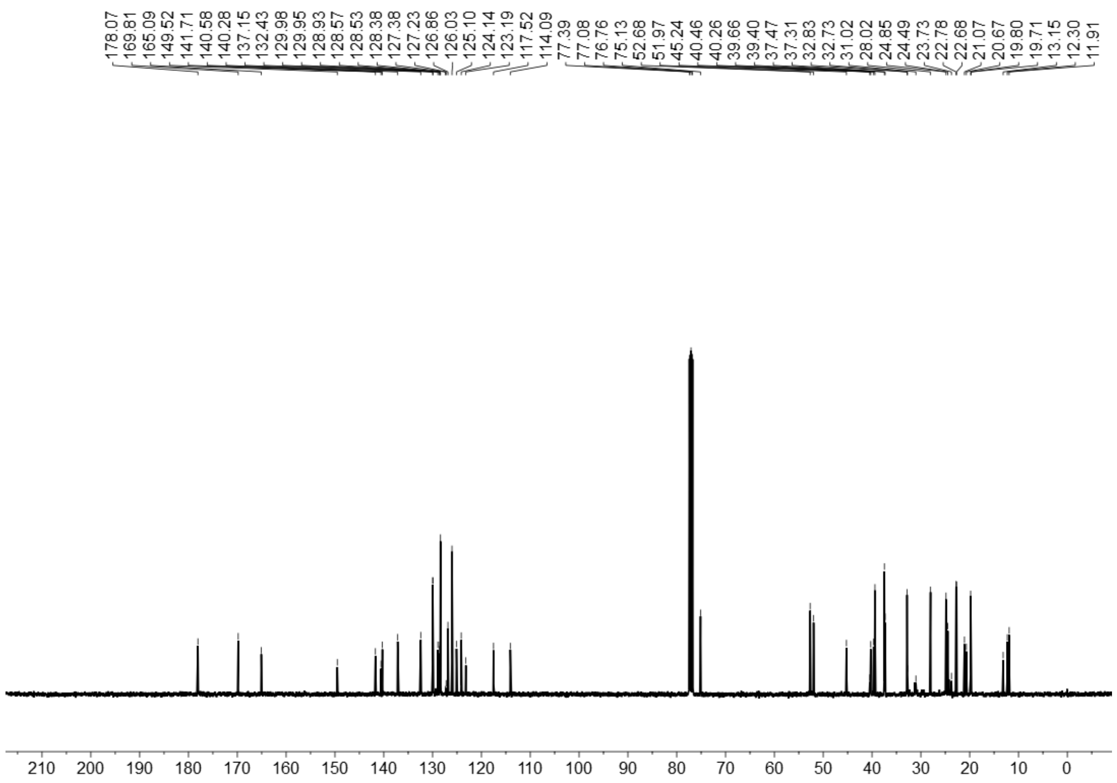

^{13}C NMR (101 MHz, $CDCl_3$)

methyl (S)-2-(1-benzyl-3-cinnamyl-4,6-difluoro-2-oxoindolin-3-yl)acetate (**6a**)

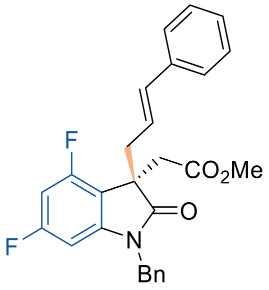

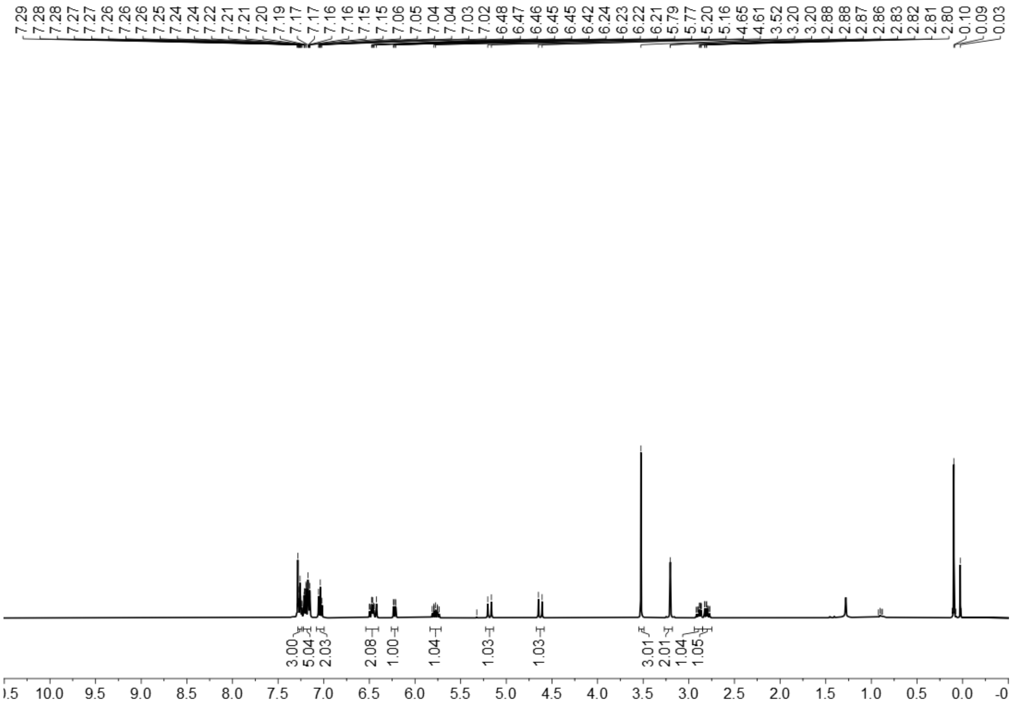

^{1}H NMR (400 MHz, $CDCl_3$)

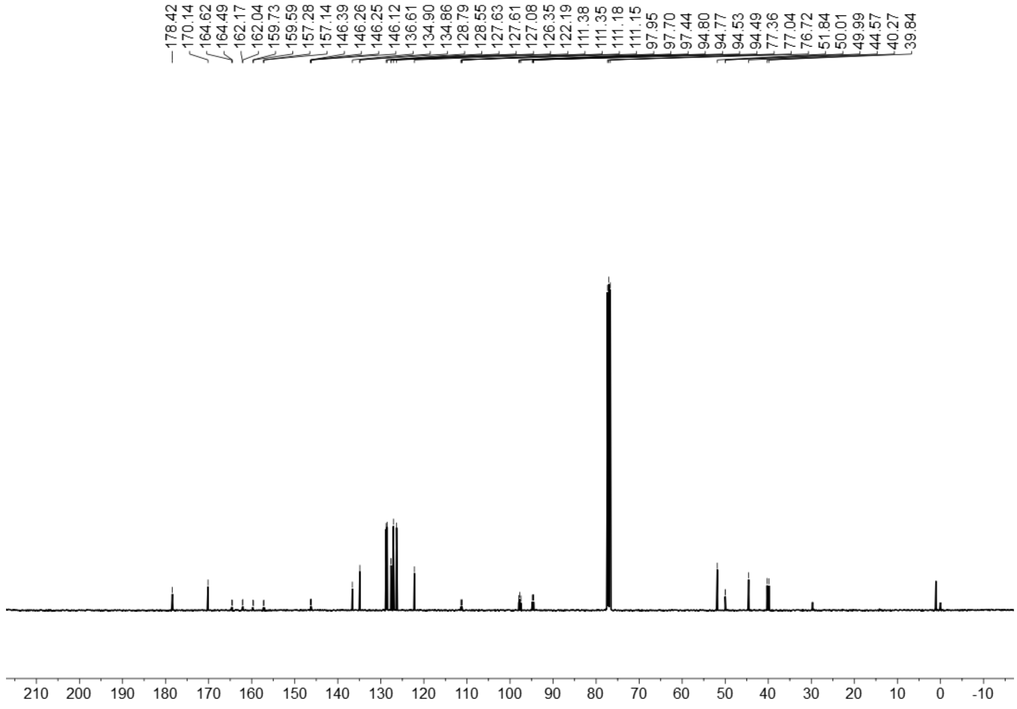

^{13}C NMR (101 MHz, $CDCl_3$)

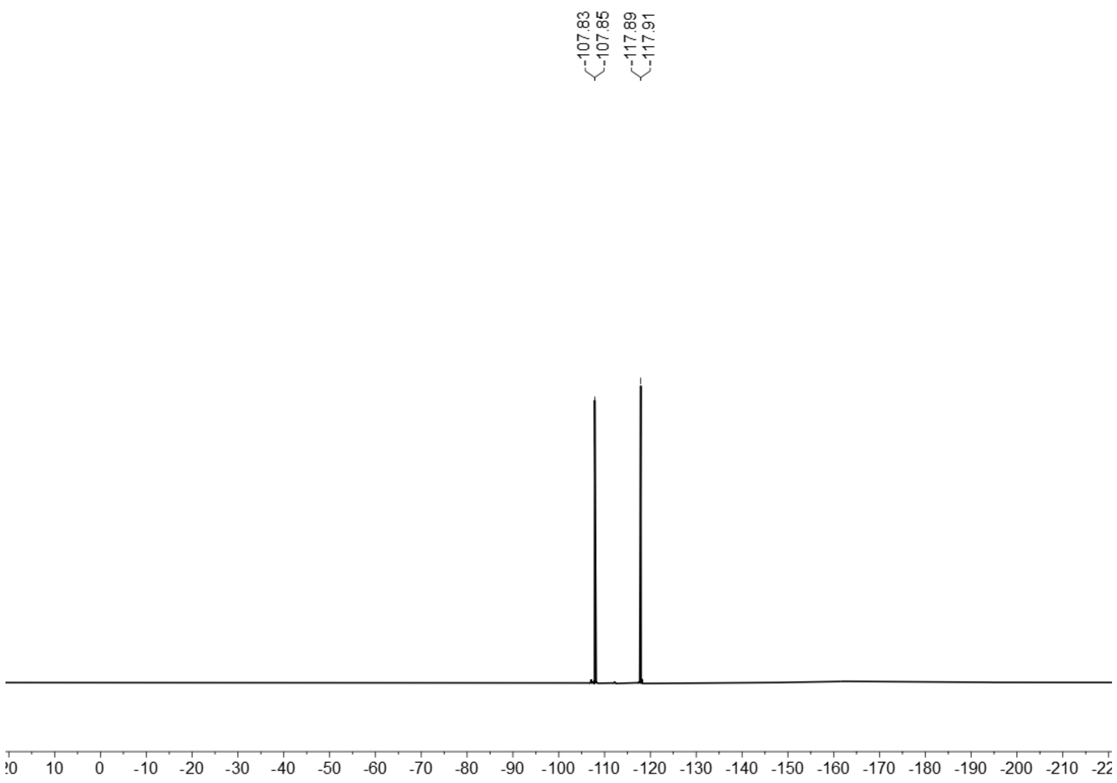

^{19}F NMR (376 MHz, $CDCl_3$)

methyl (S,E)-4-(1-benzyl-4,7-dichloro-3-(2-methoxy-2-oxoethyl)-2-oxoindolin-3-yl)but-2-enoate (**6b**)

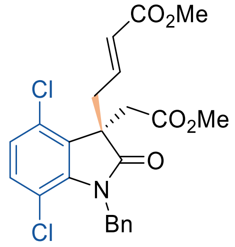

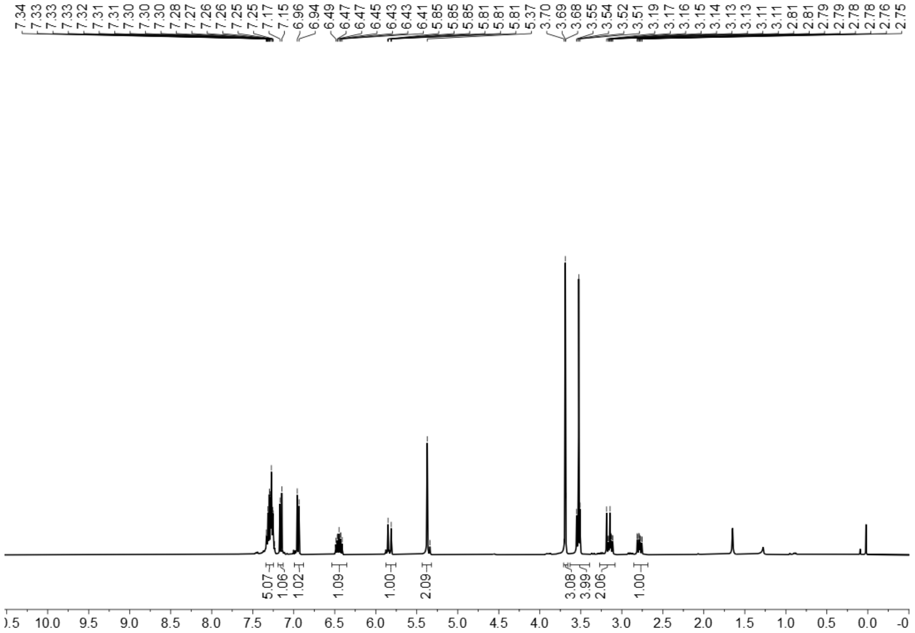

^{1}H NMR (400 MHz, $CDCl_3$)

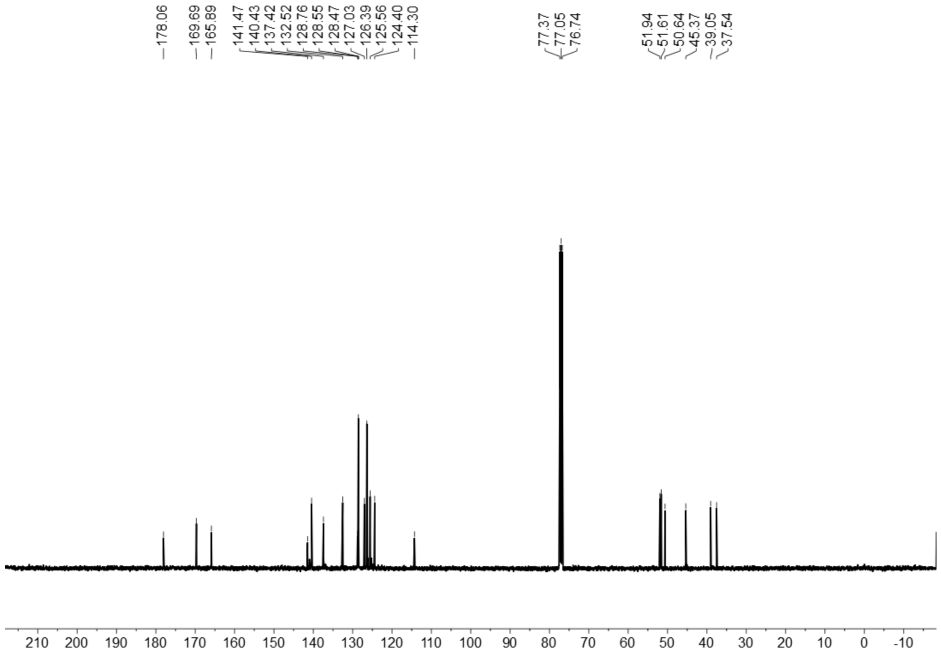

^{13}C NMR (101 MHz, $CDCl_3$)

methyl (S)-2-(1-benzyl-4,7-dichloro-3-(3-methylbut-2-en-1-yl)-2-oxoindolin-3-yl)acetate (**6c**)

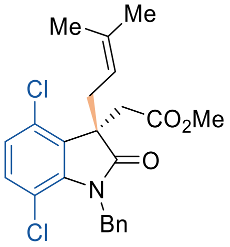

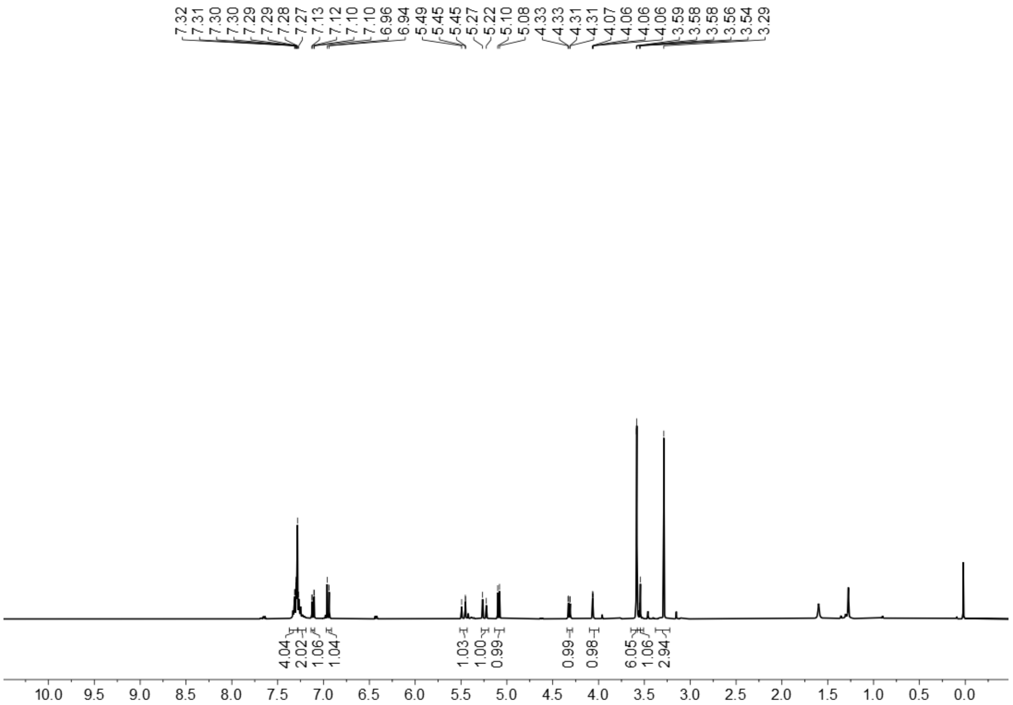

^{1}H NMR (400 MHz, $CDCl_3$)

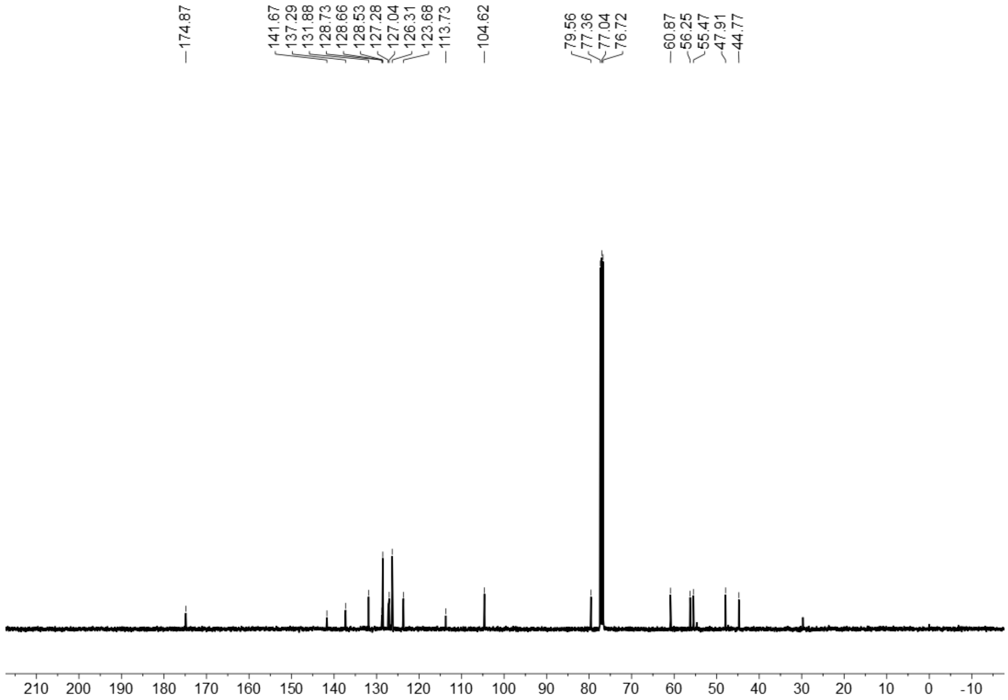

^{13}C NMR (101 MHz, $CDCl_3$)

methyl (S)-2-(1-benzyl-4,7-dichloro-3-(2-methylallyl)-2-oxoindolin-3-yl)acetate (**6d**)

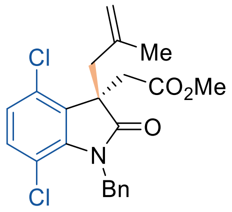

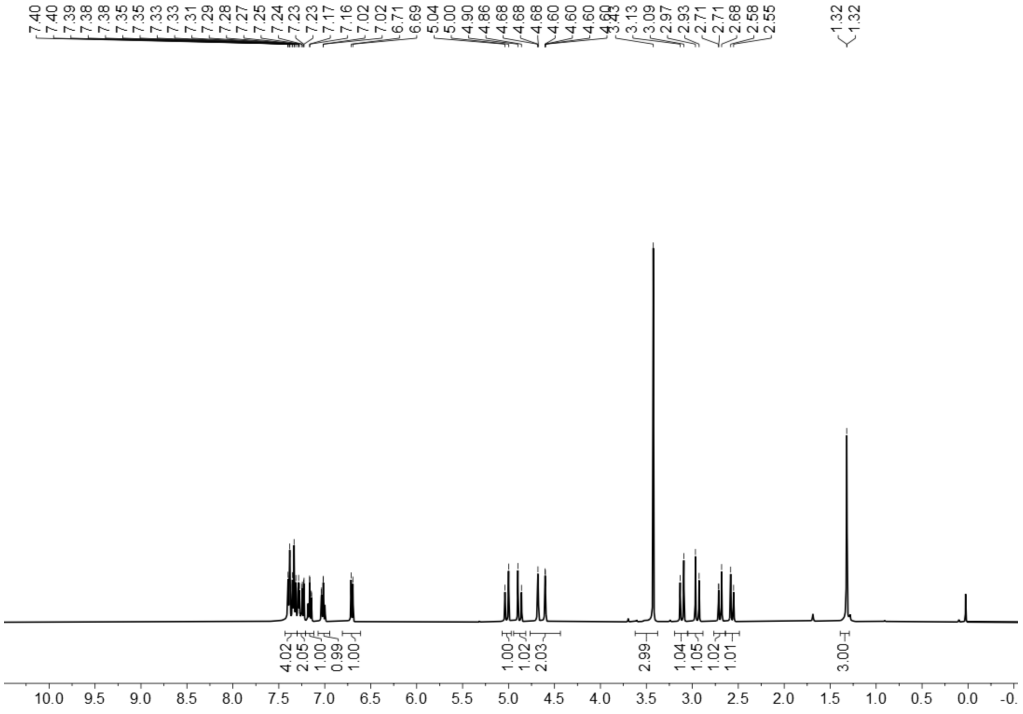

^{1}H NMR (400 MHz, $CDCl_3$)

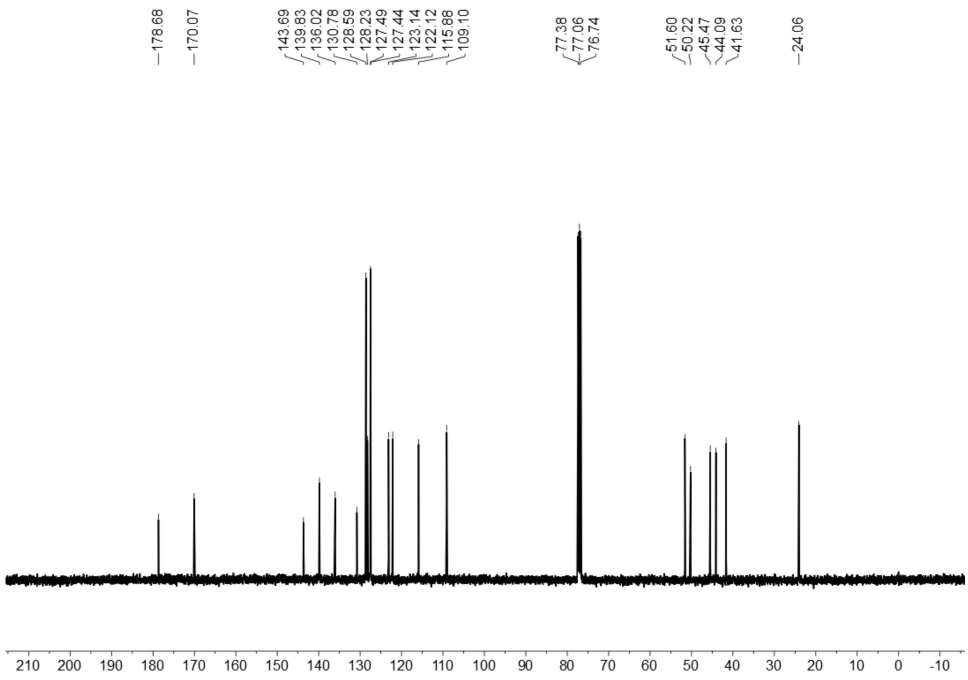

^{13}C NMR (101 MHz, $CDCl_3$)

methyl (S)-2-(1-benzyl-4,6-difluoro-2-oxo-3-(3-phenylprop-2-yn-1-yl)indolin-3-yl)acetate (**6e**)

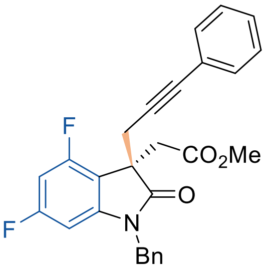

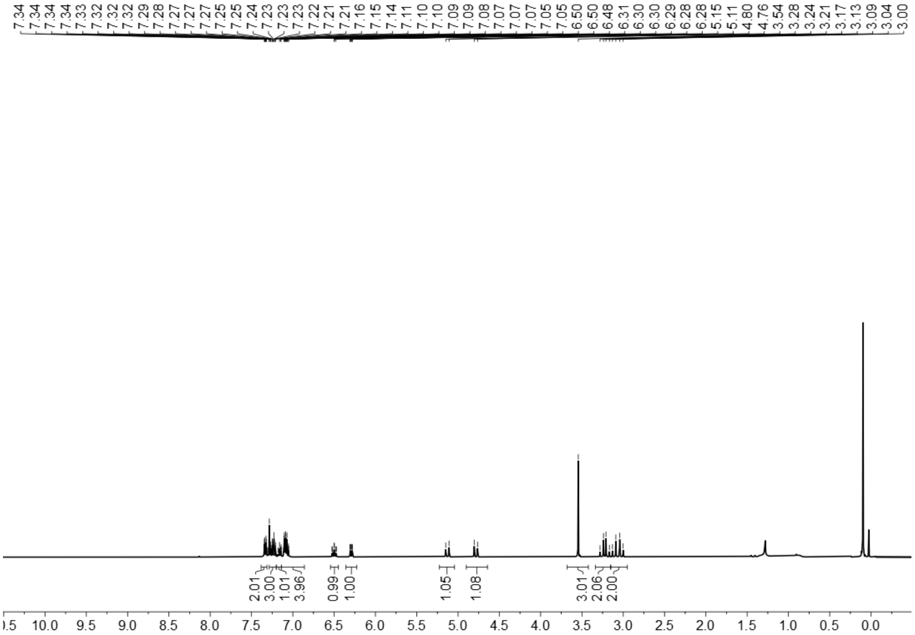

^{1}H NMR (400 MHz, $CDCl_3$)

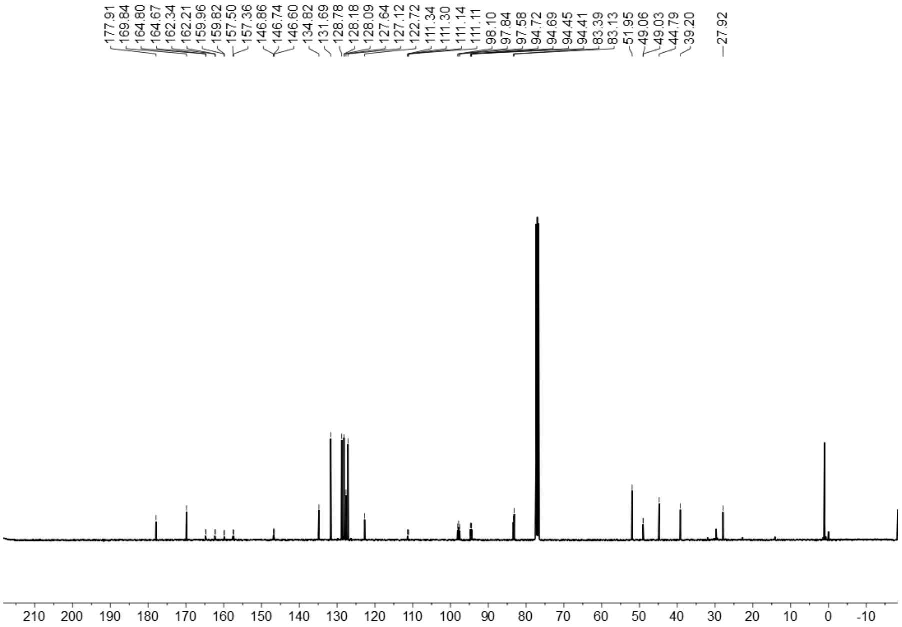

^{13}C NMR (101 MHz, $CDCl_3$)

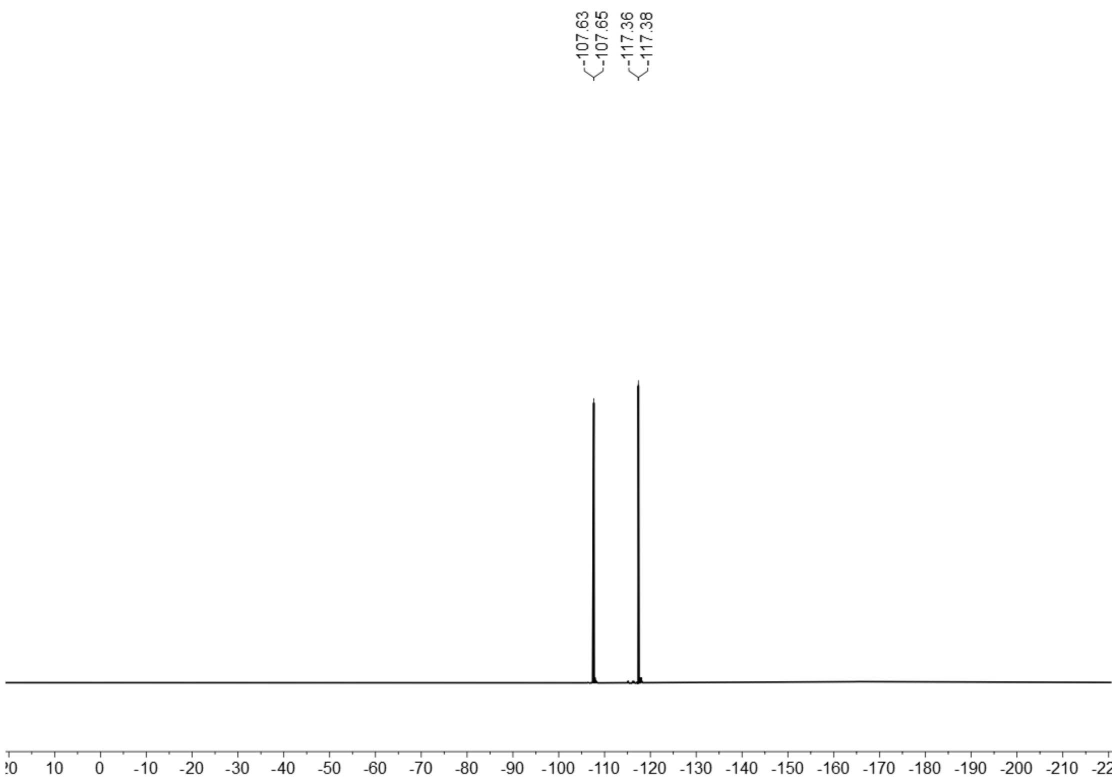

^{19}F NMR (376 MHz, $CDCl_3$)

(S)-2-(1,3-bis(3-methylbut-2-en-1-yl)-2-oxoindolin-3-yl)-N-methylacetamide (**7a**)

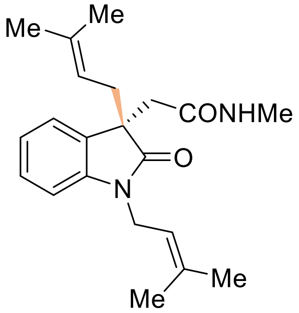

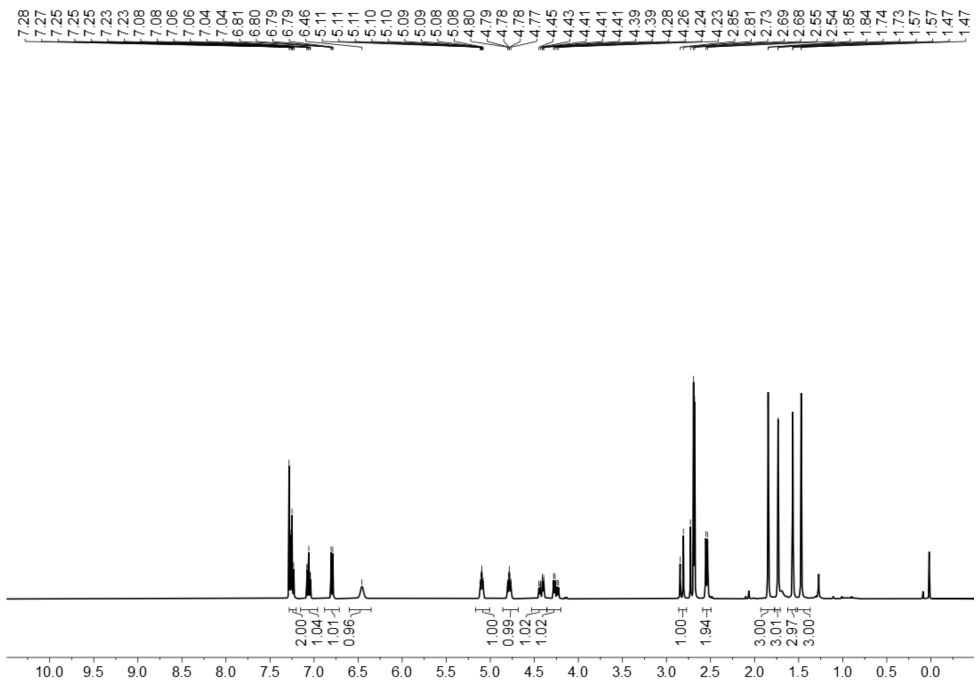

^{1}H NMR (400 MHz, $CDCl_3$)

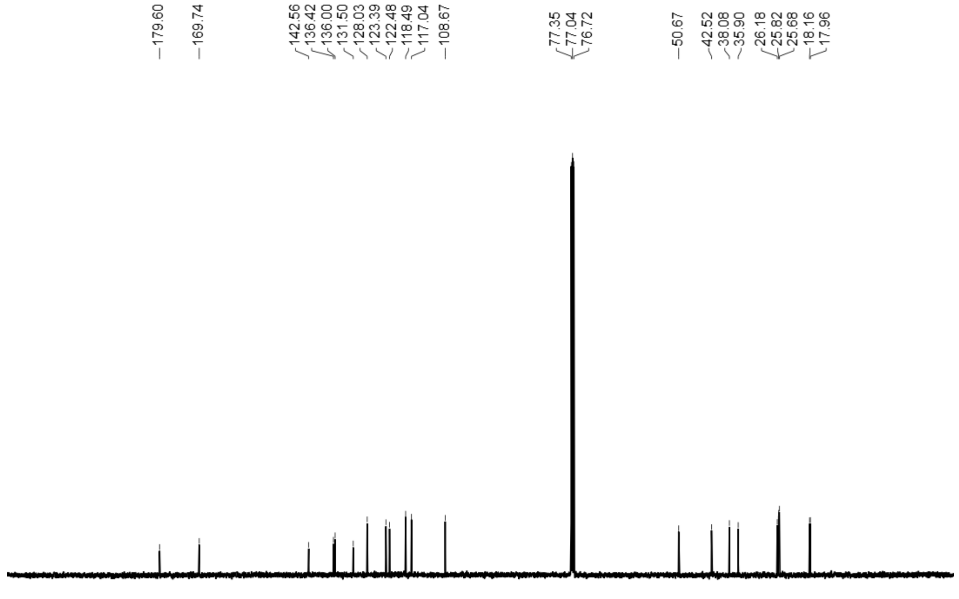

^{13}C NMR (101 MHz, $CDCl_3$)

(S)-2-(6-bromo-1,3-bis(3-methylbut-2-en-1-yl)-2-oxoindolin-3-yl)-N-methylacetamide (**7b**)

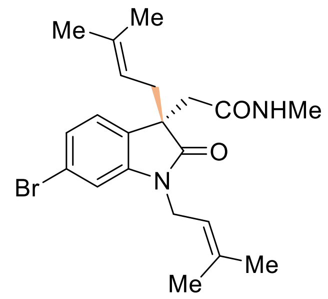

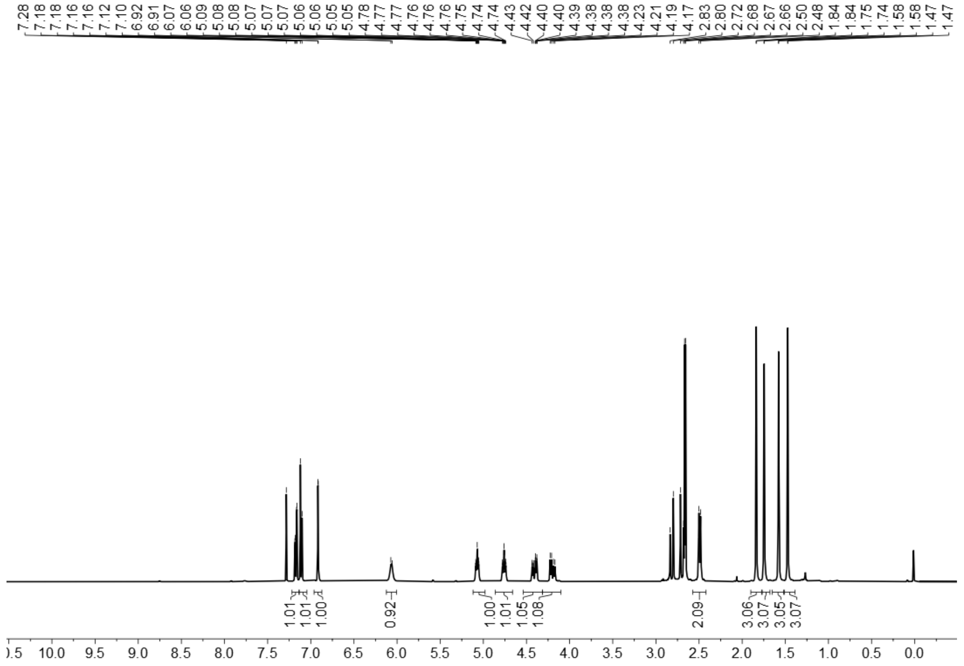

^{1}H NMR (400 MHz, $CDCl_3$)

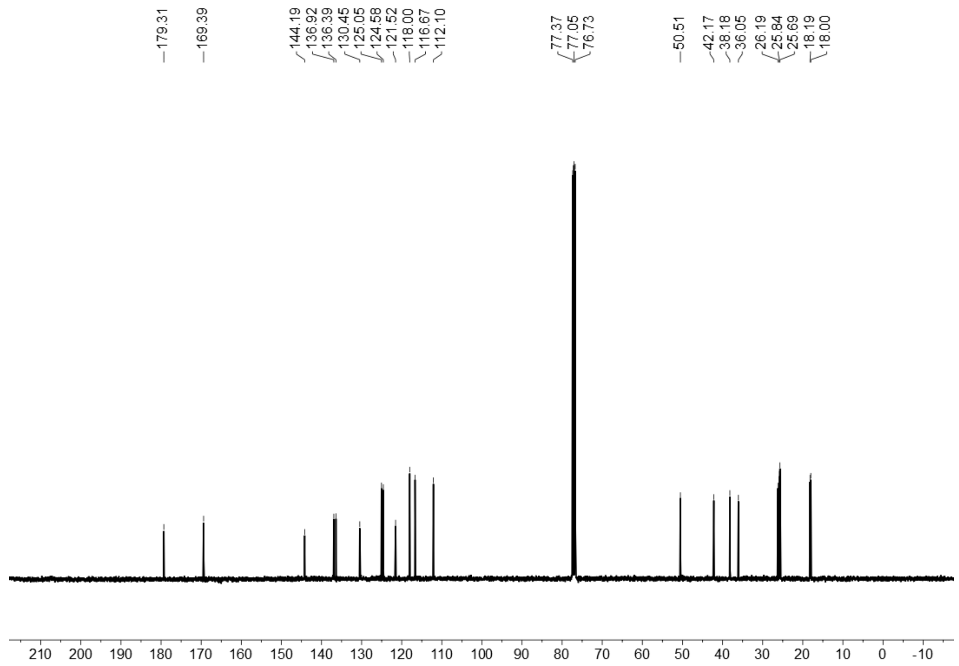

^{13}C NMR (101 MHz, $CDCl_3$)

Methyl (S,E)-2-methyl-4-(3-(2-(methylamino)-2-oxoethyl)-2-oxoindolin-3-yl)but-2-enoate (**7c**)

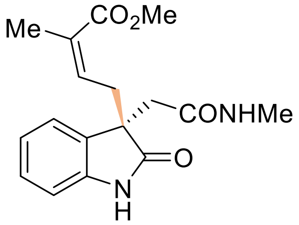

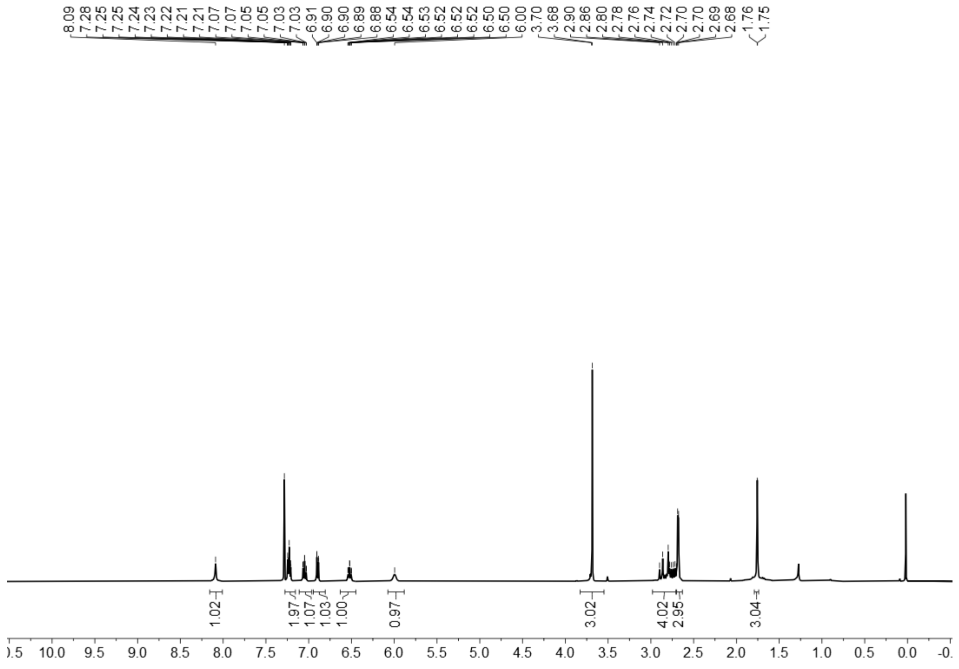

^{1}H NMR (400 MHz, $CDCl_3$)

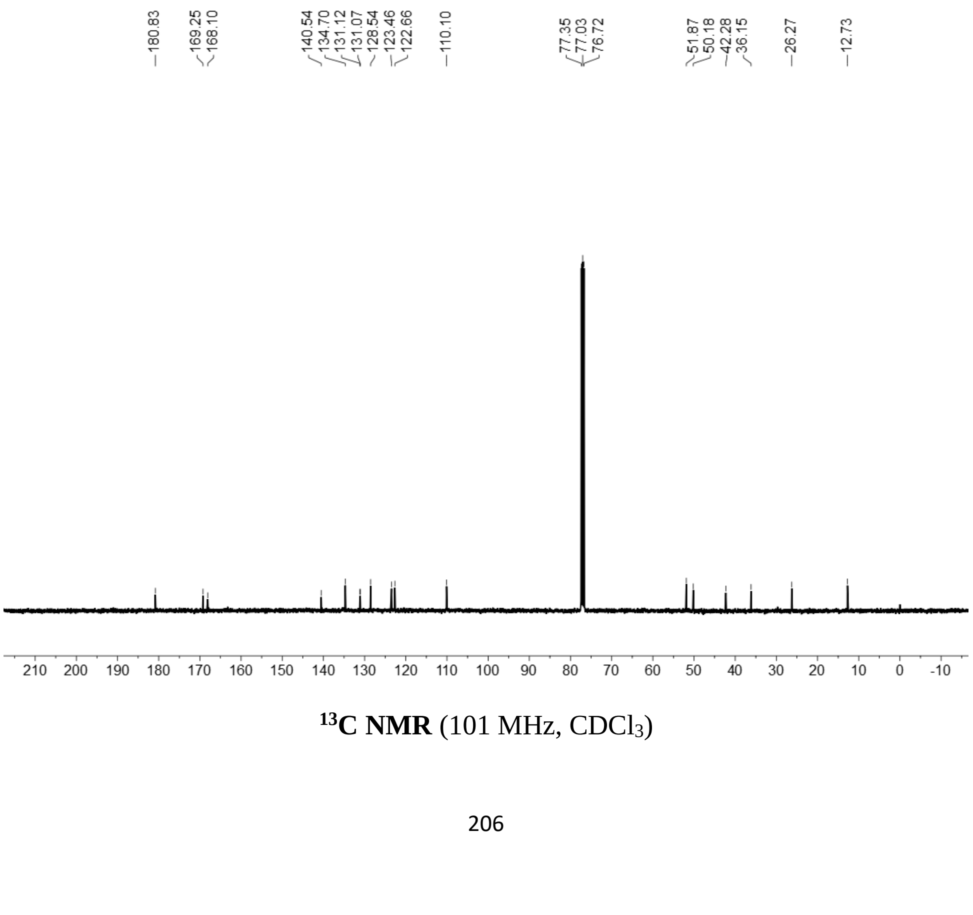

^{13}C NMR (101 MHz, $CDCl_3$)

(3aS,8aR)-1-methyl-3a,8-bis(3-methylbut-2-en-1-yl)-1,2,3,3a,8,8a-hexahydropyrrolo[2,3-b]indole (**8a**)

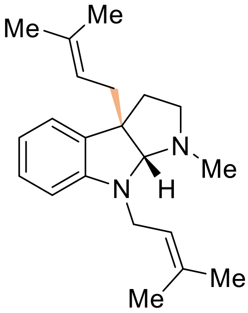

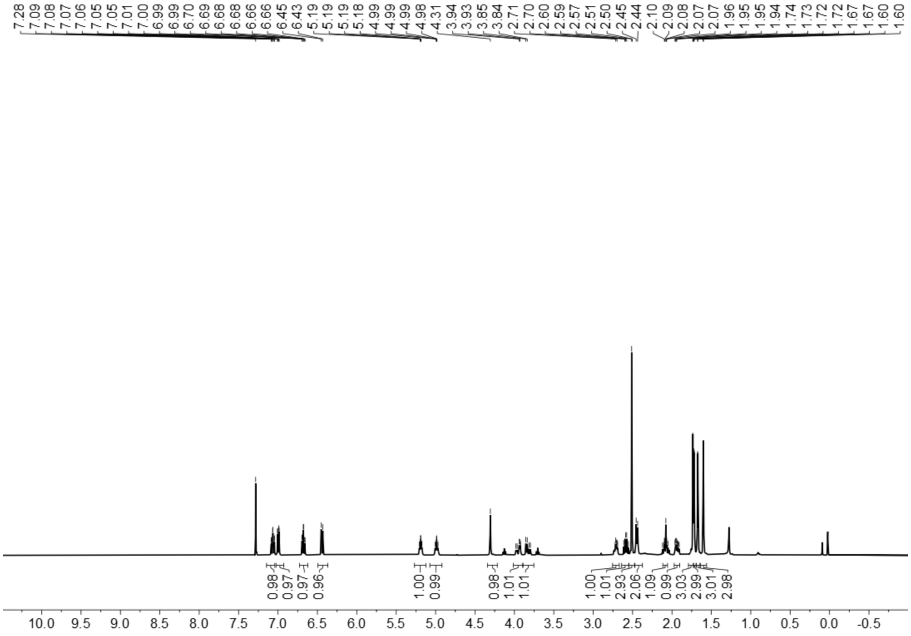

^{1}H NMR (400 MHz, $CDCl_3$)

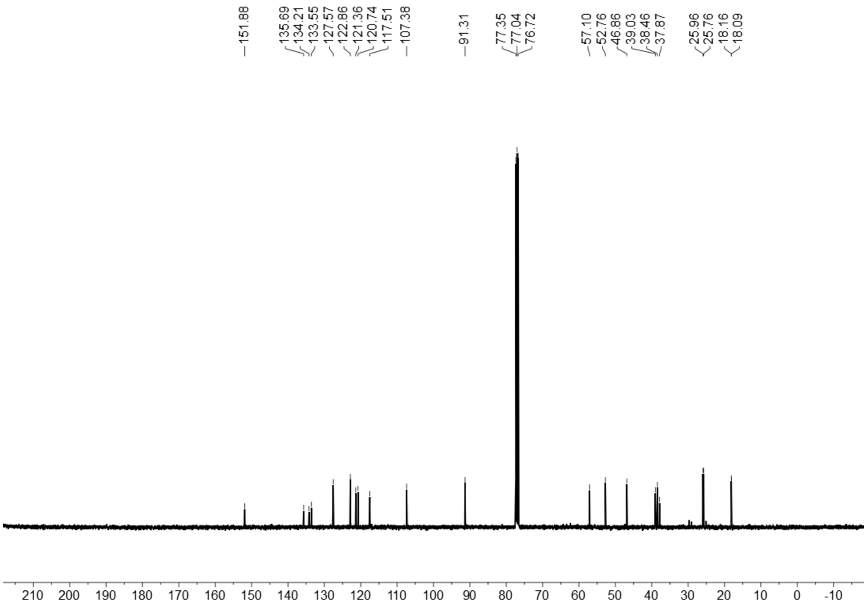

^{13}C NMR (101 MHz, $CDCl_3$)
